# A direct method to access various functional arylalkoxysilanes by Rh-catalysed intermolecular C–H silylation of alkoxysilanes†

**DOI:** 10.1039/d2sc03727k

**Published:** 2022-08-23

**Authors:** Salina Som, Jongwook Choi, Dimitris Katsoulis, Kangsang L. Lee

**Affiliations:** University of Central Florida, Department of Chemistry 4111 Libra Drive, PSB #255 Orlando FL USA 32816 k.lee@ucf.edu; Dow Chemical Company 2200 West Salzburg Road Auburn MI USA 48611

## Abstract

Efficient protocols for intermolecular C–H silylations of unactivated arenes and heteroarenes with HMe_2_SiOEt are disclosed. The silylations are catalysed by a Rh-complex (0.5 mol%) derived from commercially available [Rh(coe)_2_Cl]_2_ and (*S*,*S*)-Ph-BPE in the presence of cyclohexene at 100 °C, furnishing desired arylethoxydimethylsilanes up to 99% yield. The regioselectivity is mainly affected by the steric bulk of the substituents in arenes and by electronic effects as an ancillary factor. Mechanistic study revealed that the mono-hydrido dimeric Rh-complex, [Rh_2_(Ph-BPE)_2_(μ-H)(μ-Cl)], is an active catalytic intermediate, which further suppresses the formation of redistribution byproducts in the silylation. Preliminary results show that the current protocol can be extended to double C–H silylations affording bis-silylated arenes and is applicable to the silylation of HMeSi(OEt)_2_ to deliver the corresponding (aryl)SiMe(OEt)_2_.

## Abstract 2

The control of alkoxysilane redistribution enables the direct access of functional arylalkoxysilanes by Rh-catalyzed C–H silylations.

## Graphical abstract

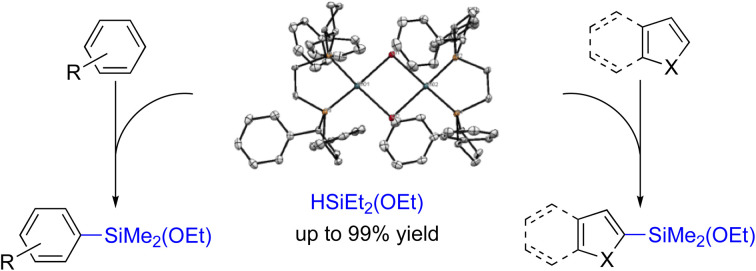

## Introduction

Among arylsilanes, arylalkoxysilanes are a highly desirable and important class of compounds, since they can be used as key monomers to produce numerous silicone polymers and materials in industry.^1^ One of the traditional methods to access arylalkoxysilanes^2^ is stoichiometric reactions between aryl Grignard or aryl lithium reagents and halo- or alkoxysilanes.^2*a*,*b*^ These methods, however, have significantly limited the scope of arenes due to the intrinsic incompatibility between such reagents and electrophilic functional groups or halogens in the aryl groups. Furthermore, these reactions are listed as ones that many industries want to avoid because of the use of non-recyclable ethereal solvents and generating a considerable amount of chemical wastes such as magnesium- or lithium salts in industrial scale production.^3^ Another industrial approach involves the alcoholysis of the products derived from benzene and chlorosilanes promoted by Lewis acid.^2*c*,*d*^ This process, however, generally results in low yield and requires repetitive distillation. Low regioselectivity with substituted arenes also remains unsolved in this process. Similar problems are also found in the direct process^2*e*^ and gas phase condensation route.^2*f*^ Due to these limitations in the industrial scale production, the arylsilanes that have been used in the silicone industry are mainly or exclusively phenyl-substituted alkoxysilanes [*e.g.*, PhSi(OEt)_3_ or Ph_2_Si(OEt)_2_], derived from the corresponding phenylchlorosilanes. In recent years, the need for more advanced (rather than just phenylsilane-based) silicone materials has continuously increased and is primarily driven by the optoelectronics and biomedical industries that desire to have improved physical properties (*e.g.*, higher refractive index, gas permeability, high thermal and UV stability, low glass transition point, *etc.*) for their product developments.^4^

**Scheme 1:**
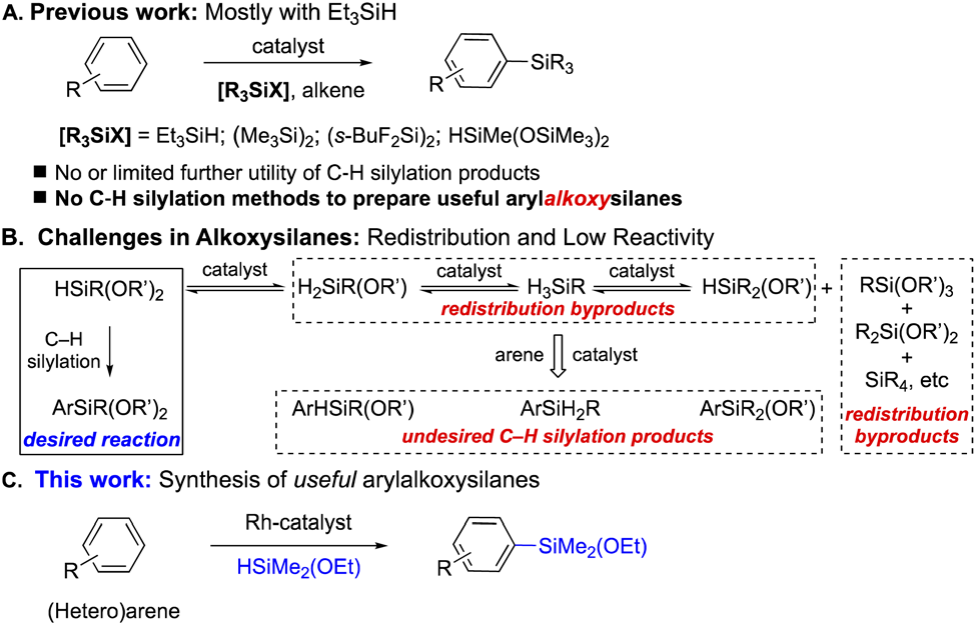
Catalytic intermolecular C–H silylation.

Transition metal-catalysed intermolecular C–H silylation of various functional arenes with alkoxysilanes can serve as an alternative and attractive direct route to synthesize arylalkoxysilanes due to its atom-economical nature with little to no chemical waste. Since the pioneering work by the Curtis group in 1982,^5^ a series of reports on C–H silylations have been published by many research groups (Scheme 1A).^6–11^ This seemingly well-established area, however, is still too far from industrial applications because the C–H silylation reactions developed so far are mostly with highly reactive trialkylsilanes to afford trialkylarylsilanes (*i.e.*, mostly Et_3_SiAr), which are unable to be utilized as monomers/reagents in silicone material synthesis due to the lack of leaving groups. Although earlier studies found that Rh- and Ir-catalysts can efficiently catalyse the silylations with a broad arene scope, these protocols are only effective with HSiMe(OSiMe_3_)_2_;^11^ the resulting arylsiloxysilanes, [aryl-SiMe(OSiMe_3_)_2_], are still inadequate to be incorporated into polymeric systems from industrial perspectives.

Compared to alkyl- or siloxysilanes, alkoxysilanes are known as challenging substrates not only because of their no/low reactivity in C–H silylations^11*b*^ (presumably due to the less hydridic H atom), but also because they often result in redistribution byproducts due to the labile nature of alkoxy moieties.^12^ In addition to the desired silylation product, other C–H silylation byproducts and new redistributed silanes can also be obtained (Scheme 1B).

Herein, we report the first direct method to synthesize various functional arylalkoxysilanes by intermolecular C–H silylations with alkoxysilanes, which were not reactive in the previous methods^13^ (Scheme 1C). The reactions are catalysed by a Rh-complex (0.5 mol%) derived from [Rh(coe)_2_Cl]_2_ and Ph-BPE to afford various functional arylalkoxysilanes in up to 99% yield with a controlled redistribution.

## Results and discussion

We set out to investigate the C–H silylation with benzene and HSiMe_2_(OEt) (1) as initial substrates (Table 1). After a series of optimization of the reaction conditions including catalyst and ligand screening,^14^ it was found that [Rh(cod)OH]_2_ with a Ph-BPE ligand in the presence of cyclohexene at 100 °C catalyses the C–H silylation to afford the desired PhSiMe_2_(OEt) (P1) in 79% yield (entry 1). A slight increase in yield was observed when [Rh(coe)_2_OH]_2_ was used (84%, entry 2). The optimal catalyst was identified as the corresponding chloride salt, [Rh(coe)_2_Cl]_2_, resulting in further increased yield (86%, entry 3). However, the use of the cyclooctadiene variant, [Rh(cod)_2_Cl]_2_, significantly decreased the yield (12%, entry 4). Rh-salts with different counter anions were not effective in this C–H silylation, furnishing low to moderate yields of P1 (entries 5–9). It is noteworthy that the redistribution of alkoxysilane 1 is a competing reaction during the desired C–H silylation; in addition to the desired product, redistribution byproducts derived from 1 were obtained in most reactions, especially when the desired silylation became sluggish. The formation of redistribution byproducts was also confirmed in control reactions: 57% of redistribution byproducts upon mixing 1 and [Rh(coe)_2_Cl]_2_ and 50% yield from 1 and complex 2.^14^

**Table 1:** Catalyst screening for Rh-catalysed C–H silylation^a^

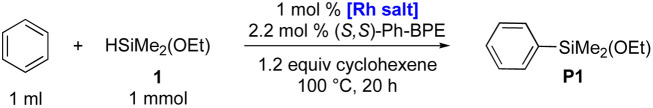			
Entry	[Rh salt]	Conv.	Yield
1	[Rh(cod)OH]_2_	>98%	79%
2	[Rh(coe)_2_OH]_2_	>98%	84%
3	**[Rh(coe)**_**2**_ **Cl]**_**2**_	**>98%**	**86%**
4	[Rh(cod)Cl]_2_	>95%	12%
5	[Rh(cod)acac]	56%	4%
6	[Rh(coe)_2_acac]	44%	28%
7	[Rh(cod)OMe]_2_	>98%	5%
8	[Rh(cod)OTf]_2_	84%	<2%
9	[Rh(nbd)_2_BF_4_]	72%	27%

a All reactions were performed under N_2_. Conversions were determined by ^1^H NMR analysis and yields were obtained after distillation.

**Scheme 2:**
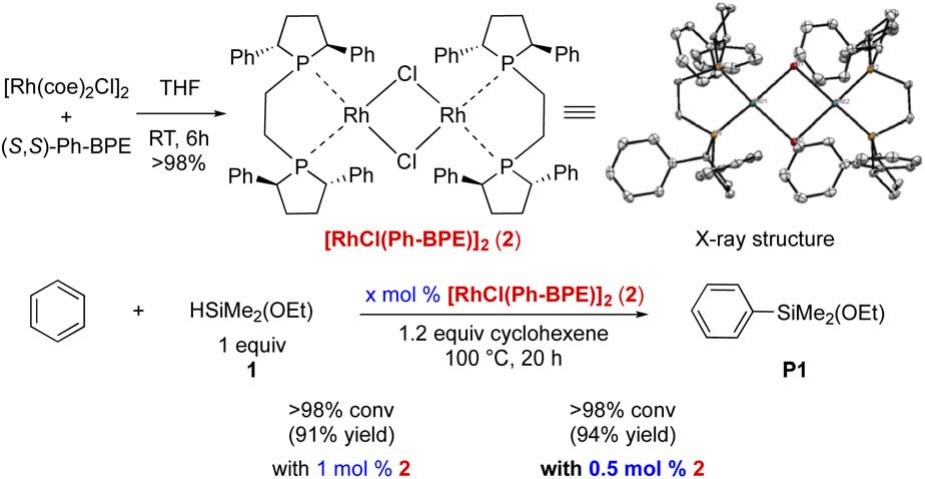
Synthesis of a well-defined Rh-complex and its catalytic activities in C–H silylation.

Next, we synthesized the corresponding preformed Rh-complex. As depicted in Scheme 2, [RhCl(Ph-BPE)]_2_ (2) was obtained in a dimeric form and characterized by X-ray crystallography. With 1 mol% of this complex 2, the silylation proceeded to afford the desired product with an increased yield (91%, *vs.* 86% in entry 3, Table 1). When the amount of catalyst 2 was decreased to 0.5 mol%, further increase in yield (94%) was observed. More importantly, by using this well-defined complex, the formation of the redistribution byproducts could be further controlled.

**Scheme 3:**
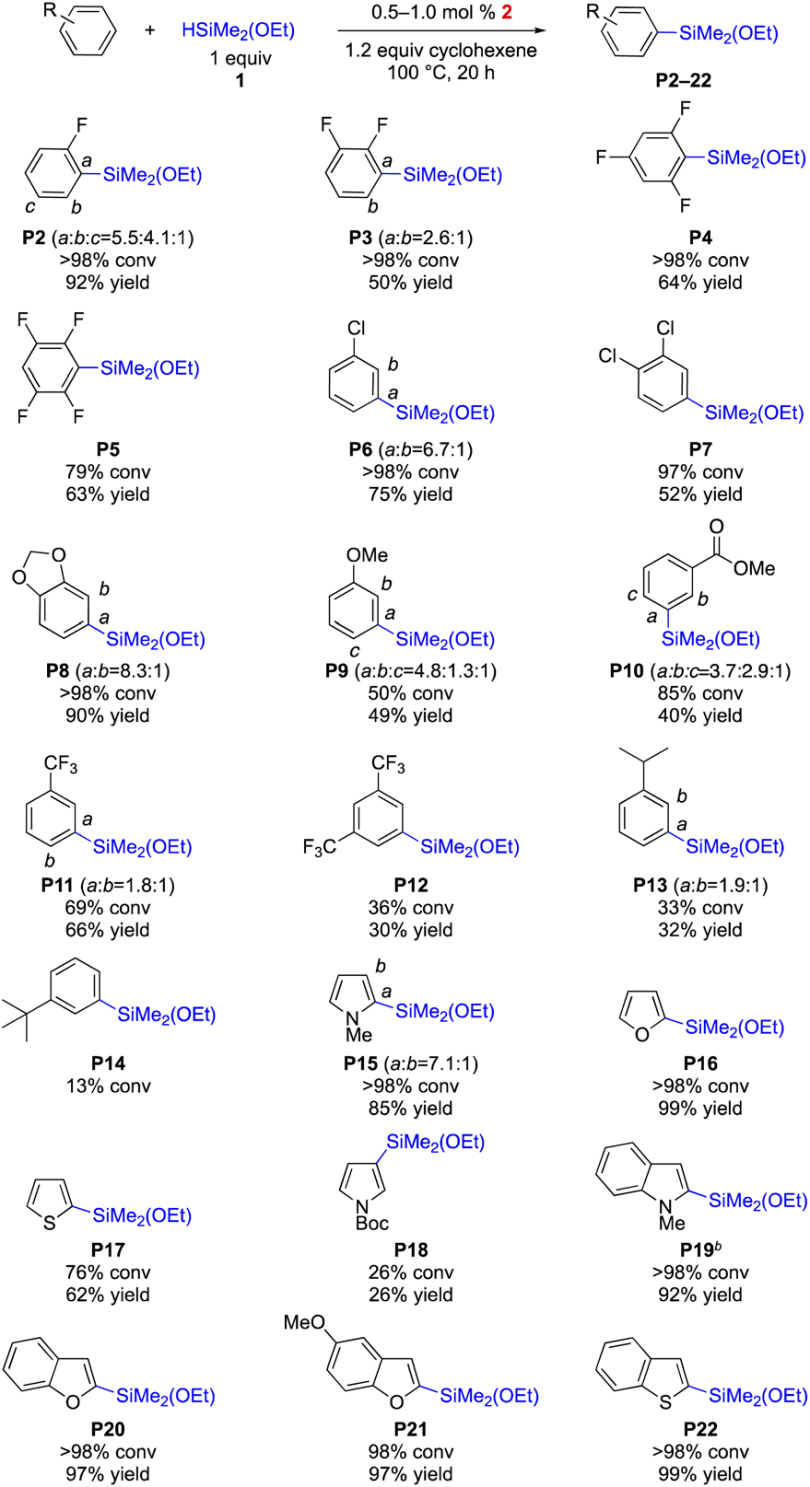
Dehydrogenative intermolecular C–H silylation of various arenes with HSiMe_2_(OEt). All reactions were performed under N_2_. Conversions were determined by ^1^H NMR analysis and yields were obtained after distillation. For the detailed reaction conditions, see the ESI.† 5 mol% of 2 was used for P13 and P14.

The efficiency of the Rh-complex (2) was tested for the C–H silylation with various arenes and heteroarenes. As shown in Scheme 3, the fluorine group is tolerated thus delivering various fluorine-containing arylethoxysilanes, which are desired compounds used to improve the physical and chemical properties of silicone materials (up to 92% yield, P2–5). Chlorobenzenes are also compatible with the catalytic system (P6–7), but the isolation of the silylation product of bromobenzene was not successful although the C–H silylation was effective. Electron-rich arenes undergo the silylations to furnish desired products P8 and P9 in up to 90% yield. The silylations of electron-deficient arenes (P10–12) are less efficient (30–66% yield). Steric hindrance is detrimental to the effectiveness of the silylation (P13–14). Heteroarenes (P15–22) are highly effective in affording arylethoxysilanes in up to 99% yield except the one with a bulky substituent (P18, 26% yield), which results in *m*-silylation (*vs. o*-silylation in P15).

For heteroarenes, this protocol is still operable without the hydrogen scavenger (cyclohexene), resulting in arylsilane P19 in 74% yield (eqn (1)). With benzene, however, this cyclohexene-free condition was less effective (28% yield, eqn (2)). The current transformation is scalable; ethoxydimethylphenylsilane P1 was isolated in a gram scale (1.66 g, 92% yield, eqn (3)).1
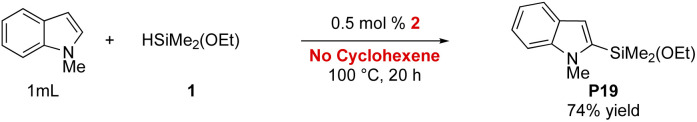
2
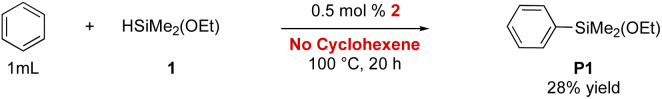
3
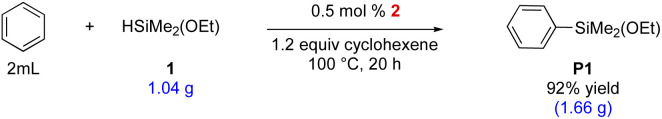


**Scheme 4:**
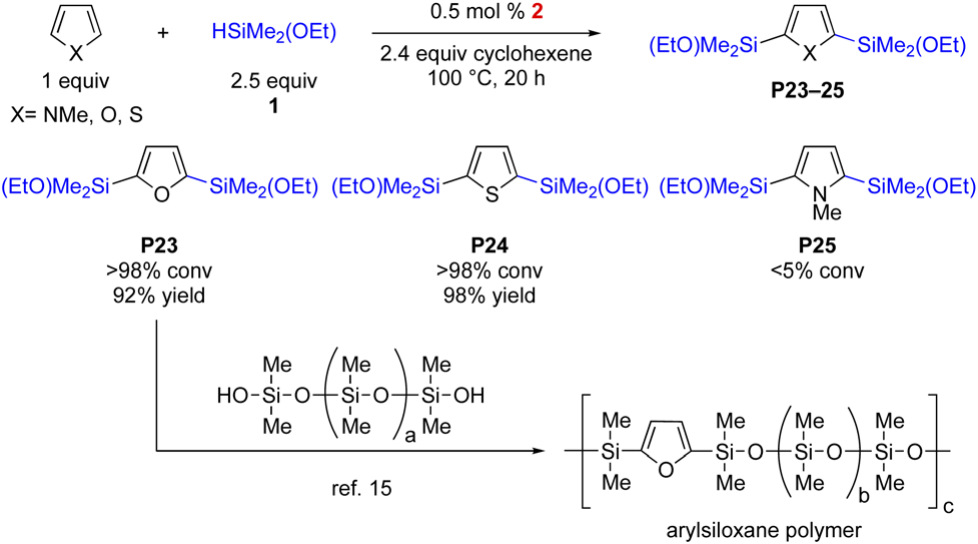
Double C–H silylations of heteroarenes. All reactions were performed under N_2_. Conversions were determined by ^1^H NMR analysis and yields were obtained after distillation.

The current protocol is also applicable toward double C–H silylation. As illustrated in Scheme 4, with excess amount of HSiMe_2_OEt (1), 0.5 mol% of complex 2 can catalyse double silylation to afford bis(ethoxydimethylsilyl)arenes in up to 98% yield (P23–24). *N*-Methylpyrrole, however, was not effective (<5%, P25). During the stepwise double silylation study, it was found that the second silylation of P15 was sluggish. The thus-obtained bis-alkoxysilanes are highly desirable monomers in the silicone industry for polysiloxane syntheses. For example, P23 can easily react with various polymeric silanols so that the aryl groups can be directly incorporated into a polymeric system.^15^

**Scheme 5:**
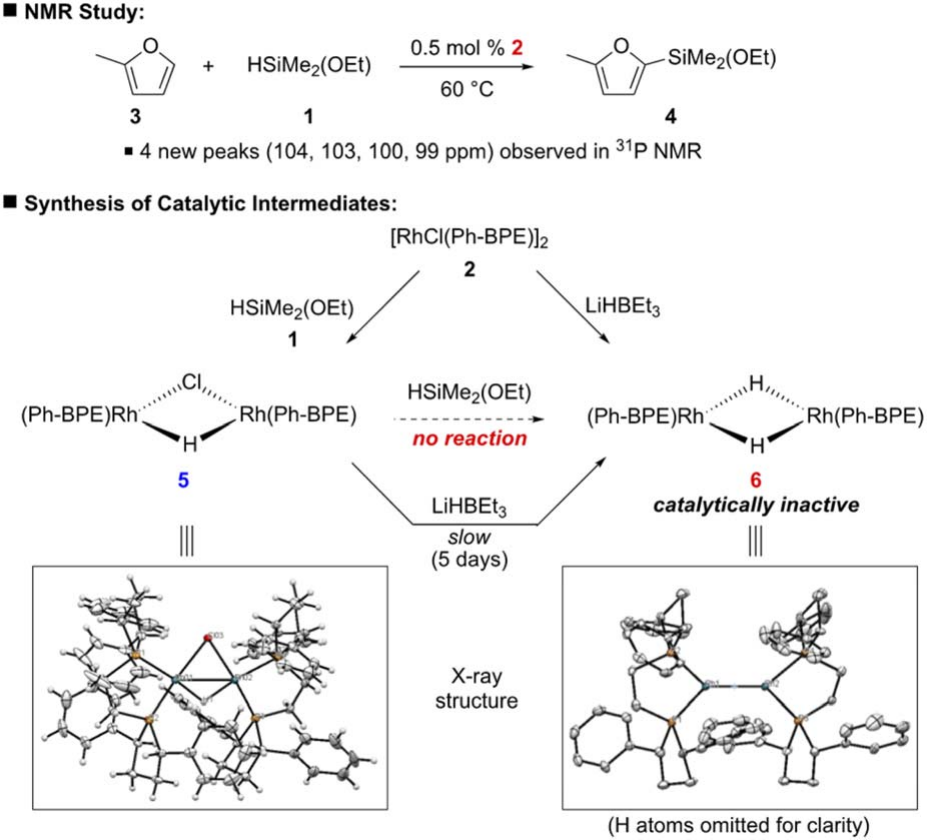
Mechanistic studies.

To have a better insight on how this C–H silylation proceeds, we investigated a few mechanistic experiments with 2-methylfuran (3), which was selected because its silylation was highly efficient to afford only the mono-silylation product (Scheme 5). First, brief kinetic experiments suggest that this silylation is aligned with the kinetic profile of 1st order in arene and catalyst, but zero order in silane 1.^14^ Next, the C–H silylation of 2-methylfuran (3) was monitored by ^31^P NMR to study the resting state of the catalyst. When the reaction mixture was heated at 60 °C for 1 h, four new broad peaks (104, 103, 100 and 99 ppm) in ^31^P NMR were observed in addition to the two peaks of 2 (106 and 105 ppm). Since this characteristic peak pattern was reminiscent of the one in [Rh_2_(dppe)_2_(μ-H)(μ-Cl)],^16^ [Rh_2_(Ph-BPE)_2_(μ-H)(μ-Cl)] (5) was independently synthesized by the reaction between 2 and 1 to confirm the formation of the mono-hydrido complex during catalytic silylation; the structure of the complex 5 was confirmed by NMR and X-ray crystallography analyses (Scheme 5).^14^

With 5 as a catalyst, the C–H silylation was performed with benzene and silane 1 under the standard conditions and afforded the desired product in high yield (97% yield), but with noticeably less amount of redistribution byproducts (eqn (4)); by using complex 5, the observed redistribution between silane 1 and complex 2 in the control experiment could be further suppressed. This improved reactivity and the ^31^P NMR study indicate that complex 5 is involved in this catalytic silylation.

We further investigated the possibility that the corresponding dihydrido complex 6, [Rh(Ph-BPE)(μ-H)]_2_, is involved. However, complex 6 could not be synthesized from the reaction between 2 or 5 and silane 1 under various reaction conditions. Gratifyingly, complex 6 was successfully synthesized with LiHBEt_3_ from 2 ^16^ and characterized by NMR and X-ray crystallography (Scheme 5). To our surprise, we observed the slow reduction of complex 5 to 6, even by LiHBEt_3_: 5 days at 60 °C. When complex 6 was subjected to the C–H silylation instead of 5, no desired product was obtained (<5% yield, eqn (4)), implying that 6 may not be involved in the catalytic cycle.4
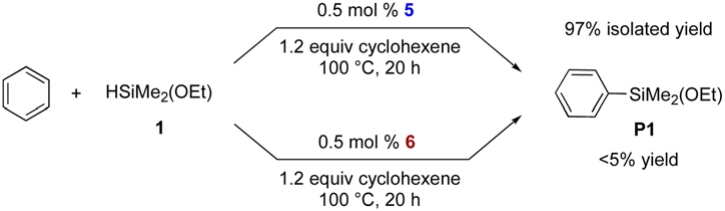


**Scheme 6:**
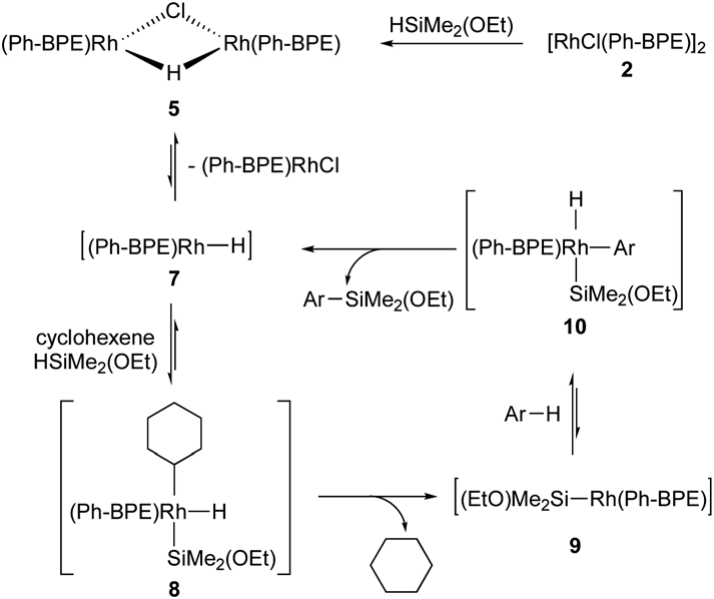
Proposed mechanism.

Based on the above results, the reaction mechanism is proposed in Scheme 6. Upon formation of complex 5 from 2 and 1, a monomeric Rh-complex 7 can be formed by disproportionation. Then it undergoes the insertion to cyclohexene and oxidative addition of the silane 1 to afford 8. After the reductive elimination to give 9, followed by the oxidative addition of arene, 10 can be obtained. Finally, the reductive elimination delivers the desired silylation product with regeneration of 7.^17^

Lastly, we investigated the feasibility of the current protocol toward C–H silylation of the more challenging dialkoxysilane, HSiMe(OEt)_2_ (11), since the resulting products, dialkoxyarylsilanes, are highly useful in developing new silicone products; functional arene-containing dialkoxysilanes can be directly used as monomers in the synthesis of polymeric materials. Our initial results are shown in eqn (5) and (6); the silylation of benzene with silane 11 was significantly slow under the standard conditions, resulting in low yield (9% yield of 12), but the silylation of *N*-methylindole was slightly more effective, affording the desired product 13 in 33% yield. The low efficiency may be attributed to the lower reactivity of HSiMe(OEt)_2_ and easier redistribution of the two ethoxy groups in 11.5
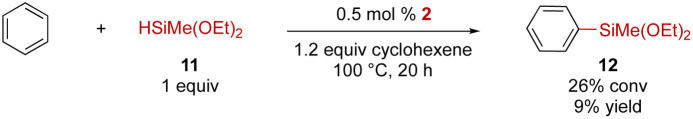
6
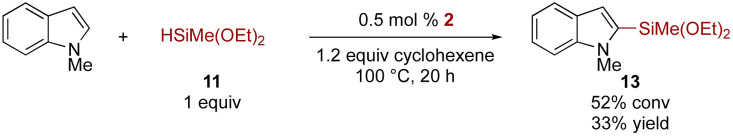


## Conclusions

In this report, we have demonstrated a direct synthetic method to access highly useful arylalkoxysilanes for silicone material synthesis. The C–H silylations between functional (hetero)arenes and HSiMe_2_OEt were catalysed by Rh-complex 2 or 5 to afford the desired arylalkoxysilanes in up to 99% yield. The control of the redistribution side reaction by catalysts was a key finding, allowing us to improve the overall catalytic efficiency and to obtain the clean products. This method is important because the silylation generates functional arylalkoxysilanes, which cannot be synthesized by other known methods. The final silylation products can be readily utilized in silicone material synthesis by incorporating various functional aryl groups into polymeric systems, so that the physical/chemical properties of polysiloxanes can be further improved. As the current catalytic protocol was less effective in the C–H silylations of HSiMe(OEt)_2_, our future efforts will be aimed toward the development of more reactive catalysts and the effective control of redistribution for the silylations of di- or trialkoxysilanes.

## Data availability

Experimental procedures and characterization data are available in the ESI.†

## Author contributions

S. S., J. C., D. K., and K. L. conceptualized the research and performed the investigation. K. L. wrote the manuscript with contributions from all authors. K. L. supervised this study.

## Conflicts of interest

The Dow Chemical Company declares the following interest: WO2018190999A1.

## Supplementary Material

SC-013-D2SC03727K-s001

SC-013-D2SC03727K-s002
