# Supplementary material for: A direct method to access various functional arylalkoxysilanes by Rh-catalysed intermolecular C–H silylation of alkoxysilanes
Source: Chem Sci. 2022 Aug 23;13(36):10759–64. doi: 10.1039/d2sc03727k (PMC9491085; doi:10.1039/d2sc03727k)
Supplement: SC-013-D2SC03727K-s001 [file SC-013-D2SC03727K-s001.pdf]

Electronic Supplementary Material (ESI) for Chemical Science.
This journal is © The Royal Society of Chemistry 2022

Direct Method to Access Various Functional Arylalkoxysilanes by Rh-catalysed Intermolecular C–H Silylation of Alkoxysilane

TBA*

Supporting Information

Table of Contents

1. **General**……………………………………………………………………..……..S2
   1.1 Reagents………………………………………………………………..…..S3
2. **Synthesis and Characterization of C–H Silylation Products**…………………………S3
   2.1 Optimization of reaction conditions……………………………………….……S3
   2.2 Redistribution Reactions in Control Experiment ……………………………………S5
   2.3 Synthesis of $[RhCl(Ph\text{-}BPE)]_2$…………………………………………………..…S6
   2.4 Representative procedure for Rh-catalyzed C–H silylation of arenes…………….….S7
   2.5 Representative procedure for Rh-catalyzed Double C–H silylation ………………..S14
3. **Synthesis and Characterization of Piers-Rubinsztajn products**……………………...S15
   3.1 Representative procedure for Piers-Rubinsztajn reaction……………………………S16
4. **Mechanistic Studies**………………………………………………………………..…S19
   4.1 Synthesis of $[RhCl(Ph\text{-}BPE)]_2$ crystals………………………………………......S19
   4.2 Synthesis of $[Rh_2(Ph\text{-}BPE)_2(\mu\text{-}Cl)(\mu\text{-}H)]$ …………………….………………….S20
   4.3 Synthesis of $[(Ph\text{-}BPE)Rh(\mu\text{-}H)]_2$……………………….……….……………….S20
   4.4 Identifying resting state of the catalyst……………………….……………………..S21
   4.5 Determination of Reaction Order in [Furan], [HSi] and [Cat] ……………………...S22
5. **NMR Spectra**……………………………………………………………………………...S25
   5.1 C–H Silylation Products…………………….…………………………………..…S25
   5.2 Double C–H Silylation Products……………………..……………………………S52
   5.3 Siloxane Products……………………..…………….……………………………..S54
   5.4 Rh complexes……………………….…………………….…………………….....S64
6. **Crystallographic Analyses**………………………….………………………………..S67
   6.1 $[RhCl(Ph\text{-}BPE)]_2$……………………….……………………………………………S67
   6.2 $[Rh_2(Ph\text{-}BPE)_2(\mu\text{-}Cl)(\mu\text{-}H)]$ ……………………………….……………………..S95
   6.3 $[(Ph\text{-}BPE)Rh(\mu\text{-}H)]_2$………….……….………………………………………...S119
7. **References**……………………………………………………….…………………….…S137

1. General:

^{1}H NMR spectra were recorded on a Bruker Advance II HD 400 (400 MHz) spectrometer and Varian Unity INOVA 400 (400 MHz) spectrometer. Chemical shifts are reported in ppm with the solvent resonance as the internal standard ($CDCl_3$: 7.26 ppm). Data are reported as follows: chemical shift, integration, multiplicity (s = singlet, d = doublet, t = triplet, q = quartet, p = pentet, sext = sextet, hept = heptet, m = multiplet, br = broad), and coupling constants (*J*, Hz). ^{13}C NMR spectra were recorded on a Bruker Advance III HD 400 (100 MHz) and Varian Unity INOVA 400 (100 MHz) spectrometer with complete proton decoupling. Chemical shifts are reported in ppm with the solvent resonance as the internal standard ($CDCl_3$: 77.16 ppm). ^{19}F NMR spectra were recorded on a Bruker Advance III HD 400 (376 MHz) spectrometer without proton decoupling. High-resolution mass spectrometry was performed on GC-MS systems consisting of an HP 6890 GC with a HP 5973 MSD and an Thermo 1300 Trace GC with a Q-Exactive Orbitrap MS.

Unless otherwise noted, all reactions were carried out with distilled and degassed solvents under an atmosphere of dry N_2 in oven- (135 °C) or flame-dried glassware with standard dry box or vacuum-line techniques. All air and moisture-sensitive experiments, work-up, and purification procedures were carried out in a N_2-filled glove box. Solvents were purified under a positive pressure of dry argon by a two-column solid-state purification system (JC Meyer Solvent System, Irvine, CA) and stored over activated 4 Å molecular sieves. $CDCl_3$ (Aldrich) was distilled and stored over activated 4 Å molecular sieves prior to use. 25mm 0.2 µm PTFE Syringe filters from VWR were used in workup procedures.

1.1 Reagents:

Rh catalysts and phosphine ligands were purchased from Strem and used as received.

5-Methoxybenzofuran (Aldrich), Thianapthene (Aldrich), Alumina Oxide (Alfa Aesar), Tris(pentafluorophenyl)borane (Strem), and Lithium triethylborohydride in THF (Aldrich) were used as received.

Benzene (Aldrich), Dimethylethoxysilane (Gelest), Methyldiethoxysilane (Gelest), 1,3-Bis(trifluoromethyl)benzene (Oakwood), 1,3-Benzodioxole (Oakwood), Anisole (Aldrich), Fluorobenzene (Oakwood), Chlorobenzene (Oakwood), 1,2-Dichlorobenzene (Oakwood), 1,2-Difluorobenzene (Aldrich), 1,3,5-Trifluorobenzene (Aldrich), 1,2,4,5-Tetrafluorobenzene

(Aldrich), 1-Methylpyrrole (Oakwood), Furan (Aldrich), Thiophene (Aldrich), Cumene (Aldrich), *N*-Boc-pyrrole (Aldrich), 1-Methylindole (Oakwood), Benzofuran (Oakwood), Methyl benzoate (Aldrich), Benzotrifluoride (Alrich), Cumene (Aldrich), 2-Methylfuran (Aldrich), Methyl thiophene-2-carboxylate (Synthonix), Phenyldimethyethoxysilane (Gelest), and Pentamethyl-disiloxane (Gelest) were distilled prior to use.

2. Synthesis and Characterization of C—H Silylation Products

2.1 Optimization of reaction conditions:

Table S1. Ligand screening[a]

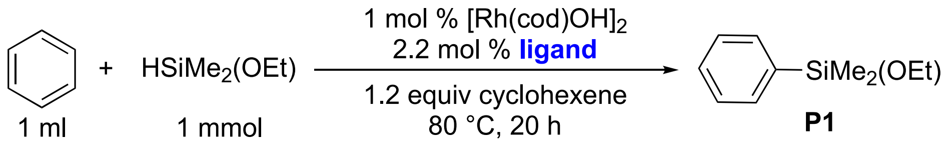

entry	[Rh]	ligand	conv	yield
1	$[Rh(cod)OH]_2$	(*S*,*S*)-Et-DuPhos	70%	7%
2	$[Rh(cod)OH]_2$	(*S*,*S*)-*i*-Pr-DuPhos	71%	5%
3	[Rh (Me-DuPhos)cod]BF_4		61%	<2%
4	[Rh (Et-DuPhos)cod]BF_4		66%	<2%
5	[Rh (*i*-Pr-DuPhos)cod]BF_4		56%	9%
6	$[Rh(cod)OH]_2$	(*S*,*S*)-*i*-Pr-BPE	39%	12%
7	[Rh (*i*-Pr-BPE)cod]BF_4		68%	28%
8	$[Rh(cod)OH]_2$	(*S*,*S*)-Ph-BPE	88%	56%
9[b]	$[Rh(cod)OH]_2$	(*S*,*S*)-Ph-BPE	>98%	79%

[a] All reactions were performed under N_2. Yields were determined by ^1H NMR analysis.
[b] at 100 °C.

Table S2. Additive screening[a]

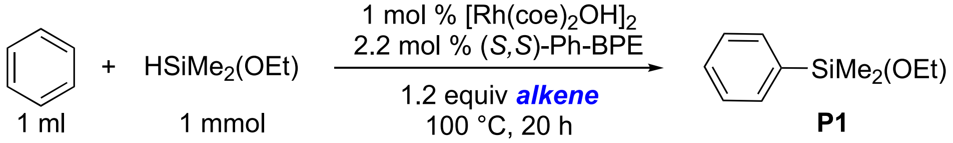

entry	alkene	conv	yield	entry	alkene	conv	yield	
1	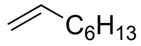	>98%	6%	6	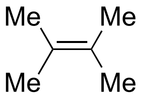	>98%	66%	
2	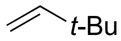	>98%	46%	7	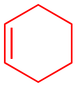	>98%	84%	**$7-28/mol**
3	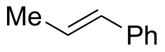	94%	51%	8	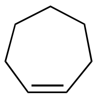	>98%	79%	
4	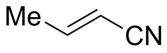	63%	<2%	9	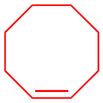	>98%	93%	**$267/mol**
5	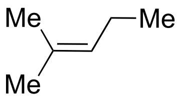	77%	11%	10	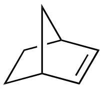	>98%	69%	

[a] All reactions were performed under N_2. Yields were determined by ^1H NMR analysis.

Table S3. Ligand loading[a]

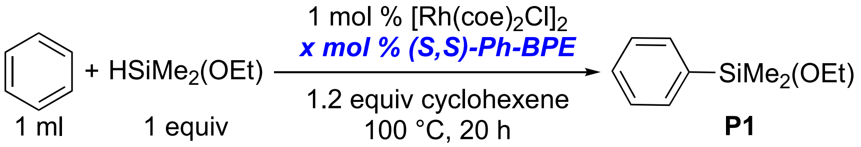

1 mol % Ph-BPE 82% conv, 9% **P1**

2 mol % Ph-BPE >98% conv, 81% **P1**

3 mol % Ph-BPE >98% conv, 85% **P1**

4 mol % Ph-BPE 55% conv, 20% **P1**

[a] All reactions were performed under N_2. Yields were determined by ^1H NMR analysis.

Table S4. Screening of reaction temperature[a]

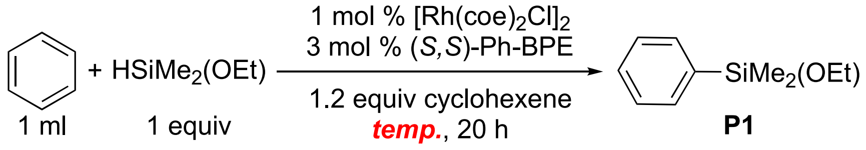

22 °C	19% conv, <2% **P1**
50 °C	20% conv, 5% **P1**
80 °C	59% conv, 32% **P1**
100 °C	>98% conv, 87% **P1**

[a] All reactions were performed under N_2. Yields were determined by ^1H NMR analysis.

2.2 Redistribution Reactions in Control Experiment:

Table S5: Control Experiment[a]

$HSiMe_2(OEt)$ (1 equiv) —[0.5 mol % **X**; 100 °C, 1 h]→

X	
$[RhCl(Ph\text{-}BPE)]_2$	50% redistribution byproducts
$[Rh(coe)_2Cl]_2$	57% redistribution byproducts
(*S*,*S*)-Ph-BPE	no redistribution byproducts observed

[a] All reactions were performed under N_2. Yields were determined by ^1H NMR analysis.

In a N_2-filled glovebox, an oven-dried pressure tube was charged with $[RhCl(Ph\text{-}BPE)]_2$ (**2**, 6.7 mg, 0.5 mol %) and $HSiMe_2(OEt)$ (**1**, 104 mg, 1 mmol). After stirring for 1 h at 100 °C in an oil bath, the reaction mixture was cooled to 22 °C. Conversion of **1** to redistribution byproducts were monitored by ^1H NMR. Note that due to the high volatility of some expected byproducts, the percentile of the observed redistribution is an approximate value based on ^1H NMR analysis.

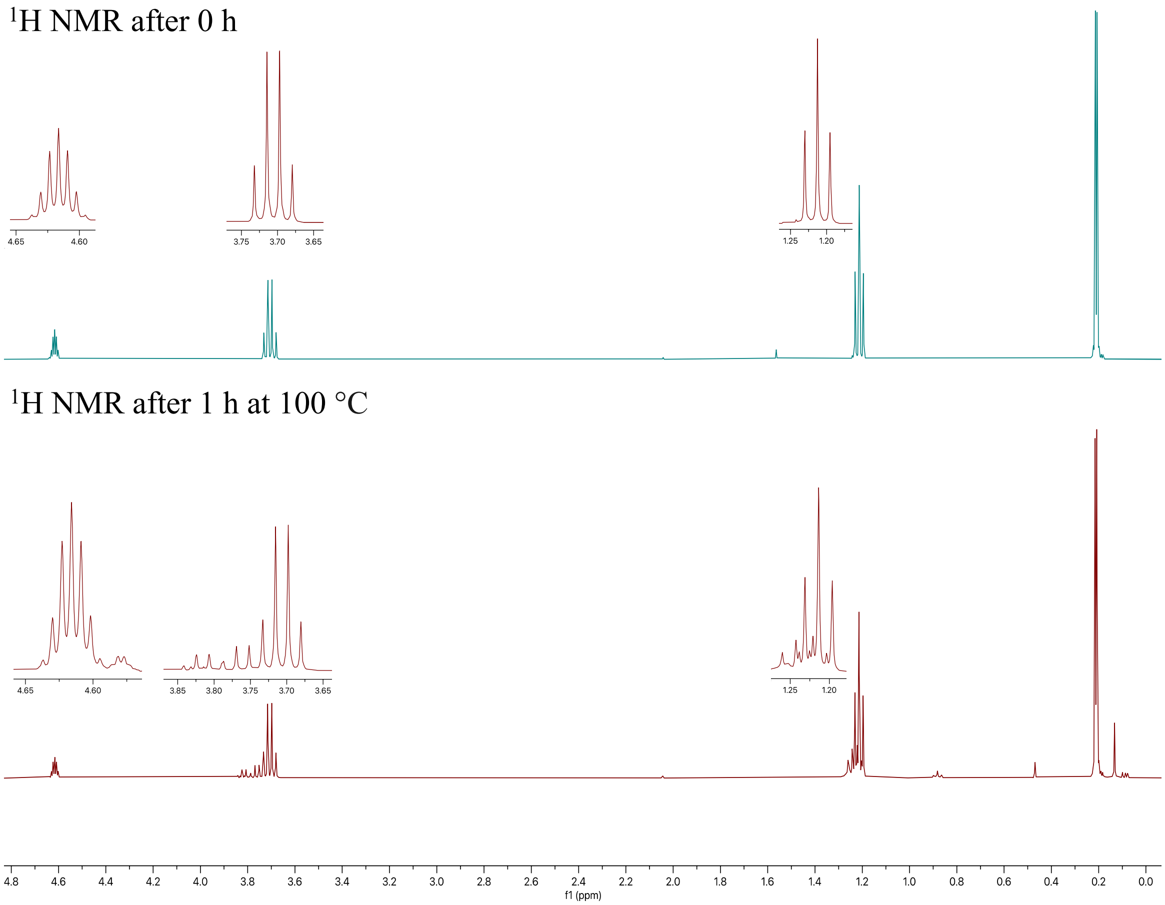

2.3 Synthesis of [RhCl(Ph-BPE)]$_2$ (2, Scheme 2):

In a N_2-filled glovebox, an oven-dried vial was charged with [Rh(coe)$_2$Cl]$_2$ (172 mg, 0.24 mmol, 1 equiv.), (*S*,*S*)-Ph-BPE (267 mg, 0.53 mmol, 2.2 equiv.), and THF (40 ml). After 6 h of stirring at 22 °C, the reaction mixture was filtered through a syringe filter and a clear orange solution was obtained. The volume of the resulting solution was decreased to one third and then hexane was added. Orangish-yellow solid form of [RhCl(Ph-BPE)]$_2$ (**2**) was obtained after trituration with hexane (>86% isolated yield). The resulting solid was collected by filtration and washed with hexane. ^{1}H NMR (400 MHz, $CDCl_3$): δ 7.98 (d, 8H, *J* = 7.4 Hz), 7.43 (t, 8H, *J* = 6.8 Hz), 7.35–7.31 (m, 4H), 7.16–7.01 (m, 20H), 4.41–3.35 (m, 4H), 72.59–2.52 (m, 4H), 2.46–2.36 (m, 4H),

2.03–1.84 (m, 8H), 1.63–1.53 (m, 4H), 0.50–0.26 (m, 8H); ^{31}P NMR ($CDCl_3$): δ 106.0, 104.8. X-ray data is shown at the end of SI.

2.4 Representative procedure for Rh-catalyzed C−H silylation of arenes (Scheme 2 and 3):

In a N_2-filled glovebox, an oven-dried pressure tube was charged with [RhCl(Ph-BPE)]$_2$ (**2**, 6.7 mg, 0.5 mol %) and benzene (1 ml). After stirring for 1 h at 100 °C in an oil bath, the reaction mixture was cooled to 22 °C. To the mixture, cyclohexene (99 mg, 1.2 equiv.) and $HSiMe_2(OEt)$ (**1**, 104 mg, 1 mmol) was added. The reaction mixture was allowed to stir for 20 h at 100 °C (>98% conversion, 94% yield by ^{1}H NMR analysis). *Note that the product is slightly decomposed on silica gels. Higher isolated yield was obtained after following workup procedure in a N_2-filled glovebox:* evaporation of the crude mixture, diluting the residue with hexane, and filtration through a syringe filter by rinsing with hexane. The filtrate was then purified by vacuum distillation affording the desired product **P1** (94% isolated yield).

When all workup procedures were performed outside of the glovebox, a lower isolated yield was obtained; the resulting mixture was concentrated and purified by silica gel chromatography affording the desired product **P1**[1] as a colorless liquid (88 mg, 49% isolated yield). ^{1}H NMR (400 MHz, $CDCl_3$): δ 7.6–7.58 (m, 2H), 7.41–7.35 (m, 3H), 3.68 (q, 2H, J = 7.0 Hz), 1.19 (t, 3H, J = 7.0 Hz), 0.39 (s, 6H). Spectroscopic data were consistent with those previously reported.[1]

Ethoxy(2-fluorophenyl)dimethylsilane and regioisomers (P2)

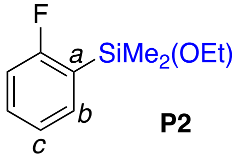

P2

Modified procedure: 0.5 mol % [RhCl(Ph-BPE)]$_2$ (6.4 mg, 0.005 mmol) and fluorobenzene (1 ml, 10.7 mmol). Complex **P2** was obtained in a mixture (*a:b:c*=5.5:4.1:1) [>98% conv., 92% isolated yield (183 mg)]. ^{1}H NMR (400 MHz, $CDCl_3$): ^{1}H NMR (400 MHz, $CDCl_3$) δ 7.59–7.53 (m, 0.36H), 7.53–7.46 (m, 0.98H), 7.42–7.30 (m, 6.36H), 7.24 (d, 0.36H, J = 2.9 Hz), 7.15 (t, 1H, J = 7.3 Hz), 7.11–7.04 (m, 1.24H), 7.00 (t, 0.93H, J = 8.4 Hz), 3.74 (q, 2H, J = 7.0 Hz), 3.68 (q, 1.90H, J = 7.0 Hz), 1.26–1.14 (m, 5.89H), 0.42 (s, 4.52H), 0.38 (s, 4.52H), 0.37 (s, 1.09H); ^{13}C NMR (101 MHz, $CDCl_3$): δ 167.20 (d, J = 241.4 Hz), 164.14 (d, J = 248.5 Hz), 162.78 (d, J = 248.3 Hz), 141.35 (d, J = 3.8 Hz), 135.58 (d, J = 7.4 Hz), 135.52 (d, J = 11.1 Hz), 133.77 (d, J = 3.7 Hz), 131.97 (d, J = 8.4 Hz), 129.79 (d, J = 6.8 Hz), 129.05 (d, J = 3.1 Hz), 124.31 (d, J = 30.0 Hz), 123.97 (d, J = 2.9 Hz), 119.88 (d, J = 18.4 Hz), 116.55 (d, J = 21.1 Hz), 115.10 (d, J = 19.6 Hz), 114.92 (d, J = 25.4 Hz), 58.96, 58.88, 58.77,

18.51, -0.91 (d, J = 1.8 Hz), -1.55, -1.68; ^{19}F NMR (376 MHz, $CDCl_3$): δ -100.72– -100.92 (m), -111.17– -111.32 (m), -113.49– -113.81 (m); HRMS (EI+): Calcd for $[C_9H_{12}FOSi]^+$ $[M–Me]^+$: 183.0636, found: 183.0634.

(2,3-difluorophenyl)ethoxydimethylsilane and regioisomer (P3)

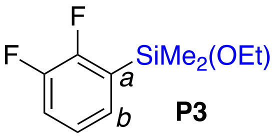

Modified procedure: 1 mol % $[RhCl(Ph\text{-}BPE)]_2$ (12.9 mg, 0.01 mmol) and 1,2-difluorobenzene (1 ml, 10.2 mmol). Complex **P3** was obtained in a mixture (*a:b*=2.6:1). The reaction was clean, but some of the desired product was lost during the workup distillation resulting in low isolated yield [>98% conv., 50% isolated yield (109 mg)]. ^{1}H NMR (400 MHz, $CDCl_3$): δ 7.35 (ddd, 0.43H, J = 10.2, 8.4, 1.5 Hz), 7.30–7.27 (m, 0.32H), 7.26–7.20 (m, 1H), 7.17 (m, 1.35H), 7.09 (m, 1.06H), 3.75 (q, 2H, , J = 7.0 Hz), 3.68 (q, 0.77H, J = 7.0 Hz), 1.21 (dt, 4.19H, J = 12.3, 7.0 Hz), 0.44 (s, 6.15H), 0.37 (s, 2.33H); ^{13}C NMR (101 MHz, $CDCl_3$) δ 154.18 (dd, J = 242.1, 11.0 Hz), 151.54 (dd, J = 250.5, 12.6 Hz), 150.51 (dd, J = 250.6, 12.0 Hz), 150.31 (dd, J = 250.3, 16.2 Hz), 135.73 (dd, J = 4.7, 2.5 Hz), 130.04 – 129.80 (m), 129.79 (dd, J = 9.9, 4.4 Hz), 127.47 (dd, J = 26.4, 1.8 Hz), 125.79 (d, J = 68.4 Hz), 124.70 (dd, J = 5.3, 3.5 Hz), 122.11 (d, J = 14.5 Hz), 118.96 (d, J = 17.2 Hz), 117.31 (d, J = 16.0 Hz), 59.09, 58.90, 18.51, 18.49, -0.88 (d, J = 1.1 Hz), -1.63; ^{19}F NMR (376 MHz, $CDCl_3$): δ -127.75 (ddd, J = 24.0, 8.1, 4.0 Hz), -136.42– -137.43 (m), -138.98 (dddd, J = 24.1, 10.4, 4.2, 1.2 Hz), -139.23 (ddd, J = 20.4, 10.4, 7.5 Hz); HRMS (CI): Calcd for $[C_{10}H_{14}F_2OSi]^+$ $[M+H]^+$: 217.0855, found: 217.0855.

Ethoxydimethyl(2,4,6-trifluorophenyl)silane (P4)

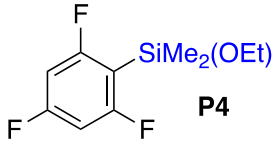

Modified procedure: 1.7 mol % $[RhCl(Ph\text{-}BPE)]_2$ (12.9 mg, 0.01 mmol), 1,3,5-trifluorobenzene (755 mg, 5.7 mmol), cyclohexene (59 mg, 0.72 mmol), and $HSiMe_2(OEt)$ (**1**, 63 mg, 0.6 mmol). The reaction was clean, but some of the desired product was lost during the workup distillation resulting in low isolated yield [>98% conv., 64% isolated yield (90 mg)]. ^{1}H NMR (400 MHz, $CDCl_3$): δ 6.66–6.54 (m, 1H), 3.72 (q, 2H, J = 7.0 Hz), 1.19 (t, 3H, J = 7.0 Hz), 0.46 (t, 6H, J = 1.8 Hz); ^{13}C NMR (101 MHz, $CDCl_3$): δ 167.50 (ddd, J = 245.8, 19.2, 14.9 Hz), 164.91 (dt, J = 250.8, 16.1 Hz), 108.09 (td, J = 35.3, 4.2 Hz), 100.19 (ddd, J = 31.9, 24.4, 3.9 Hz), 59.14, 18.34, 0.41 (t, J = 3.0 Hz); ^{19}F NMR (376 MHz, $CDCl_3$): δ -94.81– -94.98 (m), -105.82 (p, J = 9.0 Hz); HRMS (CI): Calcd for $[C_{10}H_{13}F_3OSi]^+$ $[M+H]^+$: 235.0761, found: 235.0761.

Ethoxydimethyl(2,3,5,6-tetrafluorophenyl)silane (P5)

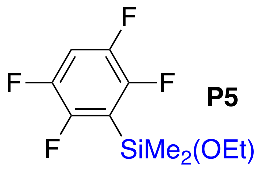

P5

Modified procedure: 1 mol % $[RhCl(Ph\text{-}BPE)]_2$ (12.9 mg, 0.01 mmol) and 1,2,4,5-tetrafluorobenzene (1.35 g, 9 mmol) [79% conv., 63% isolated yield (160 mg)]. ^{1}H NMR (400 MHz, $CDCl_3$): δ 7.06 (tt, 1H, *J* = 9.3, 7.4 Hz), 3.75 (q, 2H, *J* = 7.0 Hz), 1.21 (t, 3H, , *J* = 7.0 Hz), 0.51 (t, 6H, *J* = 1.8 Hz); ^{13}C NMR (101 MHz, $CDCl_3$): δ 148.79 (dtd, *J* = 242.1, 13.0, 3.7 Hz), 147.36–144.23 (m), 116.22 (tt, *J* = 32.2, 2.0 Hz), 108.04 (tt, *J* = 22.7, 1.9 Hz), 59.41, 18.30, 0.29 (t, *J* = 2.8 Hz); ^{19}F NMR (376 MHz, $CDCl_3$): δ -128.87– -129.05 (m), -138.74– -139.12 (m); HRMS (CI): Calcd for $[C_{10}H_{12}F_4OSi]^+$ $[M+H]^+$: 253.0666, found: 253.0667

(3-Chlorophenyl)ethoxydimethylsilane and regioisomer (P6)

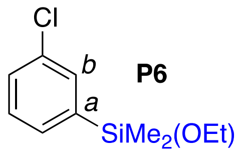

P6

Modified procedure: 1 mol % $[RhCl(Ph\text{-}BPE)]_2$ (12.9 mg, 0.01 mmol) and chlorobenzene (2 mmol). Complex **P3** was obtained in a mixture (*a*:*b*=6.7:1) [>98% conv., 75% isolated yield (161 mg)]. ^{1}H NMR (400 MHz, $CDCl_3$): δ 7.65–7.56 (m, 0.35H), 7.55–7.50 (m, 1H), 7.49 (t, 0.16H, *J* = 1.9 Hz), 7.44 (dt, 1H, *J* = 7.0, 1.2 Hz), 7.40–7.27 (m, 2.18H), 3.87–3.75 (m, 0.30H), 3.68 (q, 2H, *J* = 6.9 Hz), 1.26–1.15 (m, 3.50H), 0.38 (s, 6H), 0.37 (s, 0.97H); ^{13}C NMR (101 MHz, $CDCl_3$): δ 140.96, 140.37, 136.51, 136.31, 136.00, 134.98, 134.45, 134.41, 133.60, 133.36, 131.49, 129.73, 129.49, 128.22, 127.96, 58.93, 18.45, -1.59, -1.63; HRMS (EI+) calcd for $[C_9H_{12}ClOSi]^+$ $[M–Me]^+$: 199.0341, found: 199.0339.

(3,4-Dichlorophenyl)ethoxydimethylsilane (P7)

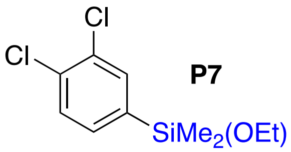

P7

Modified procedure: 1 mol % $[RhCl(Ph\text{-}BPE)]_2$ (12.9 mg, 0.01 mmol) and 1,2-dichlorobenzene (1 ml, 9 mmol). The reaction was clean, but some of the desired product was lost during the workup distillation resulting in low isolated yield [>98% conv., 52% isolated yield (129 mg)]. ^{1}H NMR (400 MHz, $CDCl_3$): δ 7.62 (d, 1H, *J* = 1.4 Hz), 7.45 (d, 1H, *J* = 7.8 Hz), 7.37 (dd, 1H, *J* = 7.8, 1.4 Hz), 3.68 (q, 4H, *J* = 7.0 Hz), 1.20 (t, 3H *J* = 7.0 Hz), 0.38 (s, 6H); ^{13}C NMR (101 MHz, $CDCl_3$): δ 139.08, 135.36, 133.94, 132.66, 132.62, 130.27, 58.96, 18.53, -1.64; HRMS (CI): Calcd for $[C_{10}H_{14}Cl_2OSi]^+$ $[M+H]^+$: 249.0264, found: 249.0265.

Benzo[*d*][1,3]dioxol-5-yl(ethoxy)dimethylsilane and regioisomer (P8)

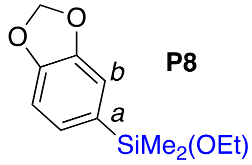

P8

Modified procedure: 1 mol % $[RhCl(Ph\text{-}BPE)]_2$ (12.9 mg, 0.01 mmol) and 1,3-Benzodioxole (1 ml, 8.7 mmol). Complex **P8** was obtained in a mixture (*a:b*=8.3:1) [>98% conv., 90% isolated yield (201 mg)]. ^{1}H NMR (400 MHz, $CDCl_3$): δ 7.10–7.03 (m, 0.26H), 6.96 (dd, 1.12H, *J* = 6.2, 2.6 Hz), 6.90–6.81 (m, 2H), 5.95 (s, 0.25H), 5.94 (s, 2H), 3.73 (q, 2H, *J* = 7.0 Hz), 3.67 (q, 0.24H, *J* = 6.9 Hz), 1.21 (t, 3.37H, *J* = 7.0 Hz), 0.42 (s, 6H), 0.36 (s, 0.73H); ^{13}C NMR (101 MHz, $CDCl_3$): δ 152.09, 148.93, 147.55, 146.32, 127.79, 126.57, 126.13, 121.50, 117.51, 112.93, 110.04, 108.70, 100.67, 100.33, 58.96, 58.73, 18.55, 18.51, -1.21, -1.49; HRMS (EI+): Calcd for $[C_{11}H_{16}O_3Si]^+$ $[M]^+$: 224.0863, found: 224.0860.

Ethoxy(3-methoxyphenyl)dimethylsilane and regioisomers (P9)

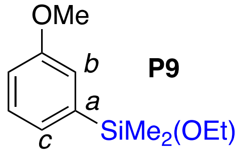

P9

Modified procedure: 5 mol % $[RhCl(Ph\text{-}BPE)]_2$ (129 mg, 0.1 mmol) and Anisole (865 mg, 8 mmol). Complex **P9** was obtained in a mixture (*a:b:c*=4.8:1.3:1) [50% conv., 49% isolated yield (204 mg)]. ^{1}H NMR (400 MHz, $CDCl_3$): δ 7.57 (d, 0.26H, *J* = 8.6 Hz), 7.51 (d, 0.65H, *J* = 8.7 Hz), 7.32 (dd, 1.60H, *J* = 8.2, 7.3 Hz,), 7.16 (dt, 1H, *J* = 7.2, 1.0 Hz), 7.12–7.11 (m, 1H), 6.98–6.90 (m, 2.03H), 3.83 (s, 3H), 3.82 (s, 1.11H), 3.81 (s, 0.62H), 3.75 (q, 0.41H, *J* = 7.2 Hz), 3.66 (dq, 2.20H, *J* = 10.2, 7.0 Hz), 1.23 (dt, 1.89H, *J* = 6.9, 3.4 Hz), 1.19 (t, 3H, *J* = 7.0 Hz), 0.37 (s, 6H), 0.36 (s, 1.41H), 0.26 (s, 1.26H); ^{13}C NMR (101 MHz, $CDCl_3$): δ 164.23, 160.91, 159.14, 139.77, 135.72, 135.44, 135.13, 131.39, 129.19, 129.06, 126.37, 125.81, 125.65, 120.57, 119.26, 119.08, 118.95, 115.64, 114.94, 113.92, 113.66, 109.70, 58.81, 58.66, 58.53, 55.19, 55.15, 55.10, 18.63, 18.55, 18.46, -1.05, -1.51, -1.60; HRMS (CI): Calcd for $[C_{10}H_{12}F_4OSi]^+$ $[M+H]^+$: 211.1149, found: 211.1149.

P10

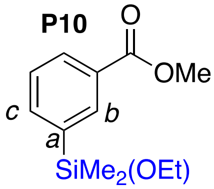

Methyl 3-(ethoxydimethylsilyl)benzoate and regioisomers (P10)

Modified procedure: 1 mol % $[RhCl(Ph\text{-}BPE)]_2$ (12.9 mg, 0.01 mmol), methyl benzoate (408 mg, 3 mmol), $HSiMe_2(OEt)$ (208 mg, 2 mmol), and cyclohexene (198 mg, 2.4 mmol). Complex **P10** was obtained in a mixture (*a:b:c*=3.7:2.9:1) [85% conv., 40% isolated yield (190 mg)]. ^{1}H NMR (400 MHz, $CDCl_3$): δ 8.25 (s, 0.62H), 8.06 (dt, 0.74H, *J* = 7.8, 1.6 Hz), 8.03 (d, 0.66H, *J* = 8.3 Hz), 7.77 (dt, 0.68H, *J* = 7.3, 1.3 Hz), 7.66 (d, 0.65H, *J* = 8.3 Hz), 7.60–7.52 (m, 0.68H), 7.46 (td, 1H, *J* = 7.5, 0.6 Hz), 7.39–7.30 (m, 2.44H),

7.28–7.26 (m, 0.21H), 7.26–7.22 (m, 0.25H), 4.88–4.68 (m, 1.40H), 3.92 (d, 3H, J = 2.1 Hz), 3.82 (q, 0.38H, J = 7.0 Hz), 3.75 (q, 1.13H, J = 7.0 Hz), 3.68 (q, 2H, J = 7.0 Hz), 3.41–3.33 (m, 0.30H), 1.25–1.14 (m, 5.98H), 0.46–0.34 (m, 7.23H), 0.17 (s, 3.41H); ^{13}C NMR (101 MHz, $CDCl_3$): δ 167.46, 167.33, 144.32, 140.75, 138.79, 138.08, 134.63, 133.69, 133.56, 133.03, 132.52, 131.78, 131.08, 130.79, 129.70, 129.65, 128.75, 128.48, 128.43, 128.32, 128.01, 127.29, 126.68, 126.30, 126.10, 64.49, 58.95, 58.91, 58.30, 52.24, 52.22, 18.54, 18.47, 18.36, -1.59, -1.64, -2.95; HRMS (EI+) calcd for $[C_{12}H_{17}O_3Si]^+$ $[M–H]^+$: 237.0942, found: 237.0940.

Ethoxydimethyl-[3-(trifluoromethyl)phenyl]silane and regioisomer (P11)

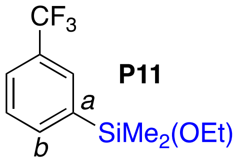

P11

Modified procedure: 2 mol % $[RhCl(Ph\text{-}BPE)]_2$ (51.6 mg, 0.04 mmol), trifluorotoluene (1 ml, 8 mmol), cyclohexene (198 mg, 2.4 mmol), and $HSiMe_2(OEt)$ (**1**, 208 mg, 2 mmol). Complex **P11** was obtained in a mixture (*a:b*=1.8:1) [69% conv., 66% isolated yield (327 mg)]. ^{1}H NMR (400 MHz, $CDCl_3$): ^{1}H NMR (400 MHz, $CDCl_3$) δ 7.81–7.79 (m, 1H), 7.75 (d, 1.13H, J = 7.4 Hz), 7.72–7.67 (m, 1H), 7.63 (t, 2H, J = 8.0 Hz), 7.52–7.47 (m, 1.20H), 3.69 (qd, 3.14H, J = 7.0, 1.2 Hz,), 1.20 (td, 4.69H, J = 7.0, 1.1 Hz), 0.41 (s, 6H), 0.40 (s, 3.28H); ^{13}C NMR (101 MHz, $CDCl_3$): δ 143.20, 139.74, 136.87 (q, J = 0.3 Hz), 133.85, 131.57 (q, J = 32.3 Hz), 130.19 (q, J = 32.0 Hz), 129.94 (q, J = 3.7 Hz), 128.23, 126.29 (q, J = 3.7 Hz), 124.56 (q, J = 272.5 Hz), 124.46 (q, J = 3.8 Hz), 123.02 (q, J = 276.8 Hz), 58.92, 58.79, 18.46, 18.35, -1.30, -1.73; ^{19}F NMR (376 MHz, $CDCl_3$): δ -62.65, -62.98; HRMS (EI+) calcd for $[C_{10}H_{12}F_3OSi]^+$ $[M–Me]^+$: 233.0604, found 233.0601.

[3,5-Bis(trifluoromethyl)phenyl]ethoxydimethylsilane (P12)

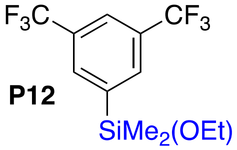

P12

Modified procedure: 2 mol % $[RhCl(Ph\text{-}BPE)]_2$ (56 mg, 0.044 mmol), 1,3-bis(trifluoromethyl)benzene (1.9 g, 8.8 mmol, 4 equiv.), cyclohexene (217 mg, 2.64 mmol), and $HSiMe_2(OEt)$ (**1**, 229 mg, 2.2 mmol) [36% conv., 30% isolated yield (189 mg)]. ^{1}H NMR (400 MHz, $CDCl_3$): δ 7.99 (s, 2H), 7.89 (s, 1H), 3.72 (q, 4H, J = 7.0 Hz), 1.22 (t, 3H, J = 7.0 Hz), 0.44 (s, 6H); ^{13}C NMR (101 MHz, $CDCl_3$): δ 141.97, 133.32 (q, J = 1.2 Hz), 130.98 (q, J = 32.8 Hz), 123.73 (q, J = 272.8 Hz), 123.40 (p, J = 3.8 Hz), 59.18, 18.49, -1.61; ^{19}F NMR (376 MHz, $CDCl_3$): δ -62.87; HRMS (EI+): Calcd for $[C_{11}H_{11}F_6OSi]^+$ $[M–Me]^+$: 301.0478, found: 301.0474.

Ethoxy (3-isopropylphenyl)dimethylsilane and regioisomer (P13)

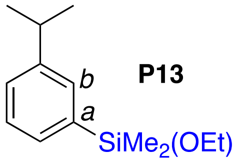

P13

Modified procedure: 5 mol % $[RhCl(Ph\text{-}BPE)]_2$ (129 mg, 0.1 mmol), cumene (962 mg, 8 mmol, 4 equiv.), cyclohexene (197 mg, 2.4 mmol), and $HSiMe_2(OEt)$ (**1**, 208 mg, 2 mmol). Complex **P13** was obtained in a mixture (*a:b*=1.9:1) [33% conv., 32% yield (142 mg)]. ^{1}H NMR (400 MHz, $CDCl_3$) δ 7.51 (d, 0.58H, *J* = 7.9 Hz), 7.43 (d, 0.71H, *J* = 0.6 Hz), 7.39 (dt, 0.84H, *J* = 7.1, 1.4 Hz), 7.31 (t, 1.04H, *J* = 7.4 Hz), 7.28 (t, 0.81H, *J* = 1.8 Hz), 7.25–7.14 (m, 1.57H), 3.75–3.59 (m, 2H), 3.04 –2.83 (m, 0.81H), 1.26 (dd, 6.16H, *J* = 6.9, 2.3 Hz), 1.22–1.15 (m, 3.31H), 0.38 (s, 3.56H), 0.37 (s, 1.92H); ^{13}C NMR (101 MHz, $CDCl_3$): δ 150.40, 148.13, 137.98, 134.24, 133.71, 132.26, 131.74, 131.63, 131.08, 128.15, 127.91, 127.70, 127.38, 126.12, 58.77, 58.63, 34.31, 34.28, 24.16, 23.97, 18.56, 18.47, -1.53, -3.97; HRMS (CI): Calcd for $[C_{13}H_{22}OSi]^+$ $[M+H]^+$: 223.1513, found: 223.1512.

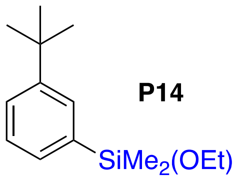

P14

(3-(*tert*-butyl)phenyl)ethoxydimethylsilane (P14)

Modified procedure: 5 mol % $[RhCl(Ph\text{-}BPE)]_2$ (129 mg, 0.1 mmol), *tert*-butylbenzene (1074 mg, 8 mmol, 4 equiv.), cyclohexene (197 mg, 2.4 mmol), and $HSiMe_2(OEt)$ (**1**, 208 mg, 2 mmol). **P14** was unable to be isolated and characterized (13% conv.). HRMS (CI): Calcd for $[C_{13}H_{21}OSi]^+$ $[M–Me]^+$: 221.1356, found: 221.1356.

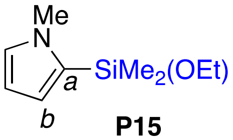

P15

2-(Ethoxydimethylsilyl)-*N*-methyl-pyrrole and regioisomer (P15)

Modified procedure: 0.5 mol % $[RhCl(Ph\text{-}BPE)]_2$ (6.4 mg, 0.005 mmol), and 1-Methylpyrrole (1 ml, 11.3 mmol) [>98% conv., 85% isolated yield (156 mg)]. Complex **P15** was obtained in a mixture (*a:b*=7.1:1). ^{1}H NMR (400 MHz, $CDCl_3$): δ 6.81 (dd, 1H, *J* = 2.4, 1.6 Hz), 6.73 (t, 0.11H, *J* = 1.8 Hz), 6.70 (t, 0.11H, *J* = 2.2 Hz), 6.44 (dd, 1H, *J* = 3.5, 1.5 Hz), 6.27 (dd, 0.11H, *J* = 2.5, 1.6 Hz), 6.16 (dd, 1H, *J* = 3.5, 2.4 Hz), 3.78 (s, 3H), 3.68 (s, 0.43H), 3.64 (q, 2.24H, *J* = 7.0 Hz), 1.16 (t, 3.58H, *J* = 7.0 Hz), 0.39 (s, 6.12H), 0.31 (s, 0.86H); ^{13}C NMR (100 MHz, $CDCl_3$): δ 130.7, 127.4, 120.6, 108.0, 58.5, 36.8, 18.4, -1.0; HRMS (EI+) calcd for $[C_9H_{17}NOSi]^+$ $[M]^+$: 183.1074, found: 183.1072.

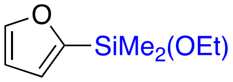

P16

Ethoxy(furan-2-yl)dimethylsilane (P16)

Modified procedure: 0.5 mol % $[RhCl(Ph\text{-}BPE)]_2$ (12.9 mg, 0.01 mmol), furan (2 ml, 27.5 mmol), cyclohexene (198 mg, 2.4 mmol), and $HSiMe_2(OEt)$ (**1**, 208 mg, 2 mmol) [>98% conv., 99% isolated yield (338 mg)]. ^{1}H NMR (400 MHz, $CDCl_3$): δ 7.67

(dd, 1H, *J* = 1.6, 0.6 Hz), 6.92–6.61 (m, 1H), 6.40 (dd, 1H, *J* = 3.2, 1.6 Hz), 3.70 (q, 2H, *J* = 7.0 Hz), 1.17 (t, 3H, *J* = 7.0 Hz), 0.38 (s, 6H); ^{13}C NMR (101 MHz, $CDCl_3$): δ 158.21, 146.99, 120.95, 109.44, 59.08, 18.41, -1.92; HRMS (EI+) calcd for $[C_8H_{14}O_2Si]^+$ $[M]^+$: 170.0758, found: 170.0755. Spectroscopic data were consistent with those previously reported.[2]

Ethoxydimethyl(thiophen-2-yl)silane (P17)

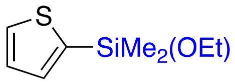

P17

Modified procedure: 1 mol % $[RhCl(Ph\text{-}BPE)]_2$ (25.8 mg, 0.02 mmol), thiophene (505 mg, 6 mmol), cyclohexene (198 mg, 2.4 mmol), and $HSiMe_2(OEt)$ (**1**, 208 mg, 2 mmol) [67% conv., 59% isolated yield (220 mg)]. ^{1}H NMR (400 MHz, $CDCl_3$): δ 7.64 (dd, 1H, *J* = 4.6, 0.9 Hz), 7.37 (dd, 1H, *J* = 3.3, 0.9 Hz), 7.22 (dd, 1H, *J* = 4.6, 3.3 Hz), 3.70 (q, 2H, *J* = 7.0 Hz), 1.19 (t, 3H, *J* = 7.0 Hz), 0.44 (s, 6H); ^{13}C NMR (100 MHz, $CDCl_3$): δ 137.5; 135.1; 131.2; 58.9; 18.5; -0.5; HRMS (EI+) calcd for $[C_8H_{14}OSSi]^+$ $[M]^+$: 186.0529, found: 186.0526.

***tert*-Butyl 3-(ethoxydimethylsilyl)-1*H*-pyrrole-1-carboxylate (P18)**

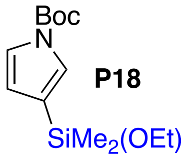

P18

Modified procedure: 2 mol % $[RhCl(Ph\text{-}BPE)]_2$ (49.5 mg, 0.038 mmol), *N*-Boc-pyrrole (1 ml, 10.2 mmol), cyclohexene (188 mg, 2.29 mmol), and $HSiMe_2(OEt)$ (**1**, 199 mg, 1.91 mmol) [26% conv., 26% isolated yield (132 mg)]. ^{1}H NMR (400 MHz, $CDCl_3$): δ 7.34 (t, 1H, *J* = 1.7 Hz), 7.30 (dd, 1H, *J* = 3.0, 1.9 Hz), 6.30 (dd, 1H, *J* = 3.0, 1.6 Hz), 3.67 (q, 2H, *J* = 7.0 Hz), 1.60 (s, 9H), 1.18 (t, 3H, *J* = 7.0 Hz), 0.33 (s, 6H); ^{13}C NMR (101 MHz, $CDCl_3$): δ 148.88, 126.37, 121.20, 120.23, 116.10, 83.83, 58.64, 28.09, 18.54, -1.13; HRMS (CI): Calcd for $[C_{12}H_{20}NO_3Si]^+$ $[M\text{–}Me]^+$: 254.1207, found: 254.1207.

2-(Ethoxydimethylsilyl)-1-methyl-1*H*-indole (P19)

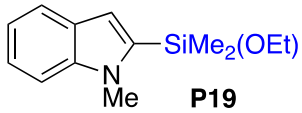

P19

Modified procedure: 0.5 mol % $[RhCl(Ph\text{-}BPE)]_2$ (6.4 mg, 0.01 mmol) and 1-methylindole (1 ml) in THF at 50 °C (>98% conv., 92% isolated yield). ^{1}H NMR (400 MHz, $CDCl_3$): δ 7.64 (d, 1H, *J* = 7.9 Hz), 7.36 (d, 1H, *J* = 8.3 Hz), 7.26 (t, 1H, *J* = 7.3 Hz), 7.11 (t, 1H, *J* = 7.4 Hz), 6.77 (s, 1H), 3.93 (s, 3H), 3.69 (q, 2H, *J* = 7.0 Hz), 1.20 (t, 3H, *J* = 7.0 Hz), 0.50 (d, 6H, *J* = 1.1 Hz); ^{13}C NMR (101 MHz, $CDCl_3$): δ 140.33, 138.86, 128.30, 122.57, 121.09, 119.34, 112.77, 109.36, 58.92, 32.87, 18.42, -0.93; HRMS (EI+) calcd for $[C_{13}H_{19}NOSi]^+$ $[M]^+$: 233.1230, found: 233.1232.

Benzofuran-2-yl(ethoxy)dimethylsilane (P20)

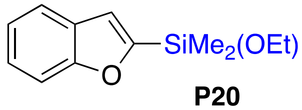

P20

Modified procedure: 0.5 mol % $[RhCl(Ph\text{-}BPE)]_2$ (6.4 mg, 0.005 mmol), and benzofuran (236 mg, 2 mmol**)** [>98% conv., 97% isolated yield (214 mg)]. ^{1}H NMR (400 MHz, $CDCl_3$): δ 7.61 (dd, 1H, *J* = 7.7, 0.6 Hz), 7.53 (dd, 1H, *J* = 8.2, 1.0 Hz), 7.30 (td, 1H, *J* = 7.7, 1.3 Hz), 7.22 (td, 1H, *J* = 7.5, 1.0 Hz), 3.77 (q, 2H, *J* = 7.0 Hz), 1.21 (t, 3H, *J* = 7.0 Hz), 0.47 (s, 6H); ^{13}C NMR (101 MHz, $CDCl_3$): δ 160.98, 158.13, 127.79, 124.91, 122.58, 121.43, 117.38, 111.64, 59.31, 18.43, -1.90; HRMS (EI+) calcd for $[C_{12}H_{16}O_2Si]^+$ $[M]^+$: 220.0914, found: 220.0912.

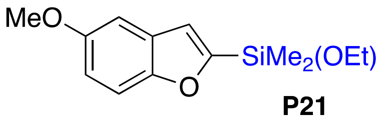

P21

Ethoxy(5-methoxybenzofuran-2-yl)dimethylsilane (P21)

Modified procedure: 0.5 mol % $[RhCl(Ph\text{-}BPE)]_2$ (6.4 mg, 0.005 mmol), and 5-methoxybenzofuran (271 mg, 1.1 mmol**)** [>98% conv., 97% isolated yield (242 mg)]. ^{1}H NMR (400 MHz, $CDCl_3$): δ 7.41 (d, 1H, *J* = 8.9 Hz), 7.04 (d, 1H, *J* = 2.6 Hz), 7.00 (d, 1H, *J* = 1.0 Hz), 6.92 (dd, 1H, *J* = 8.9, 2.6 Hz), 3.84 (s, 3H), 3.76 (q, 2H, *J* = 7.0 Hz), 1.20 (t, 3H, *J* = 7.0 Hz), 0.45 (s, 6H); ^{13}C NMR (101 MHz, $CDCl_3$): δ 161.97, 155.90, 153.28, 128.28, 117.41, 114.04, 111.97, 103.33, 59.26, 55.97, 18.40, -1.95; HRMS (EI+) calcd for $[C_{13}H_{18}O_3Si]^+$ $[M]^+$: 250.1020, found: 250.1018.

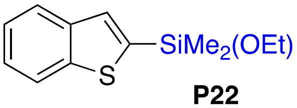

P22

Benzo[*b*]thiophen-2-yl(ethoxy)dimethylsilane (P22)

Modified procedure: 1 mol % $[RhCl(Ph\text{-}BPE)]_2$ (12.9 mg, 0.01 mmol), and thianapthene (148 mg, 1.1 mmol**)** [>98% conv., 99% isolated yield (235 mg)]. ^{1}H NMR (400 MHz, $CDCl_3$): δ 7.95–7.92 (m, 1H), 7.90–7.86 (m, 1H), 7.60 (d, 1H, *J* = 0.5 Hz), 7.42–7.34 (m, 2H), 3.79 (q, 2H, *J* = 7.0 Hz), 1.25 (t, 3H, *J* = 7.0 Hz), 0.54 (s, 6H); ^{13}C NMR (101 MHz, $CDCl_3$) δ 143.76, 140.96, 139.54, 131.98, 124.66, 124.20, 123.86, 122.40, 59.14, 18.49, -0.69; HRMS (EI+) calcd for $[C_{12}H_{16}OSSi]^+$ $[M]^+$: 236.0686, found: 236.0684.

2.5 Representative procedure for Rh-catalyzed Double C−H silylation of Heteroarenes (Scheme 4):

In a N_2-filled glovebox, an oven-dried pressure tube was charged with $[RhCl(Ph\text{-}BPE)]_2$ (**2**, 6.7 mg, 0.5 mol %), and furan (68 mg, 1 equiv.). After stirring for 1 h at 100 °C in an oil bath, the reaction mixture was cooled to 22 °C. To the mixture, cyclohexene (197 mg, 2.4 equiv.) and $HSiMe_2(OEt)$ (**1**, 261 mg, 2.5 mmol) was added. The reaction mixture was allowed to stir for 20 h at 100 °C. Yields were obtained with the following workup procedure in an N_2-filled glovebox: the crude mixture was evaporated, the residue diluted with hexane, and filtered through a syringe

filter by rinsing with hexane. The filtrate was then purified by reduced pressure distillation under vacuum to obtain the desired product **P23** [98% conv., 92% isolated yield (251 mg)].

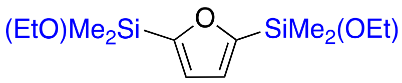

P23

2,5-Bis(ethoxydimethylsilyl)furan (P23)

^{1}H NMR (400 MHz, CDCl$_3$): δ 6.72 (s, 2H), 3.70 (q, 4H, J = 7.0 Hz), 1.16 (t, 6H, J = 7.0 Hz), 0.38 (s, 12H); ^{13}C NMR (101 MHz, CDCl$_3$) δ 162.85, 120.46, 59.07, 18.36, -1.86; HRMS (CI): Calcd for $[C_{12}H_{24}O_3Si_2]^+$ $[M]^+$: 272.1258, found: 272.1260.

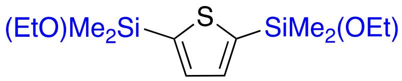

P24

2,5-Bis(ethoxydimethylsilyl)thiofuran (P24)

[>98% conv., 98% isolated yield (283 mg)]. ^{1}H NMR (400 MHz, CDCl$_3$): δ 7.42 (s, 2H), 3.70 (q, 4H, J = 7.0 Hz,), 1.18 (t, 6H, J = 7.0 Hz), 0.44 (s, 12H); ^{13}C NMR (101 MHz, CDCl$_3$) δ 143.85, 135.92, 58.91, 18.45, -0.45; HRMS (CI): Calcd for $[C_{12}H_{24}O_2SSi_2]^+$ $[M]^+$: 288.1030, found: 288.1031.

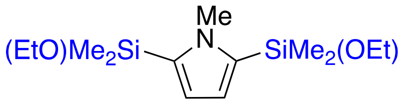

P25

2,5-Bis(ethoxydimethylsilyl)-1-methyl-1*H*-pyrrole (P25)

P25 was unable to be isolated and characterized [<5% conv.].

3. Synthesis and Characterization of Piers-Rubinsztajn Products

Some of the aryl-dimethylethoxysilanes products showed varying degrees of decomposition due to air and/or moisture sensitivity. In order to confirm the identity of the desired products, C–H silylation products were converted into stable siloxanes by the Piers–Rubinsztajn reaction.[3] *Note that the Piers–Rubinsztajn reaction conditions were not optimized.*

3.1 Representative procedure for Piers–Rubinsztajn Reaction

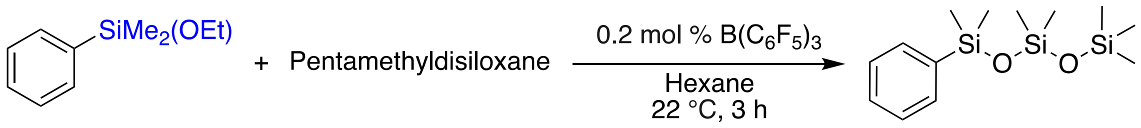

P1 **P1b**

In a N_2-filled glovebox, to an oven-dried vial, pentamethyldisiloxane (1.1 g, 4.9 mmol) was added to a solution of phenyldimethylethoxysilane (**P1**, 270 mg, 1.5 mmol) in dry hexane (3 ml). The

mixture was allowed to stir at 22 °C for 10 min before the addition of 0.2 mol% tris(pentafluorophenyl)borane (1.54 mg, 0.003 mmol). After 5 min, rapid evolution of gas and heat from the solution occurred. The mixture was allowed to stir for 3 h and cool to 22 °C before the addition of neutral alumina (0.5 g). The resulting mixture was filtered through a syringe filter by rinsing with hexane, concentrated, and purified by silica gel chromatography affording the desired product **P1b** as a colorless liquid [53% isolated yield (236 mg)]. ^{1}H NMR (400 MHz, $CDCl_3$): δ 7.58–7.55 (m, 2H), 7.36 (dd, 3H, *J* = 5.0, 1.9 Hz), 0.34 (s, 6H), 0.07 (s, 9H), 0.04 (s, 6H); ^{13}C NMR (101 MHz, $CDCl_3$): δ 140.08, 133.17, 129.33, 127.80, 1.94, 1.43, 0.92; HRMS (EI+) calcd for $[C_{12}H_{23}O_2Si_3]^+$ $[M–Me]^+$: 283.1000, found: 283.0998.

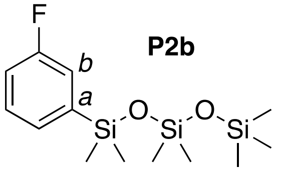

1-(3-Fluorophenyl)-1,1,3,3,5,5,5-heptamethyltrisiloxane and regioisomer (P2b)

Modified procedure: 1 mol % $B(C_6F_5)_3$ (4.7 mg, 0.0092 mmol), ethoxy(2-fluorophenyl)dimethylsilane and regioisomer (**P2**, 183 mg, 0.93 mmol), pentamethyldisiloxane (206 mg, 1.29 mmol), and dry hexane (5 ml). Complex **P2b** was obtained in a mixture (*a:b*=2.2:1) [43% isolated yield (125 mg)]. ^{1}H NMR (400 MHz, $CDCl_3$): δ 7.52 (td, 1H, *J* = 7.3, 1.9 Hz), 7.40–7.29 (m, 1.67H), 7.25–7.19 (m, 0.29H), 7.14 (t, 1H), 7.05 (tdd, 0.5H, *J* = 9.3, 4.1, 2.0 Hz), 6.98 (t, 0.95H, *J* = 8.4 Hz), 0.40 (s, 6H), 0.35 (s, 2.67H), 0.15–0.00 (m, 21.32H); ^{13}C NMR (101 MHz, $CDCl_3$) δ 167.04 (d, *J* = 241.1 Hz), 162.77 (d, *J* = 248.0 Hz), 143.22 (d, *J* = 3.9 Hz), 135.36 (d, *J* = 11.1 Hz), 131.71 (d, *J* = 8.2 Hz), 129.64 (d, *J* = 7.0 Hz), 128.64 (d, *J* = 3.0 Hz), 125.93 (d, *J* = 29.8 Hz), 123.86 (d, *J* = 2.7 Hz), 119.55 (d, *J* = 18.4 Hz), 116.23 (d, *J* = 21.1 Hz), 114.78 (d, *J* = 25.4 Hz), 1.96, 1.92, 1.48 (d, *J* = 1.5 Hz), 1.41, 1.35, 0.84; HRMS (EI+) calcd for $[C_{12}H_{22}FO_2Si_3]^+$ $[M–Me]^+$: 301.0906, found: 301.0903.

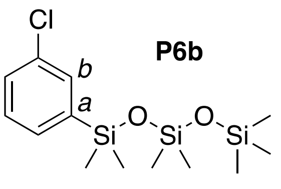

1-(3-Chlorophenyl)-1,1,3,3,5,5,5-heptamethyltrisiloxane and regioisomer (P6b)

Modified procedure: 1 mol % $B(C_6F_5)_3$ (5.1 mg, 0.01 mmol), (3-chlorophenyl)ethoxydimethylsilane and regioisomer (**P6**, 161 mg, 0.75 mmol), pentamethyldisiloxane (168 mg, 1.13 mmol), and dry hexane (5 ml). Complex **P3b** was obtained in a mixture that was not able to be separated [13% isolated yield (32 mg)]. ^{1}H NMR (400 MHz, $CDCl_3$): δ 7.62–7.26 (m, 4H), 0.46–0.29 (m, 6H), 0.12–0.02 (m, 15H); ^{13}C NMR (101

MHz, $CDCl_3$): δ 142.77, 134.90, 134.57, 134.28, 133.34, 133.16, 133.00, 131.40, 131.07, 129.67, 129.39, 129.34, 129.25, 128.05, 127.96, 127.80, 31.75, 22.82, 14.28, 1.92, 1.41, 0.83; HRMS (EI+) calcd for $[C_{12}H_{22}ClO_2Si_3]^+$ $[M–Me]^+$: 317.0611, found: 3117.0606.

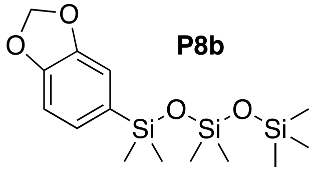
P8b

1-(Benzo[*d*][1,3]dioxol-5-yl)-1,1,3,3,5,5,5-heptamethyltrisiloxane (P8b)

Modified procedure: 1 mol % $B(C_6F_5)_3$ (4.6 mg, 0.009 mmol), benzo[*d*][1,3]dioxol-5-yl(ethoxy)dimethylsilane and regioisomer (**P8**, 201 mg, 0.9 mmol), pentamethyldisiloxane (479 mg, 3.2 mmol), and dry hexane (5 ml) [24% isolated yield (75 mg)]. ^{1}H NMR (400 MHz, $CDCl_3$): δ 6.98 (dd, *J* = 5.7, 3.2 Hz, 1H), 6.88–6.79 (m, 2H), 5.92 (s, 2H), 0.38 (s, 6H), 0.08 (s, 9H), 0.06 (s, 6H); ^{13}C NMR (101 MHz, $CDCl_3$): δ 151.64, 146.17, 126.44, 121.33, 119.34, 109.75, 100.24, 1.92, 1.36, 1.19; HRMS (EI+) calcd for $[C_{14}H_{26}O_4Si_3]^+$ $[M]^+$: 342.1133, found: 342.1133.

P16b
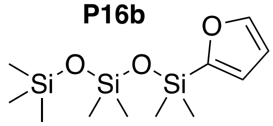

1-(Furan-2-yl)-1,1,,3,3,5,5,5-heptamethyltrisiloxane (P16b)

Modified procedure: 1 mol % $B(C_6F_5)_3$ (7 mg, 0.014 mmol), ethoxy(furan-2-yl)dimethylsilane (**P16**, 232 mg, 1.4 mmol), pentamethyldisiloxane (485 mg, 3.3 mmol), and dry hexane (5 ml) [41% isolated yield (160 mg)]. ^{1}H NMR (400 MHz, $CDCl_3$): δ 7.64 (dd, 1H, *J* = 1.7, 0.6 Hz), 6.70 (dd, 1H, *J* = 3.3, 0.6 Hz), 6.39 (dd, 1H, *J* = 3.2, 1.6 Hz), 0.36 (s, 6H), 0.08 (s, 9H), 0.02 (s, 6H); ^{13}C NMR (101 MHz, $CDCl_3$): δ 159.60, 146.48, 119.98, 109.36, 1.87, 1.17, 0.35; HRMS (EI+) calcd for $[C_{10}H_{21}O_3Si_3]^+$ $[M–Me]^+$: 273.0793, found: 273.0795.

P17b
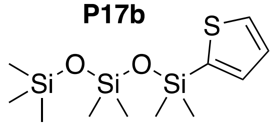

1,1,1,3,3,5,5-Heptamethyl-5-(thiophen-2-yl)trisiloxane (P17b)

Modified procedure: 1 mol % $B(C_6F_5)_3$ (2.3 mg, 0.0046 mmol), 2-(dimethylethoxysilyl)thiofuran (**P17**, 85 mg, 0.46 mmol), pentamethyldisiloxane (81 mg, 0.55 mmol), and dry hexane (2 ml) [74% isolated yield (103 mg)]. ^{1}H NMR (400 MHz, $CDCl_3$): δ 7.60 (dd, 1H, *J* = 4.6, 0.9 Hz), 7.40–7.34 (m, 0.25H), 7.32 (dd, 0.85H, *J* = 3.3, 0.9 Hz), 7.28 (dd, 0.40H, *J* = 3.3, 0.9 Hz), 7.18 (td, 1.26H, *J* = 4.4, 3.3 Hz), 0.40 (s, 6H), 0.38 (s, 2.48H), 0.09 (s, 2.81H), 0.07 (s, 8.93H), 0.05 (s, 2.12H), 0.03 (s, 6H); ^{13}C NMR (101 MHz, $CDCl_3$): δ 139.63, 139.35, 134.43, 134.33, 130.80, 130.65, 128.13, 128.05, 1.95, 1.91, 1.89, 1.86, 1.35, 1.31; HRMS (EI+) calcd for $[C_{10}H_{21}O_2SSi_3]^+$ $[M–Me]^+$: 289.0565, found: 289.0562.

1-[Benzofuran-2-yl]-1,1,3,3,5,5,5-heptamethyltrisiloxane (P20b)

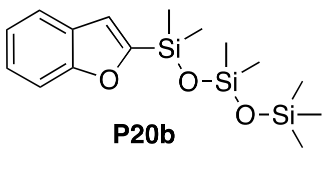
P20b

Modified procedure: 1 mol % $B(C_6F_5)_3$ (5.1 mg, 0.01 mmol), benzofuran-2-yl(ethoxy)dimethylsilane (**P20**, 203 mg, 0.92 mmol), pentamethyldisiloxane (356 mg, 2.4 mmol), and dry hexane (5 ml) [47% isolated yield (146 mg)]. ^{1}H NMR (400 MHz, $CDCl_3$): δ 7.60 (dd, 1H, J = 7.7, 0.6 Hz), 7.52 (dd, 1H, J = 8.2, 0.9 Hz), 7.30 (td, 1H, J = 7.8, 1.4 Hz), 7.26–7.17 (m, 1H), 7.04 (d, 2H, J = 1.0 Hz), 0.45 (s, 6H), 0.08 (s, 9H), 0.07 (s, 6H); ^{13}C NMR (101 MHz, $CDCl_3$): δ 162.39, 158.00, 127.93, 124.69, 122.47, 121.38, 116.42, 111.56, 1.89, 1.26, 0.41; HRMS (EI+) calcd for $[C_{15}H_{26}O_3Si_3]^+$ $[M]^+$: 338.1184, found: 338.1185.

1-((2-(Dimethylsilyl)benzofuan-5-yl)oxy-1,1,3,3,3,-pentamethyldisiloxane (P21b) and 1,1,1,3,3,5,5-Heptamethyl-5-(5-((3-(1,1,3,3,3-pentamethyldisiloaneyl)oxy)-benzofuran-2-yl)trisiloxane (P21c)

Modified procedure: 1 mol % $B(C_6F_5)_3$ (4.5 mg, 0.0087 mmol), ethoxy(5-methoxybenzofuran-2-yl)dimethylsilane (**P21**, 218 mg, 0.87 mmol), pentamethyldisiloxane (465 mg, 3.1 mmol), and dry hexane (5 ml). A mixture of two products, **P21b** and **P21c**, were obtained. The major product, **P21b** [56% isolated yield (166 mg)], and the minor product, **P21c** [15% isolated yield (66 mg)], shown below were identified by ^{1}H and ^{13}C NMR.

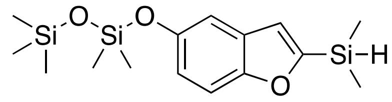

P21b

^{1}H NMR (400 MHz, $CDCl_3$): δ 7.35 (d, J = 8.9 Hz, 1H), 7.06 (d, 1H, J = 2.3 Hz), 6.95 (d, 1H, J = 0.9 Hz), 6.87 (dd, 1H, J = 8.8, 2.5 Hz), 4.50 (hept, 1H, J = 3.8 Hz), 0.42 (d, 6H, J = 3.8 Hz), 0.22 (s, 6H), 0.12 (s, 9H); ^{13}C NMR (101 MHz, $CDCl_3$): δ 161.42, 154.03, 150.28, 128.62, 117.96, 117.58, 111.43, 110.73, 1.91, -0.32, -4.60; HRMS (EI+) calcd for $[C_{15}H_{26}O_3Si_3]^+$ $[M]^+$: 338.1184, found: 338.1182.

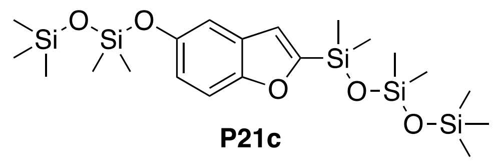

P21c

^{1}H NMR (400 MHz, $CDCl_3$): δ 7.35 (d, 1H, J = 8.7 Hz), 7.08 (d, 3H, J = 2.5 Hz), 6.94 (d, 2H, J = 1.0 Hz), 6.87 (dd, 3H, J = 8.8, 2.4 Hz), 0.43 (s, 17H), 0.23 (s, 16H), 0.12 (s, 25H), 0.08 (s, 22H), 0.07 (s, 14H); ^{13}C NMR (101 MHz, $CDCl_3$): δ 163.29, 153.65, 150.21, 128.55, 117.96, 116.43, 111.54, 110.89, 1.90, 1.88, 1.26, 0.40, -0.30; HRMS (EI+) calcd for $[C_{20}H_{40}O_5Si_5]^+$ $[M]^+$: 500.1717, found: 500.1713.

1-(Benzo[*b*]thiophen-2-yl)-1,1,3,3,5,5,5-heptamethyltrisiloxane (P22b)

P22b

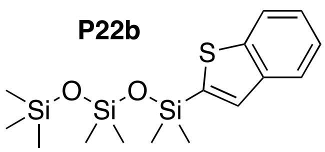

Modified procedure: 1.8 mol % $B(C_6F_5)_3$ (8.4 mg, 0.017 mmol), 2 benzo[*b*]thiophen-2-yl(ethoxy)dimethylsilane (**P22**, 216 mg, 0.91 mmol), pentamethyldisiloxane (664 mg, 4.5 mmol), and dry hexane (3 ml) [15% isolated yield (66 mg)]. ^{1}H NMR (400 MHz, $CDCl_3$): δ 7.94–7.87 (m, 1H), 7.85–7.79 (m, 1H), 7.52 (d, 1H, *J* = 0.8 Hz), 7.33 (tt, 2H, *J* = 11.6, 1.6 Hz,), 0.45 (s, 6H), 0.08 (s, 9H), 0.07 (s, 6H); ^{13}C NMR (101 MHz, $CDCl_3$): δ 143.65, 141.64, 141.04, 131.18, 124.48, 124.12, 123.81, 122.44, 1.95, 1.75, 1.40; HRMS (EI+) calcd for $[C_{15}H_{26}O_2SSi_3]^+$ $[M]^+$: 354.0956, found: 354.0953.

4. Mechanistic Study:

4.1 Synthesis of $[RhCl(Ph\text{-}BPE)]_2$ Crystals (2)

To a THF solution of $[RhCl(ethylene)_2]_2$ (80 mg, 0.205 mmol), (*S,S*)-Ph-BPE (208 mg, 0.41 mmol) in THF was slowly added. After 3 h of stirring at 22 °C, the reaction mixture was filtered through a syringe filter and a clear orange solution was obtained. Removal of the volatiles and trituration in hexanes followed by drying under vacuum gave orangish-yellow solids. Recrystallization in benzene and hexanes affords $[RhCl(Ph\text{-}BPE)]_2$ (**2**) as red crystals [85% isolated yield (0.218 g)]. Note that when $[RhCl(ethylene)_2]_2$ was used, the crystal form of **2** was obtained relatively easily. ^{1}H NMR (400 MHz, $CDCl_3$): δ 7.98 (d, 8H, *J* = 7.4 Hz), 7.43 (t, 8H, *J* = 6.8 Hz), 7.35–7.31 (m, 4H), 7.16–7.01 (m, 20H), 4.41–3.35 (m, 4H), 2.59–2.52 (m, 4H), 2.46–2.36 (m, 4H), 2.03–1.84 (m, 8H), 1.63–1.53 (m, 4H), 0.50–0.26 (m, 8H); ^{31}P NMR ($CDCl_3$): δ 106.0, 104.8.

4.2 Synthesis of $[Rh_2(Ph\text{-}BPE)_2(\mu\text{-}Cl)(\mu\text{-}H)]$ (5)

$[RhCl(Ph\text{-}BPE)]_2$ (**2**, 0.26 g, 0.2mmol) and excess of $HSiMe_2(OEt)$ (**1**, 2.8 ml) in 10 ml of benzene was heated at 100 °C. The progress of the reaction was indicated by monitoring the decrease of $[(Ph\text{-}BPE)Rh(\mu\text{-}Cl)]_2$ by ^{31}P NMR. After all the $[RhCl(Ph\text{-}BPE)]_2$ disappeared, the volatiles were removed under vacuum to afford orange solids. The solids were washed with hexane twice and then dried under vacuum. Orange crystals were obtained by layering hexanes onto benzene solution [72% isolated yield (0.185 g)]. ^{1}H NMR (C_6D_6, 400 MHz): δ 8.02 (d, 8.0 Hz, 4H, *Ph*),

7.93 (d, 8.0 Hz, 4H, *Ph*), 7.51 (t, 7.6 Hz, 4H, *Ph*), 7.37 (t, 7.4 Hz, 2H, *Ph*), 6.95–7.23 (m, 22H, *Ph*), 6.93 (t, 7.4 Hz, 4H, *Ph*), 4.57 (m, 2H), 4.06 (m, 2H), 2.81 (m, 2H), 2.64 (m, 2H), 2.49 (m, 2H), 2.35 (m, 2H), 1.85–2.14 (m, 8H), 1.71 (br q, 4H), 0.80–0.95 (br m, 4H), 0.01 (m, 4H), -7.91 (br m, 1H, Rh*H*); ^{13}C NMR (C_6D_6, 100 MHz): δ 142.4 (d, 4.5 Hz), 140.9 (d, 3.8 Hz), 139.0 (d, 5.3 Hz), 138.3 (d, 3.8 Hz), 133.8 (s), 130.7 (d, 6.0 Hz), 130.0 (d, 7.6 Hz), 126.4 (d, 13.7 Hz), 125.6 (d, 1.5 Hz), 52.5 (d, 17.4 Hz), 51.5 (d, 24.3 Hz), 50.9 (d, 14.4 Hz), 50.3 (d, 17.5 Hz), 36.8 (d, 3.8 Hz), 36.2 (d, 6.8 Hz), 31.2 (d, 4.5 Hz), 29.6 (br t); ^{31}P NMR (C_6D_6, 161.84 MHz): δ 103.8 (m), 102.6 (m), 99.9 (m), 98.8 (m).

4.3 Synthesis of [(Ph-BPE)Rh(μ-H)]$_2$ (6)

To a benzene solution of [RhCl(Ph-BPE)]$_2$ (**2**, 13 mg, 0.01 mmol), 20 µL of 1 M $LiHBEt_3$ in THF (0.02 mmol) was added. The reaction mixture was heated at 60 °C until all the [RhCl(Ph-BPE)]$_2$ and [Rh_2(Ph-BPE)$_2$(μ-Cl)(μ-H)] disappeared by ^{31}P NMR (five days). After filtration through celite, the volatiles were removed under vacuum. Washing with hexanes twice afforded dark green solids. The crystals were obtained in hexane at -35 °C. ^{1}H NMR (400 MHz, $CDCl_3$): δ 8.34 (d, *J* = 7.6 Hz, 4H, *Ph*), 7.45–7.51 (m, 6H, *Ph*), 7.30 (d, 6H, *Ph*), 7.22(t, 7.4 Hz, 4H, *Ph*), 7.15 (br, 6H, *Ph*), 6.97–7.10 (m, 14H, *Ph*), 4.27 (m, 4H), 2.67 (m, 4H), 2.26 (m, 4H), 1.82–2.21 (m, 8H), 1.69 (m, 4H), 0.85 (m, 4H), -0.39 (m, 4H), -4.23 (br, 2H, Rh-H); ^{13}C NMR (C_6D_6, 100 MHz): δ 141.8 (s), 141.7 (s), 138.7 (s), 130.8 (s), 128.6 (s), 126.7 (s), 125.6 (s), 50.9 (br), 50.4 (s), 36.6 (s), 34.9 (s), 30.9 (s), 29.4 (s), 26.4 (s), 25.6 (s), 18.9 (s), 11.6 (s); ^{31}P NMR ($CDCl_3$): δ 106.6 (m), 105.5 (m).

4.4 Identifying resting state of the catalyst

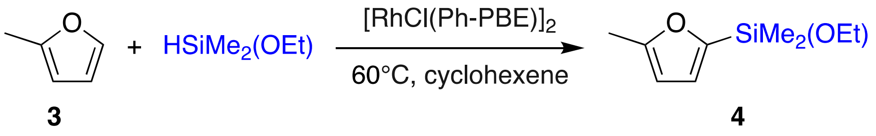

In a N_2-filled glovebox, a stock solution containing 2-methylfuran, $HMe_2Si(OEt)$, cyclohexene, and THF was placed in J-Young tube and cooled in a -35 °C fridge. Then, a stock solution of catalyst **2** in THF was added to the same J-Young tube, quickly taken out of the glove box, and cooled at

-78 °C. The tube was allowed to thaw and was shaken before it was placed in the NMR instrument with the temperature set at 60 °C. Conversion of the catalyst state was monitored by ^{31}P NMR.

^{31}P NMR after 0 h

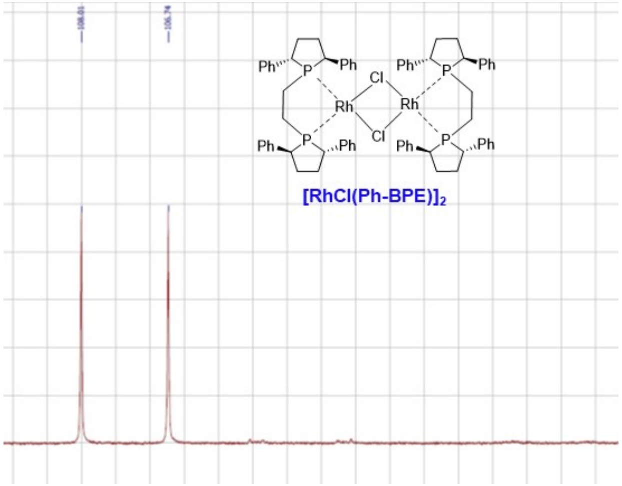

^{31}P NMR after 1 h at 60 °C

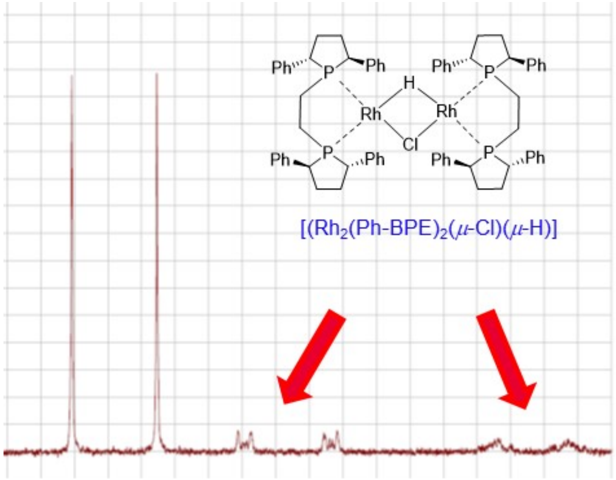

4.5 Determination of Reaction Order in [Furan], [HSi] and [Cat]

In order to determine the reaction order, the reactions between 2-methylfuran and $HSiMe_2(OEt)$ were carried out with various concentration of components. Initial rate laws were used to determine the reaction rate. The reactions were carried out in THF. The progress of the reaction was monitored by GC-MS using decane as an internal standard.

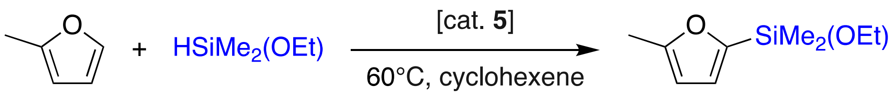

3 **4**

Rate = k_r [2-methylfuran][$HMe_2Si(OEt)$][cat. **5**][cyclohexene]

Table S6. Reaction Rate as a function of [2-methylfuran], [Si–H] and [cat. **5**]

Entry	[2-methylfuran]	[Si–H]	[cat. **5**]	[cyclohexene]	Rate (M/min)
1	0.66 M	0.60 M	3 mM	0.66 M	$2.1X10^{-4}$
2	1.32 M	0.60 M	3 mM	0.66 M	$4.5X10^{-4}$
3	1.98 M	0.60 M	3 mM	0.66 M	$6.4X10^{-4}$
4	0.66 M	1.20 M	3 mM	0.66 M	$2.4X10^{-4}$
5	0.66 M	1.80 M	3 mM	0.66 M	$2.5X10^{-4}$
6	0.66 M	0.60 M	1.5 mM	0.66 M	$1.1X10^{-4}$
7	0.66 M	0.60 M	6 mM	0.66 M	$3.7X10^{-4}$

Graph 1. Reaction Rate vs. [2-methylfuran]

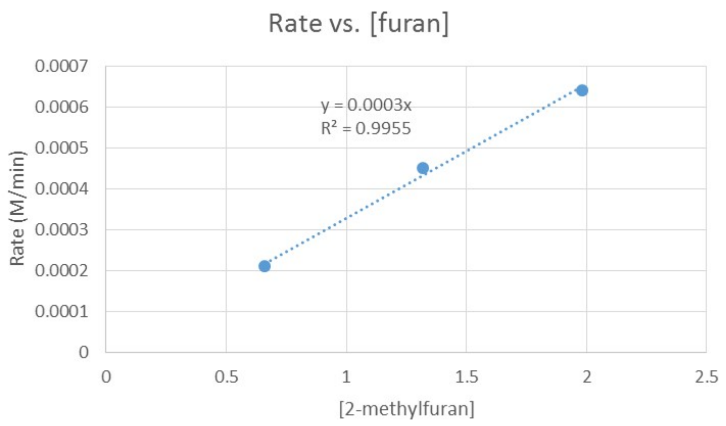

Graph 2. Reaction Rate vs. [$HMe_2Si(OEt)$]

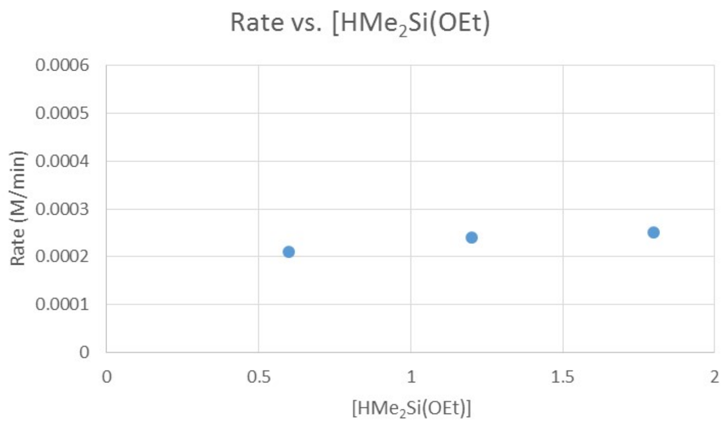

Graph 3. Reaction Rate vs. [cat. **5**]

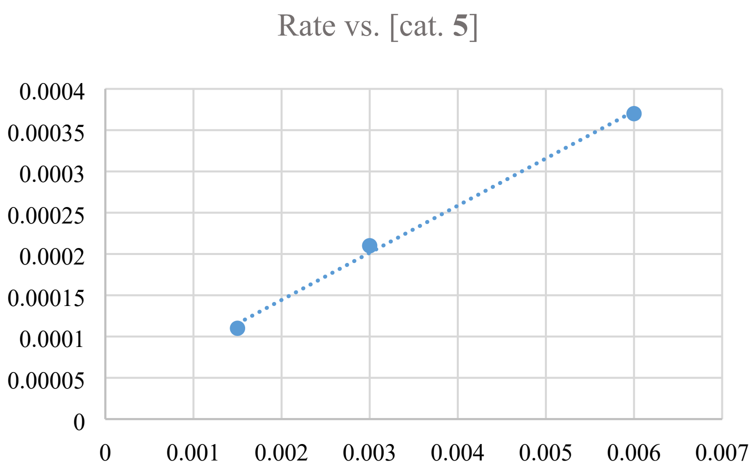

Table S7. Reaction Rate as a function of [cyclohexene]

Entry	[2-methylfuran]	[Si–H]	[cat. **5**]	[cyclohexene]	Rate (M/min)
1	0.66 M	0.60 M	3 mM	0.66 M	$2.1X10^{-4}$
8	0.66 M	0.60 M	3 mM	0.18 M	$1.3X10^{-4}$
9	0.66 M	0.60 M	3 mM	0.36 M	$1.7X10^{-4}$
10	0.66 M	0.60 M	3 mM	0.90 M	$2.1X10^{-4}$
11	0.66 M	0.60 M	3 mM	1.20 M	suppressed
12	0.66 M	0.60 M	3 mM	1.50 M	suppressed
13	0.66 M	0.60 M	3 mM	1.80 M	suppressed

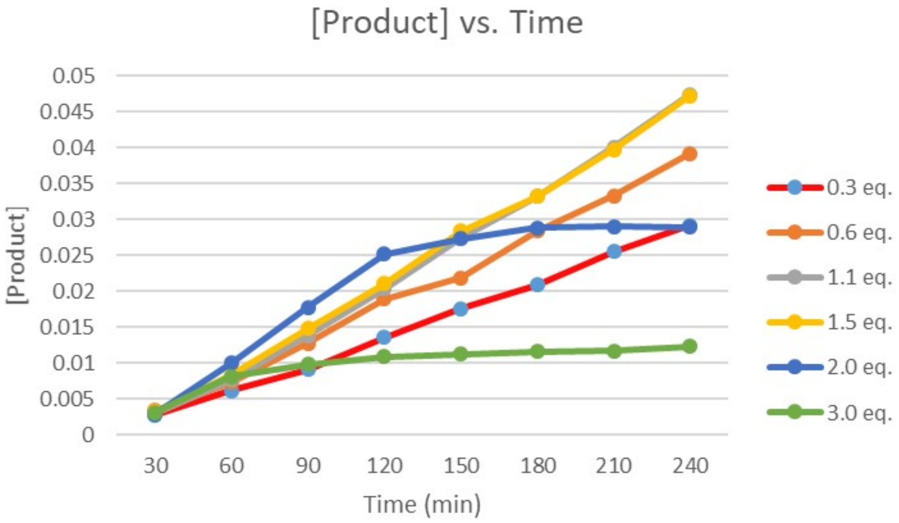
[Product] vs. Time
0.05
0.045
0.04
0.035
0.03
0.025
0.02
0.015
0.01
0.005
0
[Product]
30
60
90
120
150
180
210
240
Time (min)
0.3 eq.
0.6 eq.
1.1 eq.
1.5 eq.
2.0 eq.
3.0 eq.

5. NMR Spectra

5.1 C–H Silylation Products

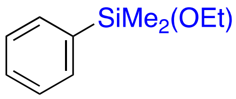

^{1}H NMR of **P2**

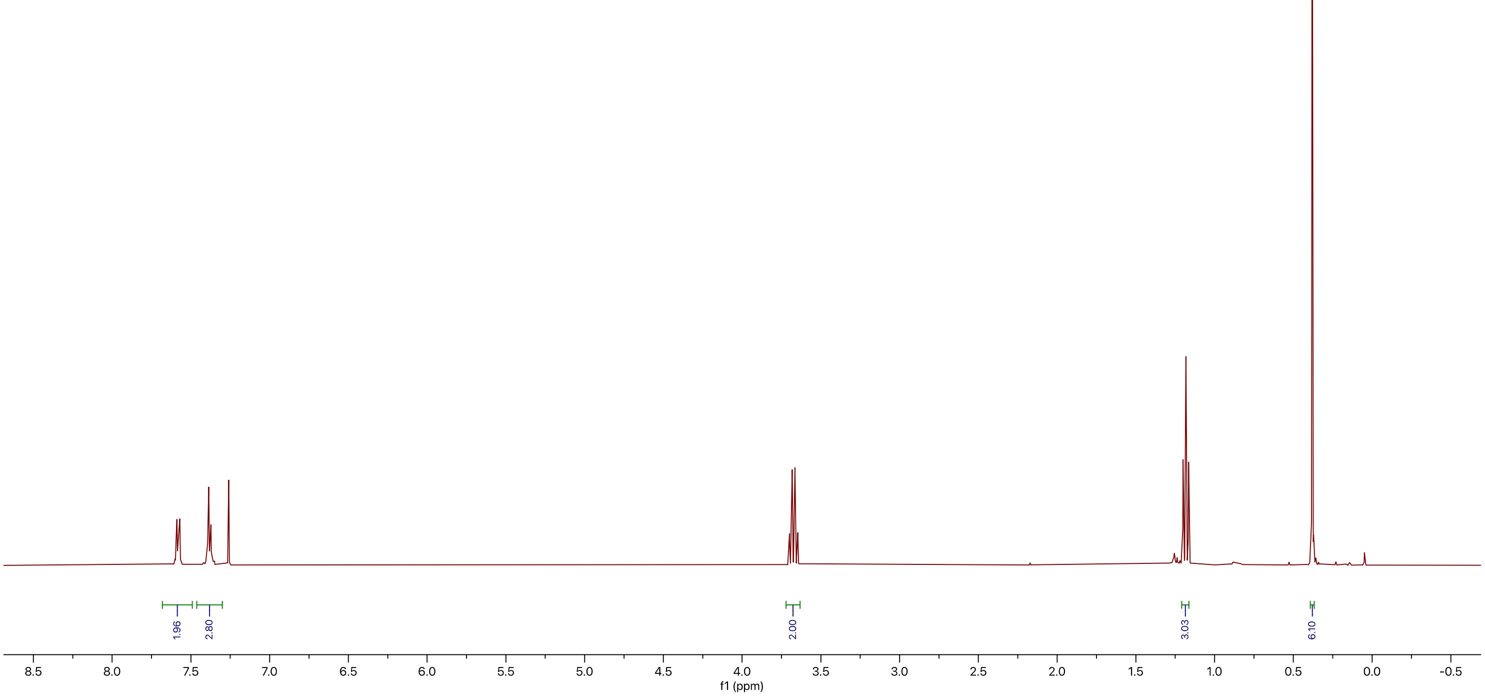

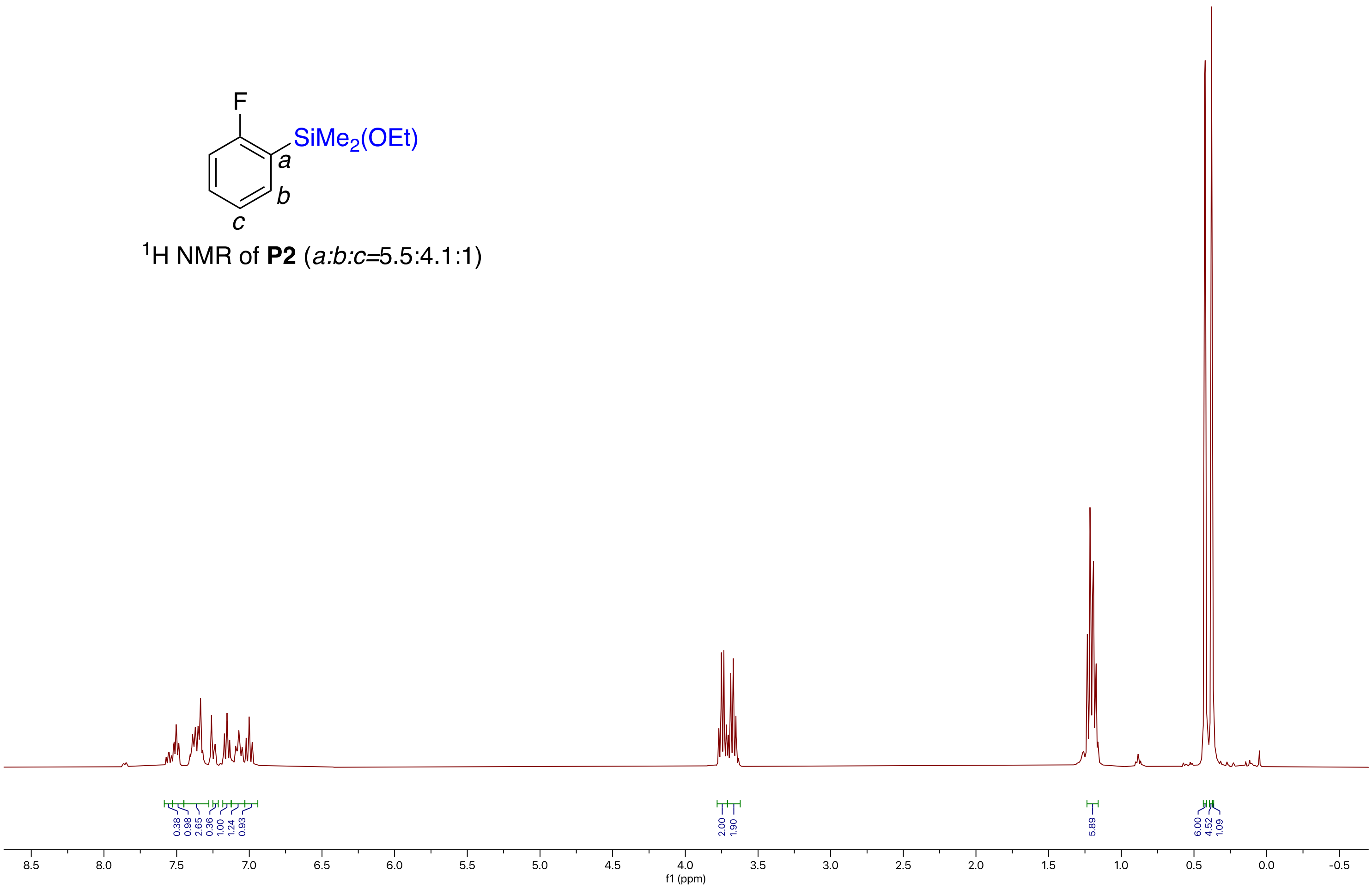
F
SiMe2(OEt)
a
b
c
1H NMR of P2 (a:b:c=5.5:4.1:1)
0.38
0.98
2.65
0.36
1.00
1.24
0.93
2.00
1.90
5.89
6.00
4.52
1.09
8.5
8.0
7.5
7.0
6.5
6.0
5.5
5.0
4.5
4.0
3.5
3.0
2.5
2.0
1.5
1.0
0.5
0.0
-0.5
f1 (ppm)

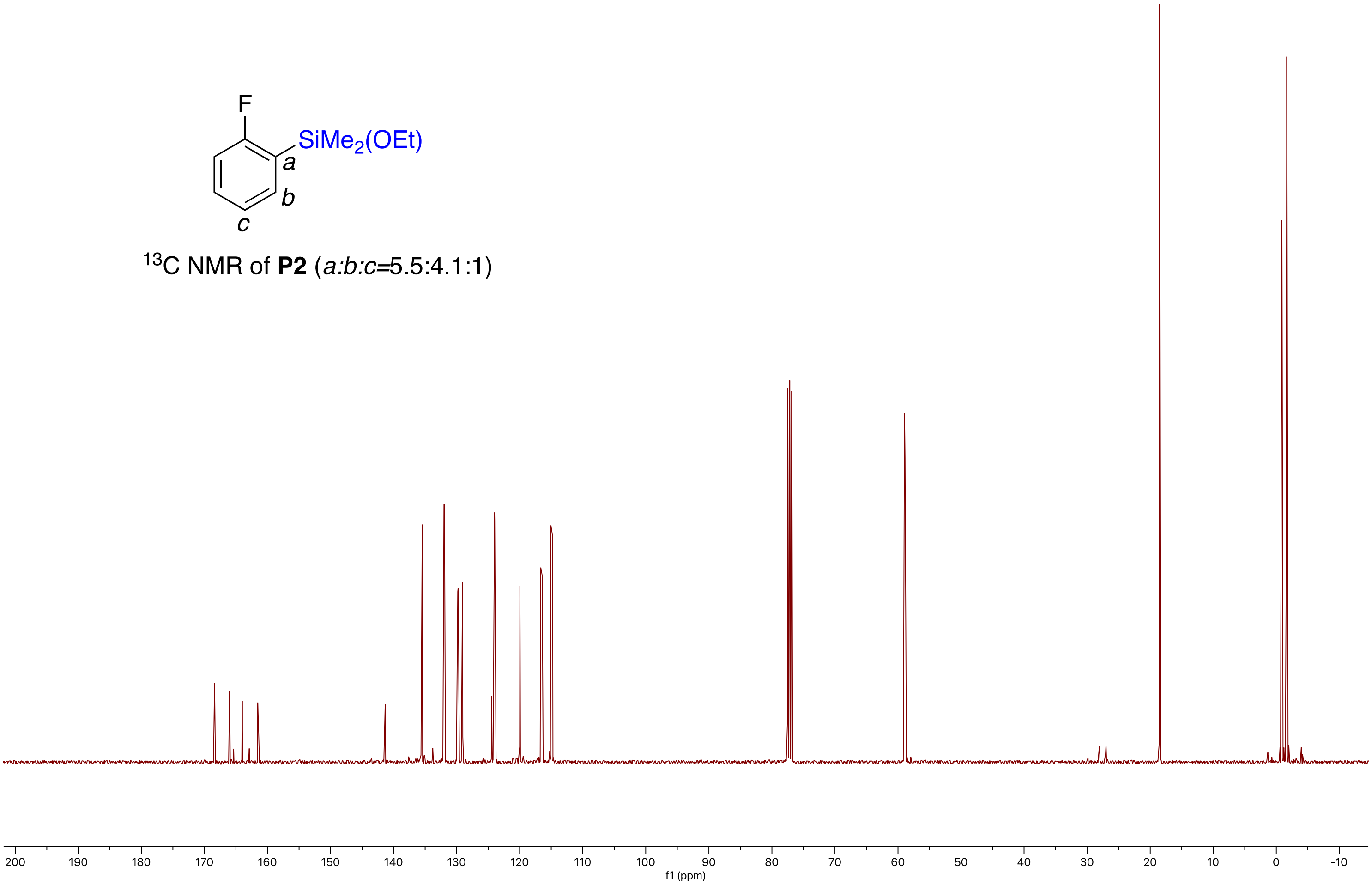
F
SiMe2(OEt)
a
b
c
13C NMR of P2 (a:b:c=5.5:4.1:1)
200
190
180
170
160
150
140
130
120
110
100
90
80
70
60
50
40
30
20
10
0
-10
f1 (ppm)

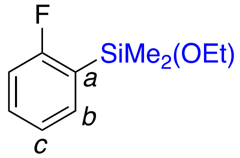

^{19}F NMR of **P2** (*a*:*b*:*c*=5.5:4.1:1)

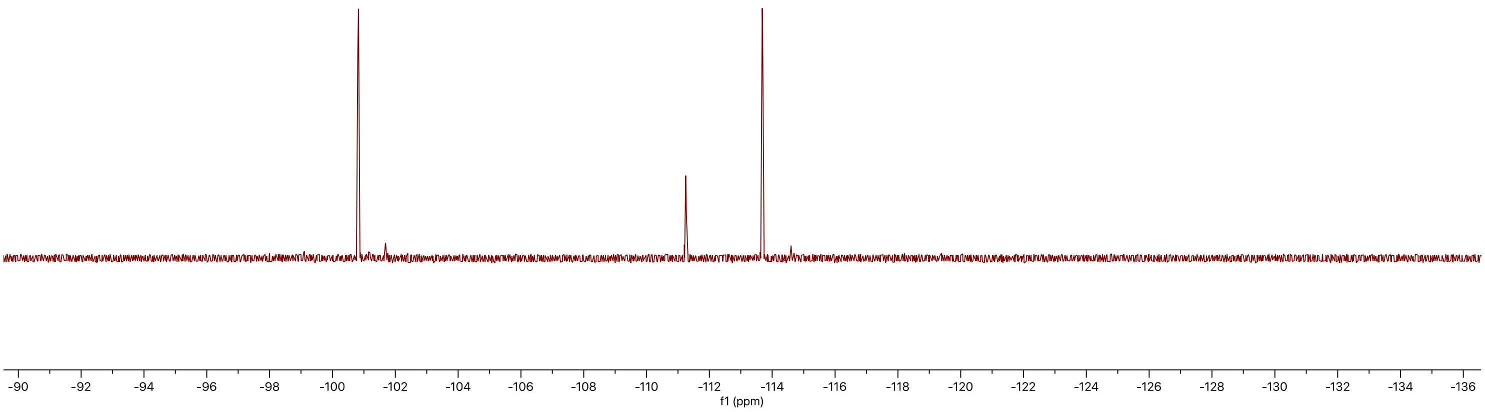

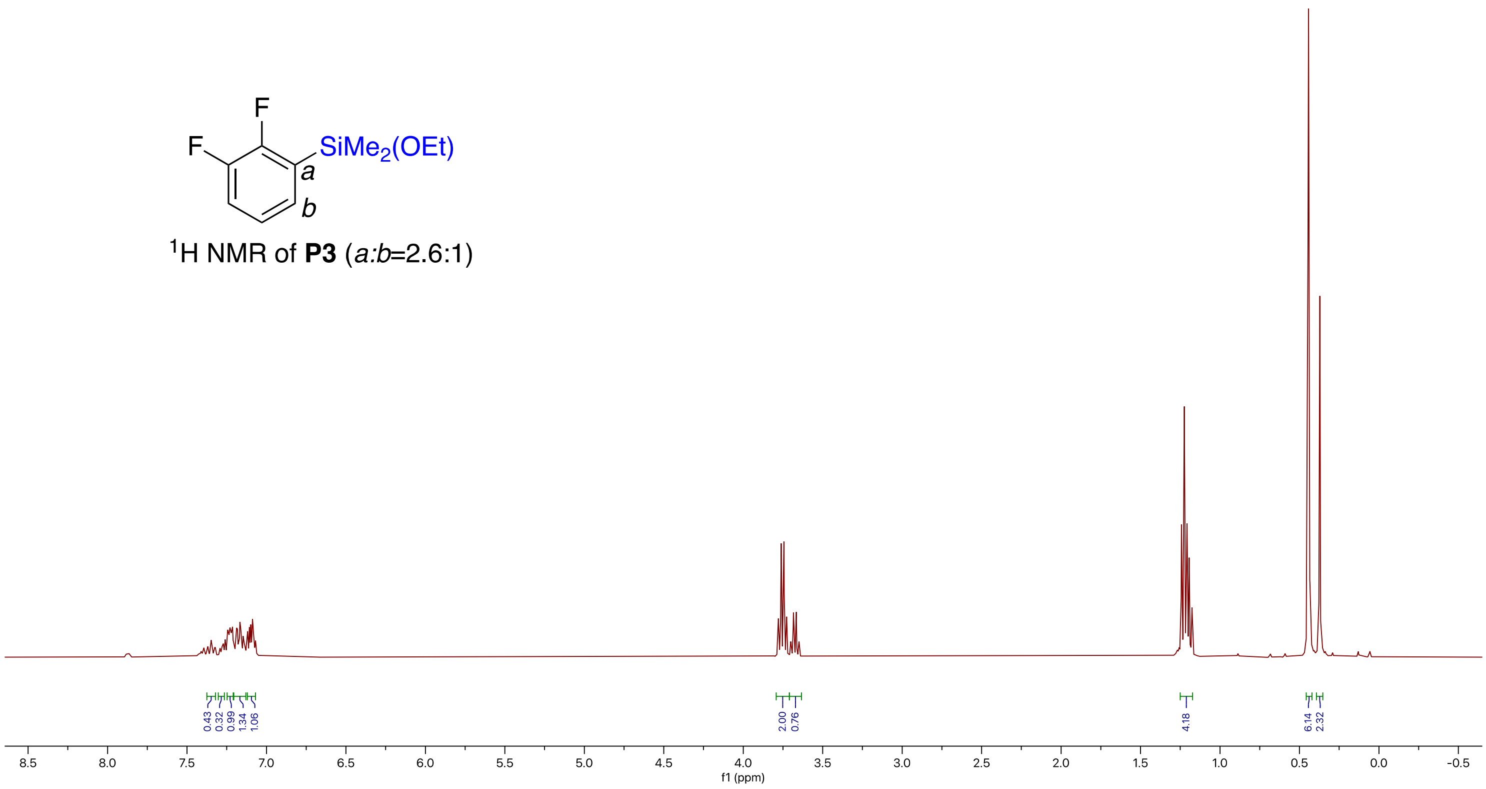
F
F
SiMe2(OEt)
a
b
1H NMR of P3 (a:b=2.6:1)
0.43
0.32
0.99
1.34
1.06
2.00
0.76
4.18
6.14
2.32
f1 (ppm)

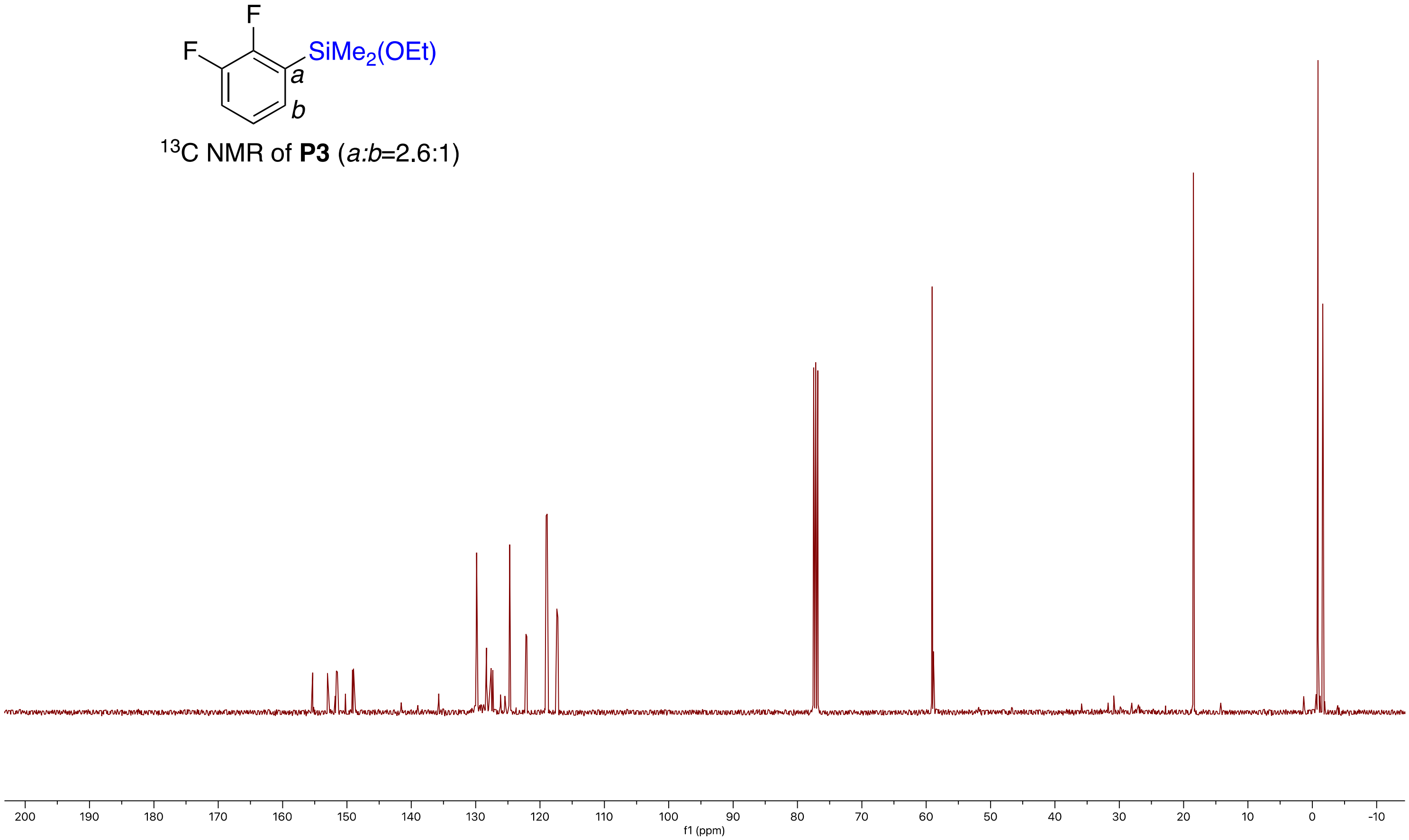
F
F
SiMe2(OEt)
a
b
13C NMR of P3 (a:b=2.6:1)
f1 (ppm)

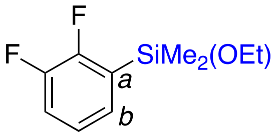

^{19}F NMR of **P3** (*a*:*b*=2.6:1)

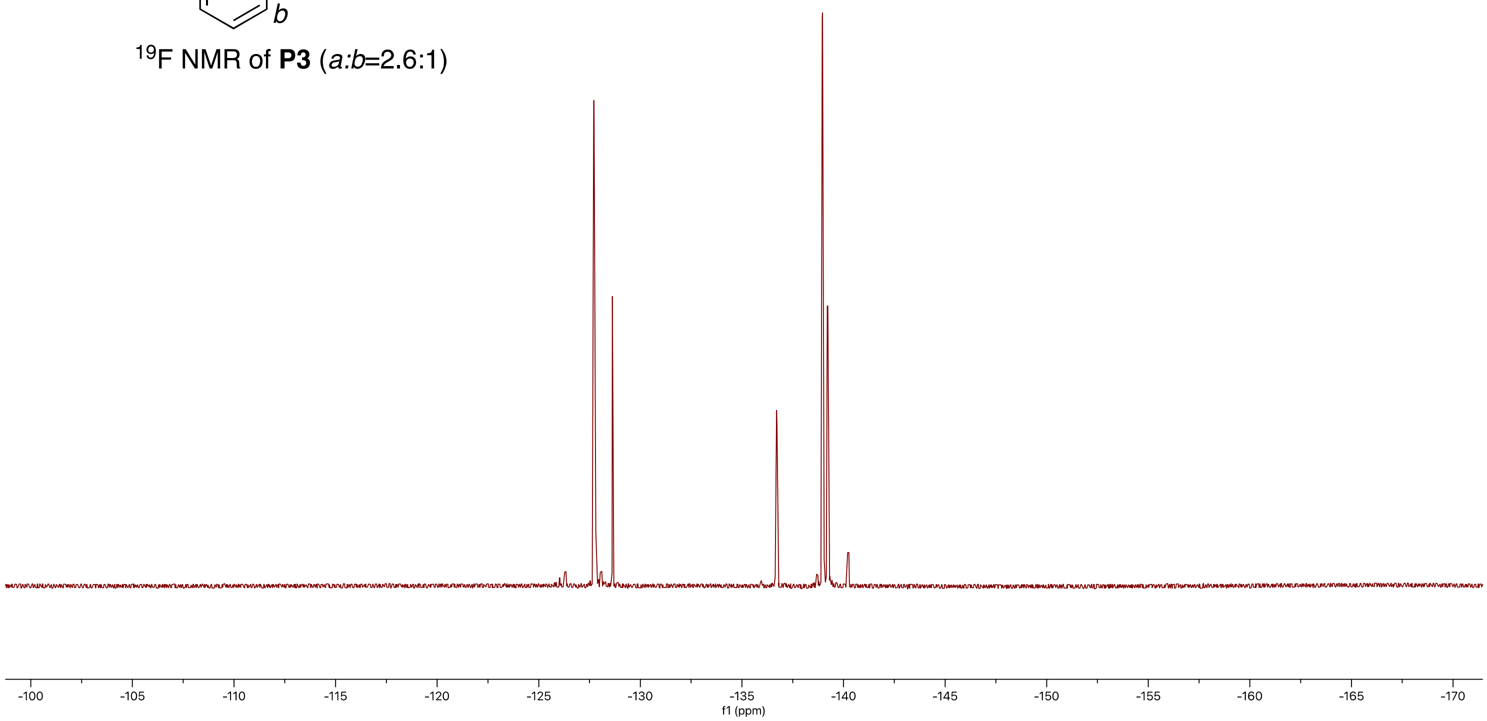

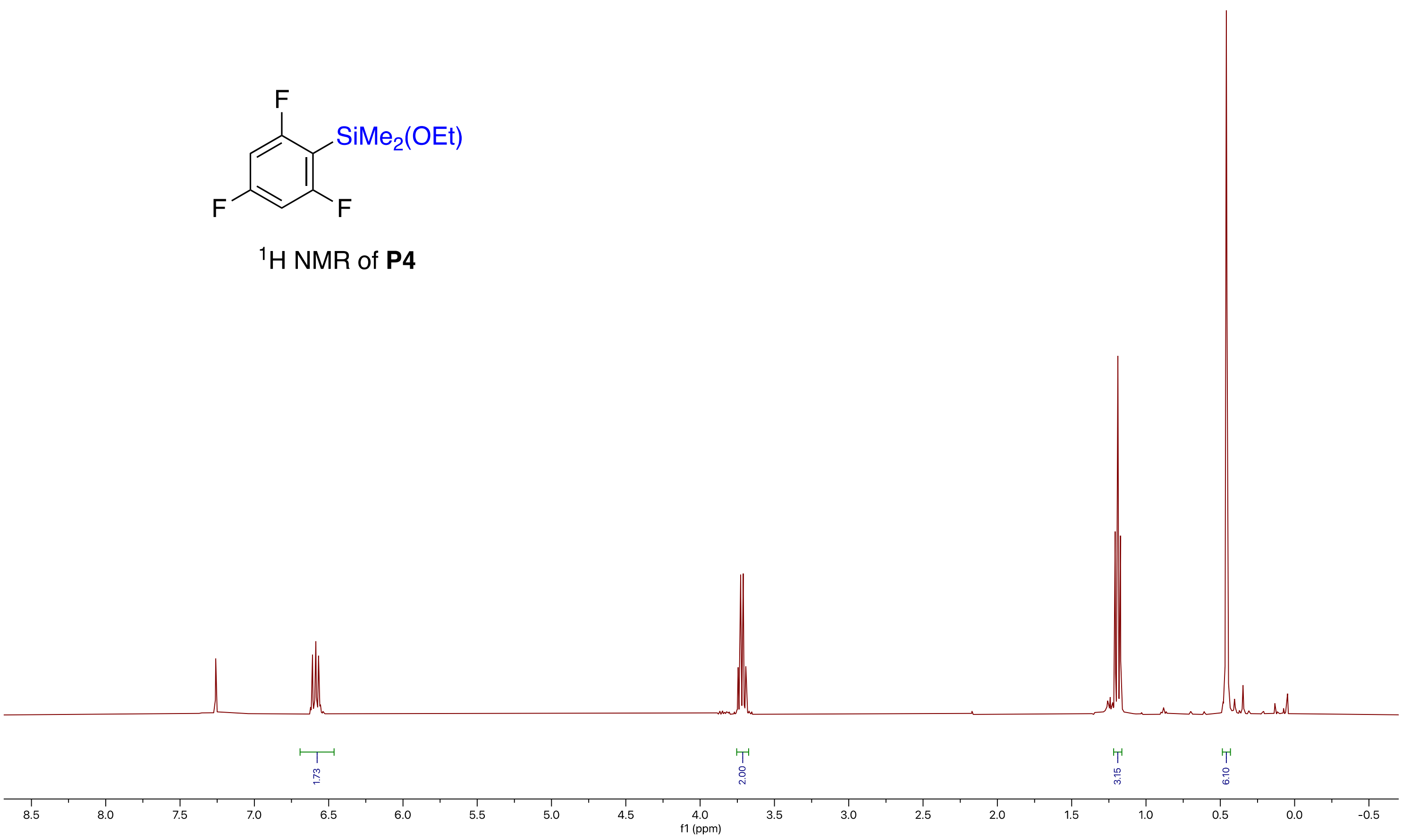
SiMe2(OEt)
F
F
F
1H NMR of P4
1.73
2.00
3.15
6.10
f1 (ppm)

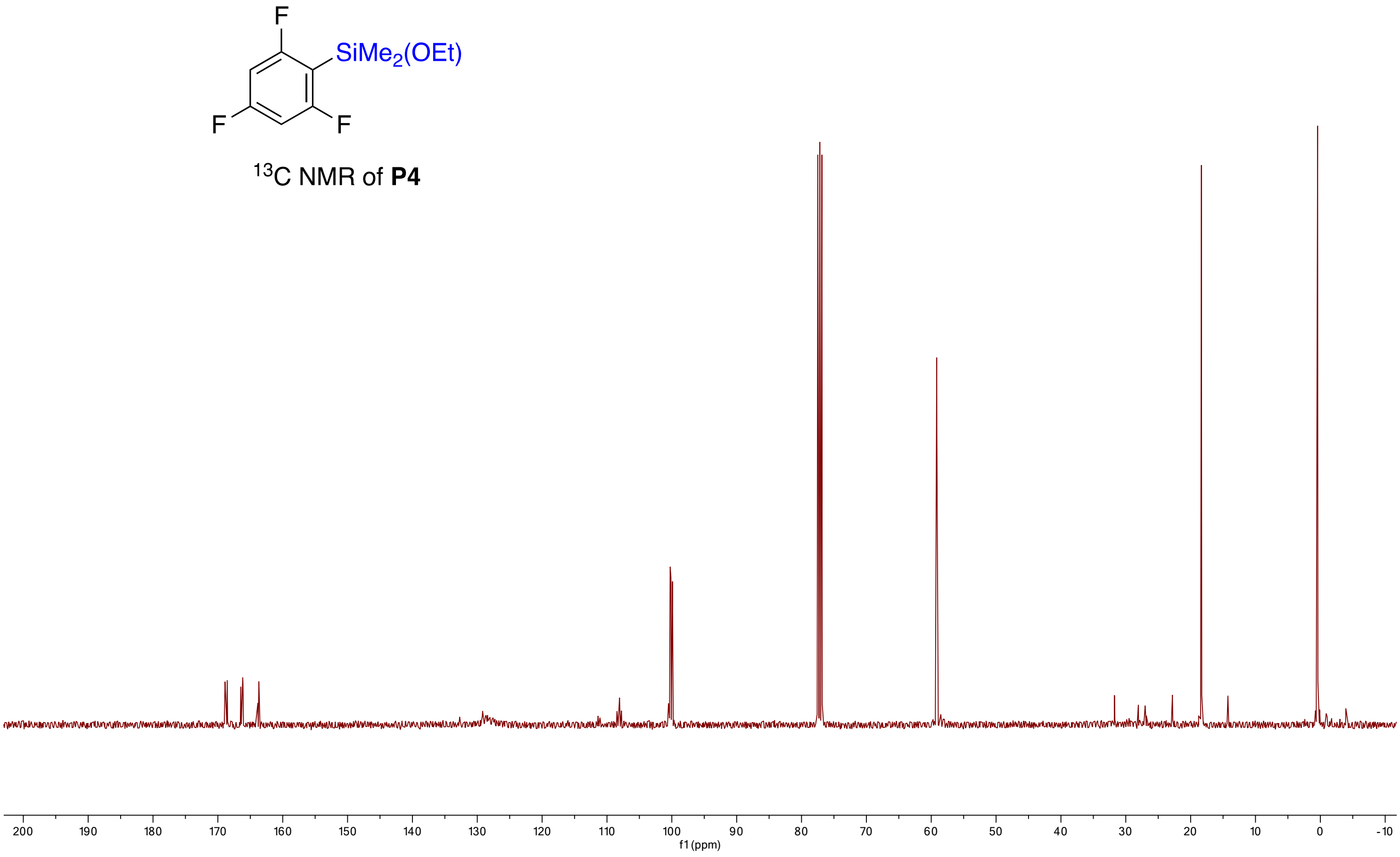
SiMe2(OEt)
F
F
F
13C NMR of P4
f1 (ppm)

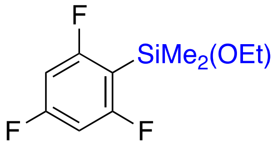
F
SiMe2(OEt)
F
F

^{19}F NMR of **P4**

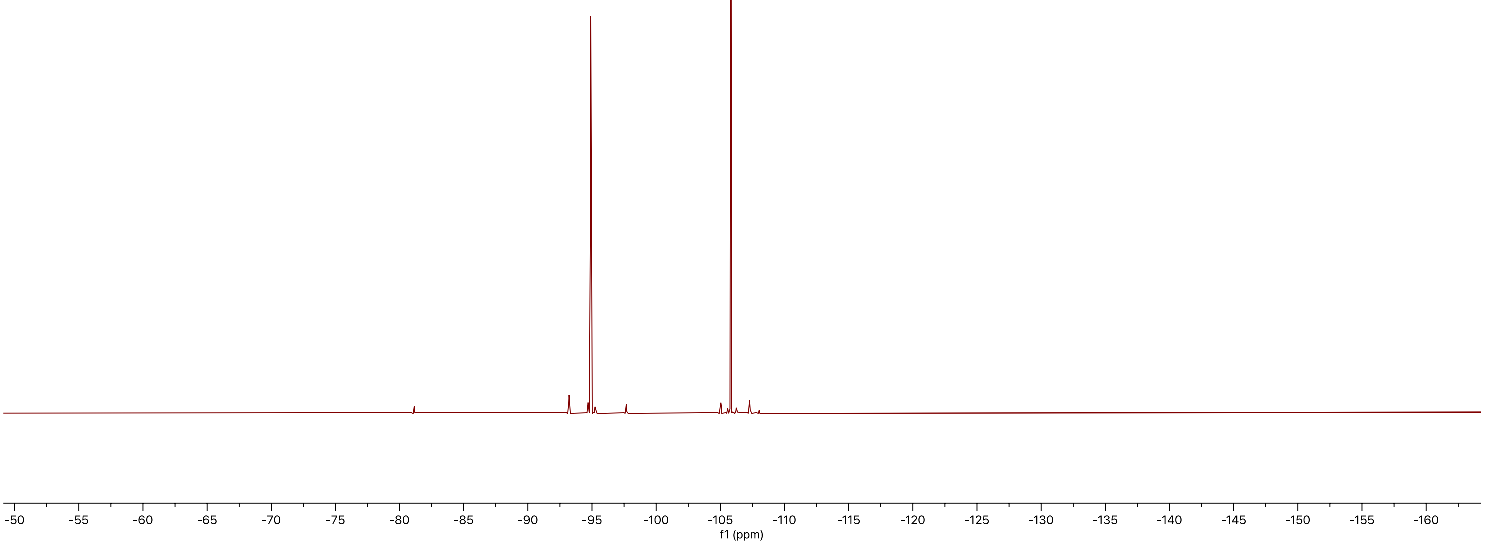
-50
-55
-60
-65
-70
-75
-80
-85
-90
-95
-100
-105
-110
-115
-120
-125
-130
-135
-140
-145
-150
-155
-160
f1 (ppm)

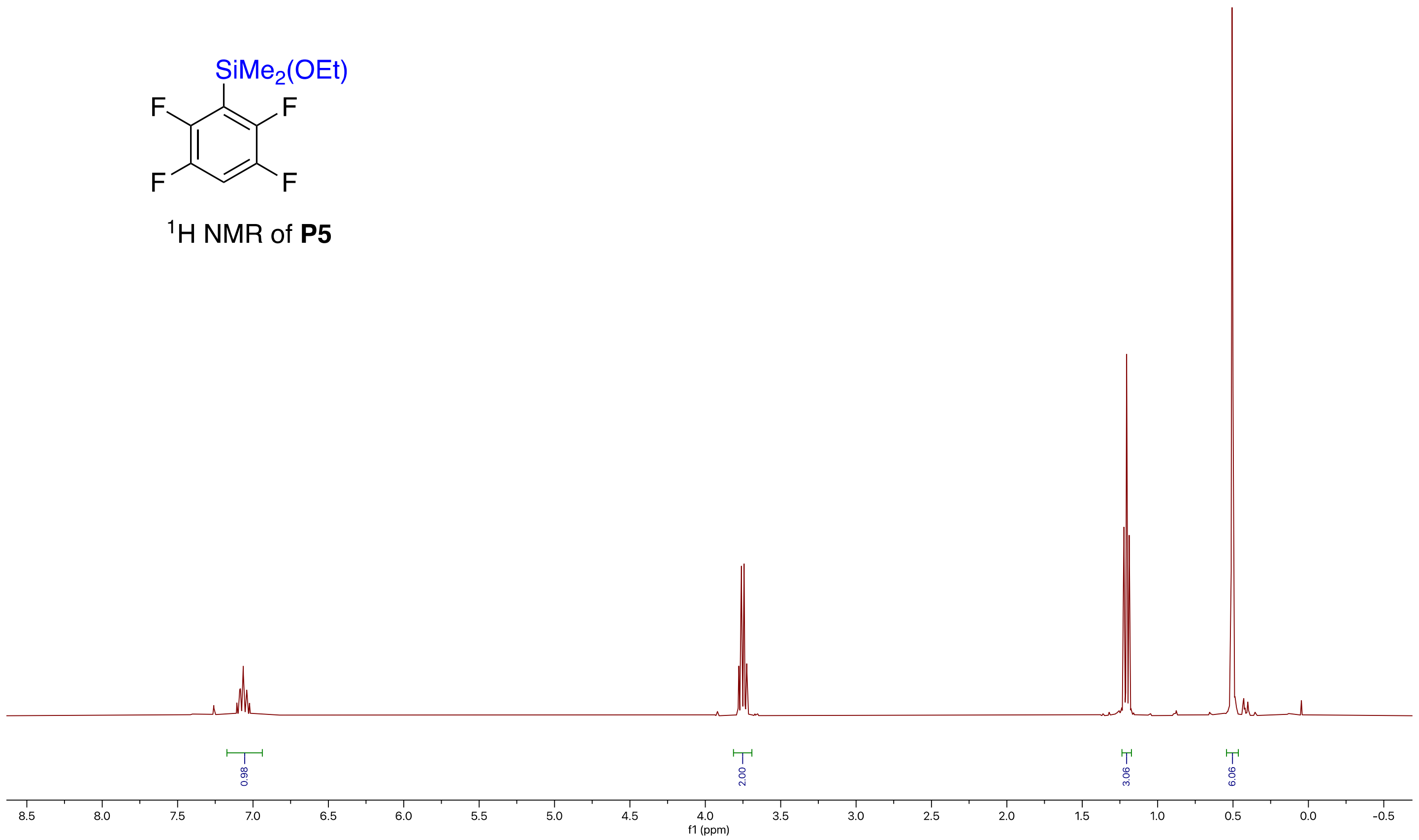
SiMe2(OEt)
F
F
F
F
1H NMR of P5
0.98
2.00
3.06
6.06
f1 (ppm)

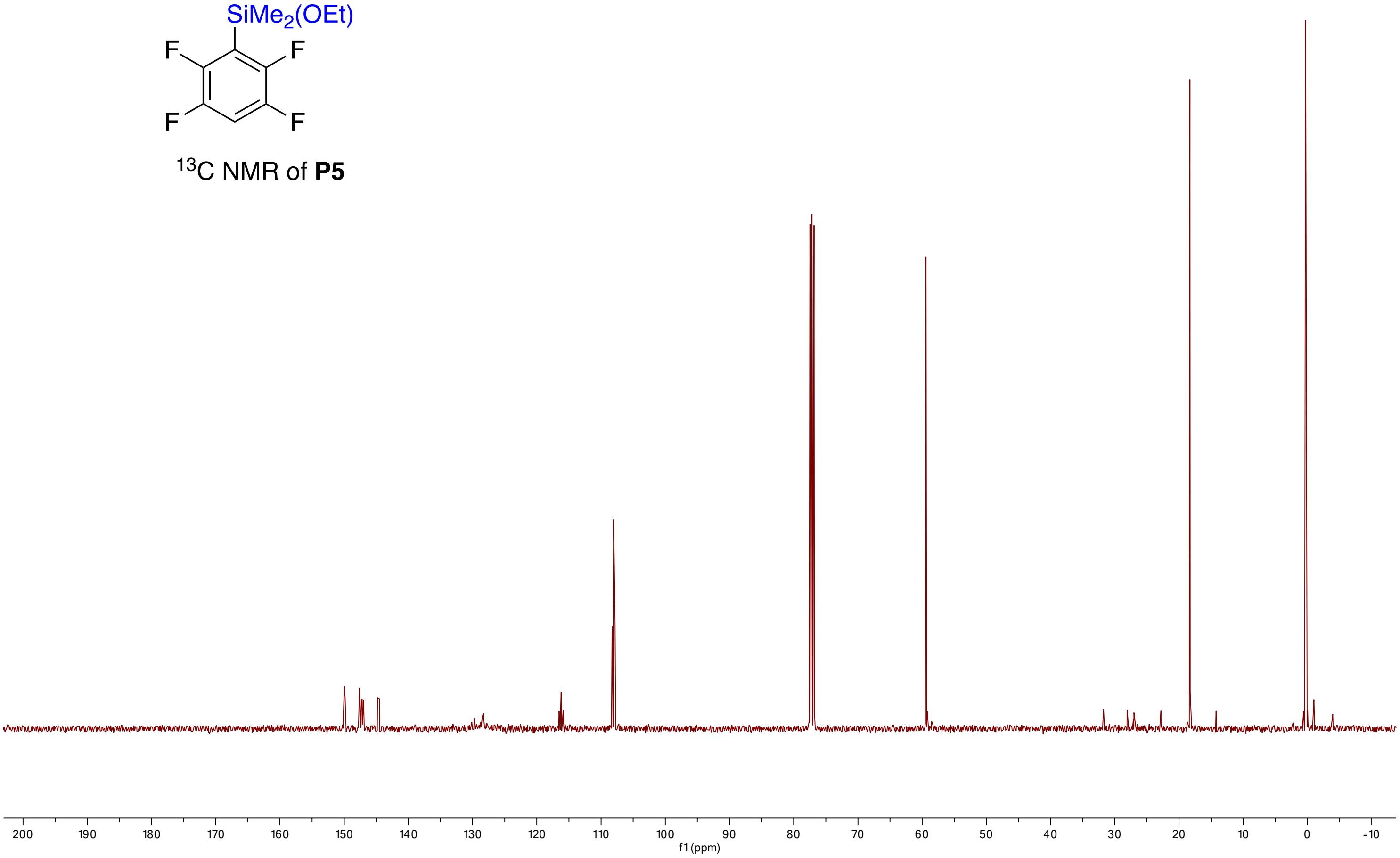
SiMe2(OEt)
F
F
F
F
13C NMR of P5
f1 (ppm)

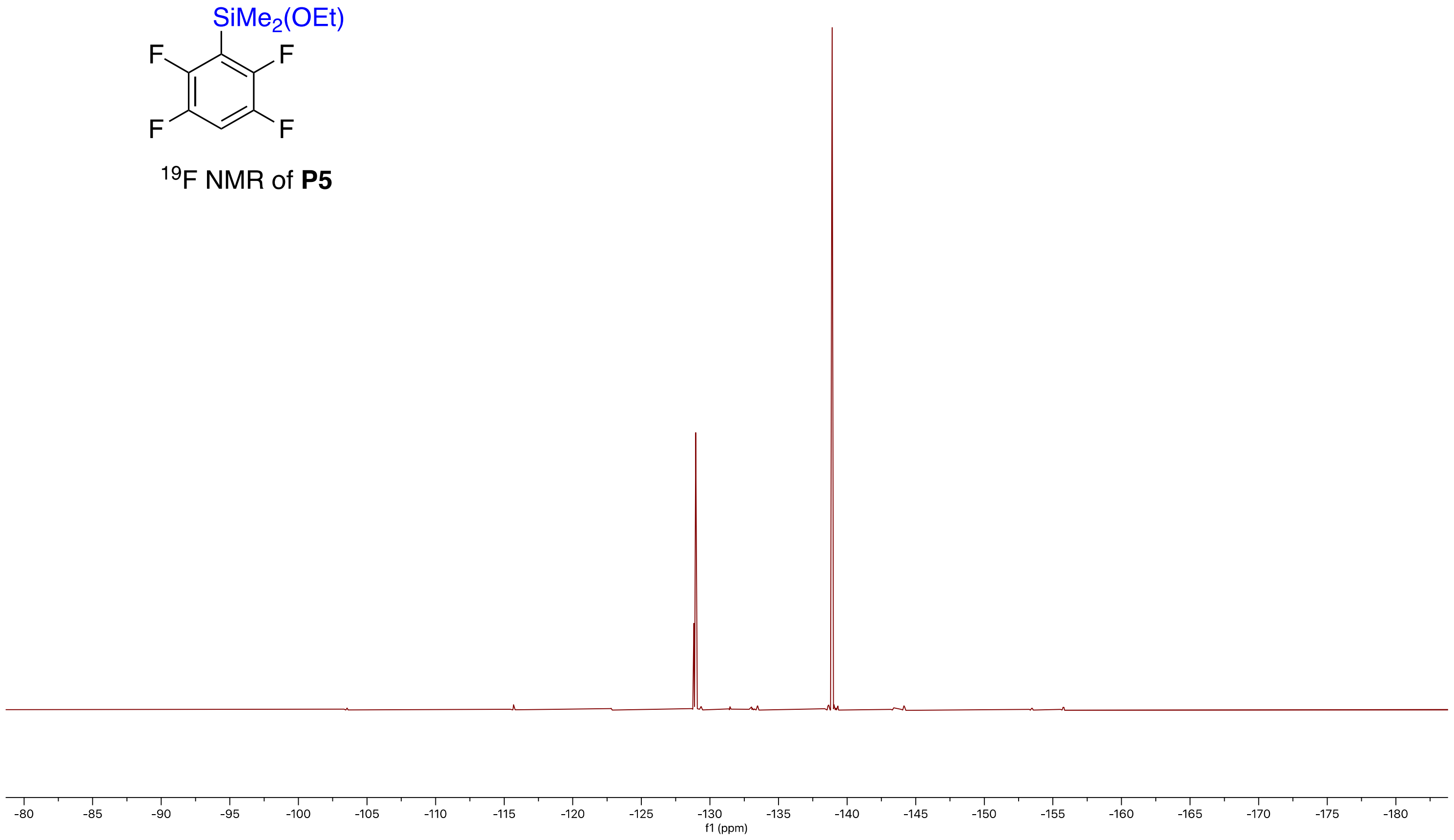
SiMe2(OEt)
F
F
F
F
19F NMR of P5
-80
-85
-90
-95
-100
-105
-110
-115
-120
-125
-130
-135
-140
-145
-150
-155
-160
-165
-170
-175
-180
f1 (ppm)

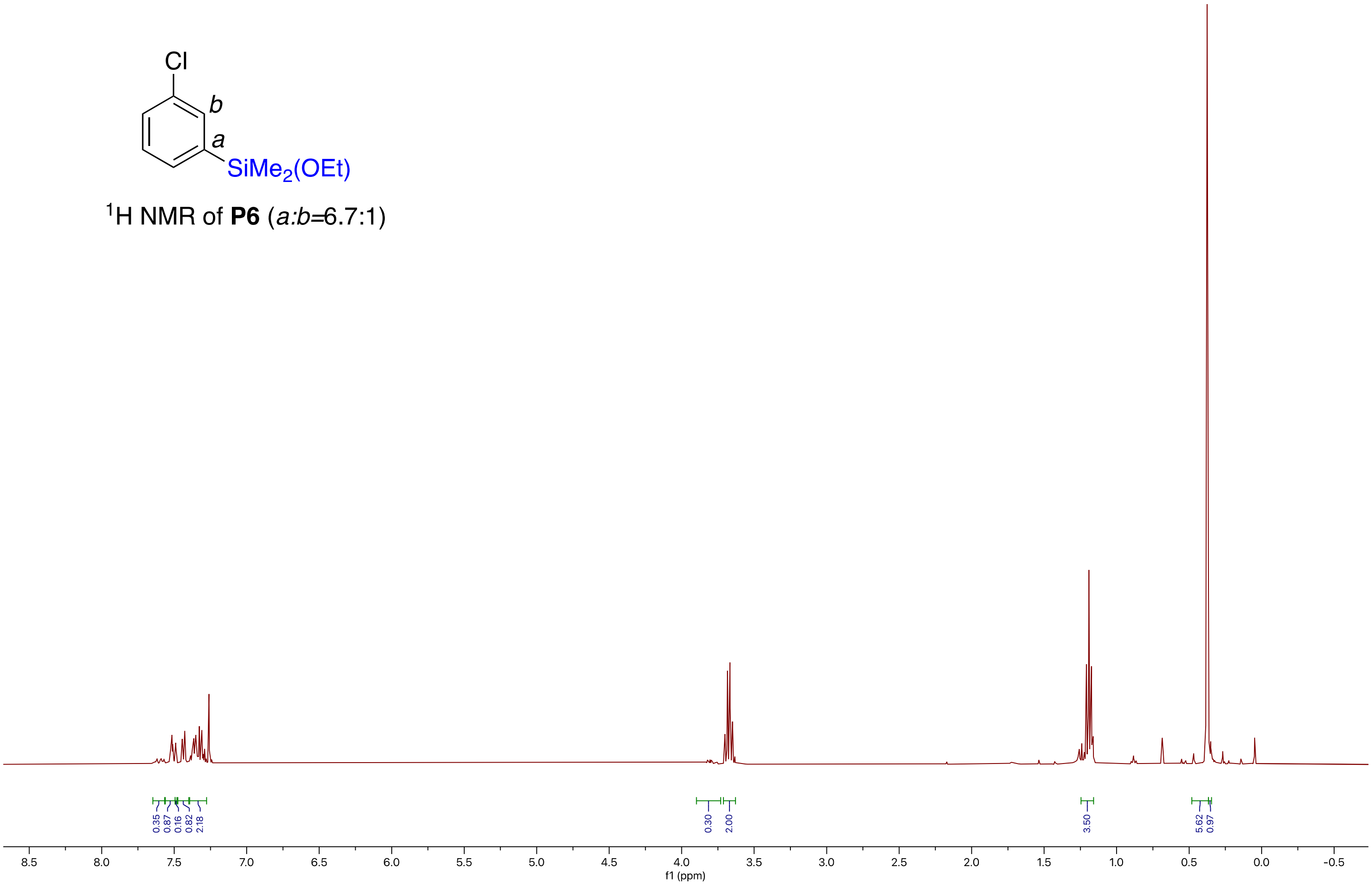

^{1}H NMR of **P6** (*a*:*b*=6.7:1)

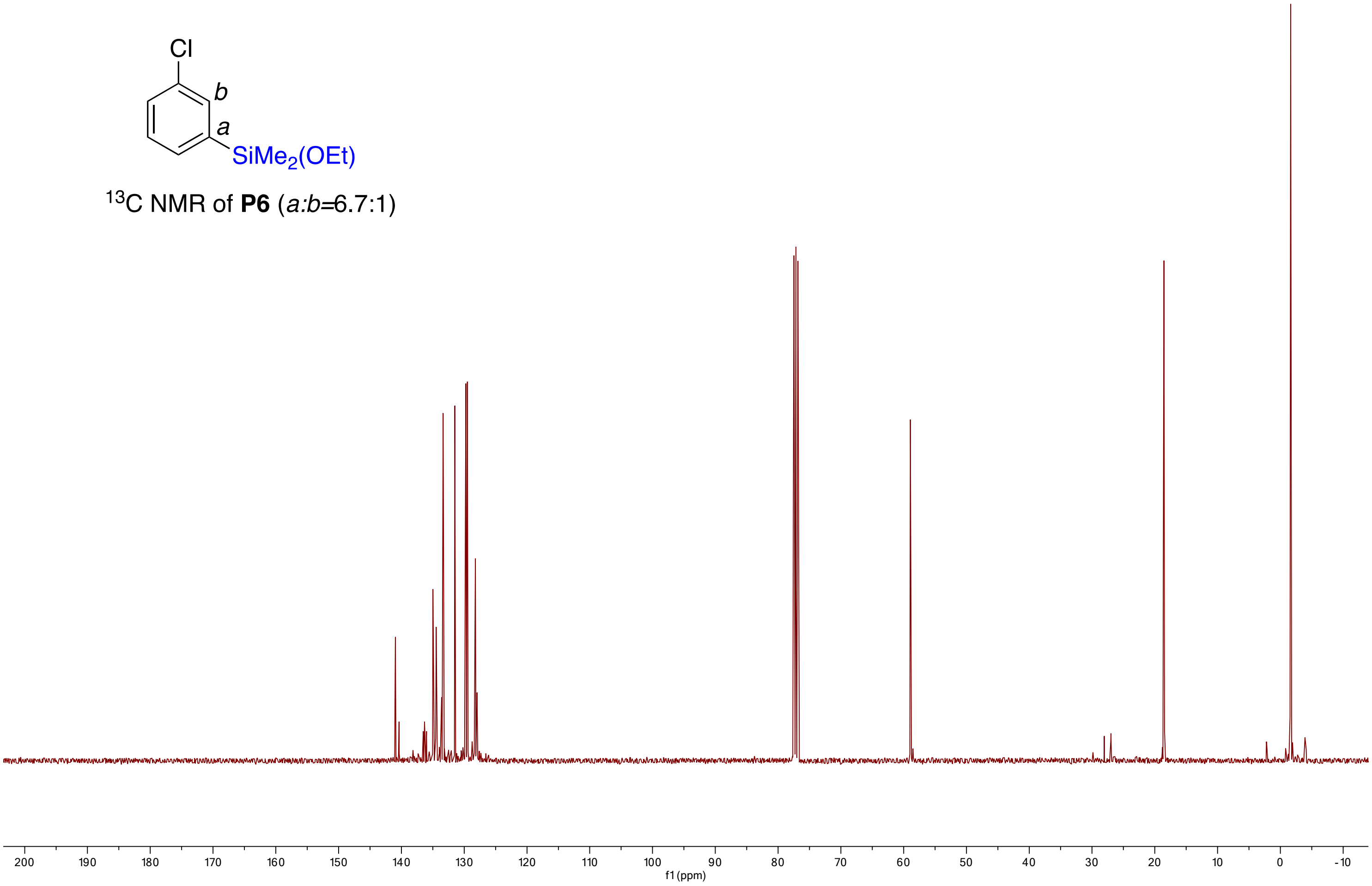

^{13}C NMR of **P6** (*a*:*b*=6.7:1)

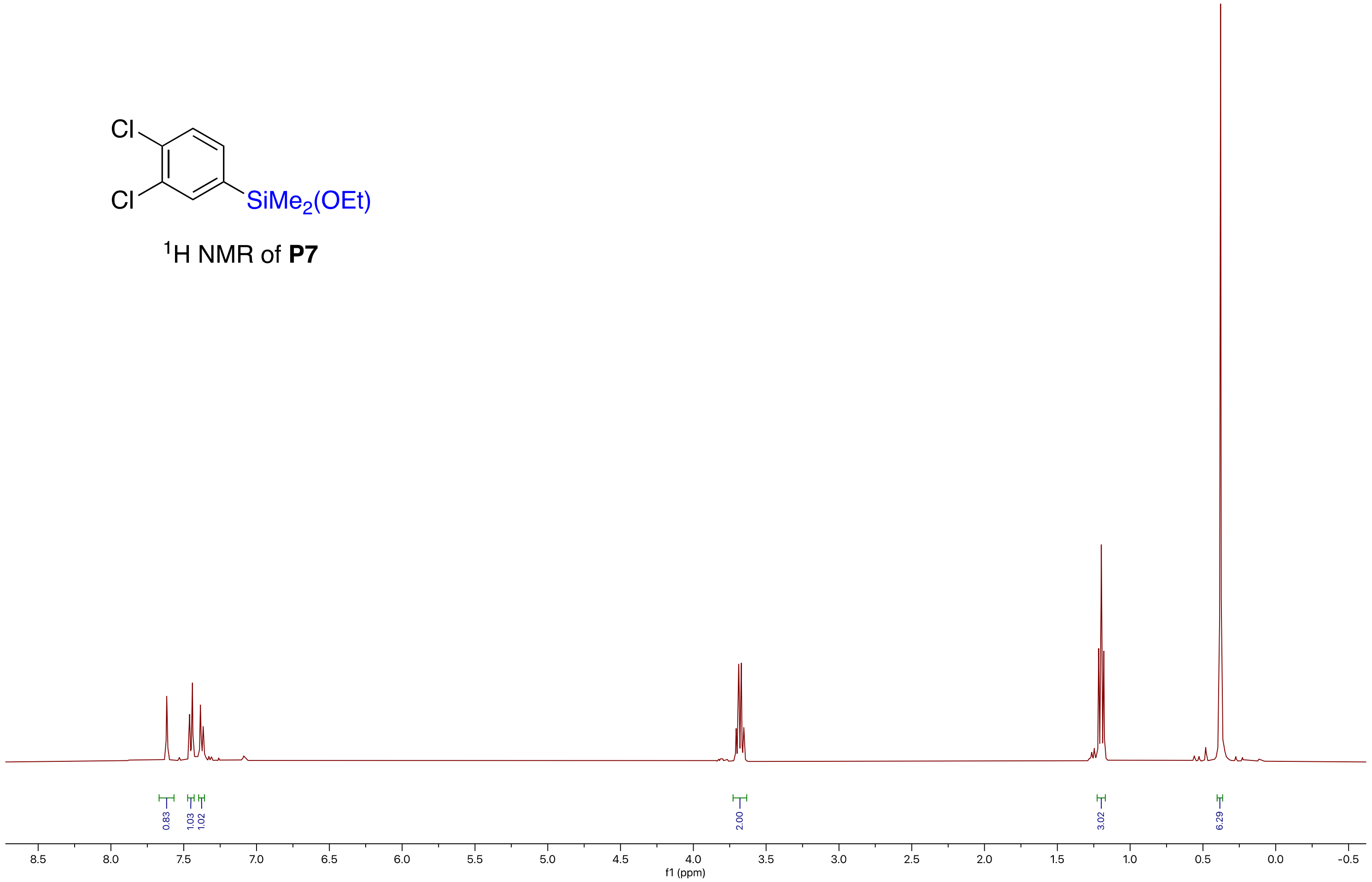
Cl
Cl
SiMe2(OEt)
1H NMR of P7
0.83
1.03
1.02
2.00
3.02
6.29
f1 (ppm)

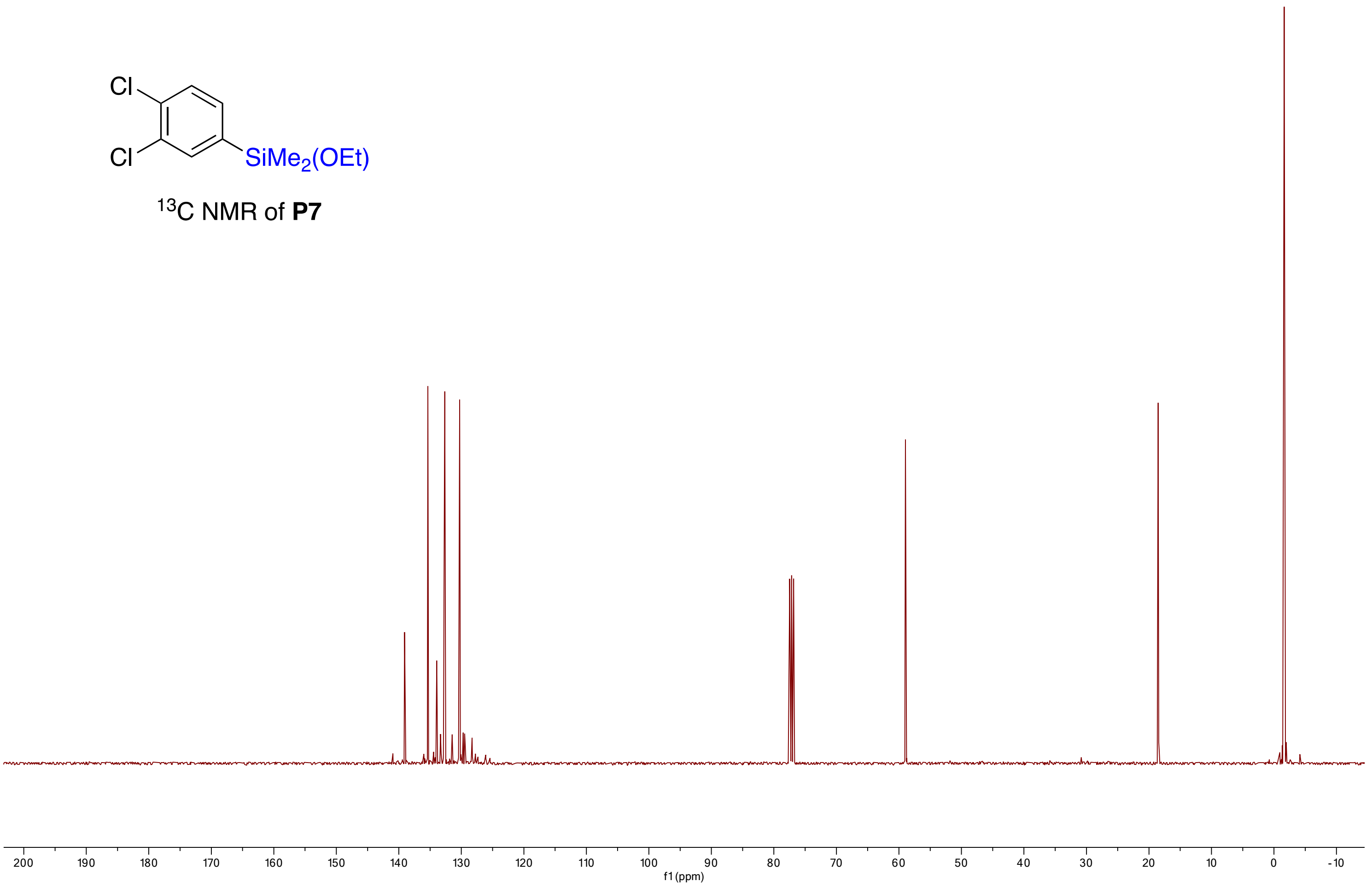
Cl
Cl
SiMe2(OEt)
13C NMR of P7
f1 (ppm)

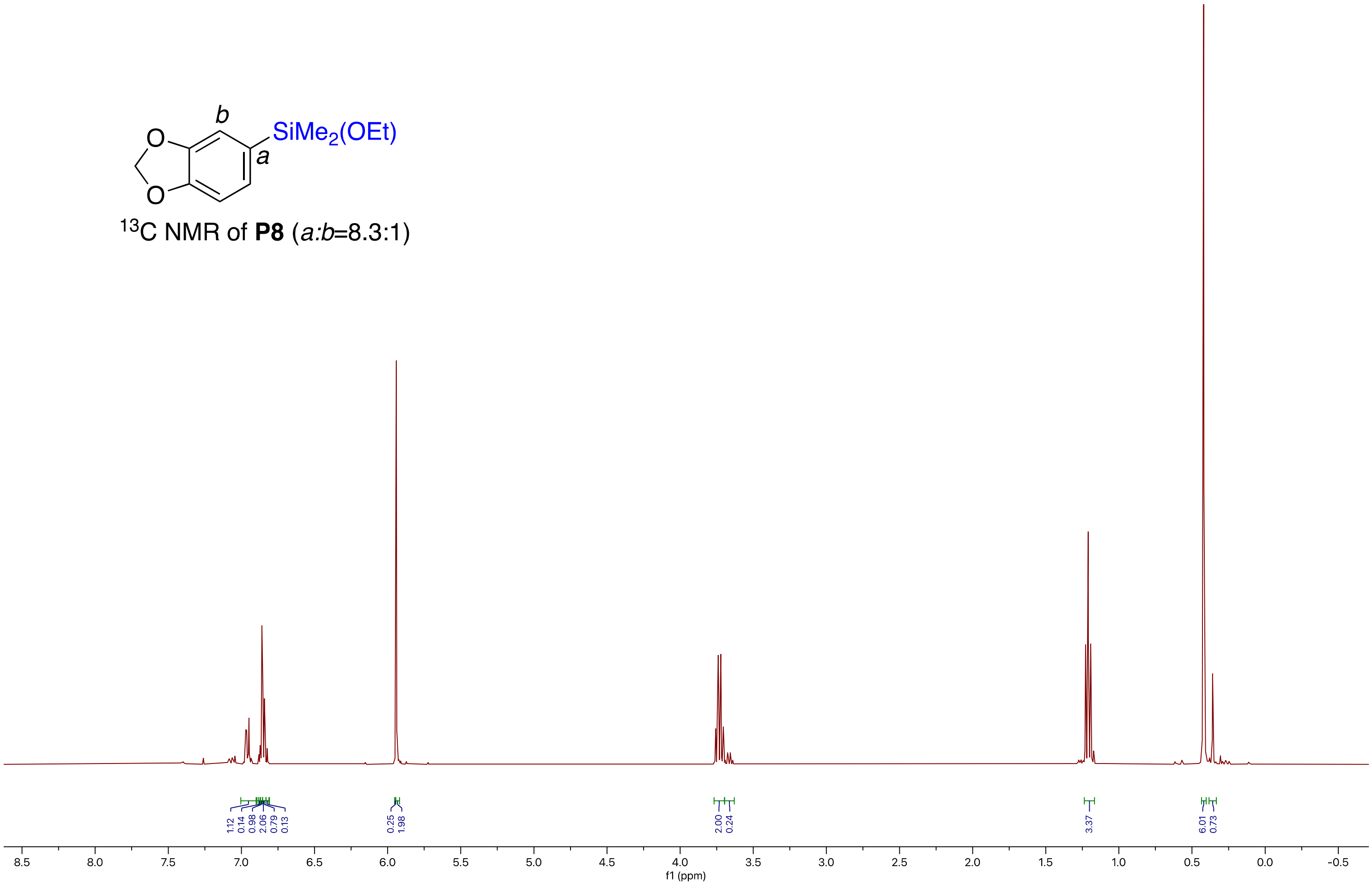

^{13}C NMR of **P8** (*a*:*b*=8.3:1)

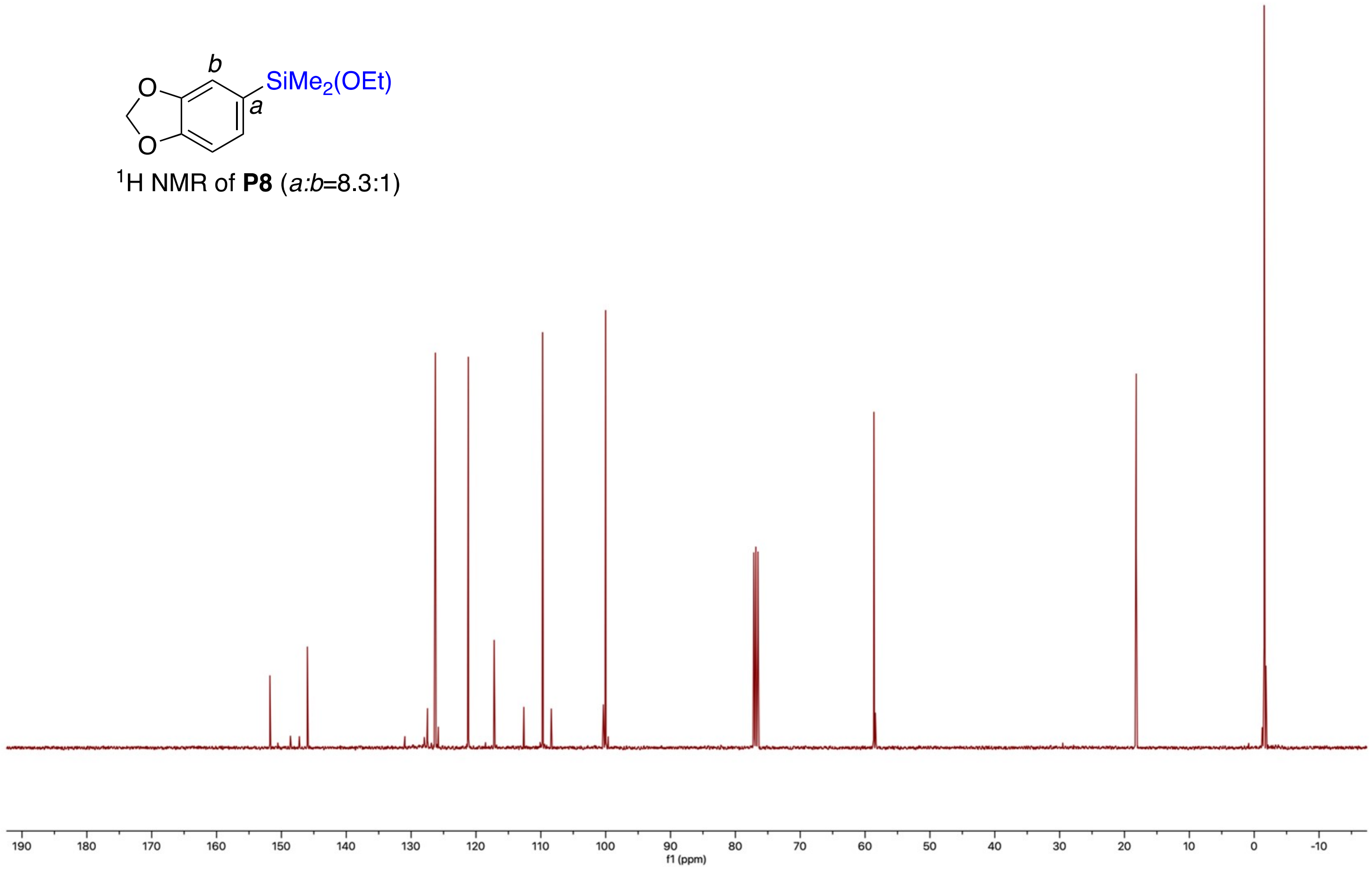

^{1}H NMR of **P8** (*a*:*b*=8.3:1)

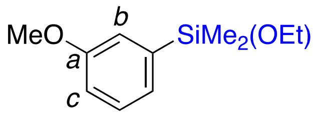

^{1}H NMR of **P9** (*a*:*b*:*c*=4.8:1.3:1)

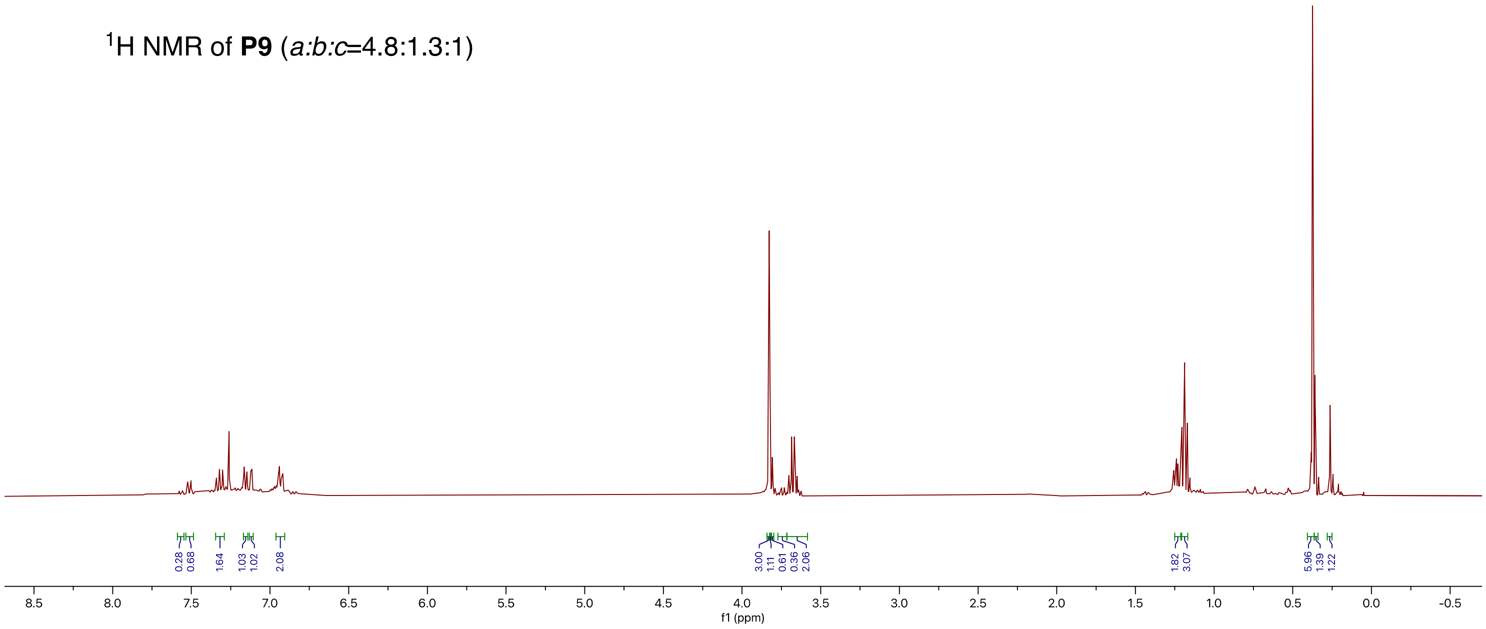

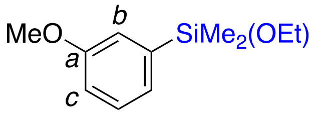

^{13}C NMR of **P9** (*a*:*b*:*c*=4.8:1.3:1)

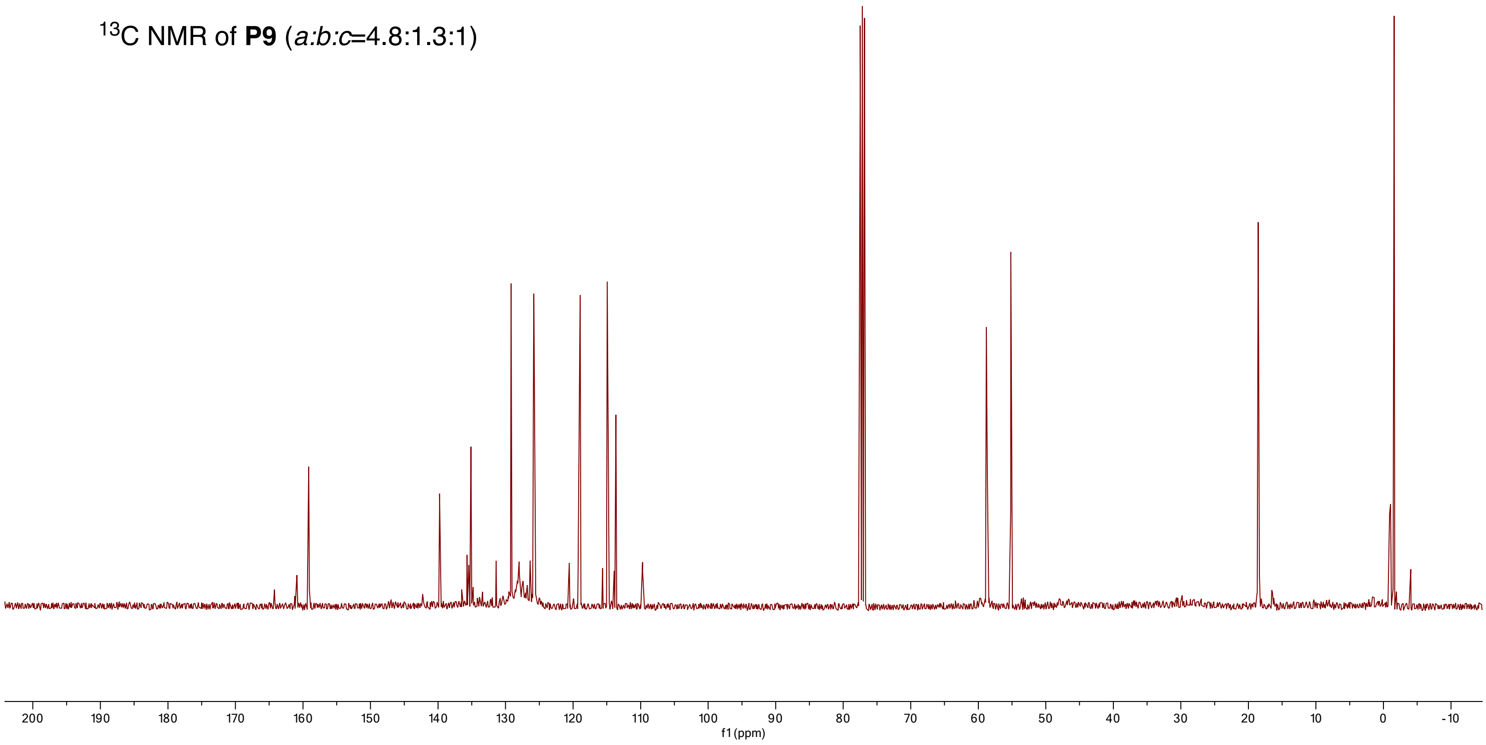

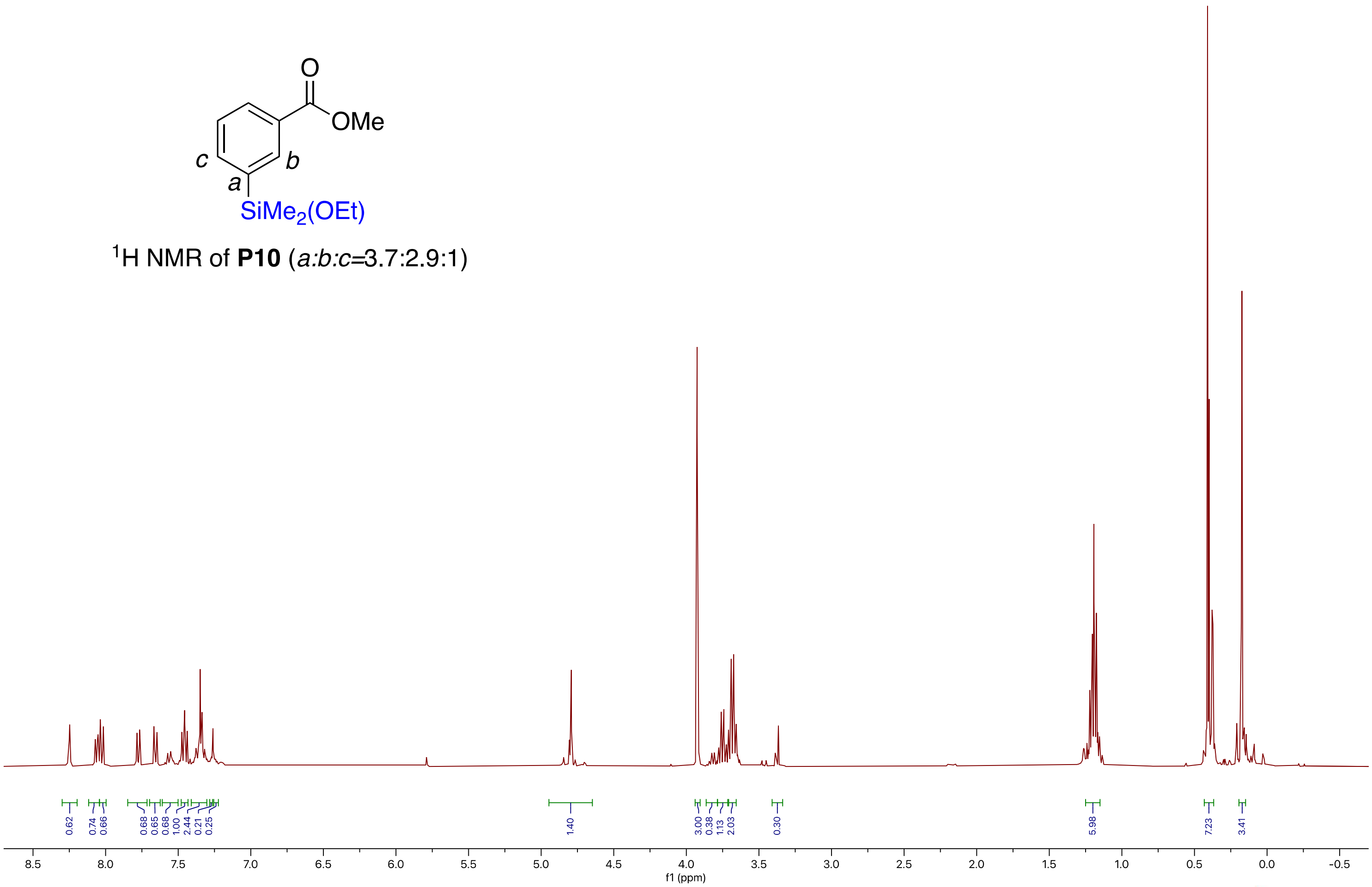

^{1}H NMR of **P10** (*a*:*b*:*c*=3.7:2.9:1)

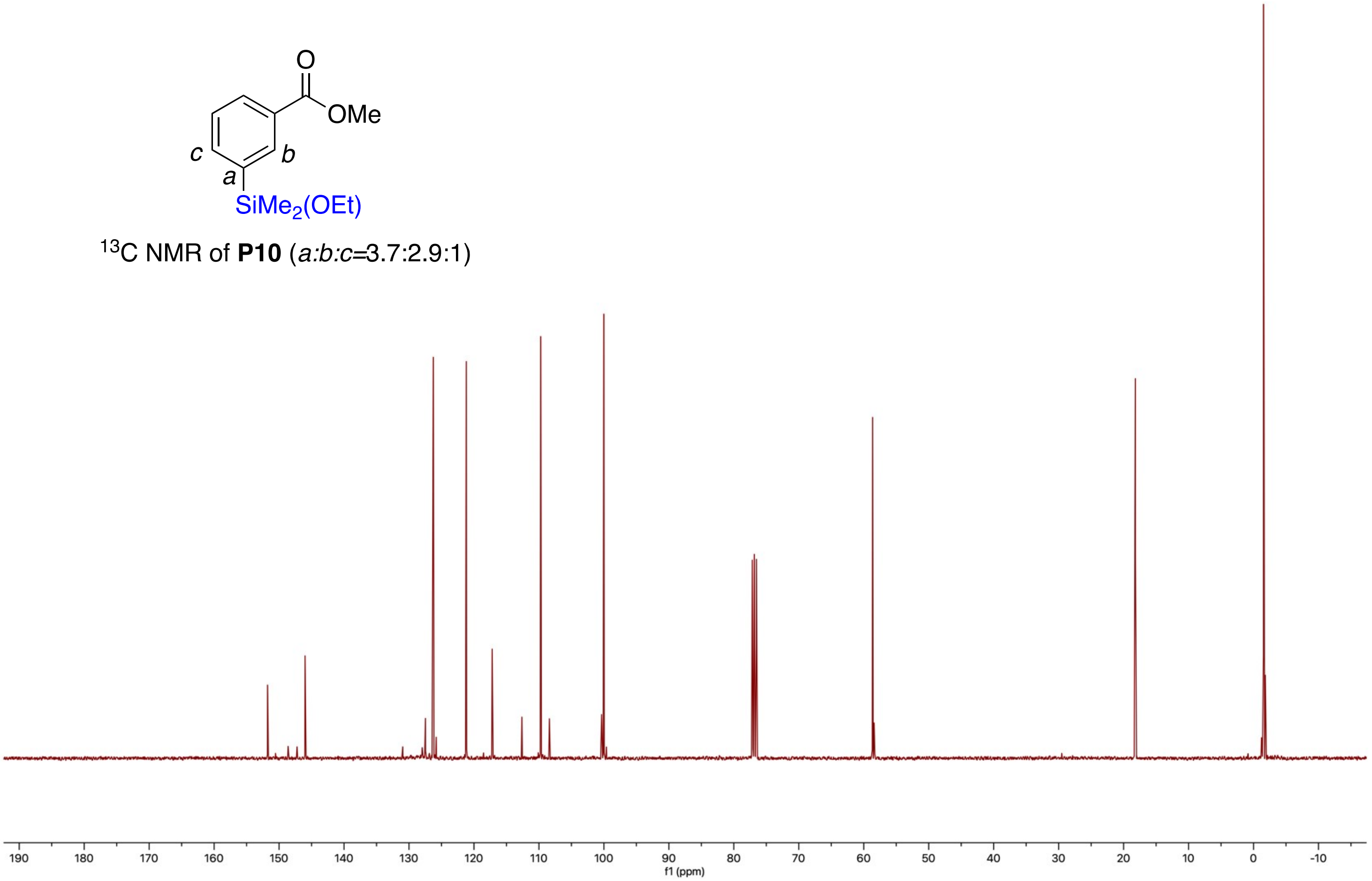

^{13}C NMR of **P10** (*a*:*b*:*c*=3.7:2.9:1)

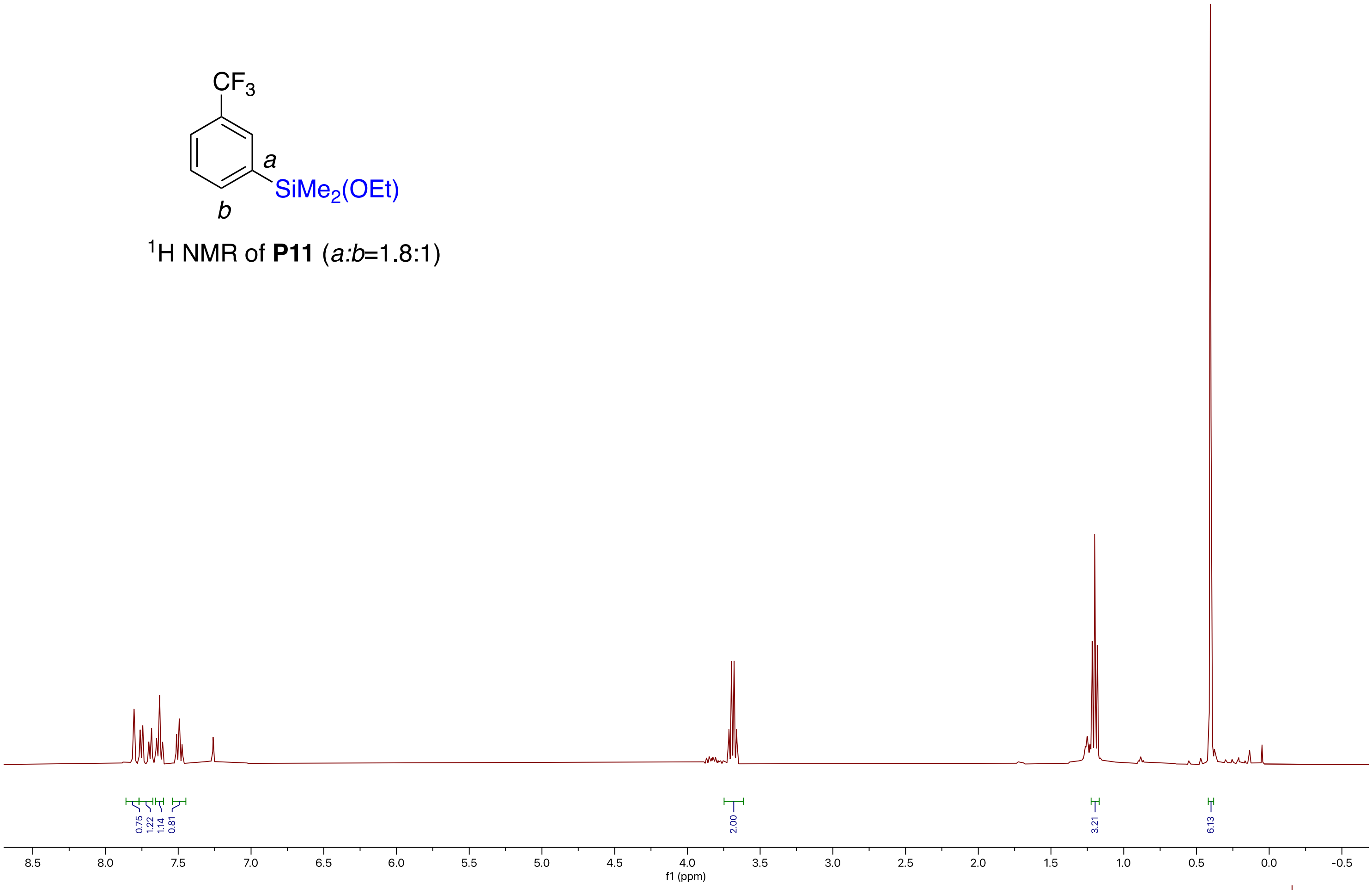

^{1}H NMR of **P11** (*a*:*b*=1.8:1)

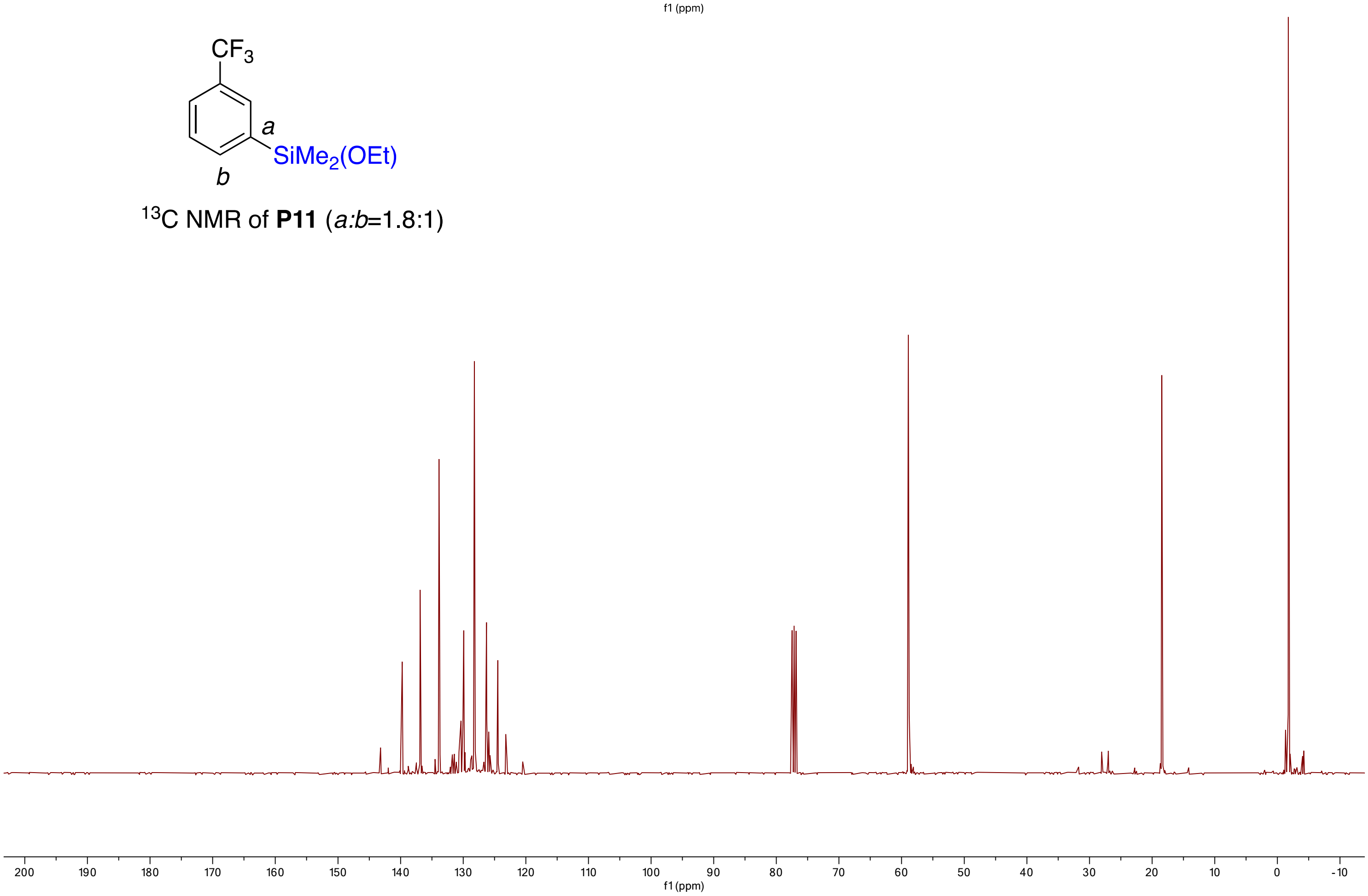

^{13}C NMR of **P11** (*a*:*b*=1.8:1)

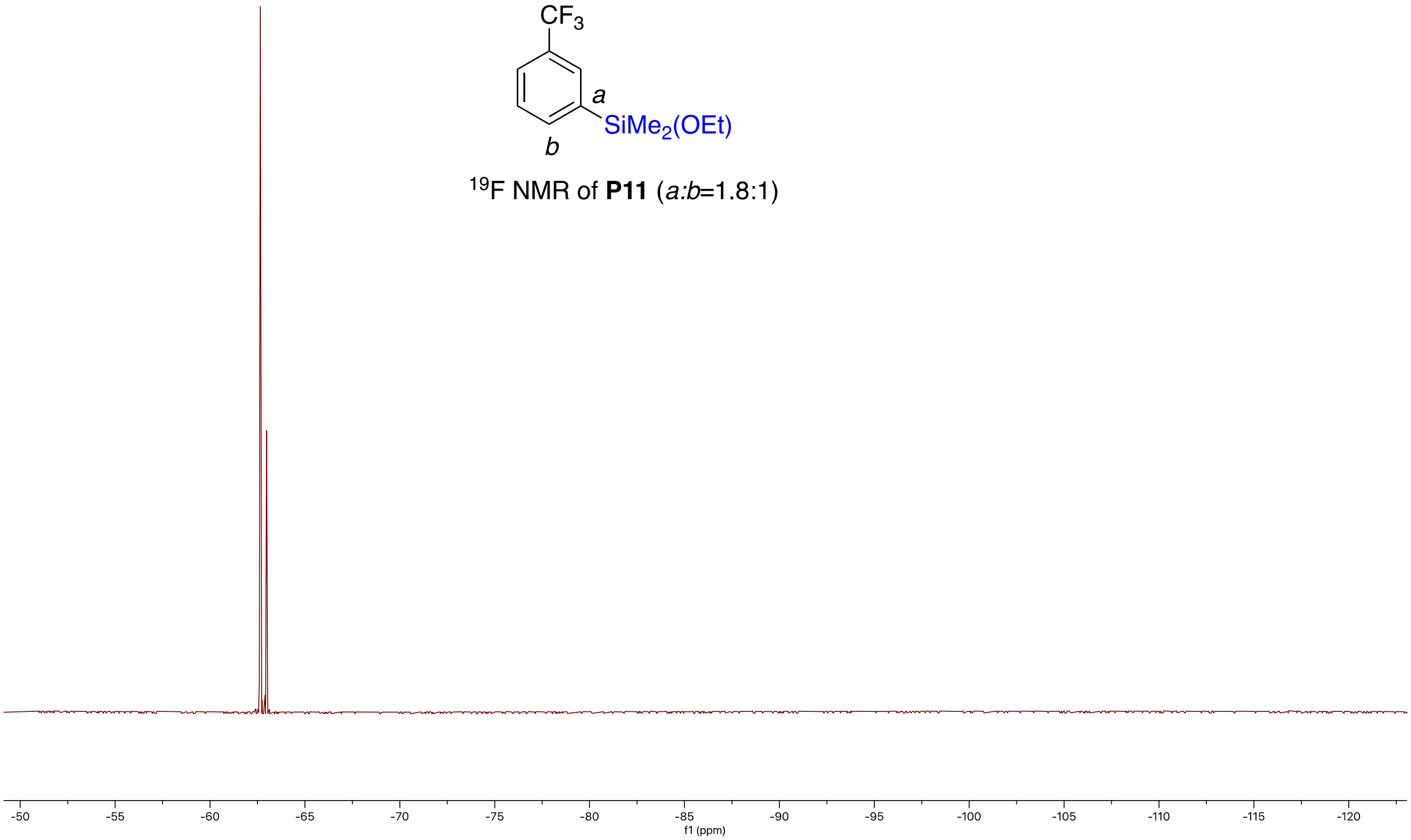

^{19}F NMR of **P11** (*a*:*b*=1.8:1)

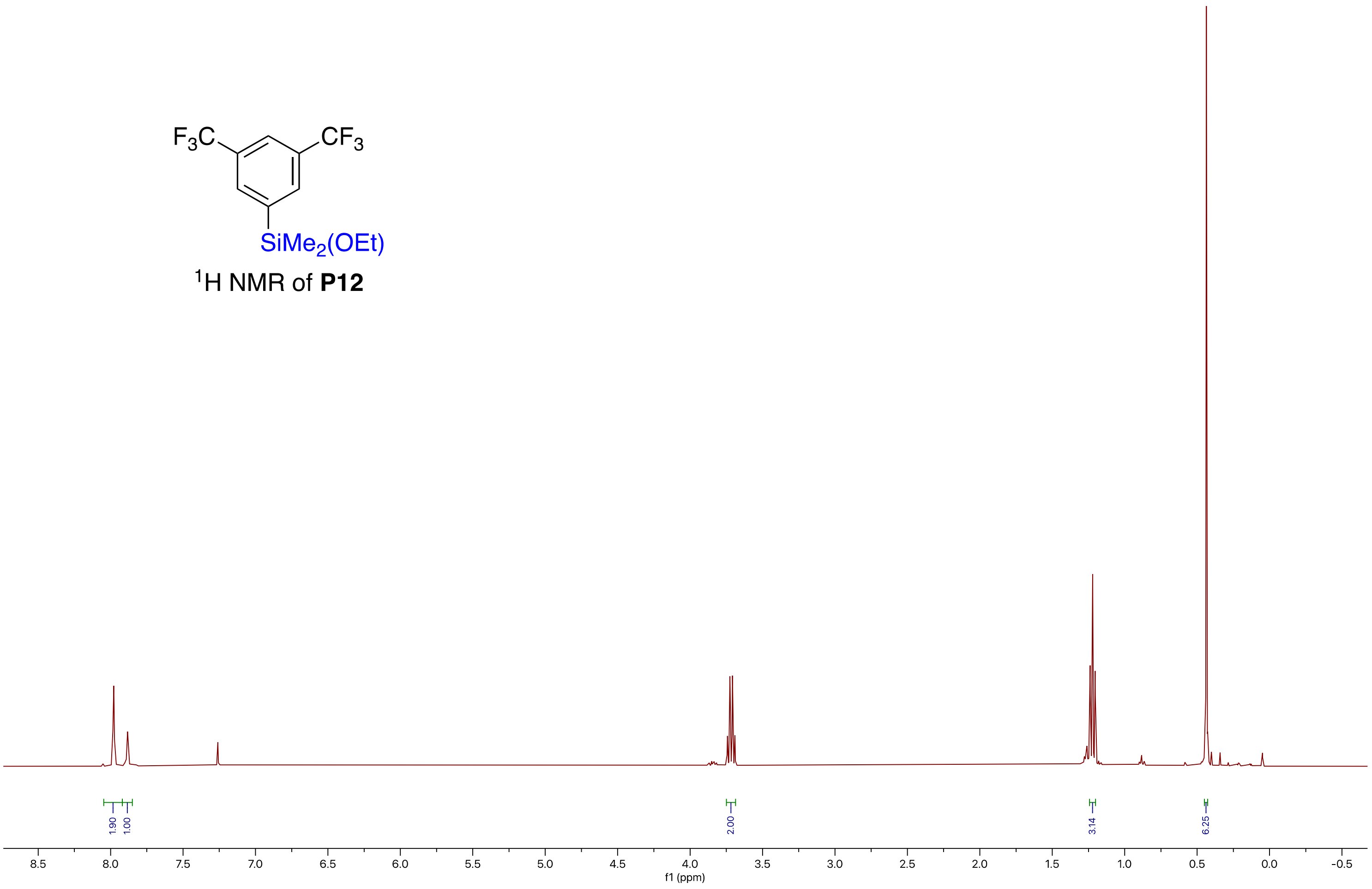

F_3C
CF_3
SiMe2(OEt)
1H NMR of P12
1.90
1.00
2.00
3.14
6.25
f1 (ppm)

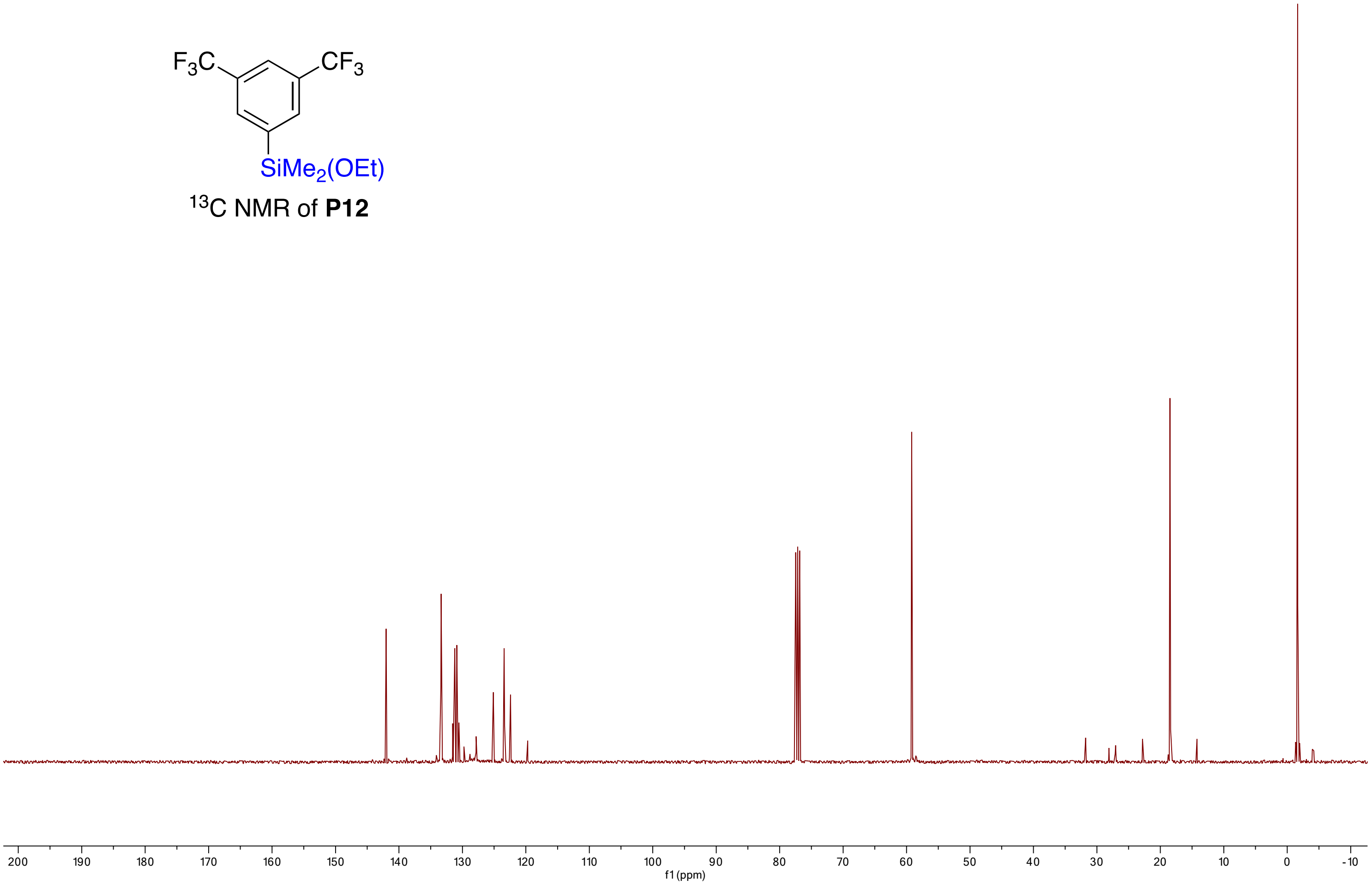

F_3C
CF_3
SiMe2(OEt)
13C NMR of P12
f1 (ppm)

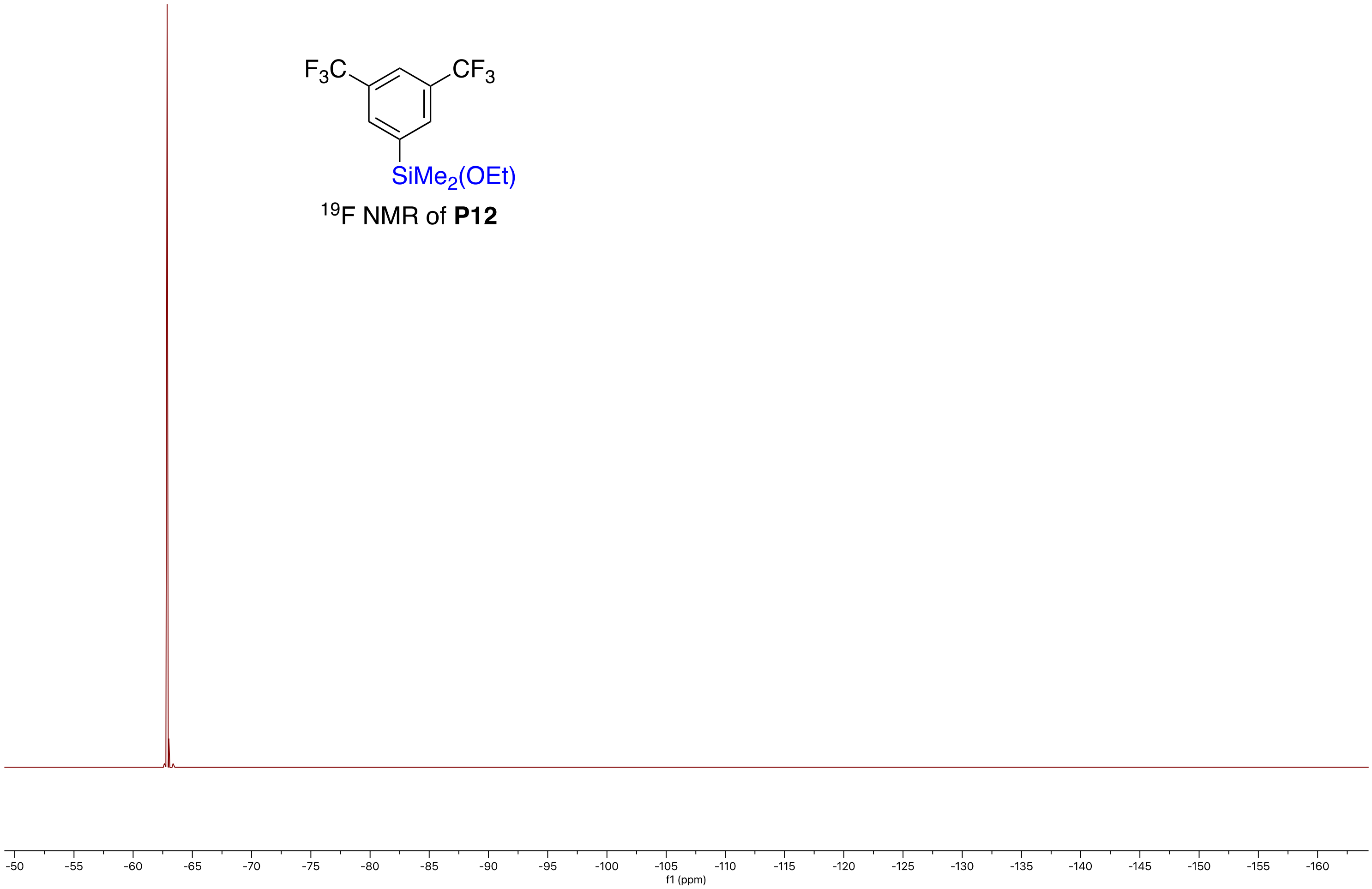
F3C
CF3
SiMe2(OEt)
19F NMR of P12
-50
-55
-60
-65
-70
-75
-80
-85
-90
-95
-100
-105
-110
-115
-120
-125
-130
-135
-140
-145
-150
-155
-160
f1 (ppm)

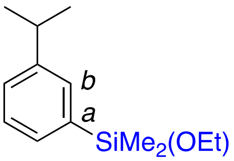

^{1}H NMR of **P13** (*a*:*b*=1.9:1)

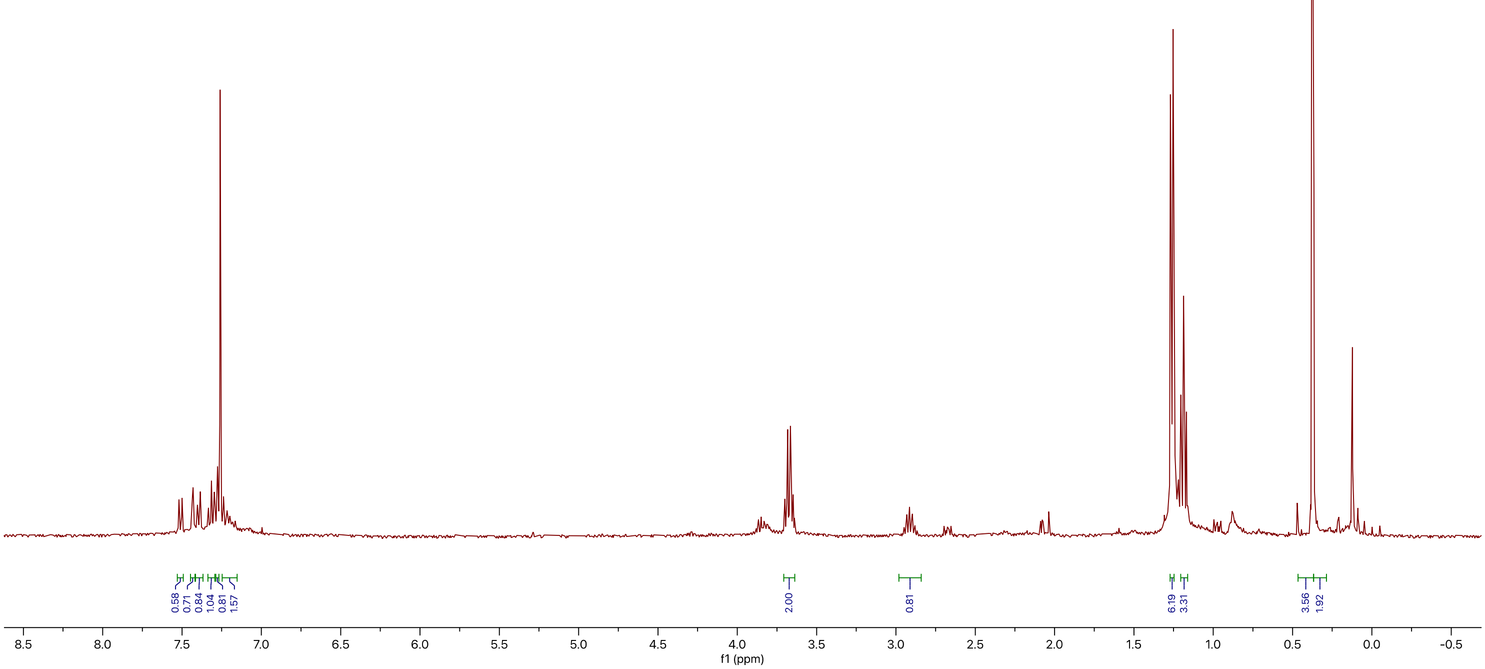

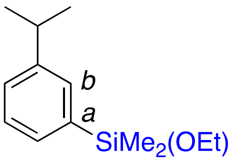

^{13}C NMR of **P13** (*a*:*b*=1.9:1)

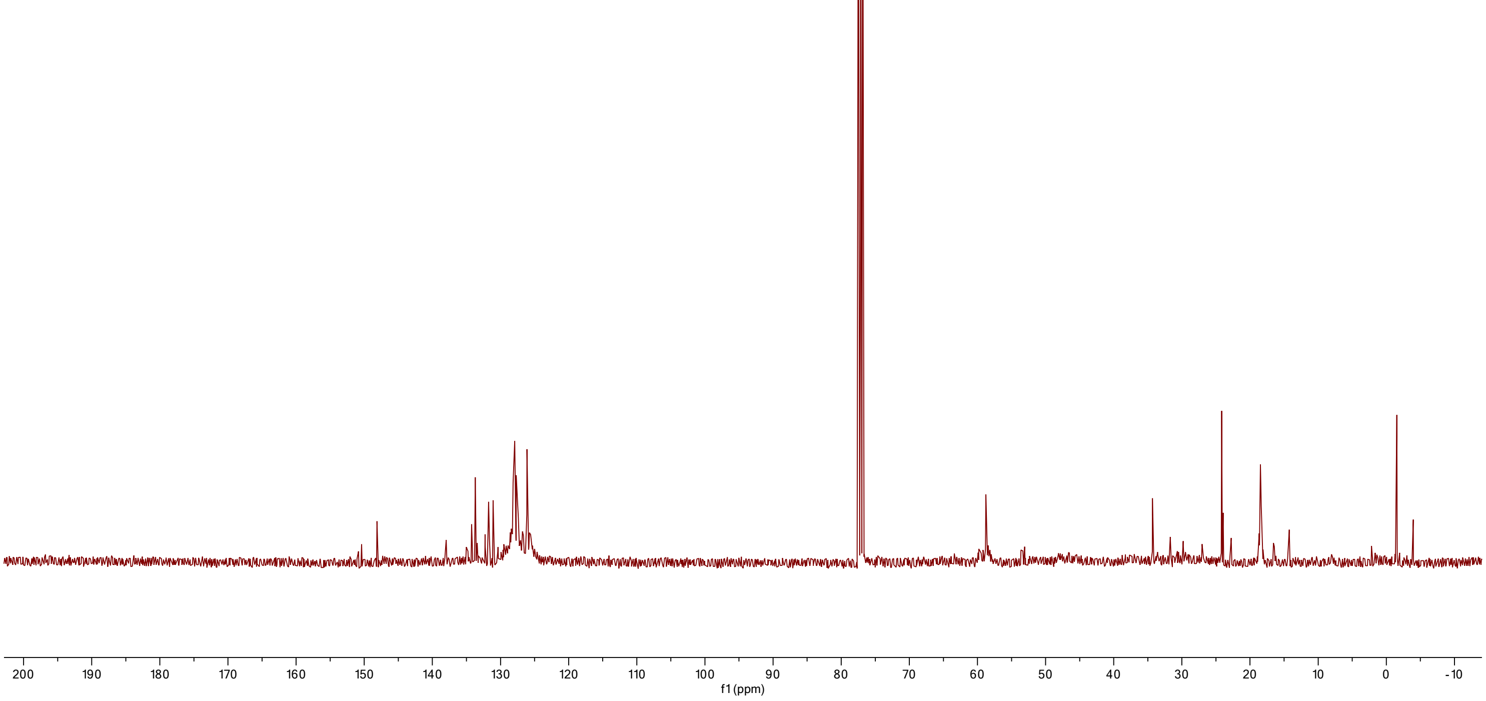

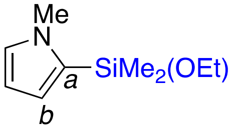

^{1}H NMR of **P15** (*a*:*b*=7.1:1)

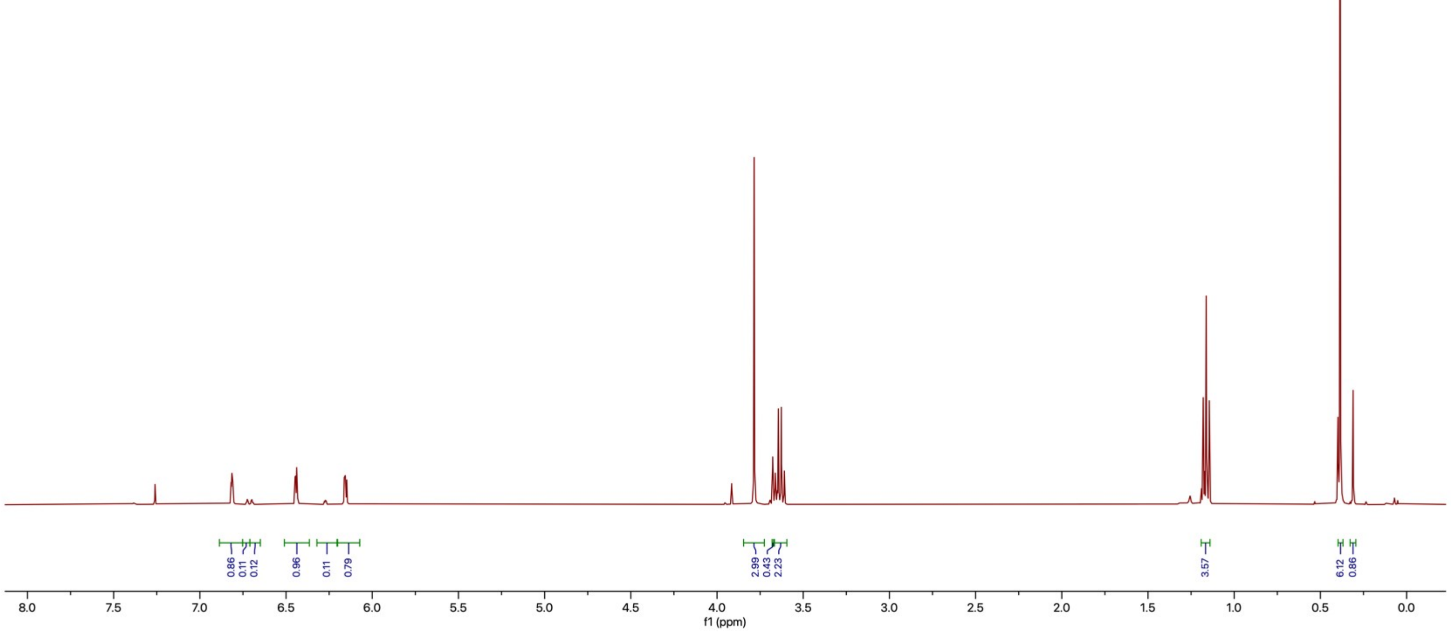

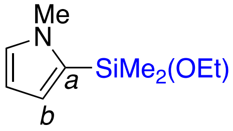

^{13}C NMR of **P15** (*a*:*b*=7.1:1)

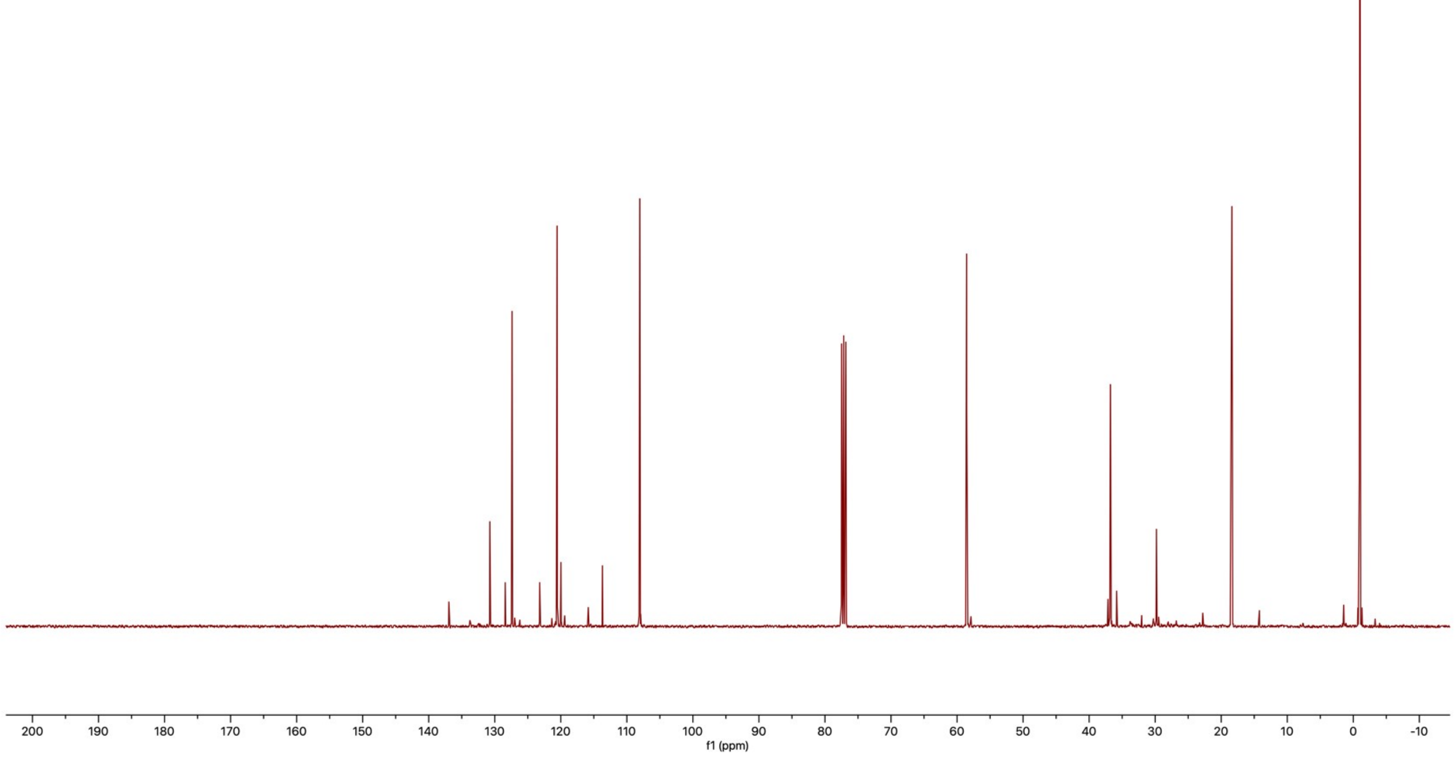

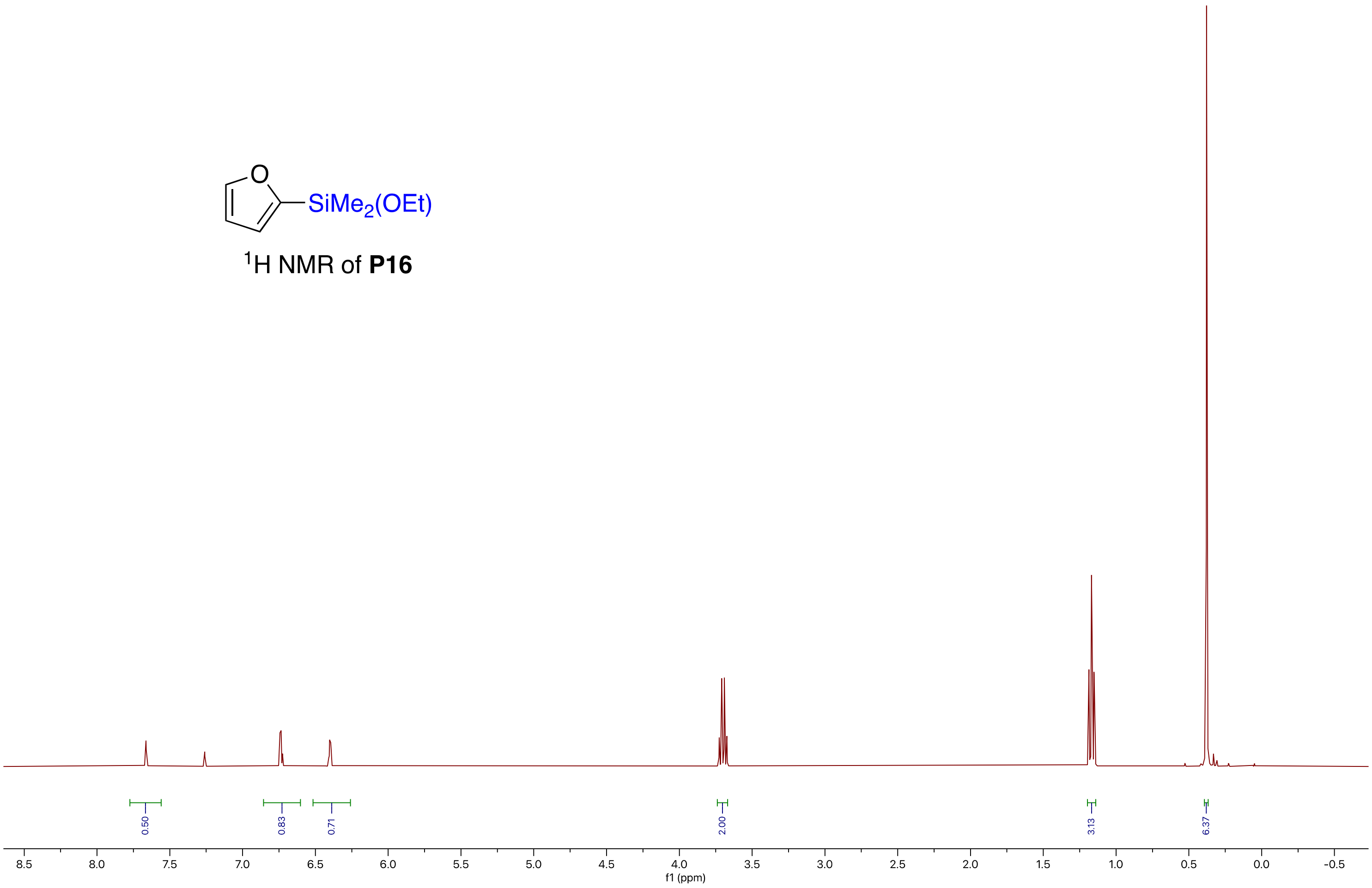
SiMe2(OEt)
1H NMR of P16
0.50
0.83
0.71
2.00
3.13
6.37
f1 (ppm)

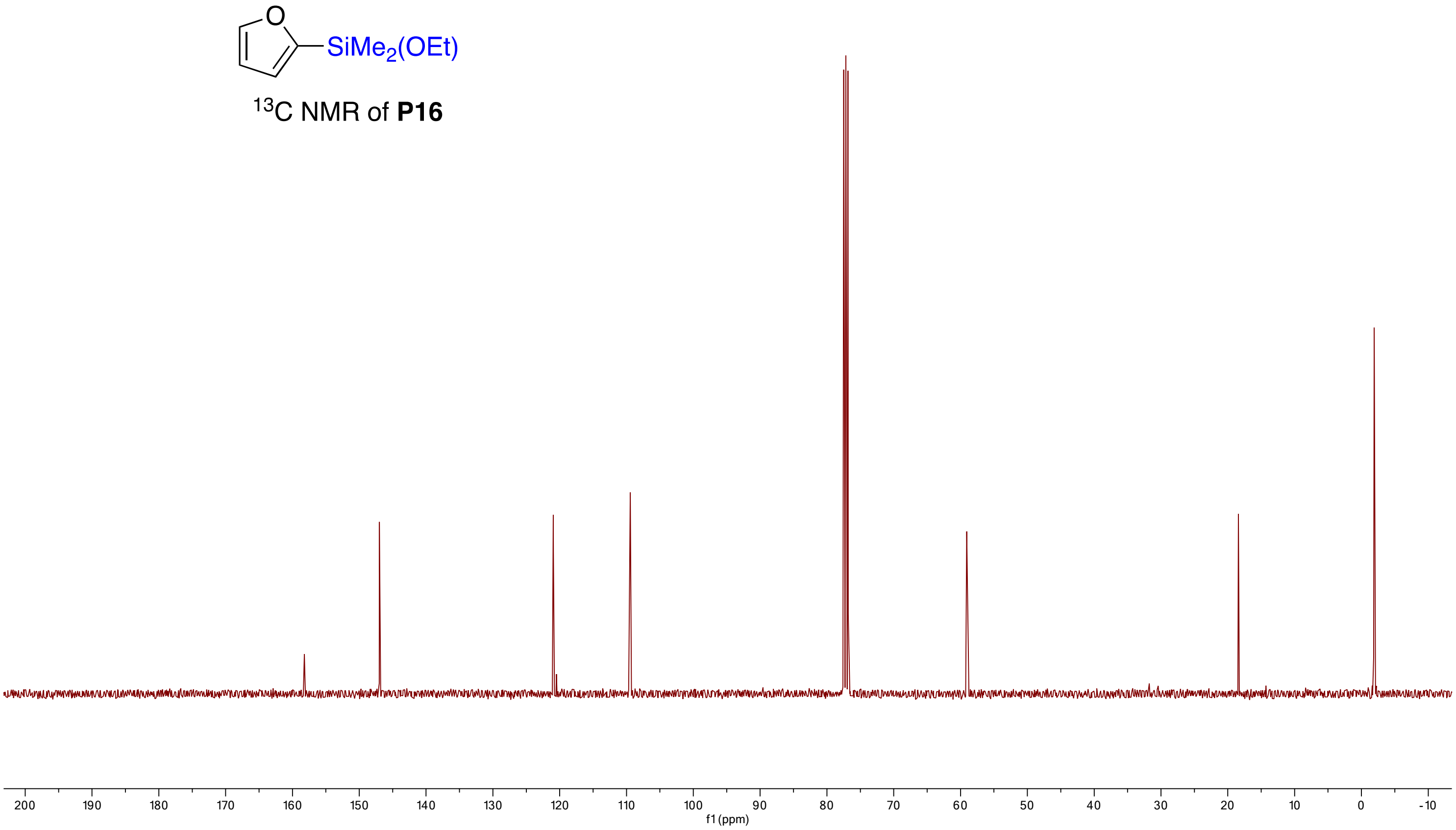
SiMe2(OEt)
13C NMR of P16
f1 (ppm)

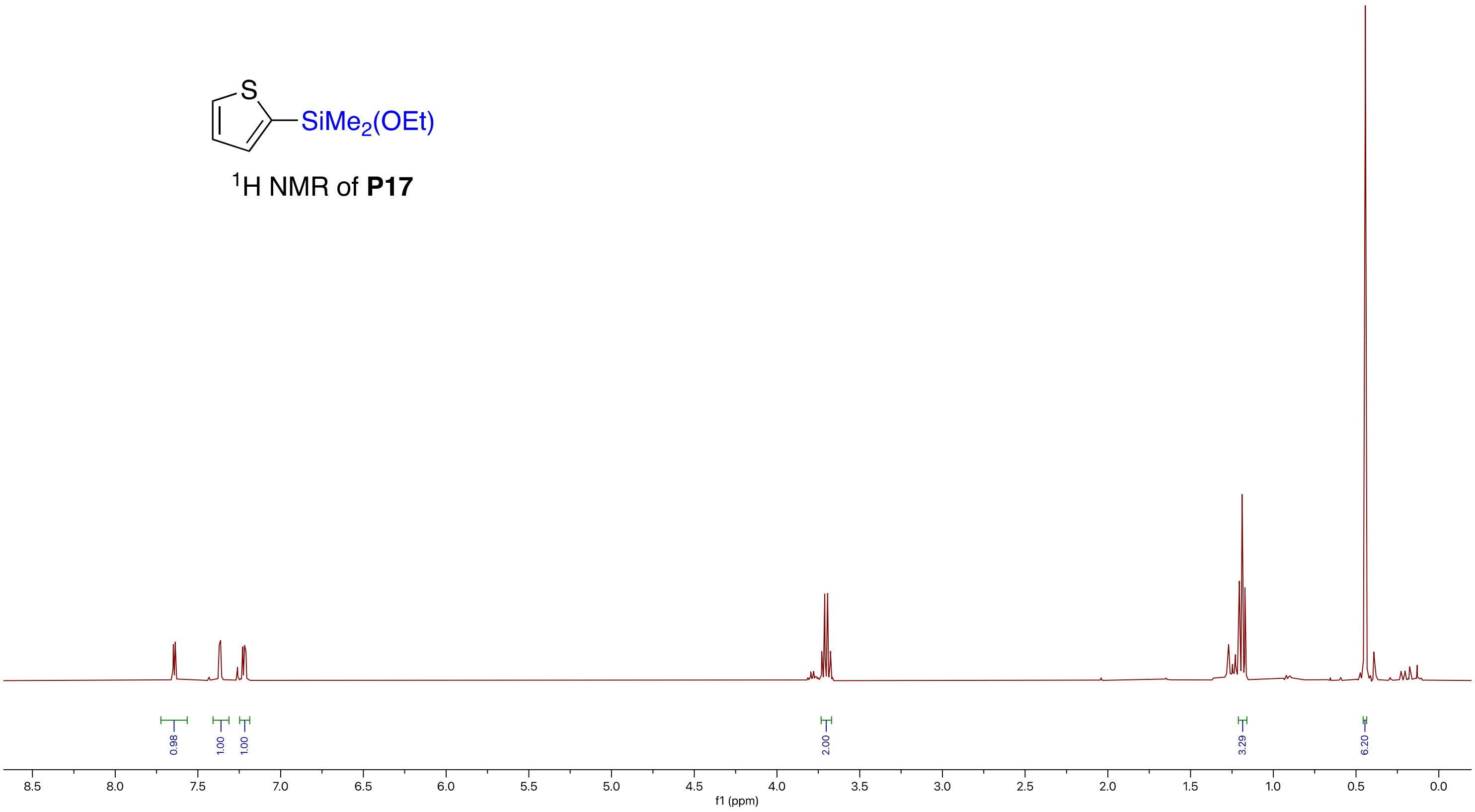
S
SiMe2(OEt)
1H NMR of P17
0.98
1.00
1.00
2.00
3.29
6.20
8.5
8.0
7.5
7.0
6.5
6.0
5.5
5.0
4.5
4.0
3.5
3.0
2.5
2.0
1.5
1.0
0.5
0.0
f1 (ppm)

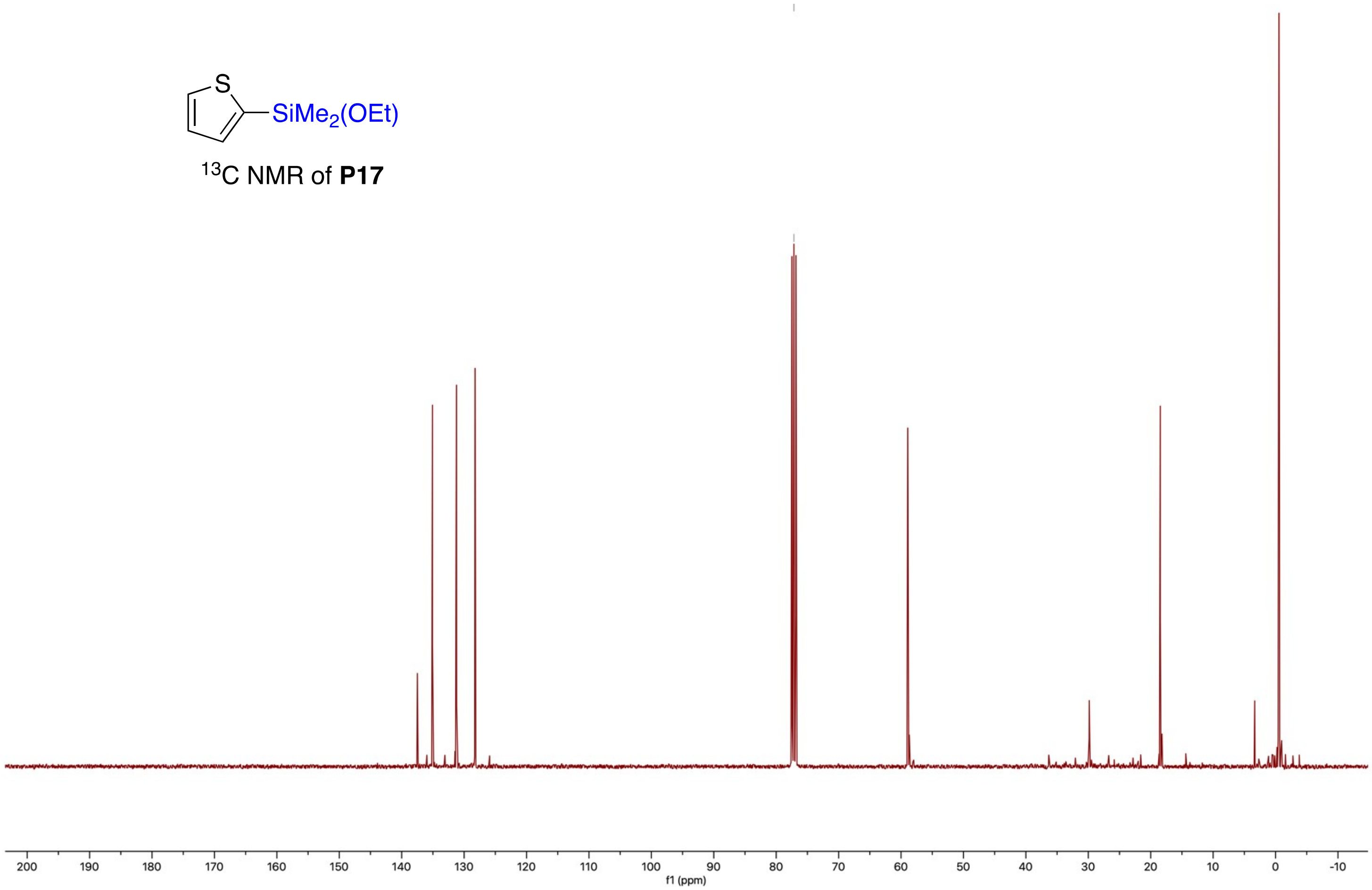
S
SiMe2(OEt)
13C NMR of P17
200
190
180
170
160
150
140
130
120
110
100
90
80
70
60
50
40
30
20
10
0
-10
f1 (ppm)

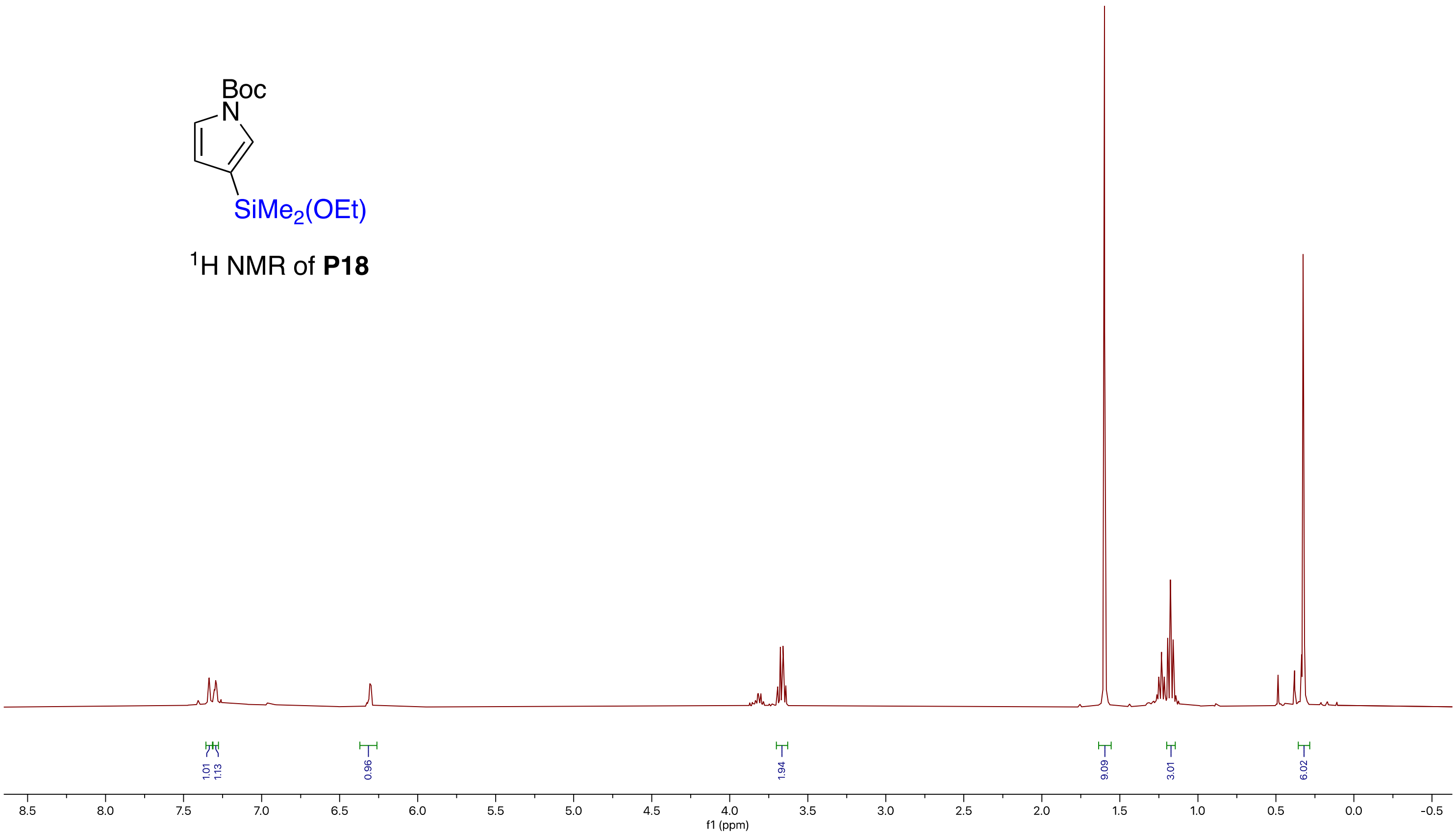
Boc
N
SiMe2(OEt)
1H NMR of P18
1.01
1.13
0.96
1.94
9.09
3.01
6.02
8.5 8.0 7.5 7.0 6.5 6.0 5.5 5.0 4.5 4.0 3.5 3.0 2.5 2.0 1.5 1.0 0.5 0.0 -0.5
f1 (ppm)

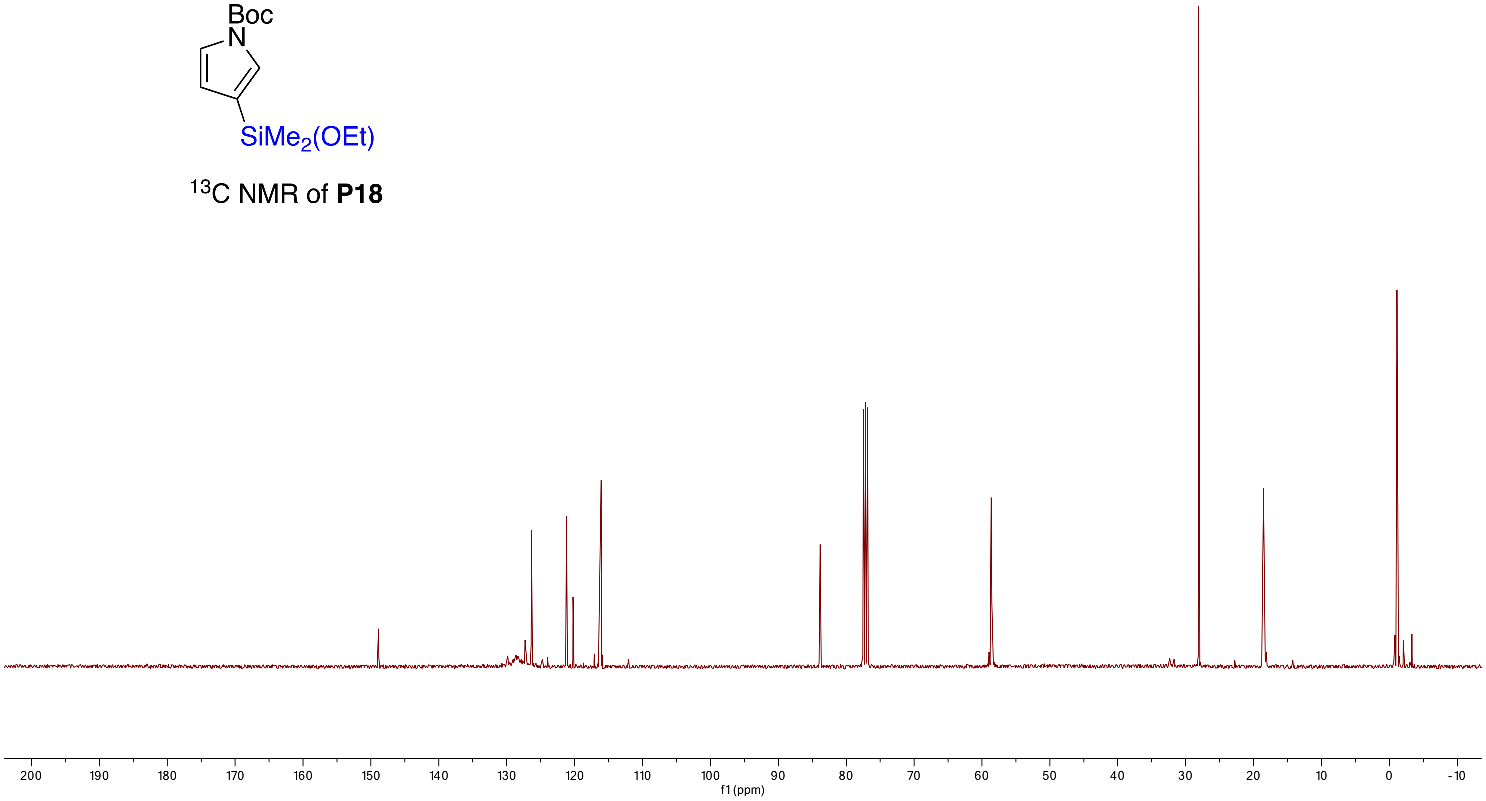
Boc
N
SiMe2(OEt)
13C NMR of P18
200 190 180 170 160 150 140 130 120 110 100 90 80 70 60 50 40 30 20 10 0 -10
f1 (ppm)

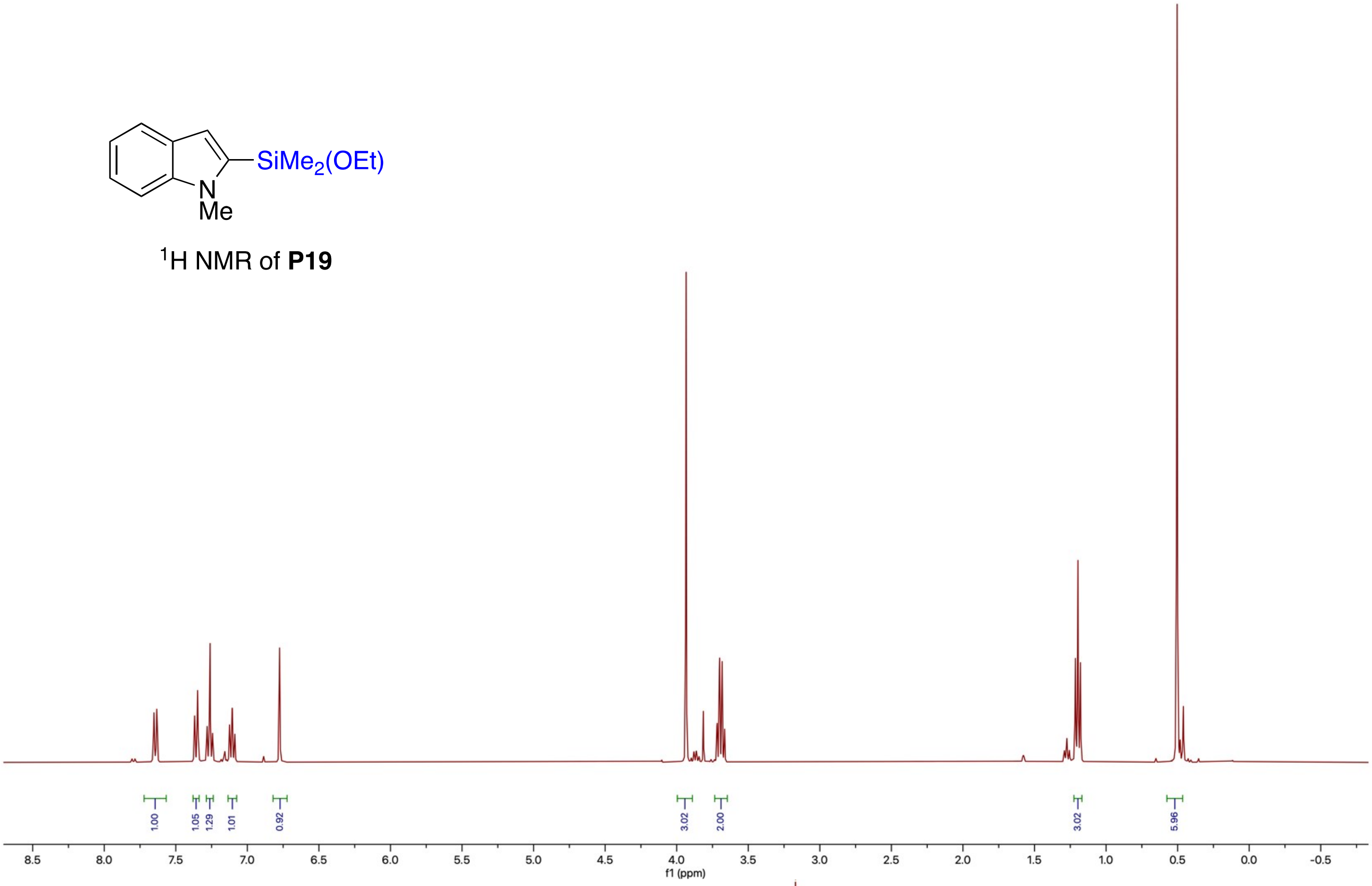

^{1}H NMR of **P19**

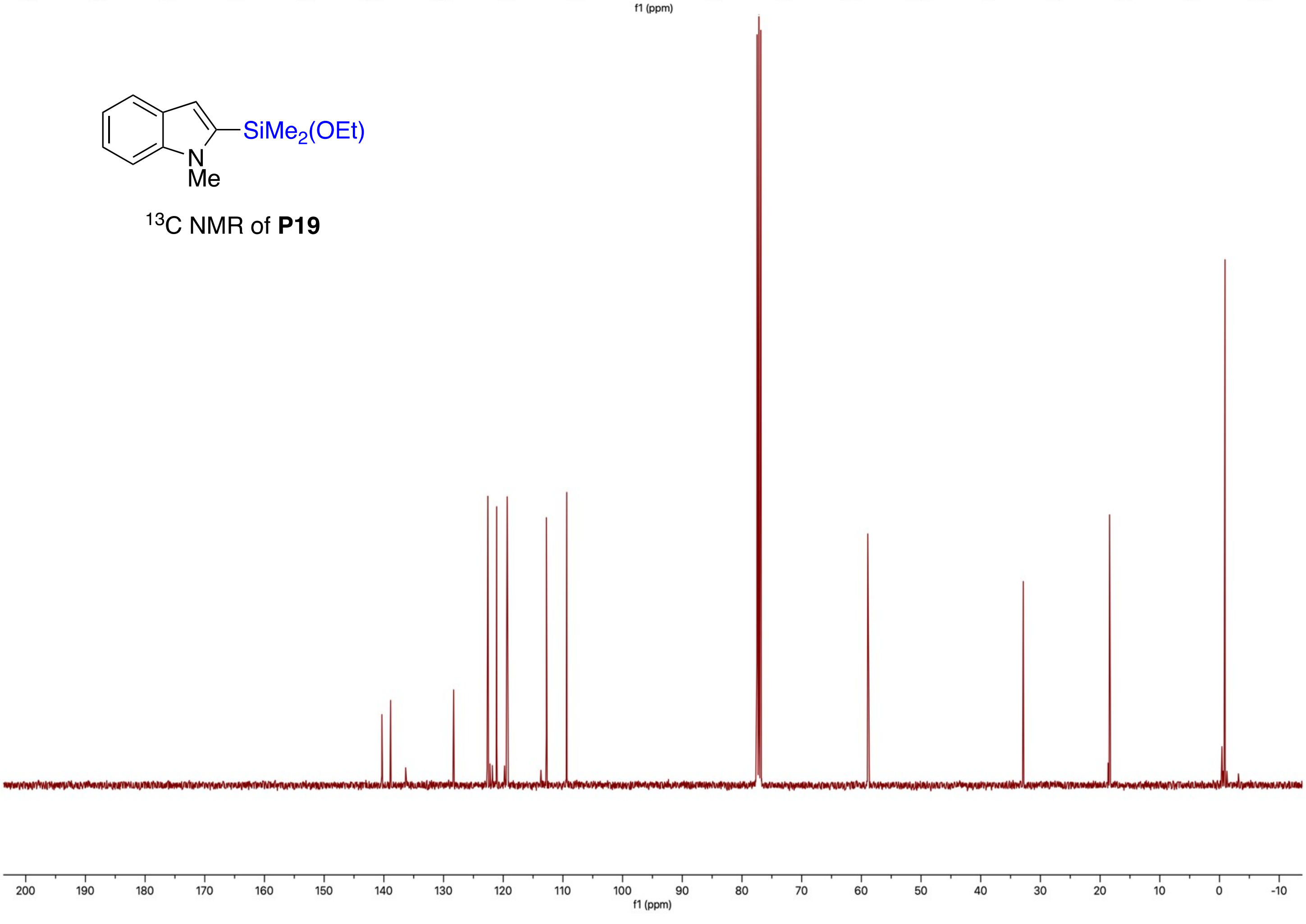

^{13}C NMR of **P19**

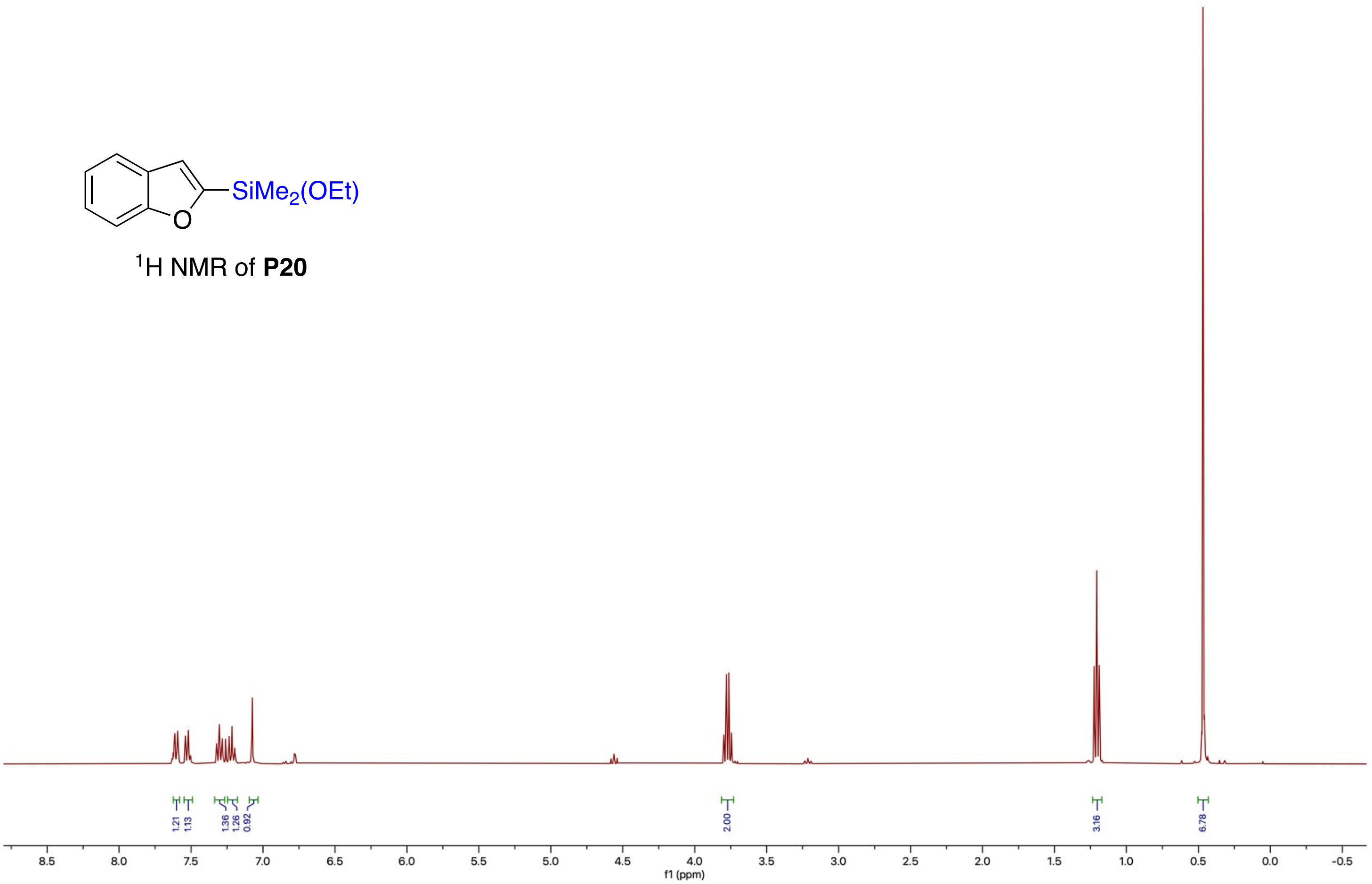
SiMe2(OEt)
O
1H NMR of P20
1.21
1.13
1.36
1.26
0.92
2.00
3.16
6.78
8.5 8.0 7.5 7.0 6.5 6.0 5.5 5.0 4.5 4.0 3.5 3.0 2.5 2.0 1.5 1.0 0.5 0.0 -0.5
f1 (ppm)

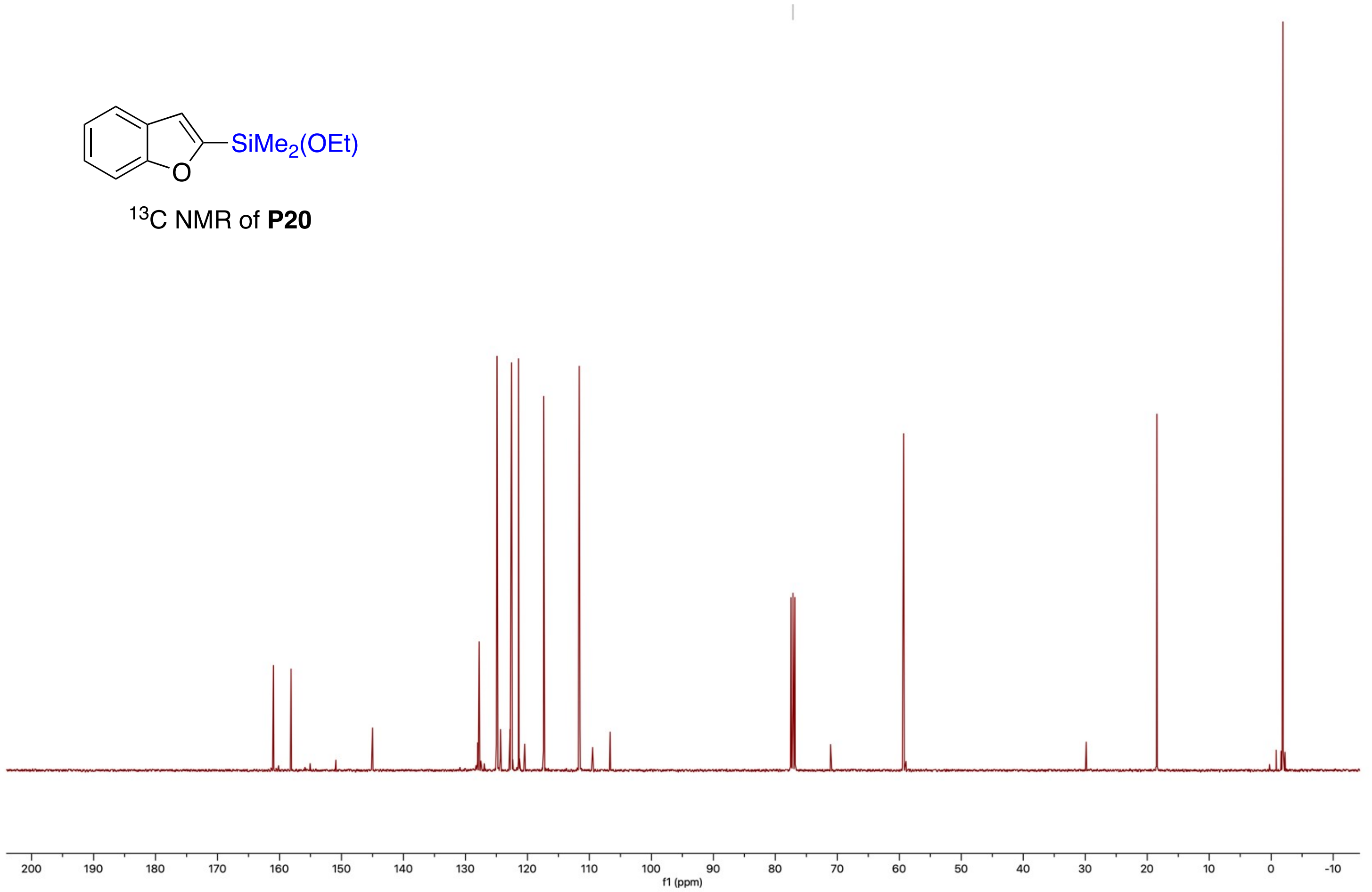
SiMe2(OEt)
O
13C NMR of P20
200 190 180 170 160 150 140 130 120 110 100 90 80 70 60 50 40 30 20 10 0 -10
f1 (ppm)

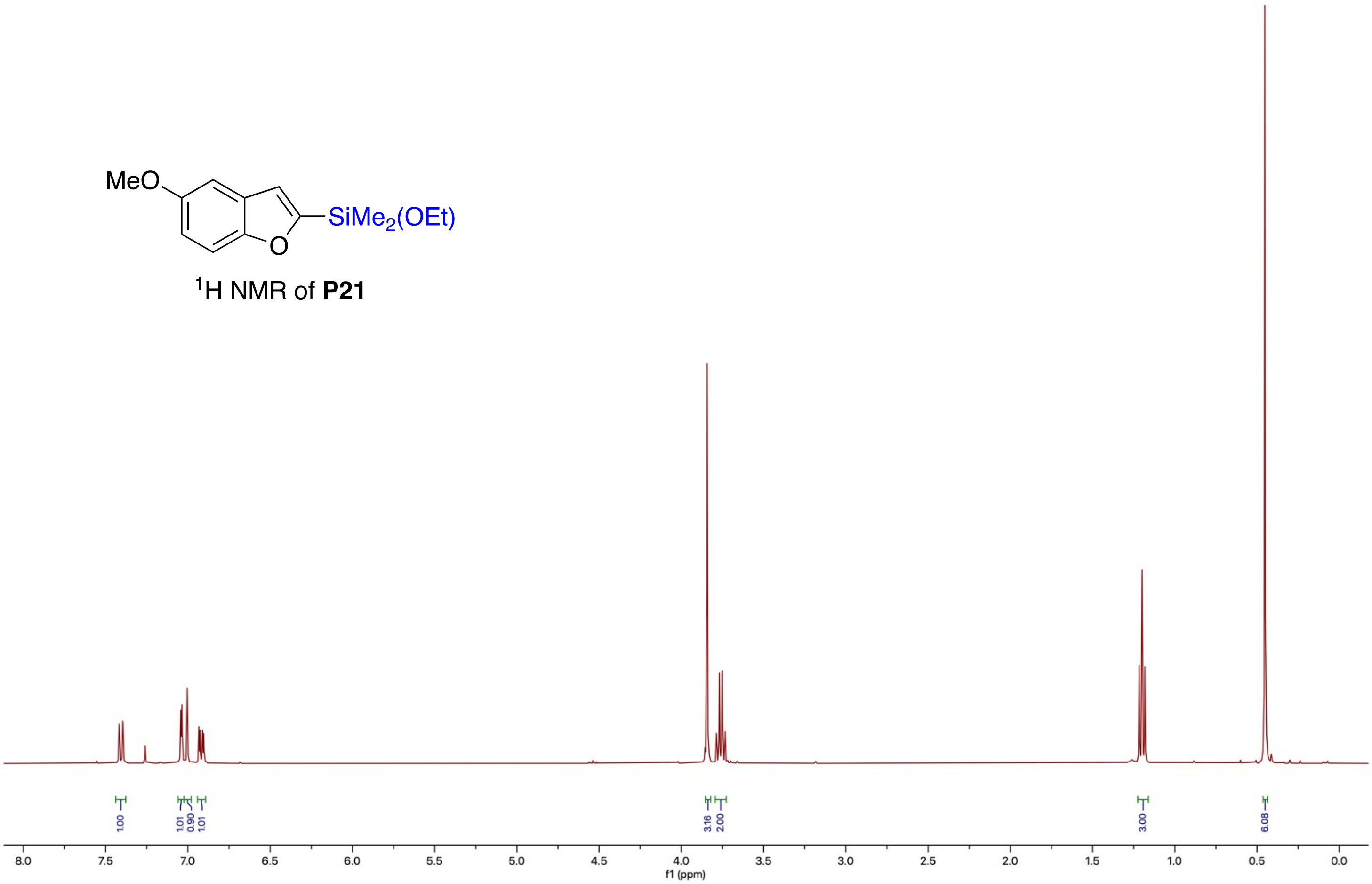
MeO
SiMe2(OEt)
O
1H NMR of P21
1.00
1.01
0.90
1.01
3.16
2.00
3.00
6.08
8.0 7.5 7.0 6.5 6.0 5.5 5.0 4.5 4.0 3.5 3.0 2.5 2.0 1.5 1.0 0.5 0.0
f1 (ppm)

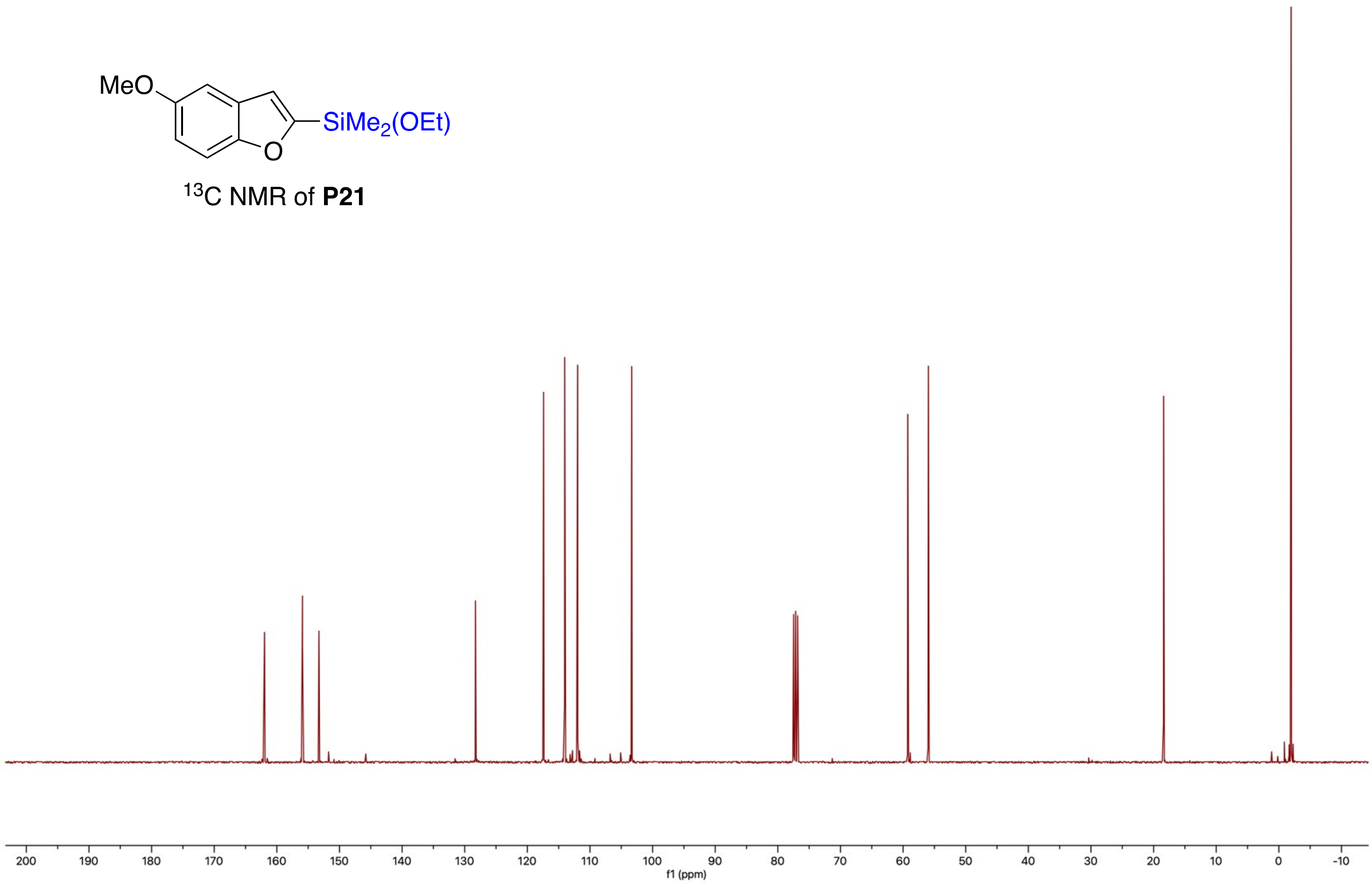
MeO
SiMe2(OEt)
O
13C NMR of P21
200 190 180 170 160 150 140 130 120 110 100 90 80 70 60 50 40 30 20 10 0 -10
f1 (ppm)

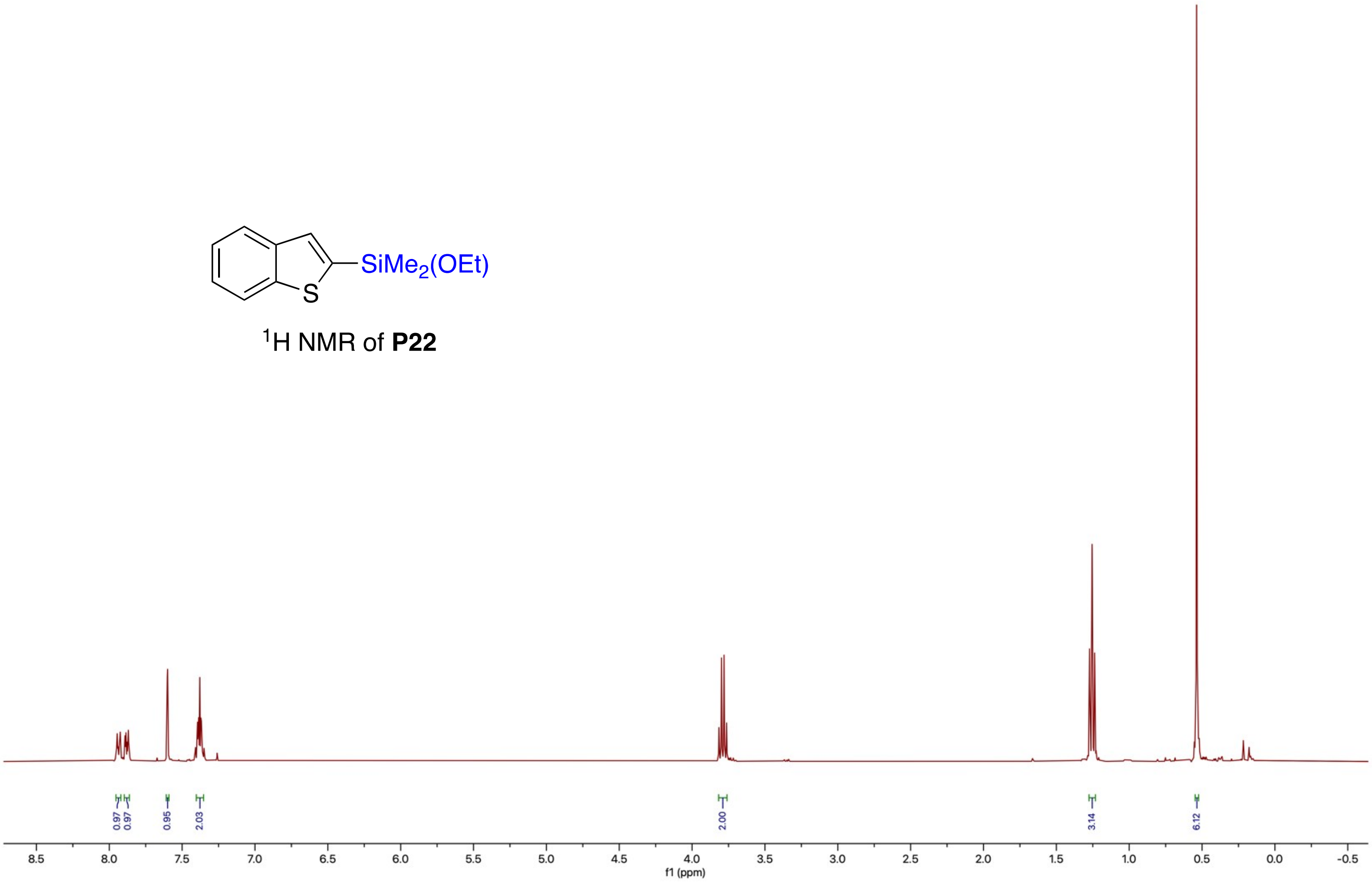
SiMe2(OEt)
S
1H NMR of P22
0.97
0.97
0.95
2.03
2.00
3.14
6.12
f1 (ppm)

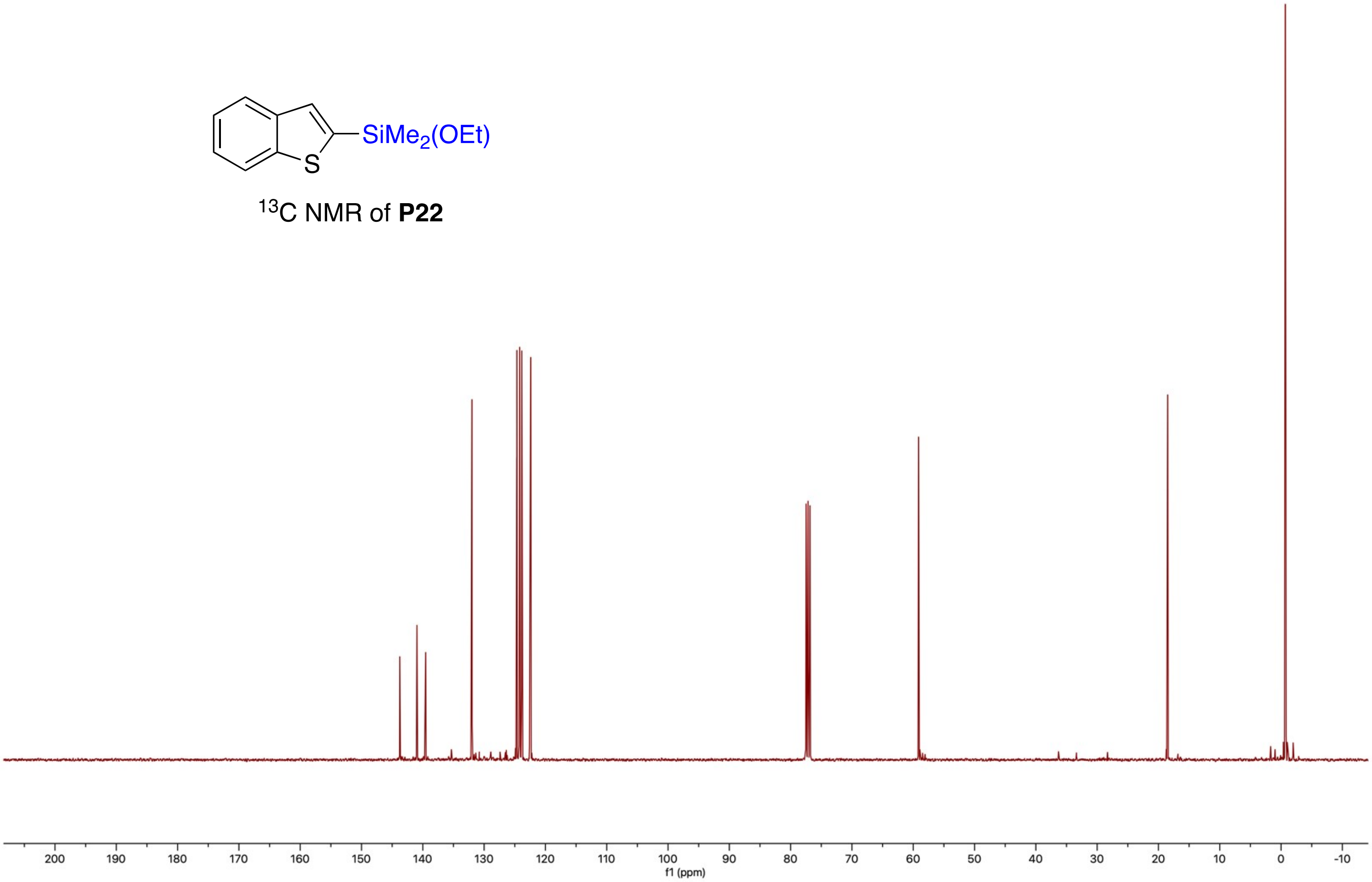
SiMe2(OEt)
S
13C NMR of P22
f1 (ppm)

5.2 Double C–H Silylation Products

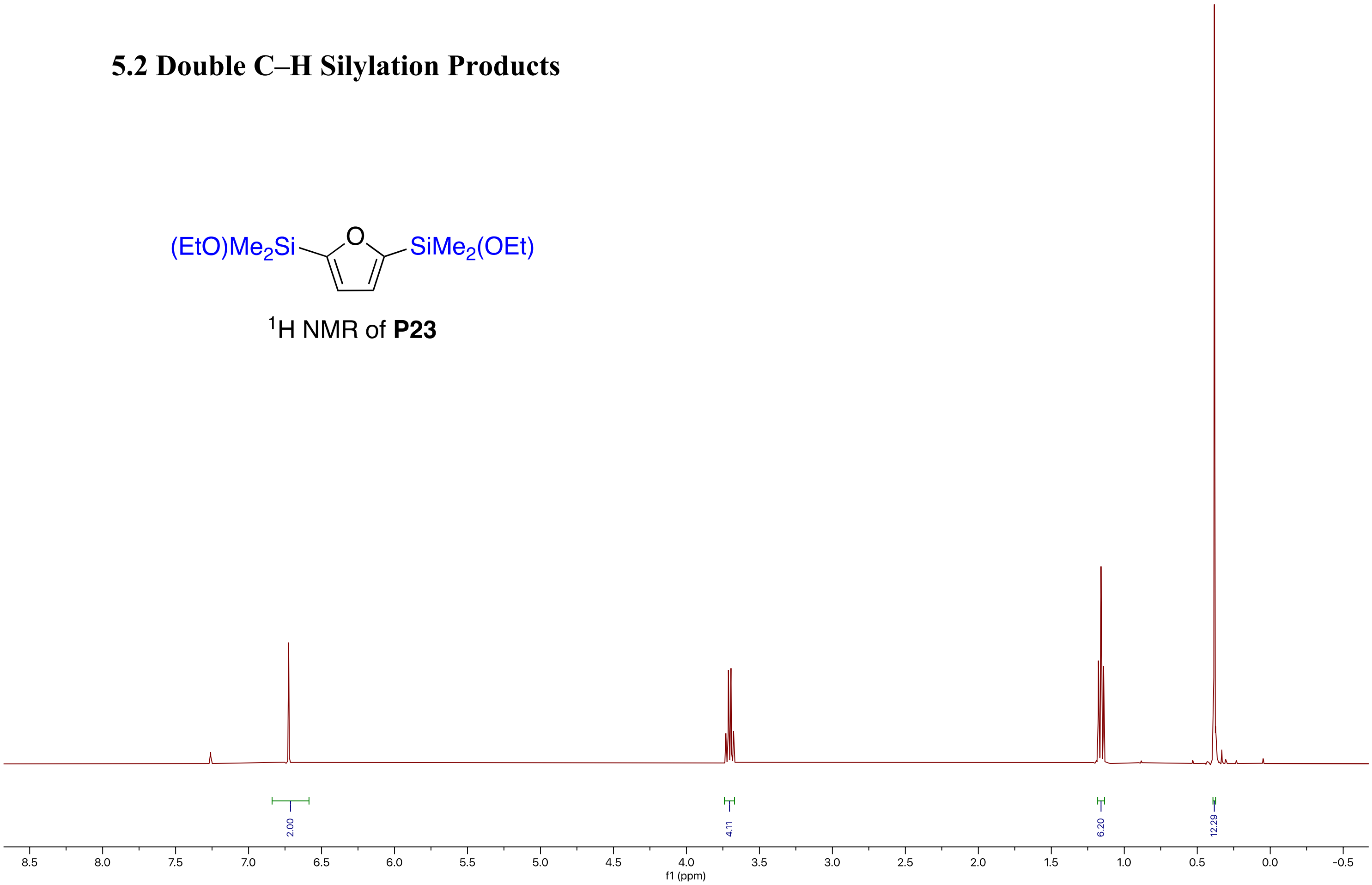

^{1}H NMR of **P23**

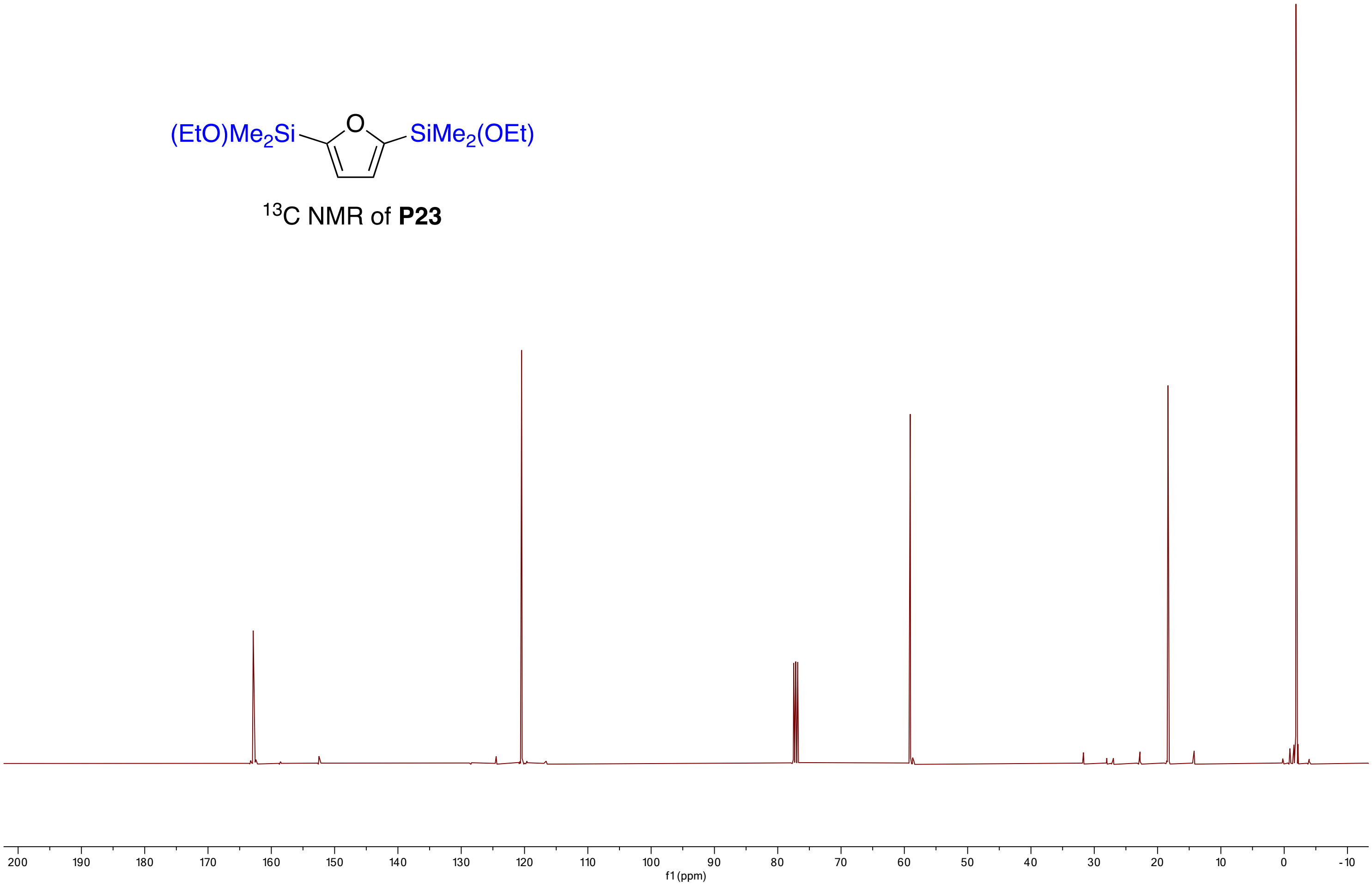

^{13}C NMR of **P23**

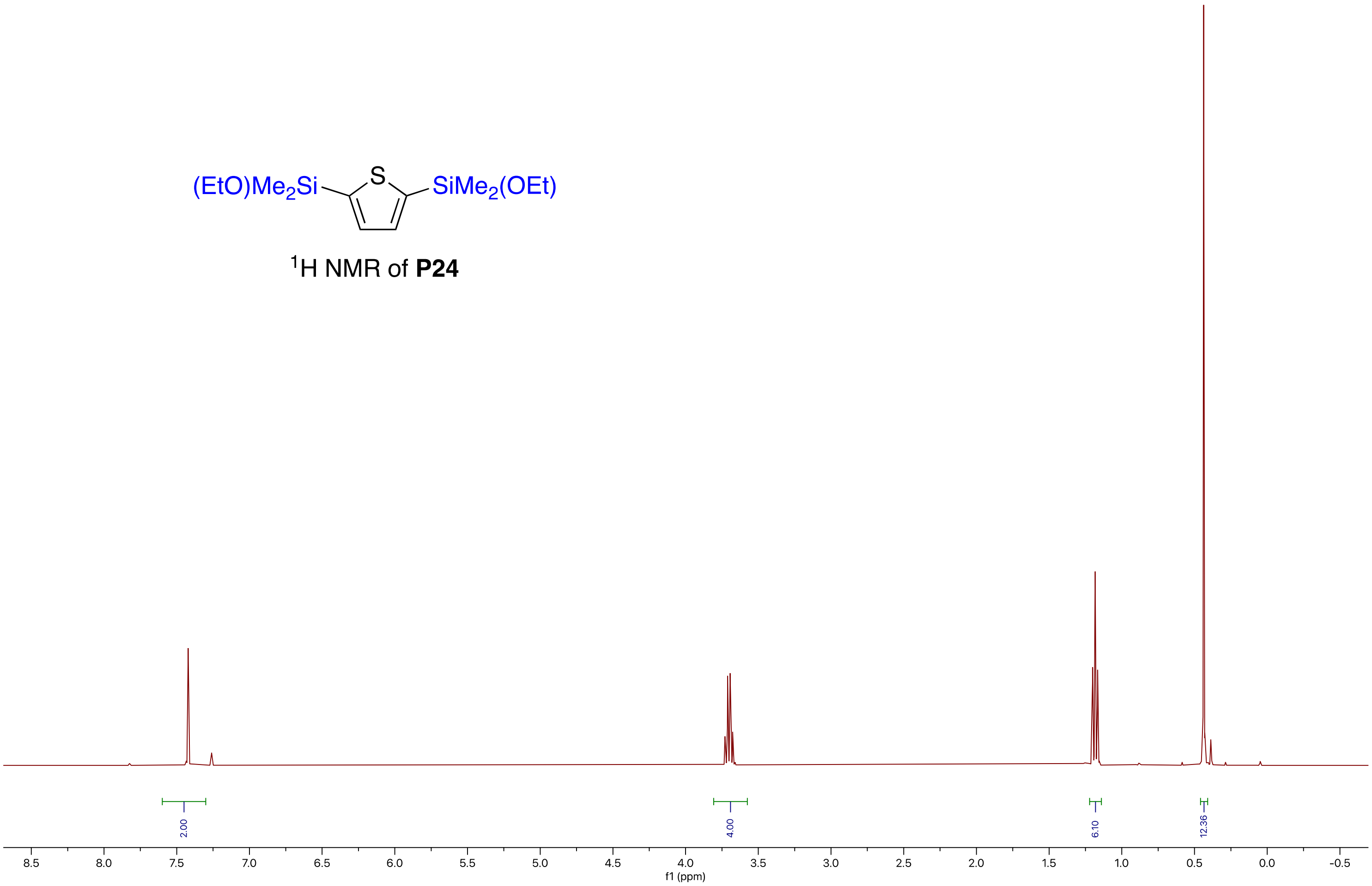
(EtO)Me2Si
S
SiMe2(OEt)
1H NMR of P24
2.00
4.00
6.10
12.36
8.5 8.0 7.5 7.0 6.5 6.0 5.5 5.0 4.5 4.0 3.5 3.0 2.5 2.0 1.5 1.0 0.5 0.0 -0.5
f1 (ppm)

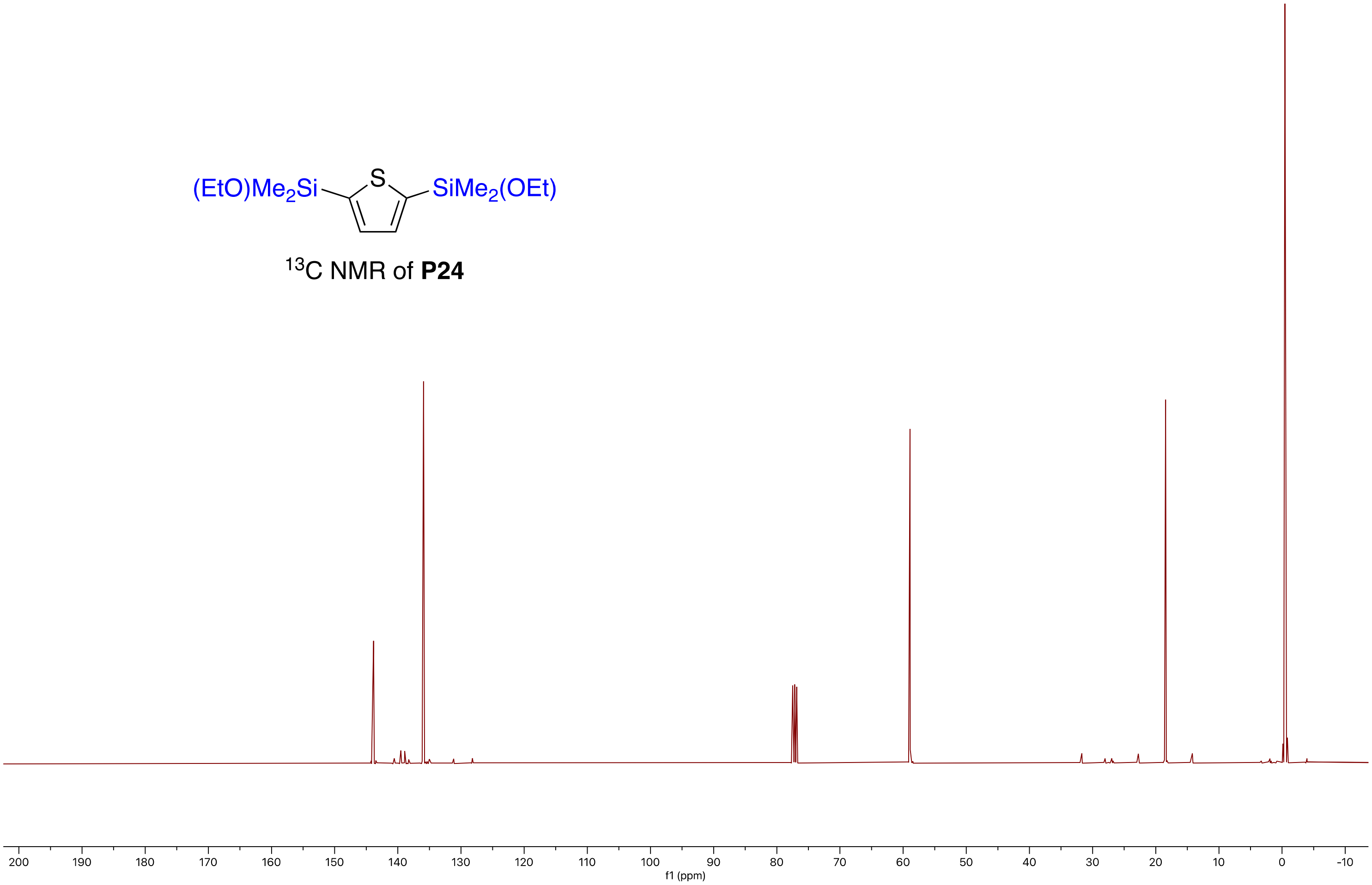
(EtO)Me2Si
S
SiMe2(OEt)
13C NMR of P24
200 190 180 170 160 150 140 130 120 110 100 90 80 70 60 50 40 30 20 10 0 -10
f1 (ppm)

5.3 Siloxane Products

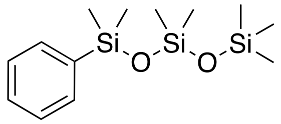

^{1}H NMR of **P1b**

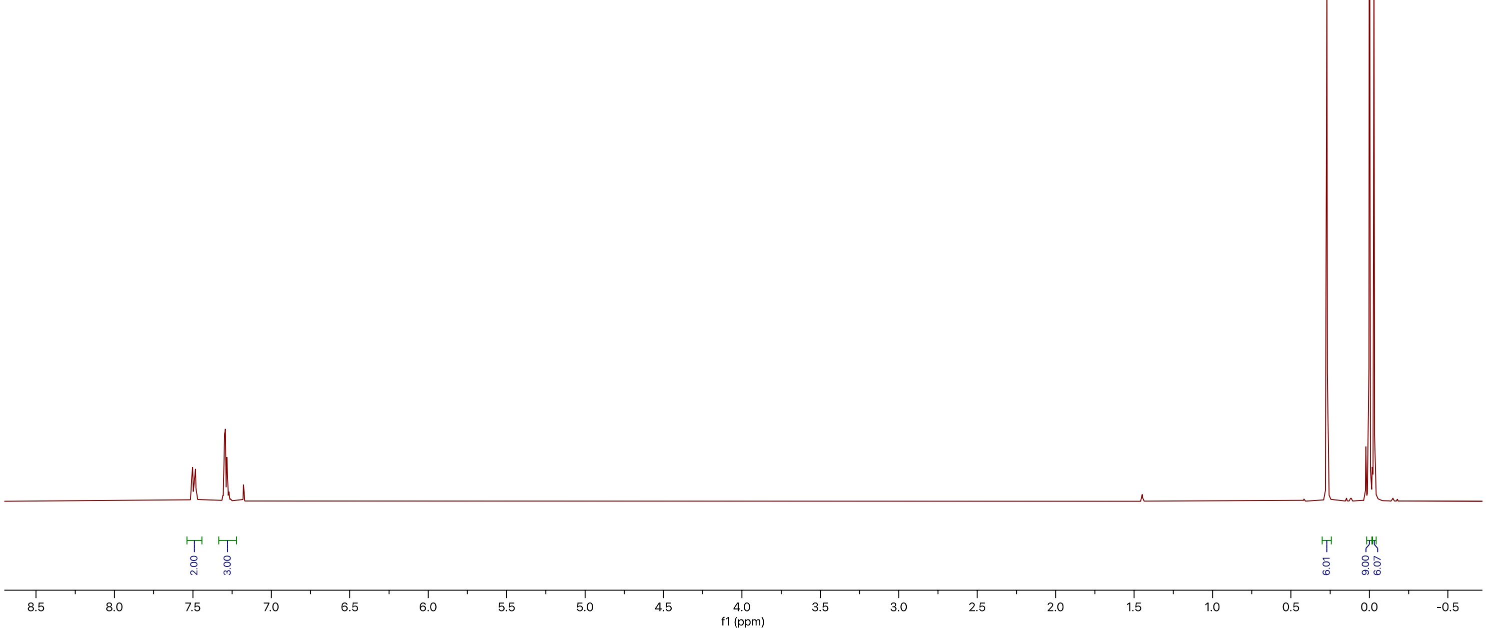

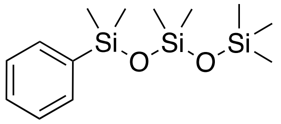

^{13}C NMR of **P1b**

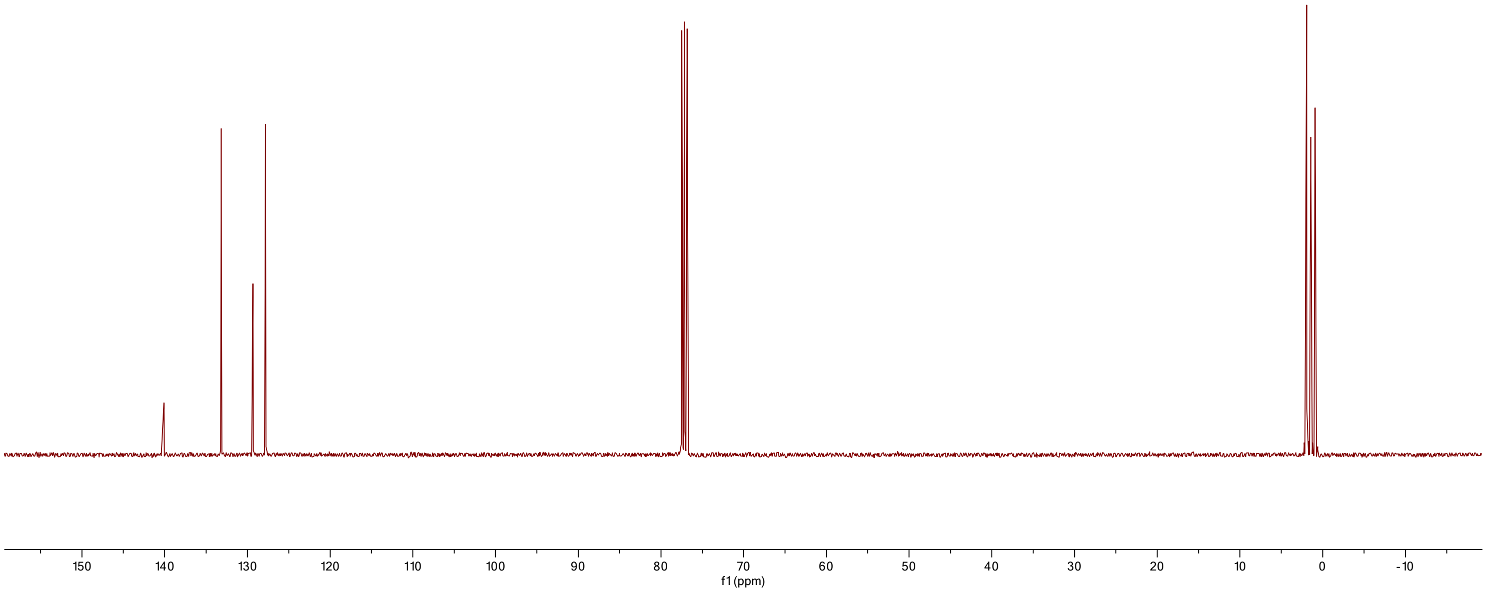

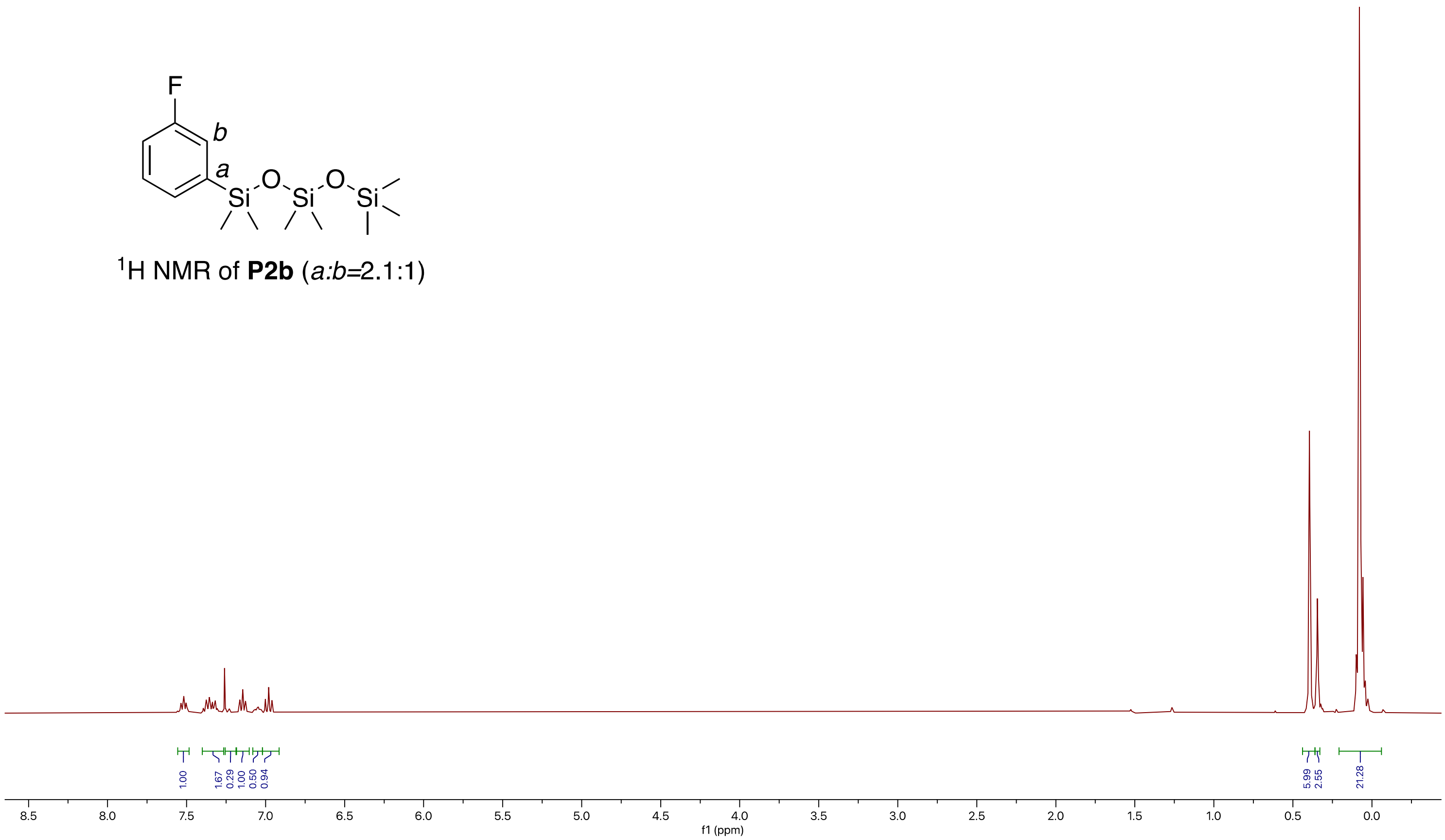

^{1}H NMR of **P2b** (*a*:*b*=2.1:1)

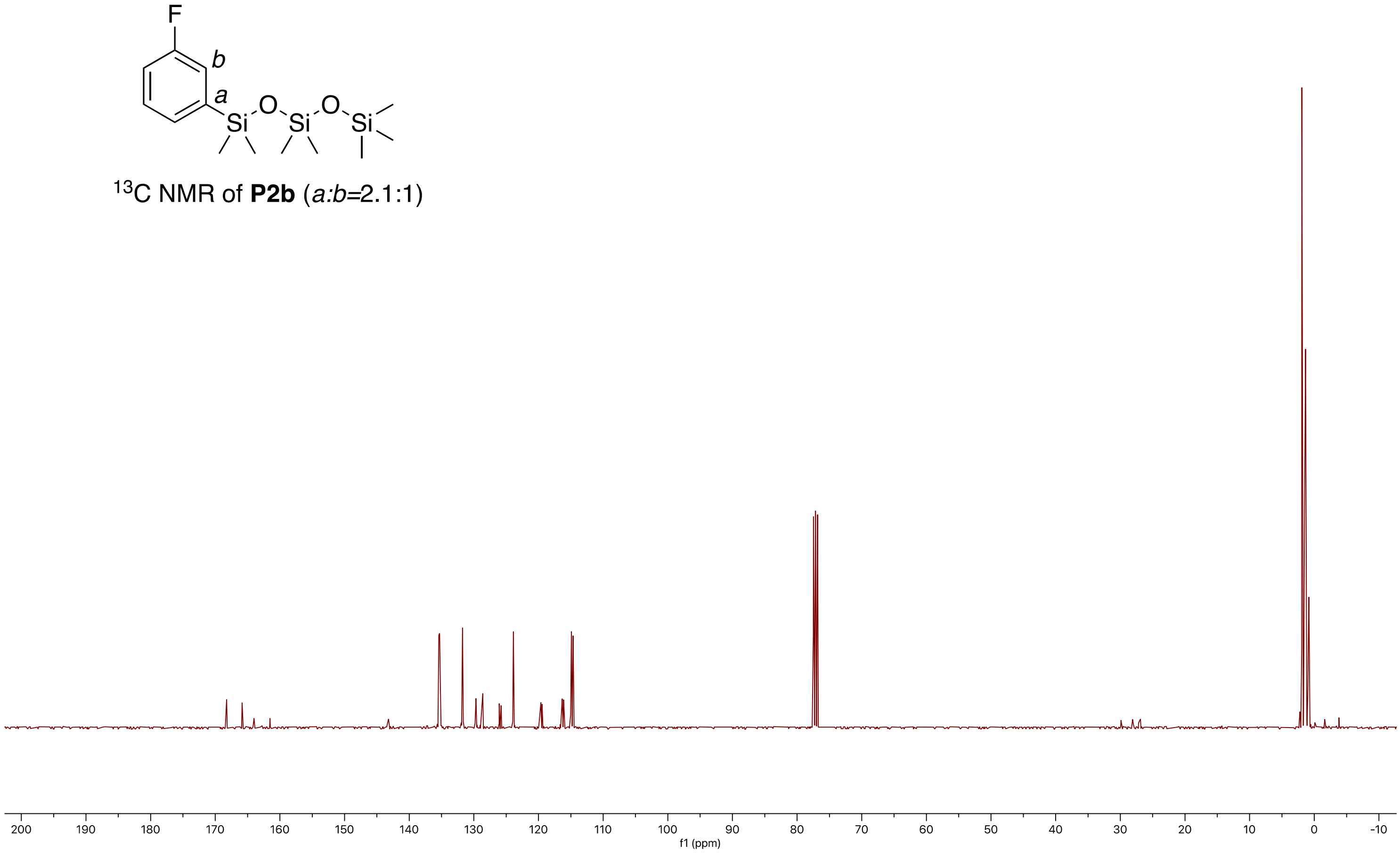

^{13}C NMR of **P2b** (*a*:*b*=2.1:1)

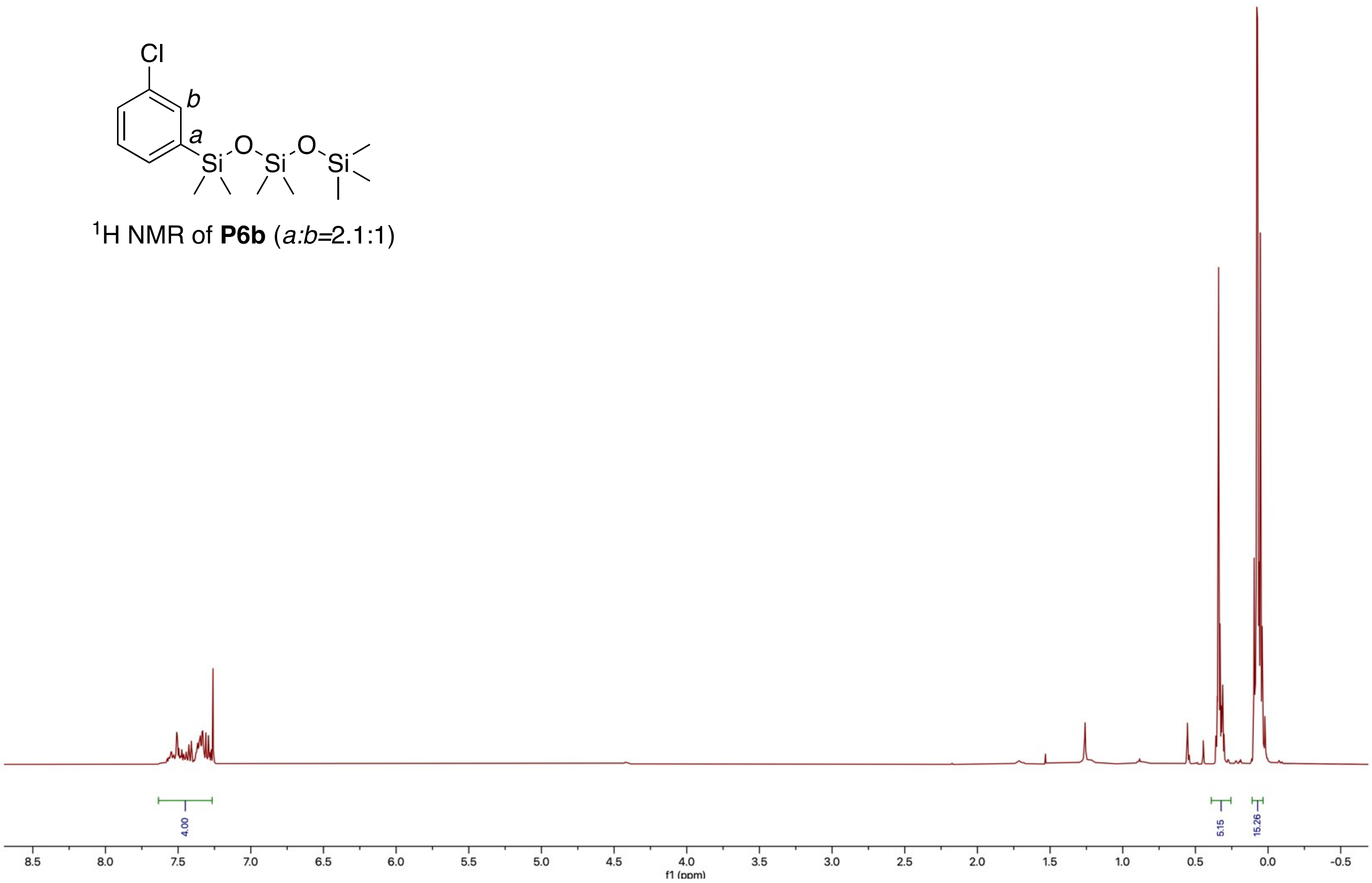

^{1}H NMR of **P6b** (*a*:*b*=2.1:1)

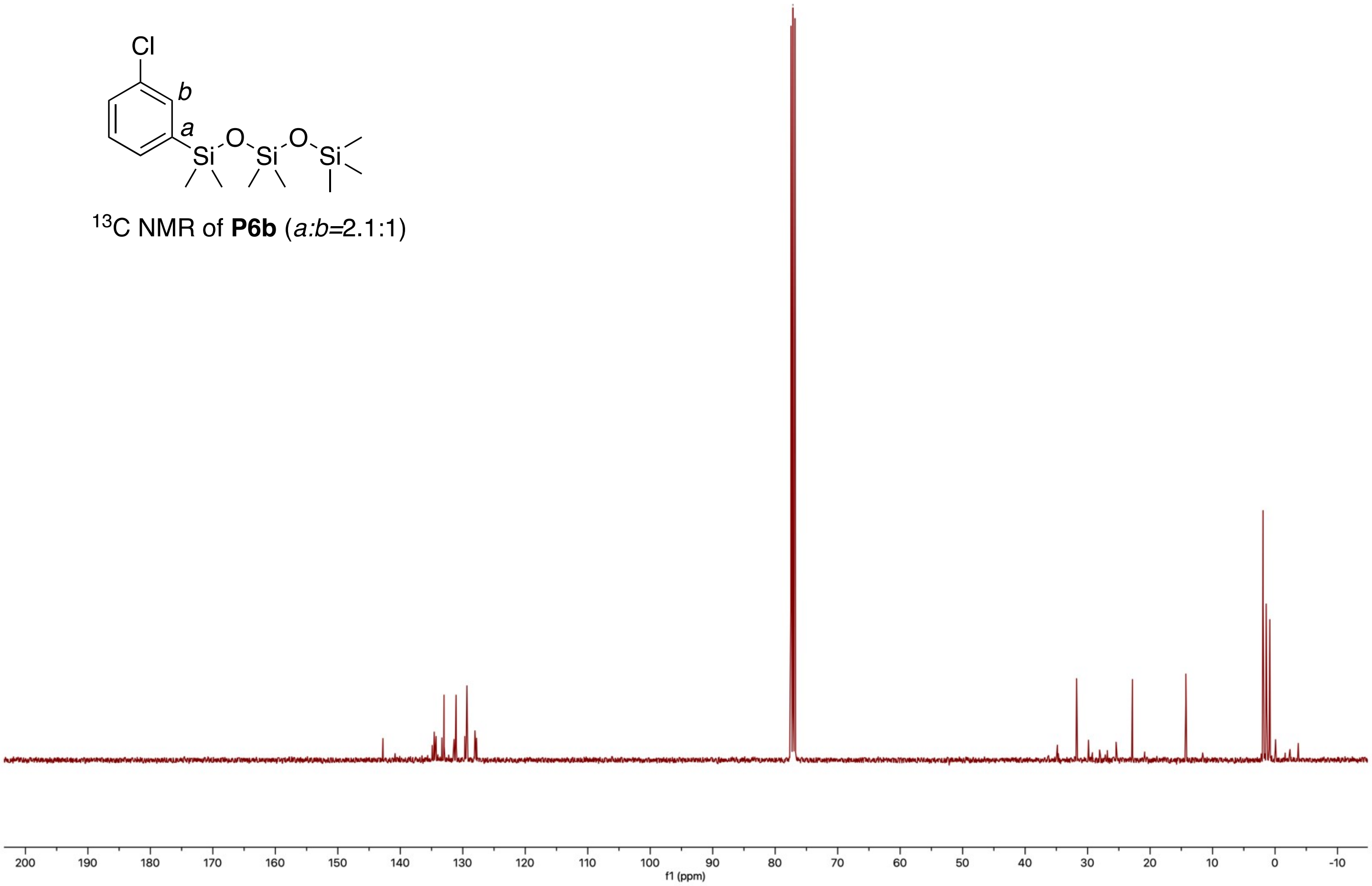

^{13}C NMR of **P6b** (*a*:*b*=2.1:1)

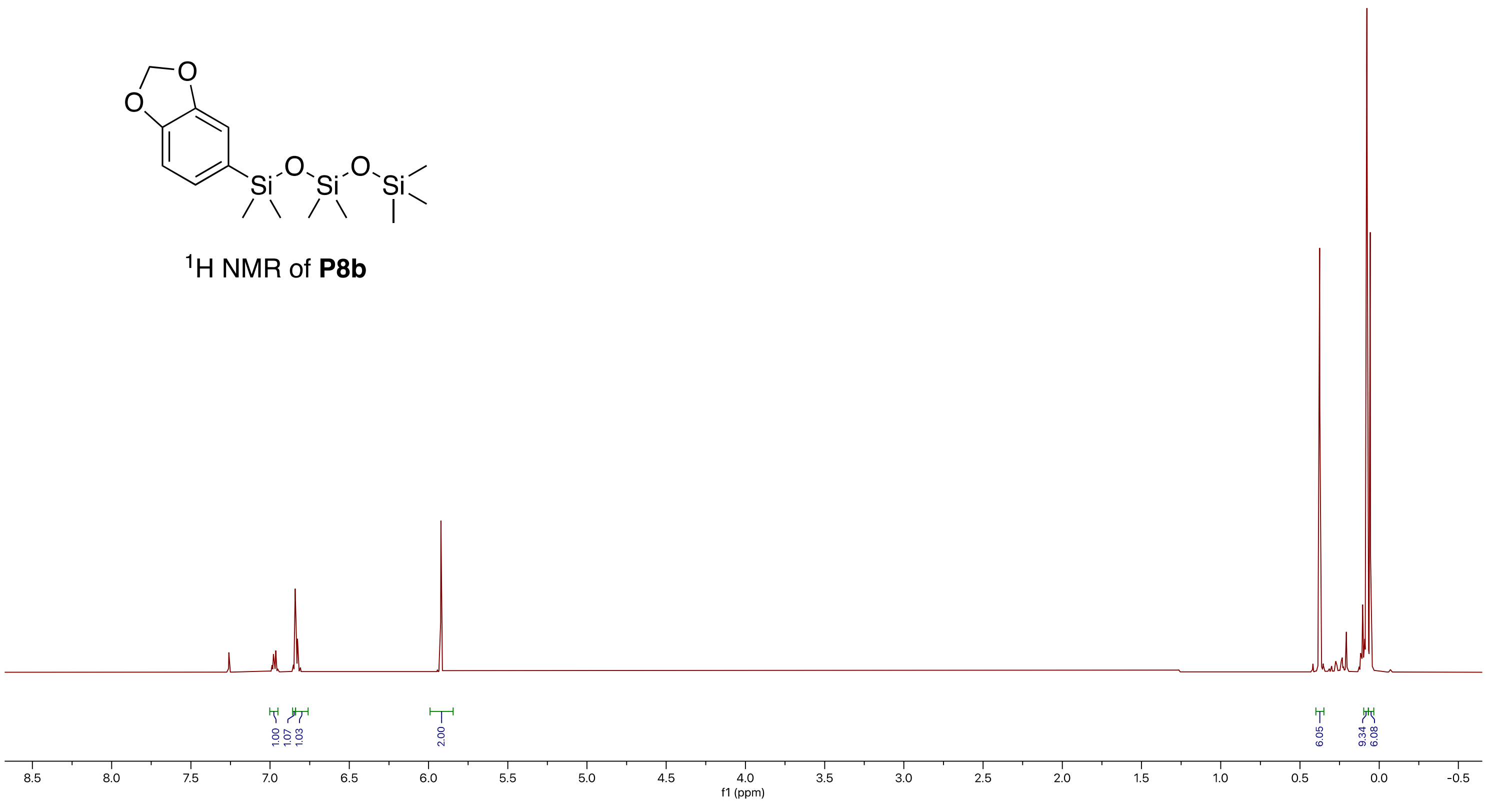
^{1}H NMR of **P8b**
1.00
1.07
1.03
2.00
6.05
9.34
6.08
f1 (ppm)

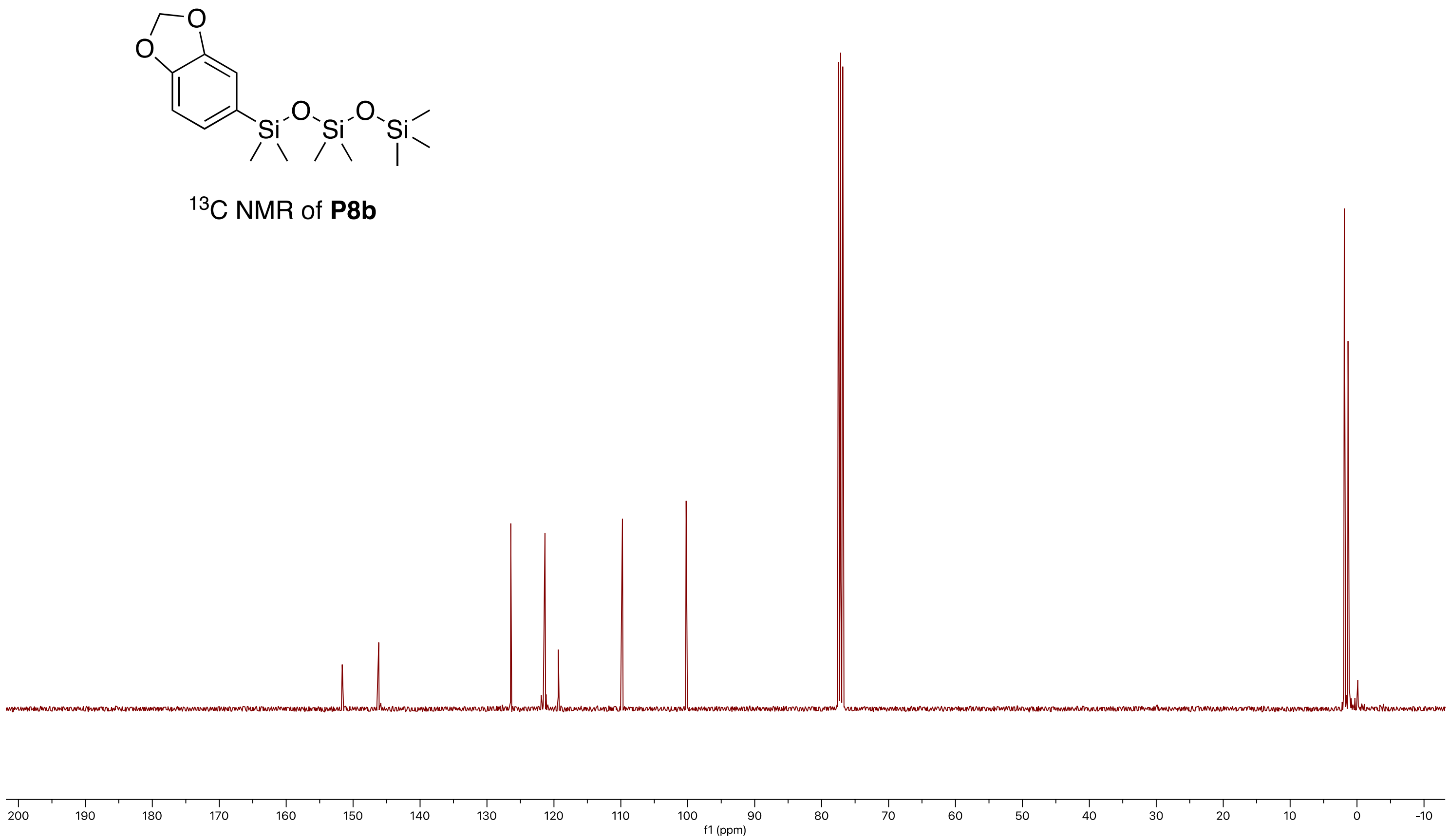
^{13}C NMR of **P8b**
f1 (ppm)

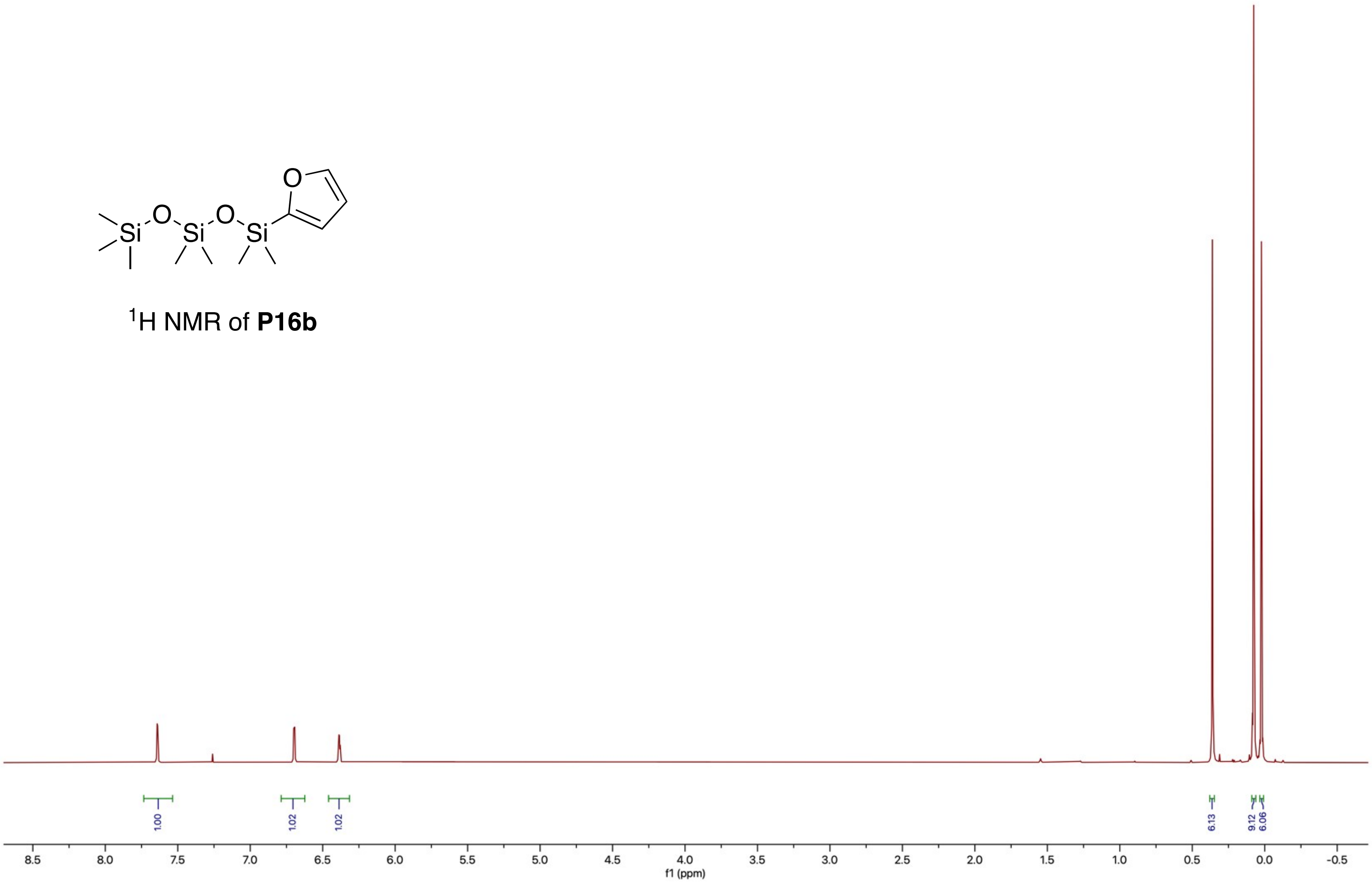

^{1}H NMR of **P16b**

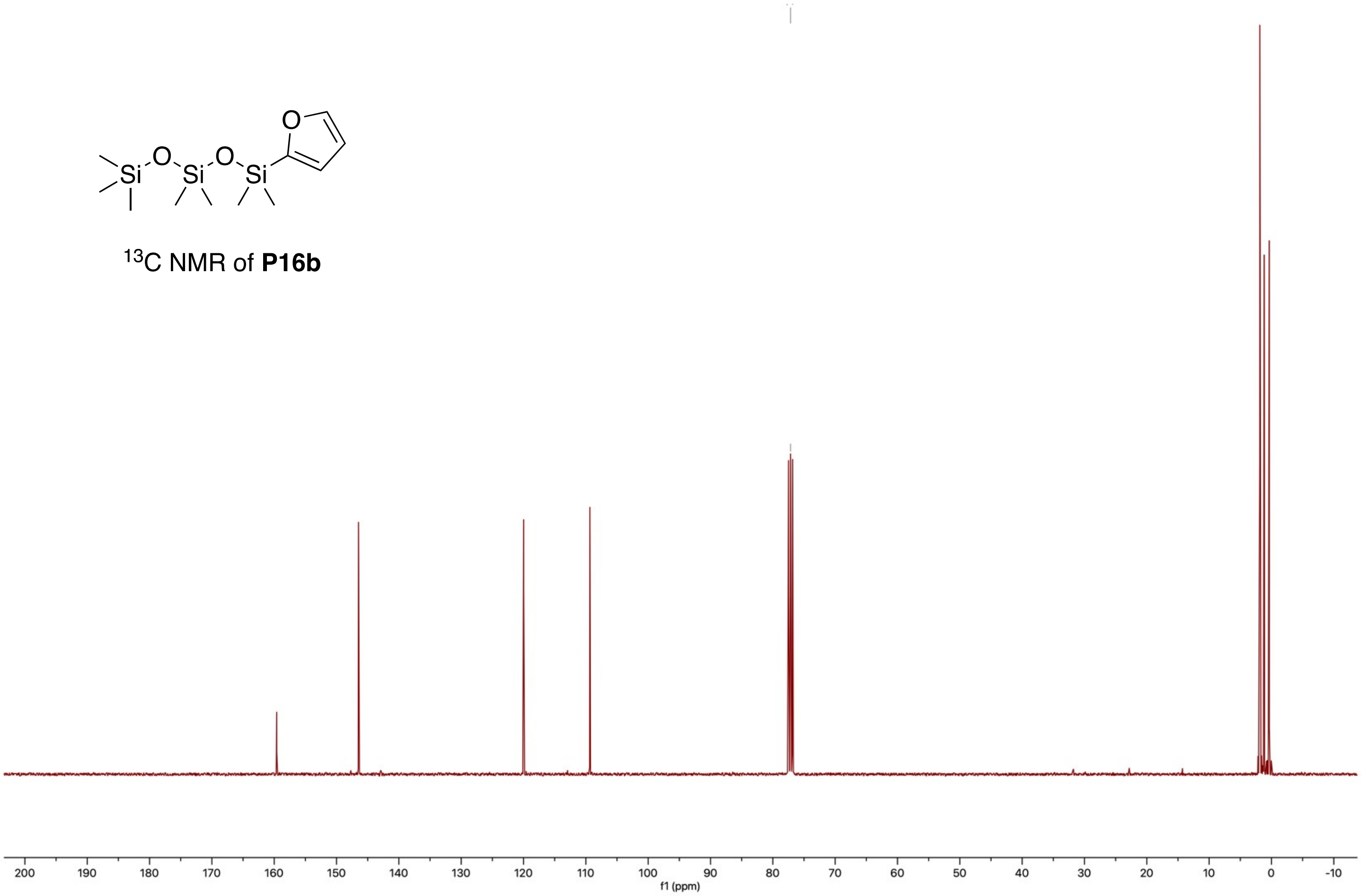

^{13}C NMR of **P16b**

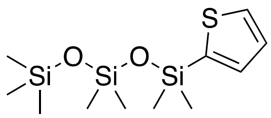

^{1}H NMR of **P17b**

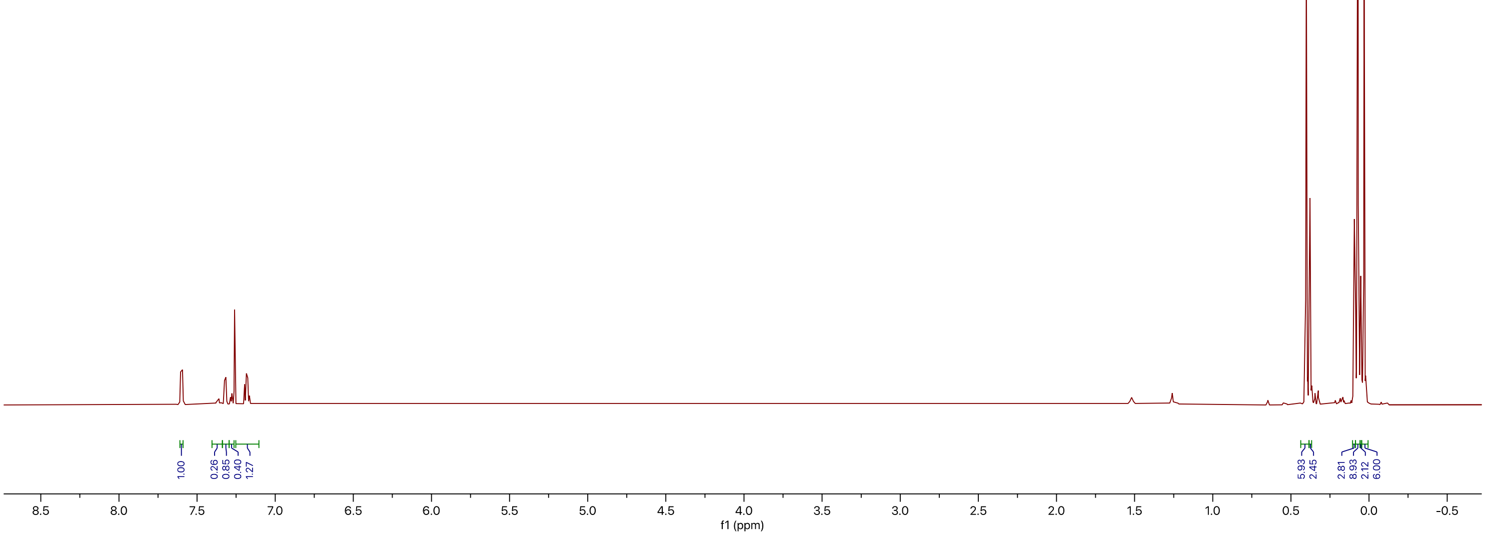

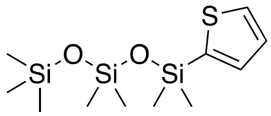

^{13}C NMR of **P17b**

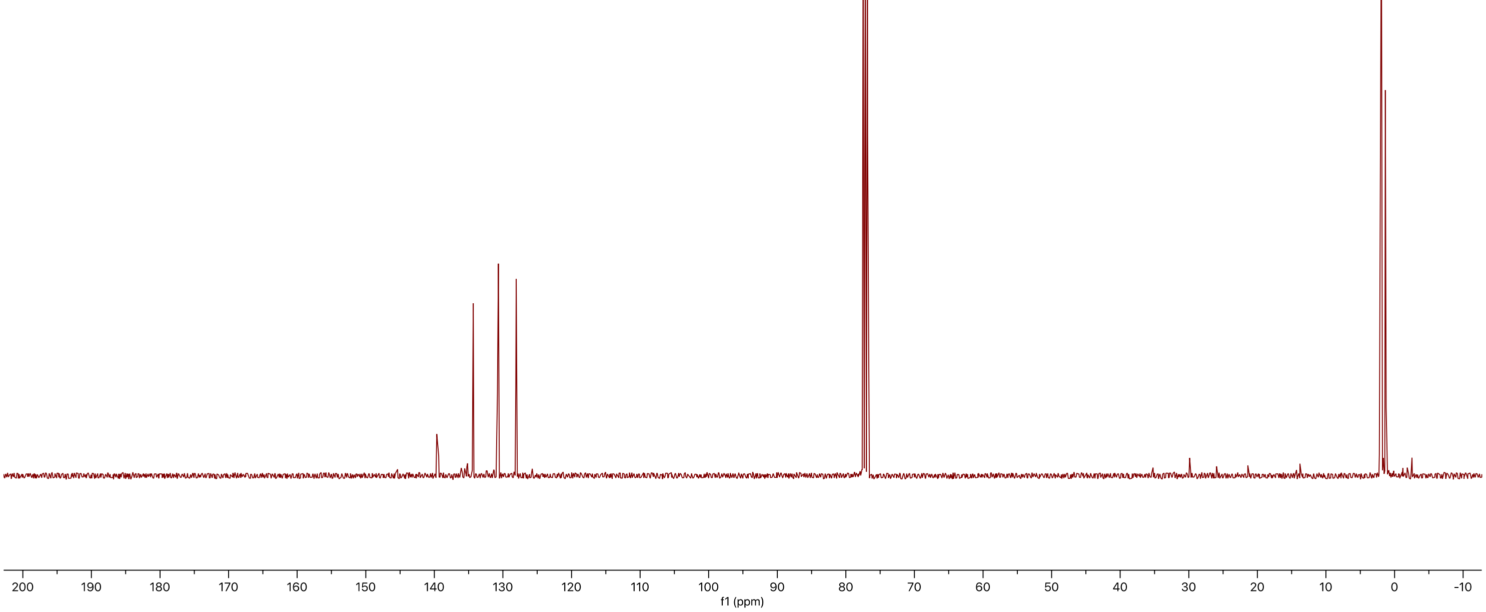

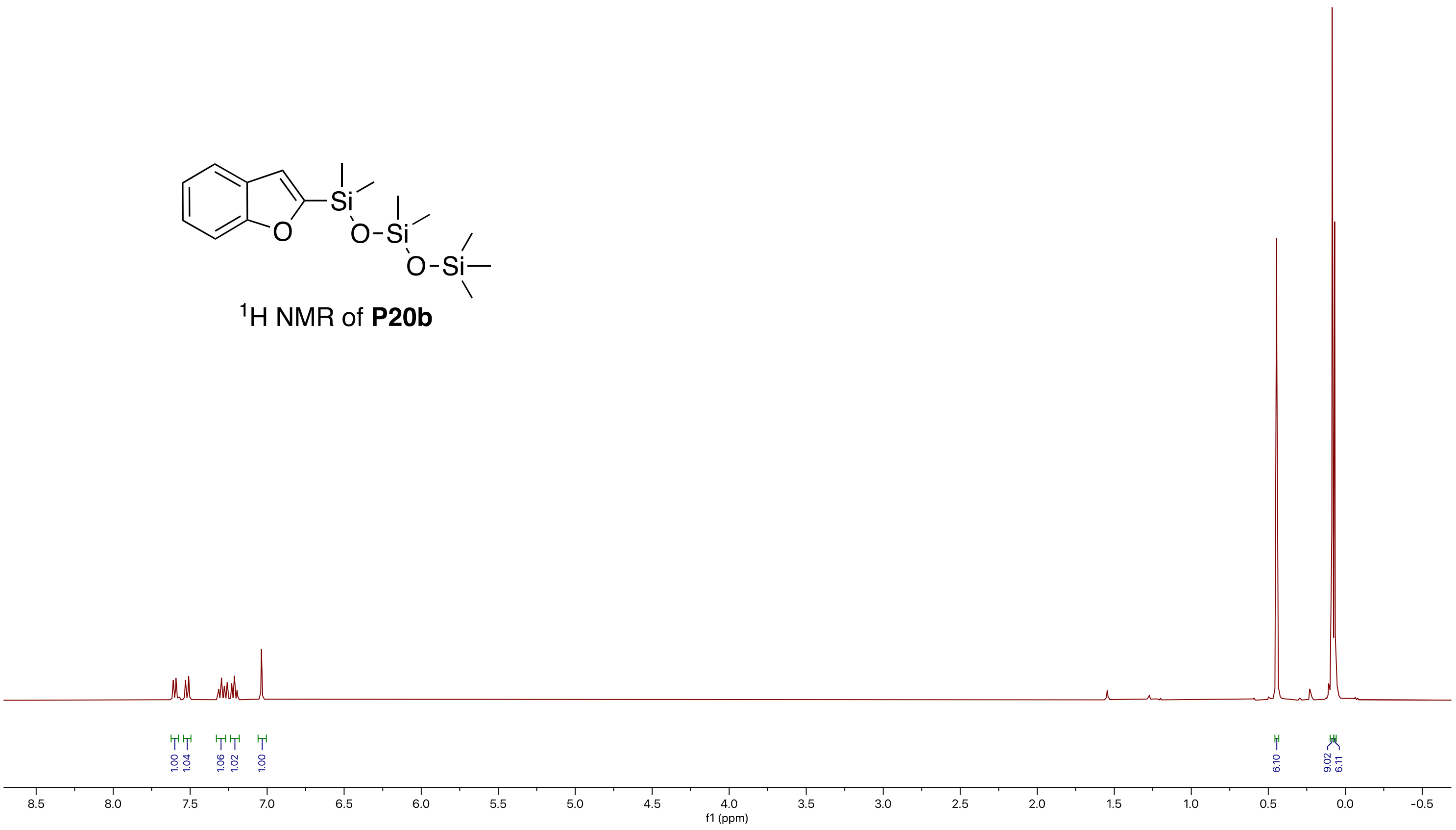

^{1}H NMR of **P20b**

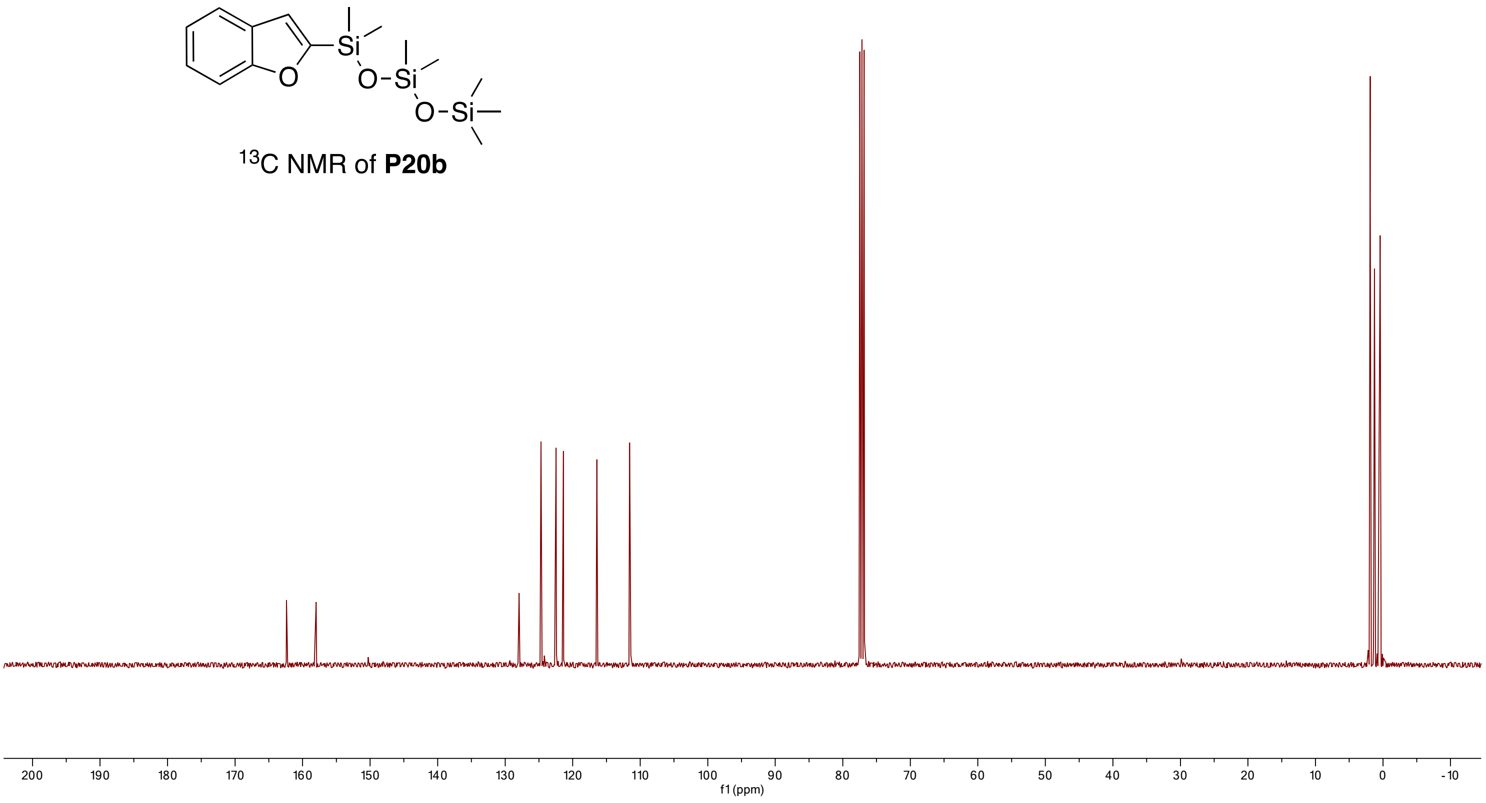

^{13}C NMR of **P20b**

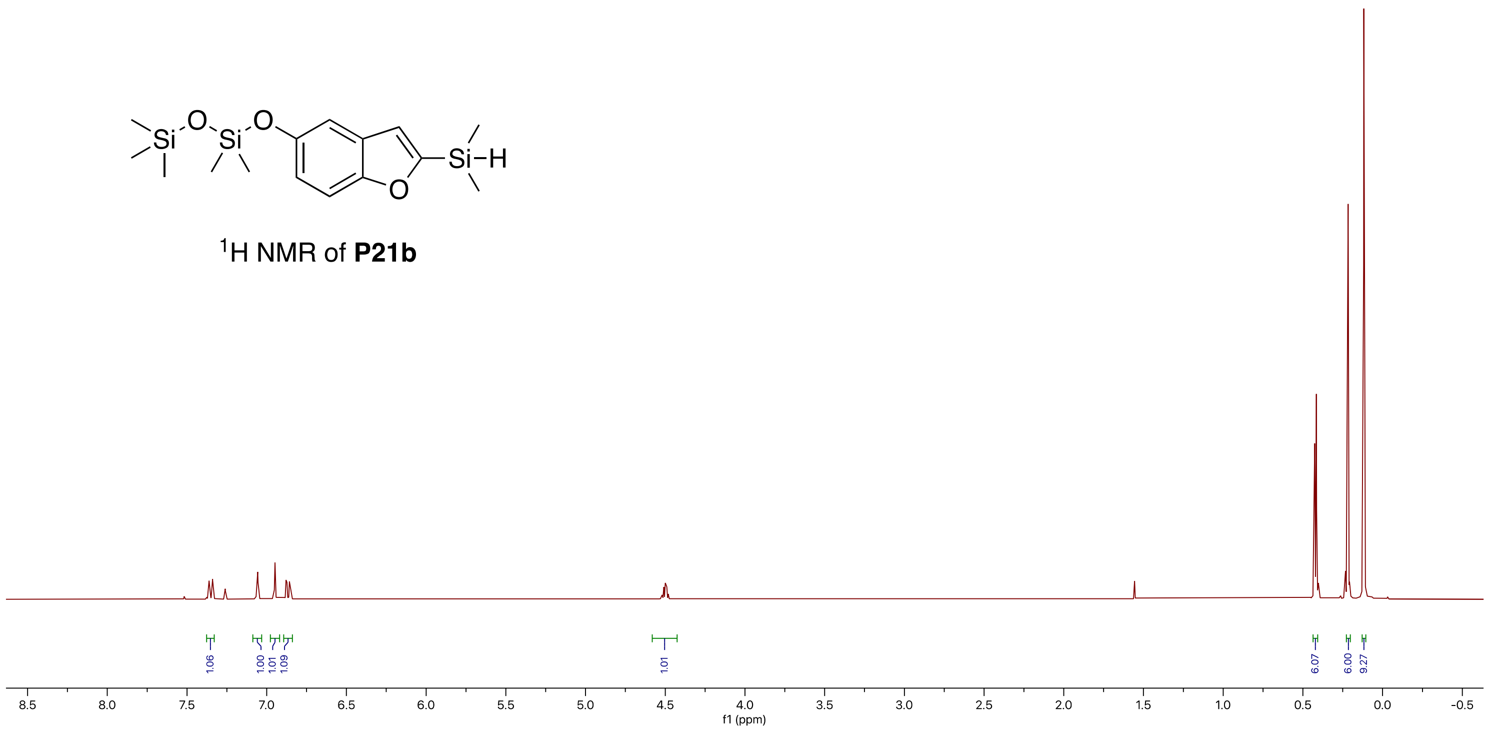

^{1}H NMR of **P21b**

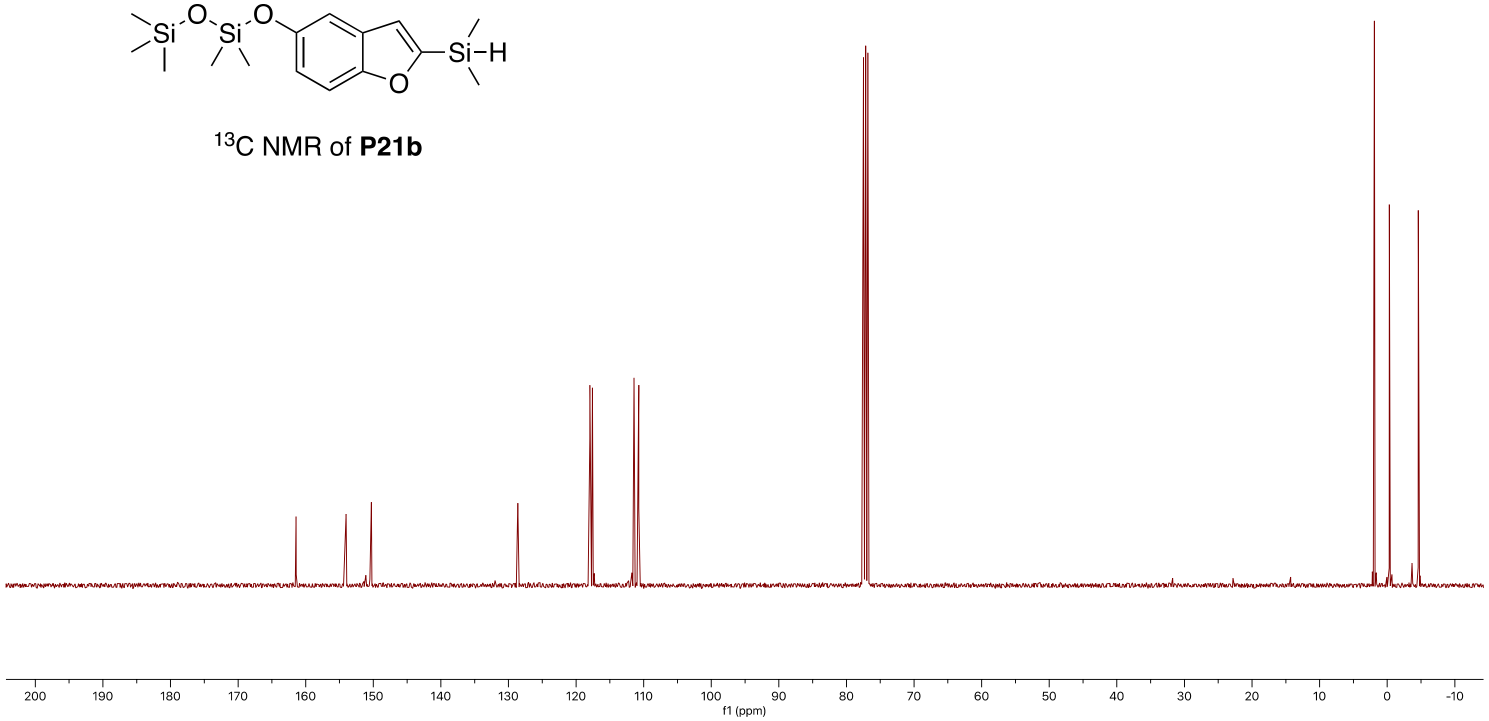

^{13}C NMR of **P21b**

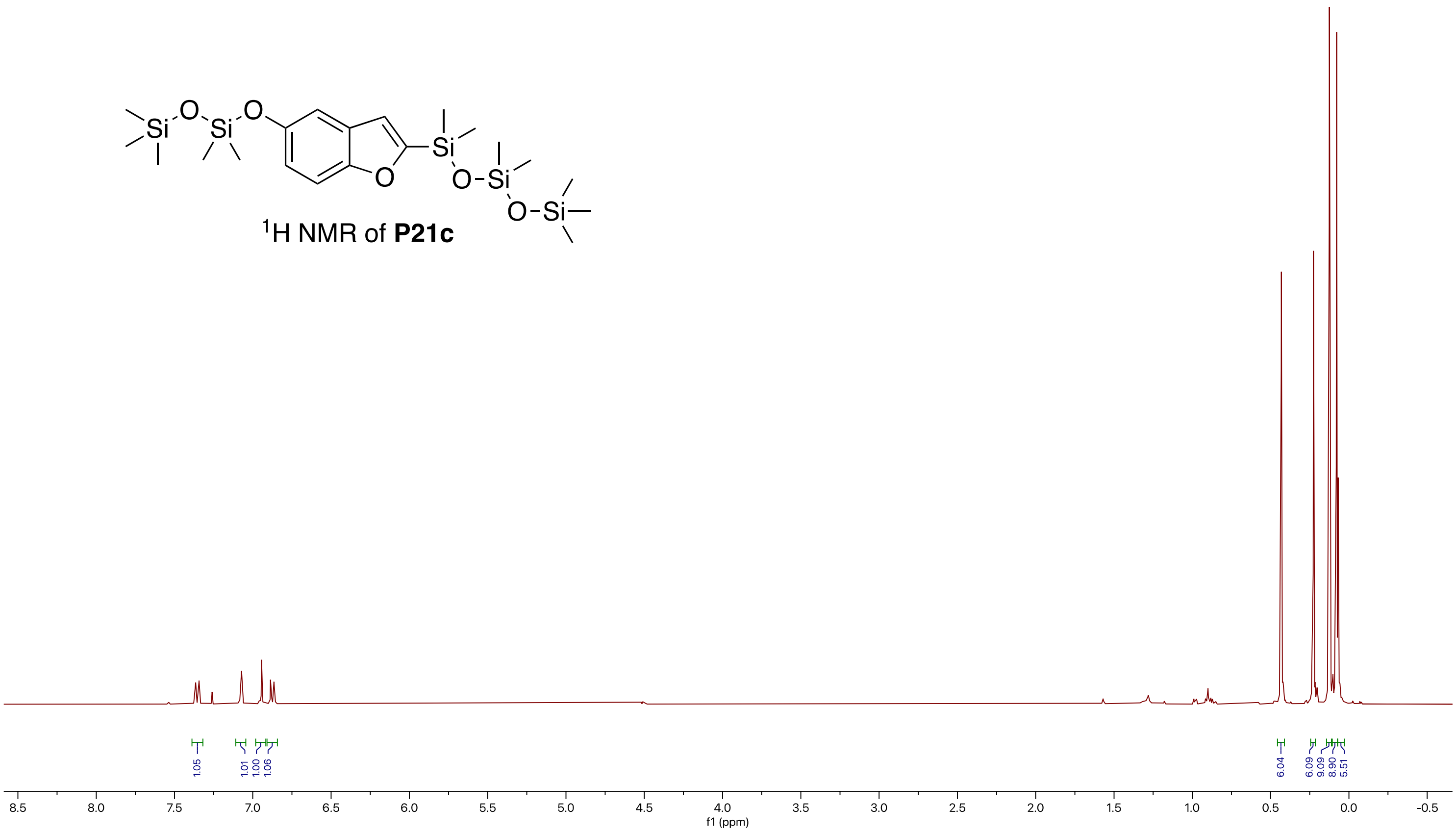

Si
O
Si
O
O
Si
O
Si
O
Si
1H NMR of P21c
1.05
1.01
1.00
1.06
6.04
6.09
9.09
8.90
5.51
8.5 8.0 7.5 7.0 6.5 6.0 5.5 5.0 4.5 4.0 3.5 3.0 2.5 2.0 1.5 1.0 0.5 0.0 -0.5
f1 (ppm)

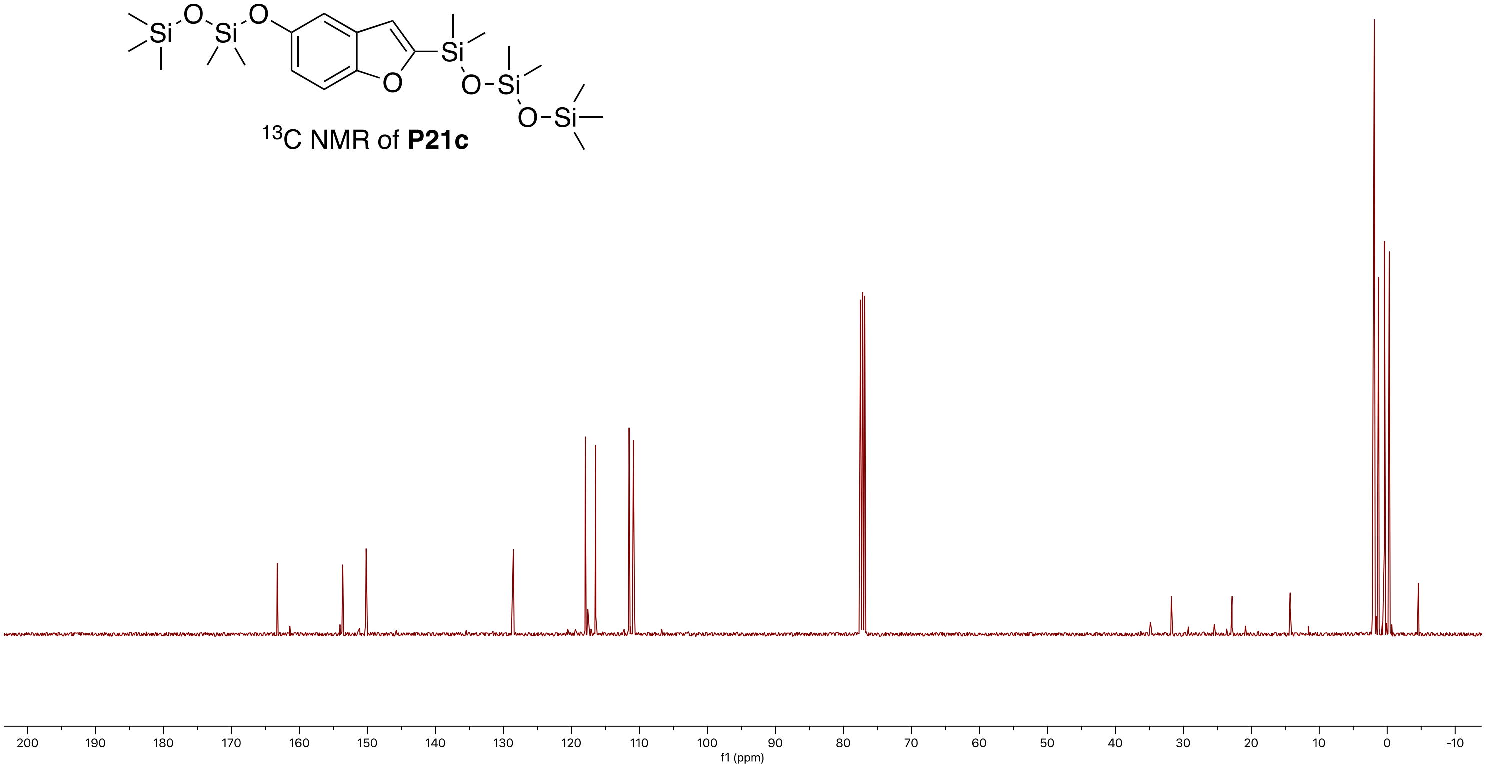

Si
O
Si
O
O
Si
O
Si
O
Si
13C NMR of P21c
200 190 180 170 160 150 140 130 120 110 100 90 80 70 60 50 40 30 20 10 0 -10
f1 (ppm)

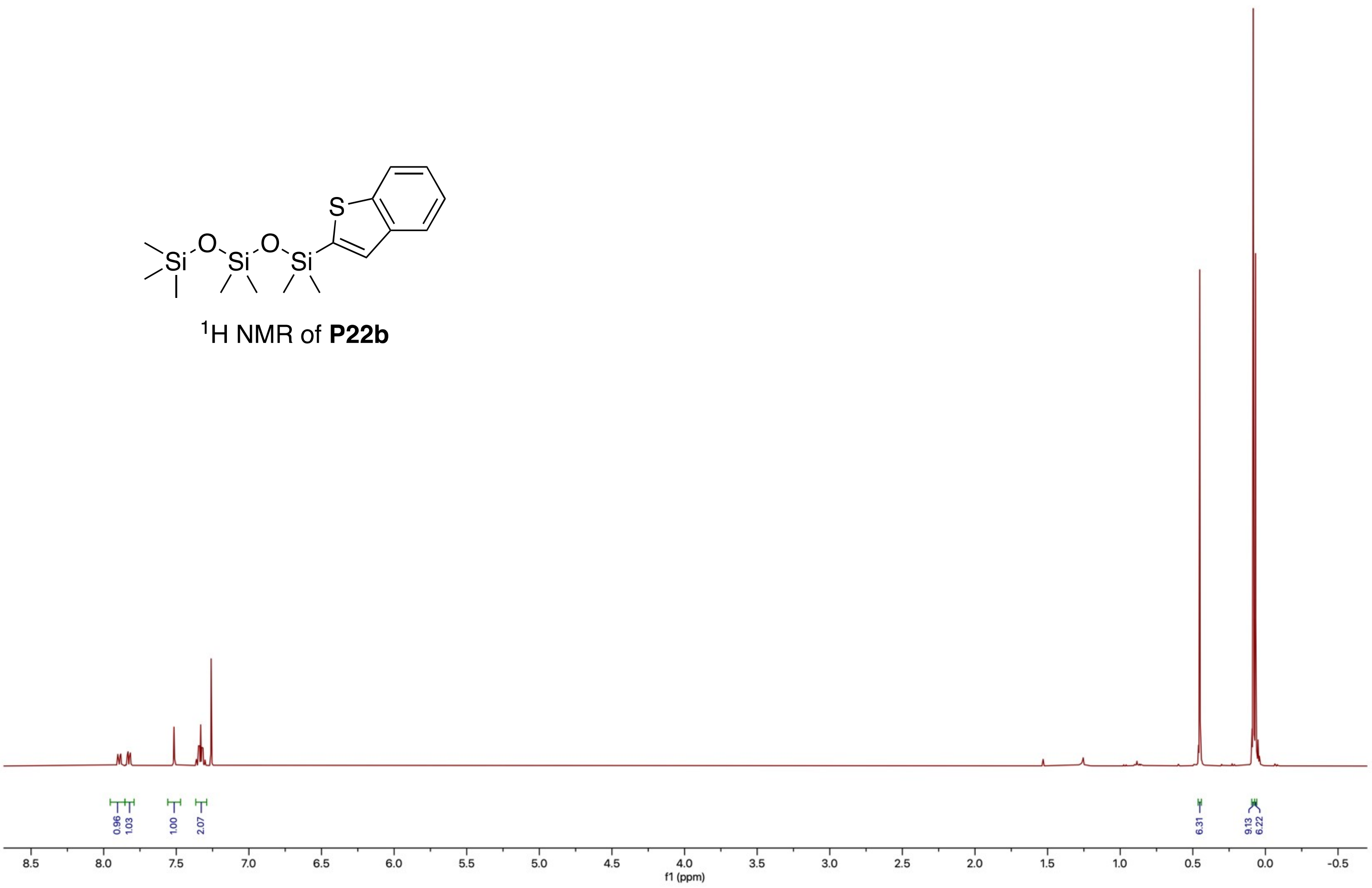

Si O Si O Si S
^{1}H NMR of **P22b**
0.96
1.03
1.00
2.07
6.31
9.13
6.22
8.5 8.0 7.5 7.0 6.5 6.0 5.5 5.0 4.5 4.0 3.5 3.0 2.5 2.0 1.5 1.0 0.5 0.0 -0.5
f1 (ppm)

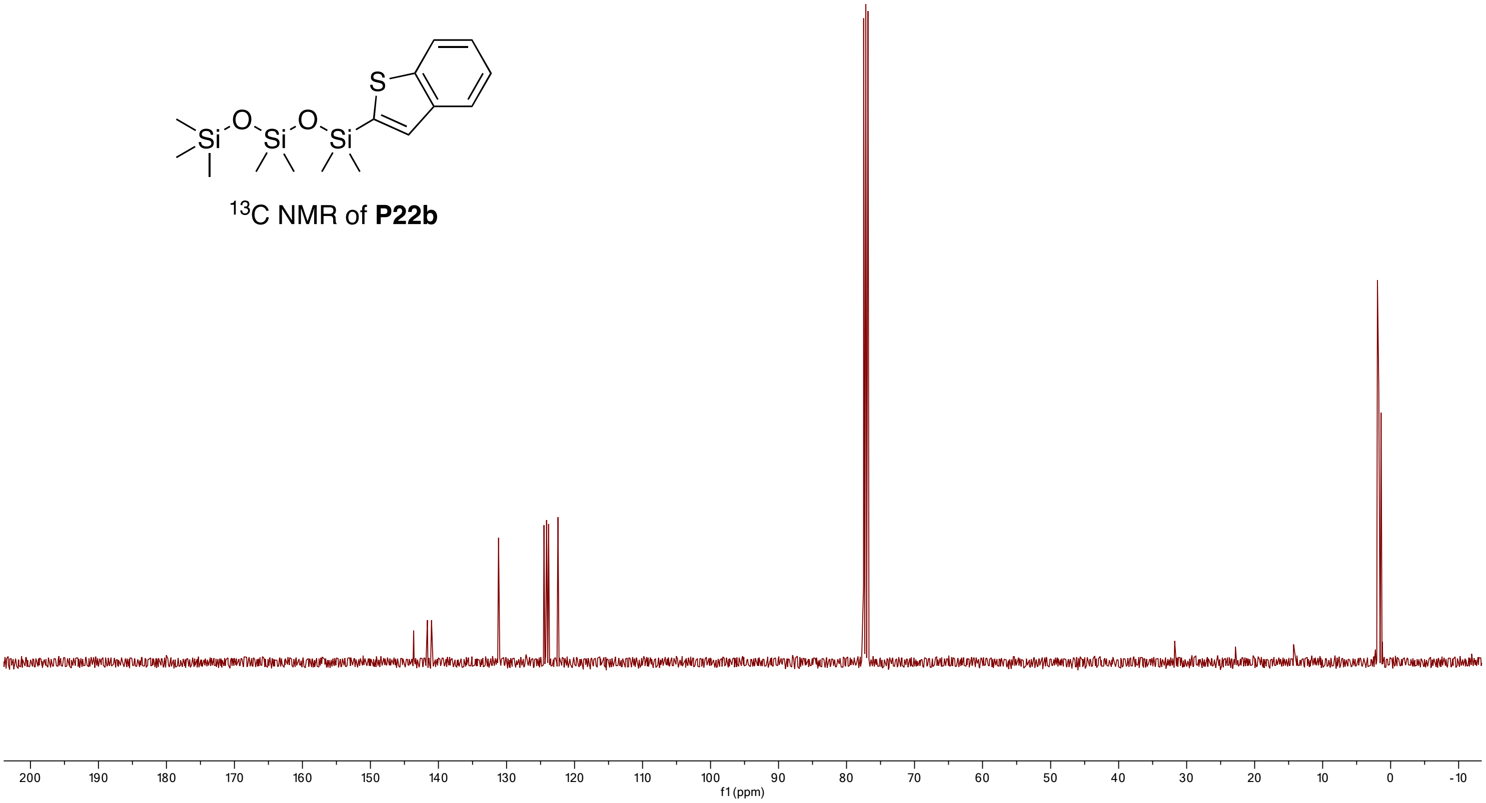

Si O Si O Si S
^{13}C NMR of **P22b**
200 190 180 170 160 150 140 130 120 110 100 90 80 70 60 50 40 30 20 10 0 -10
f1 (ppm)

5.4 Rh complexes

[RhCl(Ph-BPE)]$_2$ (2)

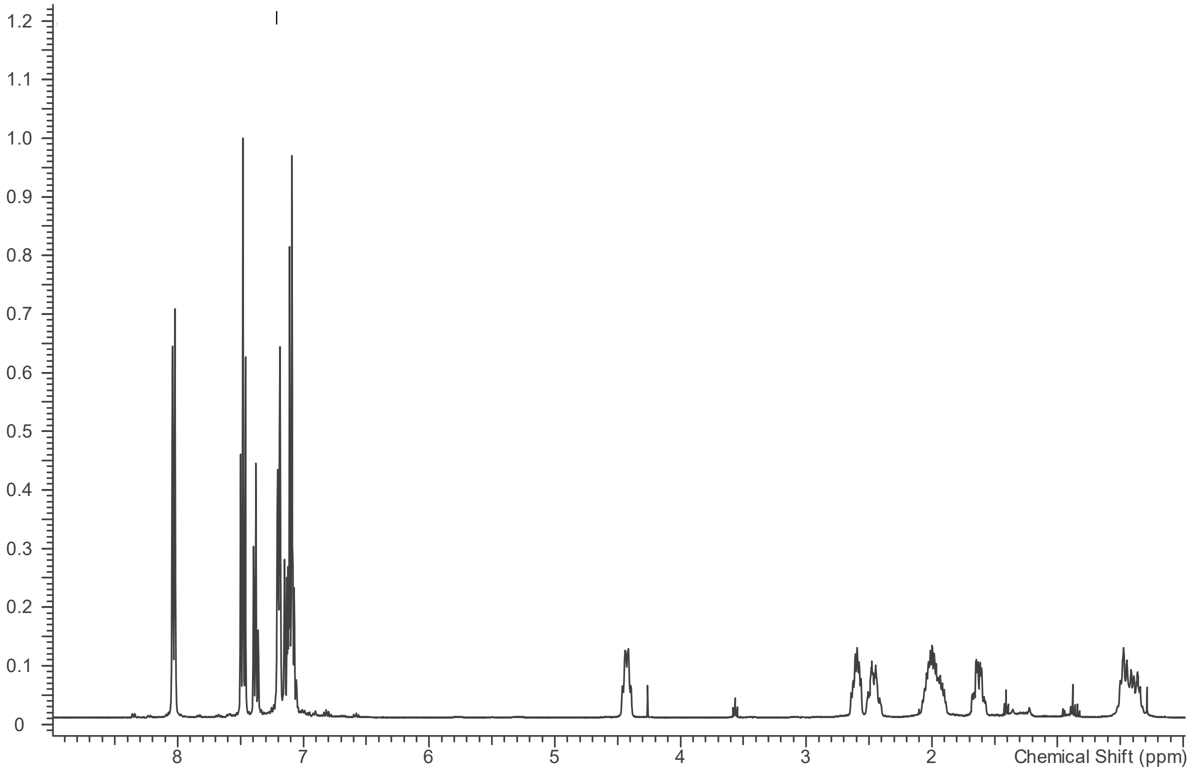

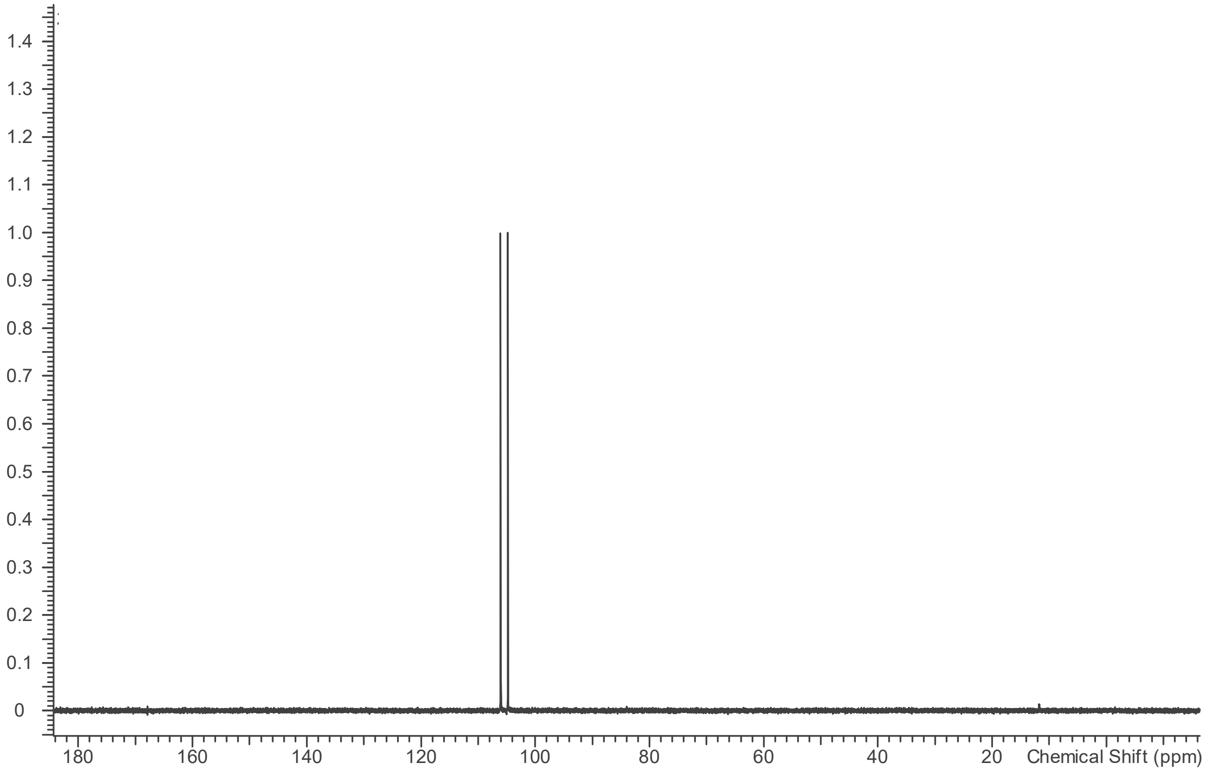

[Rh_2(Ph-BPE)$_2$(μ-Cl)(μ-H)] (5)

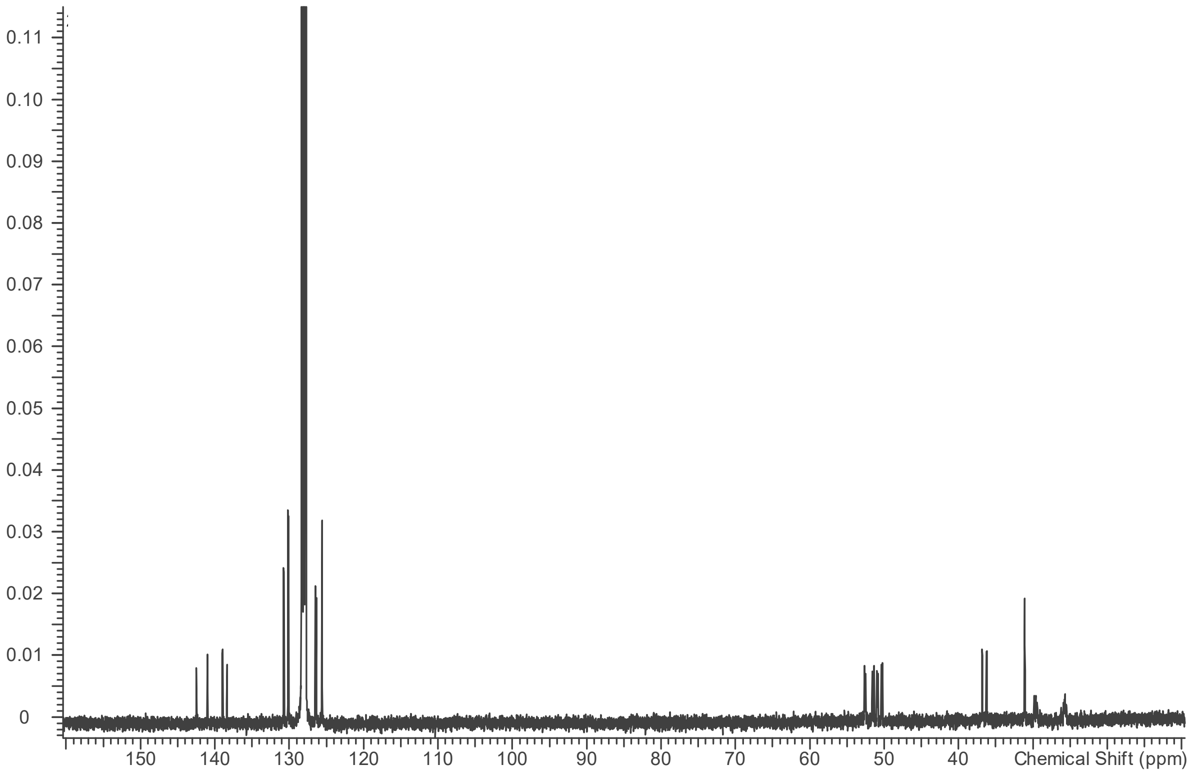

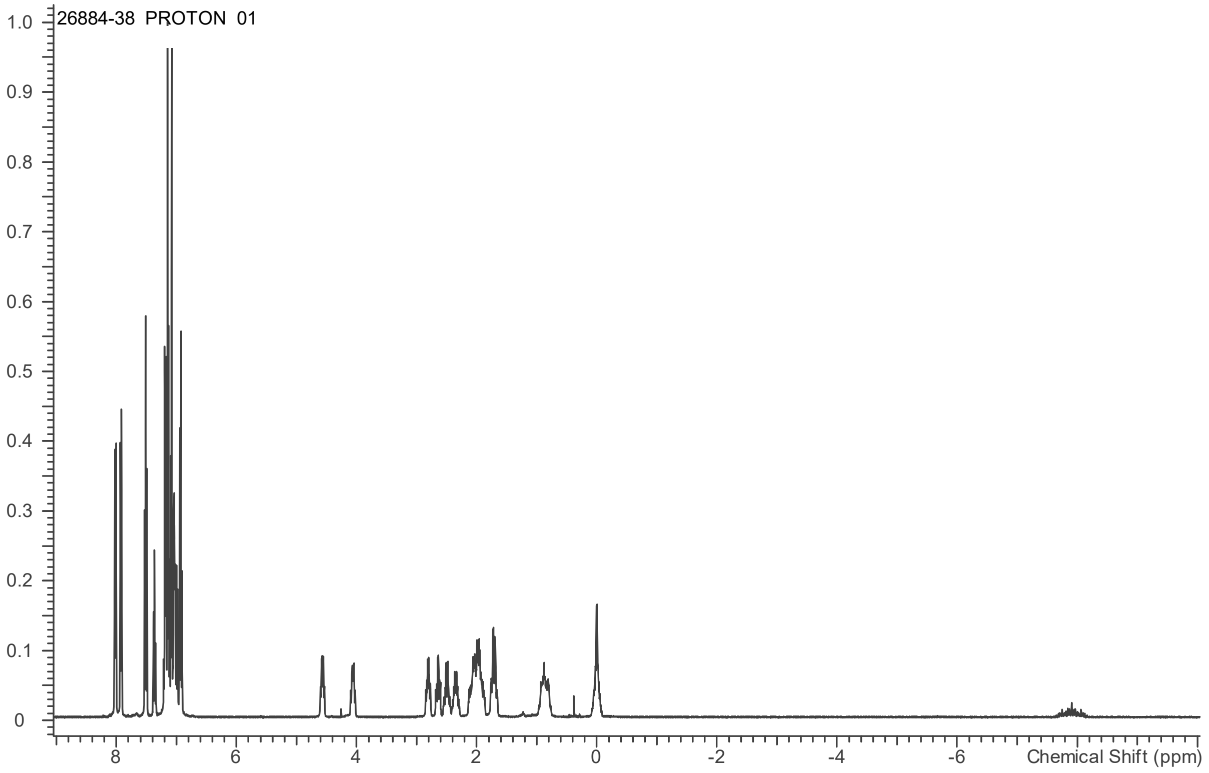

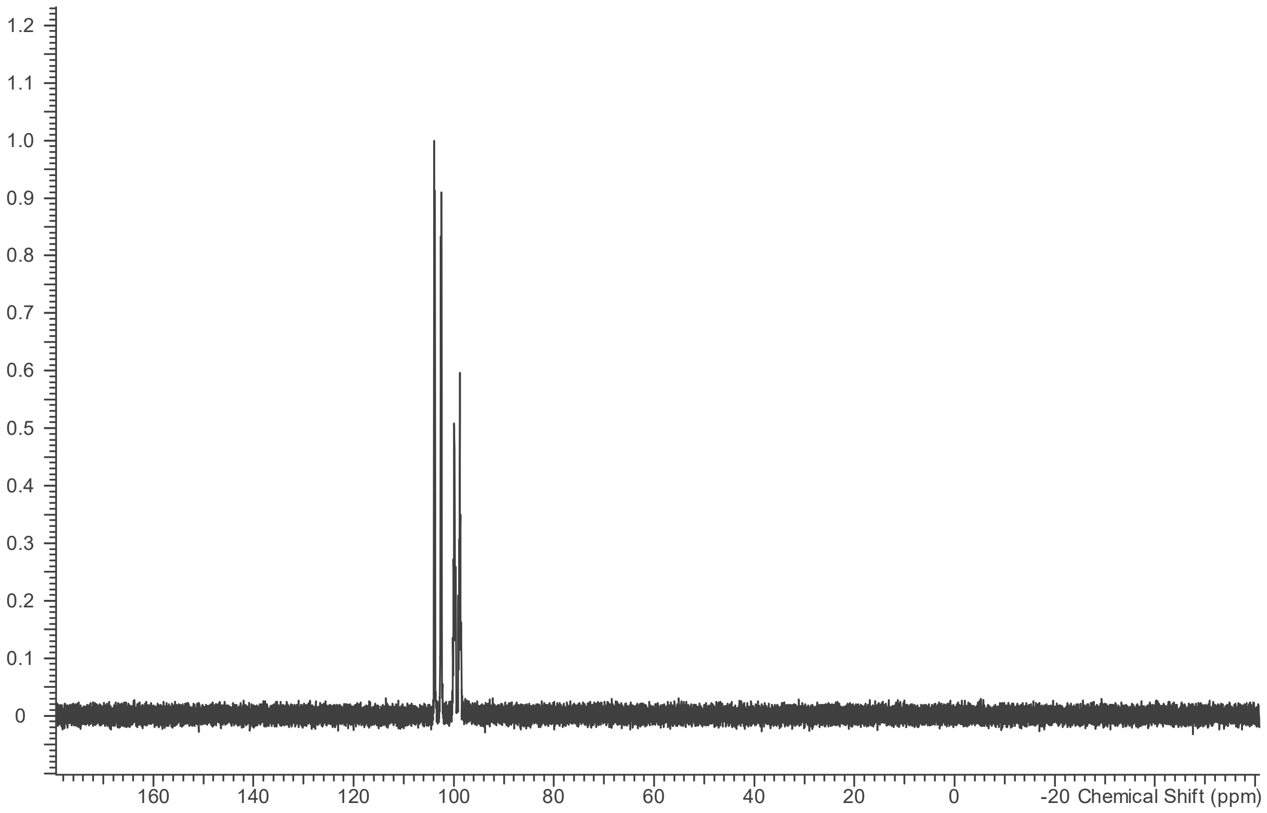

[(^{Ph}BPE)Rh(μ-H)]$_2$ (6)

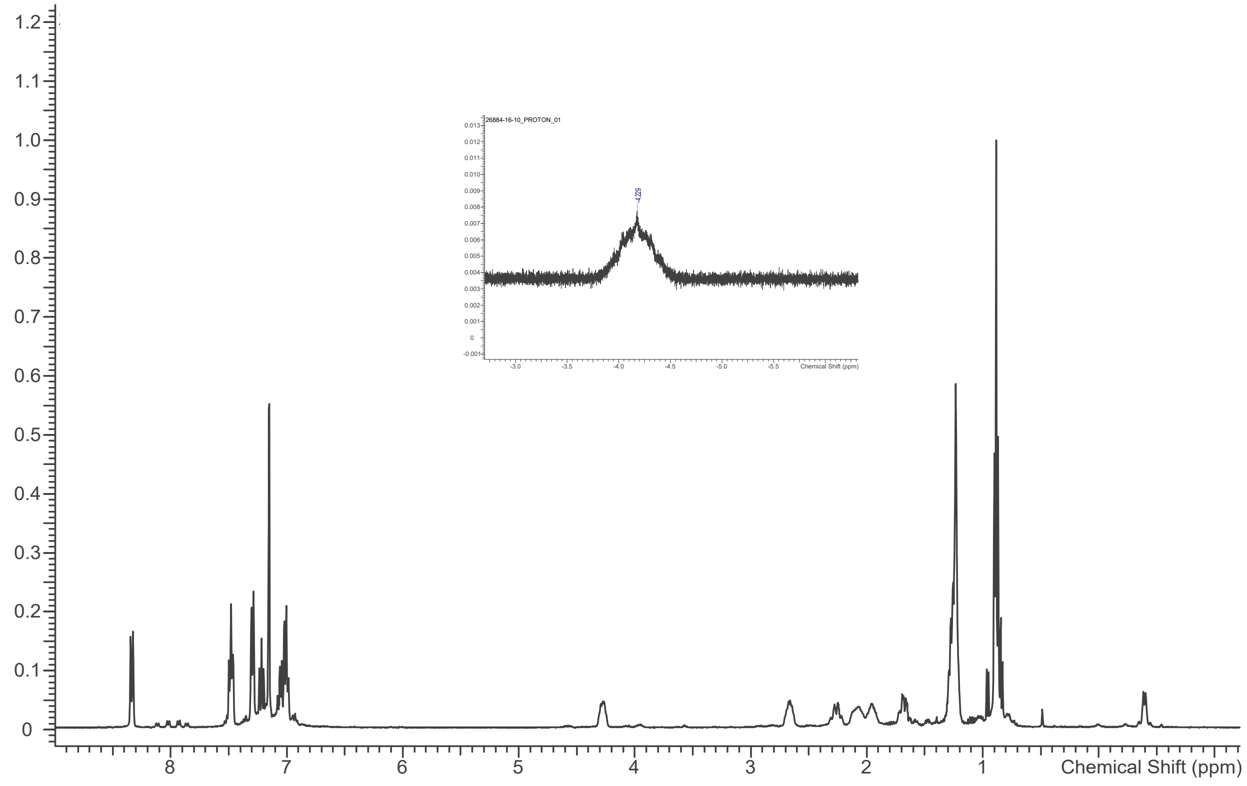

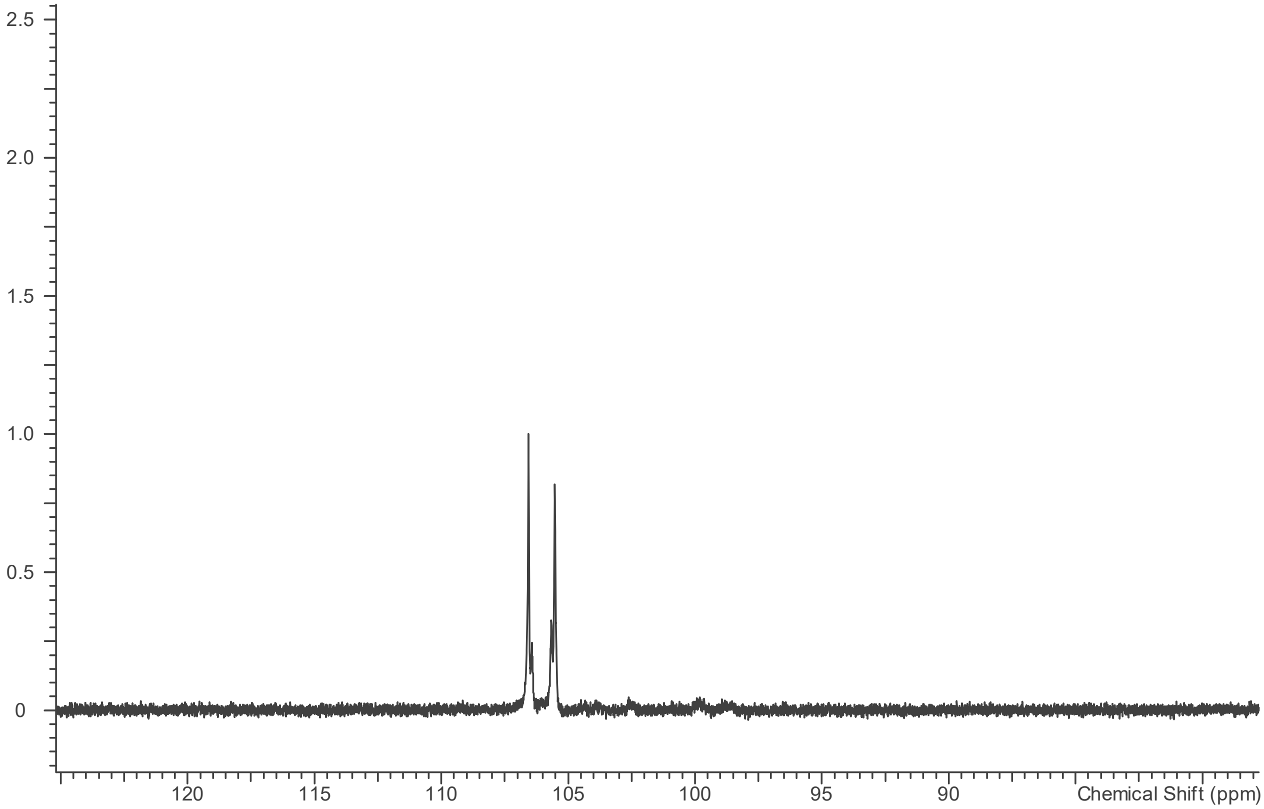

6. Crystallographic Data

6.1 [RhCl(Ph-BPE)]$_2$ (2)

Table S8. Crystal data and structure refinement for **2**.

Identification code	**2**	
Empirical formula	C34 H36 Cl P2 Rh	
Formula weight	644.93	
Temperature	100(2) K	
Wavelength	0.71073 Å	
Crystal system	Triclinic	
Space group	P1	
Unit cell dimensions	a = 10.3091(3) Å	□= 107.8640(10)°.
	b = 12.7665(4) Å	□= 101.0930(10)°.
	c = 13.3424(4) Å	□ = 112.0990(10)°.
Volume	1451.17(8) Å^3	
Z	2	

Density (calculated)	1.476 Mg/m^3
Absorption coefficient	0.813 mm^{-1}
F(000)	664
Crystal size	0.333 x 0.186 x 0.072 mm^3
Theta range for data collection	1.711 to 28.431°.
Index ranges	-13<=h<=13, -16<=k<=17, -17<=l<=17
Reflections collected	26262
Independent reflections	12341 [R(int) = 0.0180]
Completeness to theta = 25.000°	100.0 %
Absorption correction	None
Max. and min. transmission	1.0 and 0.915
Refinement method	Full-matrix least-squares on F^2
Data / restraints / parameters	12341 / 3 / 685
Goodness-of-fit on F^2	1.030
Final R indices [I>2sigma(I)]	R1 = 0.0203, wR2 = 0.0459
R indices (all data)	R1 = 0.0227, wR2 = 0.0469
Absolute structure parameter	-0.006(7)
Extinction coefficient	n/a
Largest diff. peak and hole	0.597 and -0.438 e.Å^{-3}

l

Table S9. Atomic coordinates (x 10^4) and equivalent isotropic displacement parameters (Å^{2}x 10^3) for **2**. U(eq) is defined as one third of the trace of the orthogonalized U^{ij} tensor.

	x	y	z	U(eq)
Rh(01)	4407(1)	4900(1)	10268(1)	10(1)
Rh(02)	4994(1)	5211(1)	7792(1)	10(1)
Cl(1)	3412(1)	5498(1)	8859(1)	14(1)
P(3)	5151(1)	4322(1)	11535(1)	11(1)
P(4)	2916(1)	5182(1)	11139(1)	12(1)
P(1)	6297(1)	4879(1)	6760(1)	12(1)
P(2)	4188(1)	5898(1)	6655(1)	12(1)
Cl(2)	6061(1)	4675(1)	9229(1)	15(1)

C(42)	3439(5)	817(3)	13137(3)	25(1)
C(18)	4782(4)	5608(3)	5428(3)	17(1)
C(38)	7101(4)	4725(3)	12269(3)	15(1)
C(35)	4628(4)	2628(3)	10963(3)	15(1)
C(29)	1229(4)	4109(3)	5196(3)	17(1)
C(34)	1047(4)	3171(3)	5569(3)	21(1)
C(26)	6052(5)	9120(3)	10845(3)	32(1)
C(17)	6296(4)	5643(3)	5788(3)	18(1)
C(4)	8277(3)	5282(3)	7400(3)	15(1)
C(16)	9544(3)	7288(3)	9125(3)	18(1)
C(9)	5779(4)	1423(3)	7605(3)	25(1)
C(57)	203(3)	3106(3)	9511(3)	18(1)
C(47)	9473(3)	8063(3)	12730(3)	22(1)
C(27)	4745(5)	8999(3)	10213(3)	27(1)
C(10)	6272(3)	2149(3)	7033(3)	21(1)
C(2)	7242(3)	3391(3)	5537(3)	22(1)
C(50)	8966(4)	6679(3)	13971(3)	21(1)
C(22)	2183(4)	5455(3)	6013(3)	15(1)
C(53)	834(3)	4390(3)	10424(3)	17(1)
C(8)	4305(5)	959(3)	7555(3)	26(1)
C(67)	4641(4)	9106(3)	14625(3)	24(1)
C(45)	8210(4)	6092(3)	12822(3)	16(1)
C(30)	476(4)	3760(4)	4060(3)	24(1)
C(52)	3075(3)	4804(3)	12374(3)	14(1)
C(11)	9286(3)	6664(3)	7993(3)	16(1)
C(31)	-420(4)	2510(4)	3335(3)	30(1)
C(39)	4272(3)	2022(2)	11756(2)	16(1)
C(48)	10241(3)	8633(3)	13888(3)	24(1)
C(32)	-578(4)	1599(3)	3715(3)	27(1)
C(60)	-862(4)	701(3)	7883(3)	35(1)
C(49)	9979(4)	7941(3)	14506(3)	26(1)
C(19)	4722(4)	7599(3)	7214(3)	19(1)
C(6)	3829(3)	1941(3)	6358(3)	20(1)
C(41)	4810(4)	1097(3)	13010(3)	22(1)
C(13)	10794(3)	8641(3)	7987(3)	24(1)

C(59)	-921(4)	886(3)	8943(4)	40(1)
C(65)	6607(4)	8782(3)	14114(3)	27(1)
C(20)	3410(4)	7705(3)	6539(3)	28(1)
C(44)	2881(3)	1728(3)	11889(3)	21(1)
C(58)	-401(3)	2077(3)	9751(3)	28(1)
C(62)	250(3)	2910(3)	8445(3)	22(1)
C(33)	157(4)	1930(3)	4834(3)	25(1)
C(24)	6509(3)	8301(3)	9142(3)	23(1)
C(46)	8470(4)	6804(3)	12207(3)	19(1)
C(64)	5614(3)	7954(3)	13047(3)	22(1)
C(21)	2263(3)	6450(3)	5594(3)	20(1)
C(1)	5823(3)	3265(3)	5821(2)	16(1)
C(12)	9929(3)	7359(3)	7432(3)	19(1)
C(36)	5980(3)	2649(3)	10608(3)	20(1)
C(7)	3324(4)	1216(3)	6923(3)	26(1)
C(3)	8667(3)	4516(3)	6483(3)	21(1)
C(28)	4298(3)	8518(3)	9041(3)	22(1)
C(61)	-288(4)	1707(3)	7631(3)	30(1)
C(51)	4549(4)	4779(3)	12739(3)	15(1)
C(14)	11065(4)	9244(3)	9120(3)	26(1)
C(68)	3631(3)	8251(3)	13552(2)	19(1)
C(23)	5177(3)	8169(2)	8486(2)	18(1)
C(55)	1441(3)	6570(3)	11228(3)	21(1)
C(66)	6117(5)	9366(4)	14910(3)	27(1)
C(37)	7448(3)	3852(3)	11407(3)	19(1)
C(5)	5306(3)	2418(2)	6403(2)	18(1)
C(25)	6941(4)	8768(3)	10299(3)	32(1)
C(15)	10438(3)	8564(3)	9684(3)	25(1)
C(40)	5232(3)	1711(3)	12334(2)	18(1)
C(43)	2479(4)	1145(3)	12588(3)	25(1)
C(63)	4102(3)	7667(3)	12741(2)	16(1)
C(56)	3061(3)	6774(3)	11561(2)	16(1)
C(54)	520(3)	5375(3)	10154(2)	18(1)

Table S10. Bond lengths [Å] and angles [°] for **2**.

Rh(01)-P(3)	2.1640(9)
Rh(01)-P(4)	2.1655(9)
Rh(01)-Cl(1)	2.4291(9)
Rh(01)-Cl(2)	2.4380(9)
Rh(02)-P(2)	2.1533(10)
Rh(02)-P(1)	2.1595(9)
Rh(02)-Cl(1)	2.4168(9)
Rh(02)-Cl(2)	2.4334(9)
P(3)-C(51)	1.840(3)
P(3)-C(38)	1.850(3)
P(3)-C(35)	1.872(4)
P(4)-C(52)	1.849(3)
P(4)-C(56)	1.872(3)
P(4)-C(53)	1.878(3)
P(1)-C(17)	1.847(3)
P(1)-C(4)	1.852(3)
P(1)-C(1)	1.865(3)
P(2)-C(18)	1.838(3)
P(2)-C(22)	1.862(3)
P(2)-C(19)	1.877(4)
C(42)-C(41)	1.379(5)
C(42)-C(43)	1.383(5)
C(42)-H(42)	0.9500
C(18)-C(17)	1.522(4)
C(18)-H(18A)	0.9900
C(18)-H(18B)	0.9900
C(38)-C(45)	1.510(5)
C(38)-C(37)	1.537(4)
C(38)-H(38)	1.0000
C(35)-C(39)	1.513(4)
C(35)-C(36)	1.550(4)
C(35)-H(35)	1.0000
C(29)-C(34)	1.396(5)

C(29)-C(30)	1.403(5)
C(29)-C(22)	1.509(5)
C(34)-C(33)	1.386(5)
C(34)-H(34)	0.9500
C(26)-C(27)	1.370(5)
C(26)-C(25)	1.393(5)
C(26)-H(26)	0.9500
C(17)-H(17A)	0.9900
C(17)-H(17B)	0.9900
C(4)-C(11)	1.505(4)
C(4)-C(3)	1.538(4)
C(4)-H(4)	1.0000
C(16)-C(15)	1.386(4)
C(16)-C(11)	1.391(4)
C(16)-H(16)	0.9500
C(9)-C(8)	1.387(5)
C(9)-C(10)	1.388(4)
C(9)-H(9)	0.9500
C(57)-C(62)	1.380(5)
C(57)-C(58)	1.391(4)
C(57)-C(53)	1.504(4)
C(47)-C(46)	1.387(4)
C(47)-C(48)	1.396(5)
C(47)-H(47)	0.9500
C(27)-C(28)	1.399(5)
C(27)-H(27)	0.9500
C(10)-C(5)	1.388(4)
C(10)-H(10)	0.9500
C(2)-C(1)	1.544(4)
C(2)-C(3)	1.550(4)
C(2)-H(2A)	0.9900
C(2)-H(2B)	0.9900
C(50)-C(45)	1.384(5)
C(50)-C(49)	1.392(5)
C(50)-H(50)	0.9500

C(22)-C(21)	1.519(4)
C(22)-H(22)	1.0000
C(53)-C(54)	1.534(4)
C(53)-H(53)	1.0000
C(8)-C(7)	1.390(5)
C(8)-H(8)	0.9500
C(67)-C(66)	1.375(5)
C(67)-C(68)	1.392(4)
C(67)-H(67)	0.9500
C(45)-C(46)	1.389(5)
C(30)-C(31)	1.391(5)
C(30)-H(30)	0.9500
C(52)-C(51)	1.520(4)
C(52)-H(52A)	0.9900
C(52)-H(52B)	0.9900
C(11)-C(12)	1.394(4)
C(31)-C(32)	1.374(5)
C(31)-H(31)	0.9500
C(39)-C(40)	1.387(4)
C(39)-C(44)	1.400(4)
C(48)-C(49)	1.374(5)
C(48)-H(48)	0.9500
C(32)-C(33)	1.385(5)
C(32)-H(32)	0.9500
C(60)-C(61)	1.373(5)
C(60)-C(59)	1.378(6)
C(60)-H(60)	0.9500
C(49)-H(49)	0.9500
C(19)-C(23)	1.520(4)
C(19)-C(20)	1.551(5)
C(19)-H(19)	1.0000
C(6)-C(7)	1.383(5)
C(6)-C(5)	1.392(4)
C(6)-H(6)	0.9500
C(41)-C(40)	1.395(4)

C(41)-H(41)	0.9500
C(13)-C(14)	1.385(5)
C(13)-C(12)	1.388(4)
C(13)-H(13)	0.9500
C(59)-C(58)	1.386(5)
C(59)-H(59)	0.9500
C(65)-C(64)	1.376(4)
C(65)-C(66)	1.394(5)
C(65)-H(65)	0.9500
C(20)-C(21)	1.522(4)
C(20)-H(20A)	0.9900
C(20)-H(20B)	0.9900
C(44)-C(43)	1.392(4)
C(44)-H(44)	0.9500
C(58)-H(58)	0.9500
C(62)-C(61)	1.396(4)
C(62)-H(62)	0.9500
C(33)-H(33)	0.9500
C(24)-C(25)	1.381(5)
C(24)-C(23)	1.397(4)
C(24)-H(24)	0.9500
C(46)-H(46)	0.9500
C(64)-C(63)	1.402(4)
C(64)-H(64)	0.9500
C(21)-H(21A)	0.9900
C(21)-H(21B)	0.9900
C(1)-C(5)	1.516(4)
C(1)-H(1)	1.0000
C(12)-H(12)	0.9500
C(36)-C(37)	1.553(4)
C(36)-H(36A)	0.9900
C(36)-H(36B)	0.9900
C(7)-H(7)	0.9500
C(3)-H(3A)	0.9900
C(3)-H(3B)	0.9900

C(28)-C(23)	1.388(4)
C(28)-H(28)	0.9500
C(61)-H(61)	0.9500
C(51)-H(51A)	0.9900
C(51)-H(51B)	0.9900
C(14)-C(15)	1.379(5)
C(14)-H(14)	0.9500
C(68)-C(63)	1.393(4)
C(68)-H(68)	0.9500
C(55)-C(54)	1.526(4)
C(55)-C(56)	1.540(4)
C(55)-H(55A)	0.9900
C(55)-H(55B)	0.9900
C(66)-H(66)	0.9500
C(37)-H(37A)	0.9900
C(37)-H(37B)	0.9900
C(25)-H(25)	0.9500
C(15)-H(15)	0.9500
C(40)-H(40)	0.9500
C(43)-H(43)	0.9500
C(63)-C(56)	1.503(4)
C(56)-H(56)	1.0000
C(54)-H(54A)	0.9900
C(54)-H(54B)	0.9900
P(3)-Rh(01)-P(4)	85.16(4)
P(3)-Rh(01)-Cl(1)	176.24(3)
P(4)-Rh(01)-Cl(1)	91.58(3)
P(3)-Rh(01)-Cl(2)	98.51(3)
P(4)-Rh(01)-Cl(2)	176.11(3)
Cl(1)-Rh(01)-Cl(2)	84.79(3)
P(2)-Rh(02)-P(1)	85.45(4)
P(2)-Rh(02)-Cl(1)	93.21(3)
P(1)-Rh(02)-Cl(1)	176.85(4)
P(2)-Rh(02)-Cl(2)	173.64(3)
P(1)-Rh(02)-Cl(2)	96.49(3)

Cl(1)-Rh(02)-Cl(2)	85.15(3)
Rh(02)-Cl(1)-Rh(01)	95.33(3)
C(51)-P(3)-C(38)	100.65(16)
C(51)-P(3)-C(35)	111.47(16)
C(38)-P(3)-C(35)	90.59(15)
C(51)-P(3)-Rh(01)	112.07(12)
C(38)-P(3)-Rh(01)	126.56(12)
C(35)-P(3)-Rh(01)	113.33(12)
C(52)-P(4)-C(56)	109.19(14)
C(52)-P(4)-C(53)	101.34(15)
C(56)-P(4)-C(53)	93.01(14)
C(52)-P(4)-Rh(01)	114.01(11)
C(56)-P(4)-Rh(01)	114.11(9)
C(53)-P(4)-Rh(01)	122.66(11)
C(17)-P(1)-C(4)	105.23(16)
C(17)-P(1)-C(1)	102.98(15)
C(4)-P(1)-C(1)	92.33(13)
C(17)-P(1)-Rh(02)	111.41(11)
C(4)-P(1)-Rh(02)	121.05(11)
C(1)-P(1)-Rh(02)	120.88(10)
C(18)-P(2)-C(22)	102.54(16)
C(18)-P(2)-C(19)	104.25(16)
C(22)-P(2)-C(19)	91.84(17)
C(18)-P(2)-Rh(02)	112.55(12)
C(22)-P(2)-Rh(02)	124.19(12)
C(19)-P(2)-Rh(02)	118.06(12)
Rh(02)-Cl(2)-Rh(01)	94.68(3)
C(41)-C(42)-C(43)	119.7(3)
C(41)-C(42)-H(42)	120.2
C(43)-C(42)-H(42)	120.2
C(17)-C(18)-P(2)	108.2(2)
C(17)-C(18)-H(18A)	110.1
P(2)-C(18)-H(18A)	110.1
C(17)-C(18)-H(18B)	110.1
P(2)-C(18)-H(18B)	110.1

H(18A)-C(18)-H(18B)	108.4
C(45)-C(38)-C(37)	117.4(3)
C(45)-C(38)-P(3)	116.2(2)
C(37)-C(38)-P(3)	104.0(2)
C(45)-C(38)-H(38)	106.1
C(37)-C(38)-H(38)	106.1
P(3)-C(38)-H(38)	106.1
C(39)-C(35)-C(36)	117.0(3)
C(39)-C(35)-P(3)	114.7(2)
C(36)-C(35)-P(3)	100.7(2)
C(39)-C(35)-H(35)	108.0
C(36)-C(35)-H(35)	108.0
P(3)-C(35)-H(35)	108.0
C(34)-C(29)-C(30)	118.2(3)
C(34)-C(29)-C(22)	119.9(3)
C(30)-C(29)-C(22)	121.8(3)
C(33)-C(34)-C(29)	120.8(3)
C(33)-C(34)-H(34)	119.6
C(29)-C(34)-H(34)	119.6
C(27)-C(26)-C(25)	118.7(3)
C(27)-C(26)-H(26)	120.6
C(25)-C(26)-H(26)	120.6
C(18)-C(17)-P(1)	108.5(2)
C(18)-C(17)-H(17A)	110.0
P(1)-C(17)-H(17A)	110.0
C(18)-C(17)-H(17B)	110.0
P(1)-C(17)-H(17B)	110.0
H(17A)-C(17)-H(17B)	108.4
C(11)-C(4)-C(3)	117.7(3)
C(11)-C(4)-P(1)	112.8(2)
C(3)-C(4)-P(1)	106.5(2)
C(11)-C(4)-H(4)	106.4
C(3)-C(4)-H(4)	106.4
P(1)-C(4)-H(4)	106.4
C(15)-C(16)-C(11)	120.8(3)

C(15)-C(16)-H(16)	119.6
C(11)-C(16)-H(16)	119.6
C(8)-C(9)-C(10)	120.1(3)
C(8)-C(9)-H(9)	120.0
C(10)-C(9)-H(9)	120.0
C(62)-C(57)-C(58)	118.4(3)
C(62)-C(57)-C(53)	122.1(3)
C(58)-C(57)-C(53)	119.4(3)
C(46)-C(47)-C(48)	120.2(3)
C(46)-C(47)-H(47)	119.9
C(48)-C(47)-H(47)	119.9
C(26)-C(27)-C(28)	120.9(4)
C(26)-C(27)-H(27)	119.5
C(28)-C(27)-H(27)	119.5
C(5)-C(10)-C(9)	120.9(3)
C(5)-C(10)-H(10)	119.6
C(9)-C(10)-H(10)	119.6
C(1)-C(2)-C(3)	111.5(2)
C(1)-C(2)-H(2A)	109.3
C(3)-C(2)-H(2A)	109.3
C(1)-C(2)-H(2B)	109.3
C(3)-C(2)-H(2B)	109.3
H(2A)-C(2)-H(2B)	108.0
C(45)-C(50)-C(49)	121.7(3)
C(45)-C(50)-H(50)	119.2
C(49)-C(50)-H(50)	119.2
C(29)-C(22)-C(21)	118.4(3)
C(29)-C(22)-P(2)	114.3(2)
C(21)-C(22)-P(2)	102.7(2)
C(29)-C(22)-H(22)	106.9
C(21)-C(22)-H(22)	106.9
P(2)-C(22)-H(22)	106.9
C(57)-C(53)-C(54)	119.5(3)
C(57)-C(53)-P(4)	114.3(2)
C(54)-C(53)-P(4)	104.2(2)

C(57)-C(53)-H(53)	105.9
C(54)-C(53)-H(53)	105.9
P(4)-C(53)-H(53)	105.9
C(9)-C(8)-C(7)	119.7(3)
C(9)-C(8)-H(8)	120.2
C(7)-C(8)-H(8)	120.2
C(66)-C(67)-C(68)	120.3(3)
C(66)-C(67)-H(67)	119.8
C(68)-C(67)-H(67)	119.8
C(50)-C(45)-C(46)	118.1(3)
C(50)-C(45)-C(38)	120.4(3)
C(46)-C(45)-C(38)	121.5(3)
C(31)-C(30)-C(29)	120.3(4)
C(31)-C(30)-H(30)	119.8
C(29)-C(30)-H(30)	119.8
C(51)-C(52)-P(4)	109.5(2)
C(51)-C(52)-H(52A)	109.8
P(4)-C(52)-H(52A)	109.8
C(51)-C(52)-H(52B)	109.8
P(4)-C(52)-H(52B)	109.8
H(52A)-C(52)-H(52B)	108.2
C(16)-C(11)-C(12)	118.2(3)
C(16)-C(11)-C(4)	119.6(3)
C(12)-C(11)-C(4)	122.1(3)
C(32)-C(31)-C(30)	120.7(3)
C(32)-C(31)-H(31)	119.6
C(30)-C(31)-H(31)	119.6
C(40)-C(39)-C(44)	118.2(3)
C(40)-C(39)-C(35)	123.5(3)
C(44)-C(39)-C(35)	118.3(3)
C(49)-C(48)-C(47)	119.5(3)
C(49)-C(48)-H(48)	120.3
C(47)-C(48)-H(48)	120.3
C(31)-C(32)-C(33)	119.6(3)
C(31)-C(32)-H(32)	120.2

C(33)-C(32)-H(32)	120.2
C(61)-C(60)-C(59)	119.4(3)
C(61)-C(60)-H(60)	120.3
C(59)-C(60)-H(60)	120.3
C(48)-C(49)-C(50)	119.8(3)
C(48)-C(49)-H(49)	120.1
C(50)-C(49)-H(49)	120.1
C(23)-C(19)-C(20)	116.8(3)
C(23)-C(19)-P(2)	111.1(2)
C(20)-C(19)-P(2)	106.2(2)
C(23)-C(19)-H(19)	107.4
C(20)-C(19)-H(19)	107.4
P(2)-C(19)-H(19)	107.4
C(7)-C(6)-C(5)	121.2(3)
C(7)-C(6)-H(6)	119.4
C(5)-C(6)-H(6)	119.4
C(42)-C(41)-C(40)	120.3(3)
C(42)-C(41)-H(41)	119.8
C(40)-C(41)-H(41)	119.8
C(14)-C(13)-C(12)	120.1(3)
C(14)-C(13)-H(13)	119.9
C(12)-C(13)-H(13)	119.9
C(60)-C(59)-C(58)	120.4(3)
C(60)-C(59)-H(59)	119.8
C(58)-C(59)-H(59)	119.8
C(64)-C(65)-C(66)	119.9(3)
C(64)-C(65)-H(65)	120.0
C(66)-C(65)-H(65)	120.0
C(21)-C(20)-C(19)	110.8(3)
C(21)-C(20)-H(20A)	109.5
C(19)-C(20)-H(20A)	109.5
C(21)-C(20)-H(20B)	109.5
C(19)-C(20)-H(20B)	109.5
H(20A)-C(20)-H(20B)	108.1
C(43)-C(44)-C(39)	120.8(3)

C(43)-C(44)-H(44)	119.6
C(39)-C(44)-H(44)	119.6
C(59)-C(58)-C(57)	120.7(3)
C(59)-C(58)-H(58)	119.7
C(57)-C(58)-H(58)	119.7
C(57)-C(62)-C(61)	120.6(3)
C(57)-C(62)-H(62)	119.7
C(61)-C(62)-H(62)	119.7
C(32)-C(33)-C(34)	120.4(3)
C(32)-C(33)-H(33)	119.8
C(34)-C(33)-H(33)	119.8
C(25)-C(24)-C(23)	121.3(3)
C(25)-C(24)-H(24)	119.3
C(23)-C(24)-H(24)	119.3
C(47)-C(46)-C(45)	120.8(3)
C(47)-C(46)-H(46)	119.6
C(45)-C(46)-H(46)	119.6
C(65)-C(64)-C(63)	121.3(3)
C(65)-C(64)-H(64)	119.3
C(63)-C(64)-H(64)	119.3
C(22)-C(21)-C(20)	107.7(3)
C(22)-C(21)-H(21A)	110.2
C(20)-C(21)-H(21A)	110.2
C(22)-C(21)-H(21B)	110.2
C(20)-C(21)-H(21B)	110.2
H(21A)-C(21)-H(21B)	108.5
C(5)-C(1)-C(2)	116.4(2)
C(5)-C(1)-P(1)	109.07(19)
C(2)-C(1)-P(1)	104.72(19)
C(5)-C(1)-H(1)	108.8
C(2)-C(1)-H(1)	108.8
P(1)-C(1)-H(1)	108.8
C(13)-C(12)-C(11)	120.9(3)
C(13)-C(12)-H(12)	119.6
C(11)-C(12)-H(12)	119.6

C(35)-C(36)-C(37)	111.7(2)
C(35)-C(36)-H(36A)	109.3
C(37)-C(36)-H(36A)	109.3
C(35)-C(36)-H(36B)	109.3
C(37)-C(36)-H(36B)	109.3
H(36A)-C(36)-H(36B)	107.9
C(6)-C(7)-C(8)	119.8(3)
C(6)-C(7)-H(7)	120.1
C(8)-C(7)-H(7)	120.1
C(4)-C(3)-C(2)	111.4(2)
C(4)-C(3)-H(3A)	109.3
C(2)-C(3)-H(3A)	109.3
C(4)-C(3)-H(3B)	109.3
C(2)-C(3)-H(3B)	109.3
H(3A)-C(3)-H(3B)	108.0
C(23)-C(28)-C(27)	120.9(3)
C(23)-C(28)-H(28)	119.5
C(27)-C(28)-H(28)	119.5
C(60)-C(61)-C(62)	120.4(3)
C(60)-C(61)-H(61)	119.8
C(62)-C(61)-H(61)	119.8
C(52)-C(51)-P(3)	111.0(2)
C(52)-C(51)-H(51A)	109.4
P(3)-C(51)-H(51A)	109.4
C(52)-C(51)-H(51B)	109.4
P(3)-C(51)-H(51B)	109.4
H(51A)-C(51)-H(51B)	108.0
C(15)-C(14)-C(13)	119.5(3)
C(15)-C(14)-H(14)	120.3
C(13)-C(14)-H(14)	120.3
C(67)-C(68)-C(63)	120.8(3)
C(67)-C(68)-H(68)	119.6
C(63)-C(68)-H(68)	119.6
C(28)-C(23)-C(24)	117.5(3)
C(28)-C(23)-C(19)	123.3(3)

C(24)-C(23)-C(19)	119.1(3)
C(54)-C(55)-C(56)	107.4(2)
C(54)-C(55)-H(55A)	110.2
C(56)-C(55)-H(55A)	110.2
C(54)-C(55)-H(55B)	110.2
C(56)-C(55)-H(55B)	110.2
H(55A)-C(55)-H(55B)	108.5
C(67)-C(66)-C(65)	119.7(3)
C(67)-C(66)-H(66)	120.1
C(65)-C(66)-H(66)	120.1
C(38)-C(37)-C(36)	109.6(2)
C(38)-C(37)-H(37A)	109.8
C(36)-C(37)-H(37A)	109.8
C(38)-C(37)-H(37B)	109.8
C(36)-C(37)-H(37B)	109.8
H(37A)-C(37)-H(37B)	108.2
C(10)-C(5)-C(6)	118.4(3)
C(10)-C(5)-C(1)	122.4(3)
C(6)-C(5)-C(1)	119.1(3)
C(24)-C(25)-C(26)	120.6(3)
C(24)-C(25)-H(25)	119.7
C(26)-C(25)-H(25)	119.7
C(14)-C(15)-C(16)	120.5(3)
C(14)-C(15)-H(15)	119.8
C(16)-C(15)-H(15)	119.8
C(39)-C(40)-C(41)	120.8(3)
C(39)-C(40)-H(40)	119.6
C(41)-C(40)-H(40)	119.6
C(42)-C(43)-C(44)	120.1(3)
C(42)-C(43)-H(43)	120.0
C(44)-C(43)-H(43)	120.0
C(68)-C(63)-C(64)	117.9(3)
C(68)-C(63)-C(56)	122.5(3)
C(64)-C(63)-C(56)	119.6(2)
C(63)-C(56)-C(55)	118.0(2)

C(63)-C(56)-P(4)	113.9(2)
C(55)-C(56)-P(4)	105.67(19)
C(63)-C(56)-H(56)	106.2
C(55)-C(56)-H(56)	106.2
P(4)-C(56)-H(56)	106.2
C(55)-C(54)-C(53)	104.9(2)
C(55)-C(54)-H(54A)	110.8
C(53)-C(54)-H(54A)	110.8
C(55)-C(54)-H(54B)	110.8
C(53)-C(54)-H(54B)	110.8
H(54A)-C(54)-H(54B)	108.8

Symmetry transformations used to generate equivalent atoms:

Table S11. Anisotropic displacement parameters ($Å^2 x 10^3$) for **2**. The anisotropic displacement factor exponent takes the form: $-2\pi^2[h^2 a^{*2}U^{11} + ... + 2 h k a^* b^* U^{12}]$.

	U^{11}	U^{22}	U^{33}	U^{23}	U^{13}	U^{12}
Rh(01)	11(1)	13(1)	9(1)	5(1)	5(1)	6(1)
Rh(02)	10(1)	13(1)	9(1)	5(1)	5(1)	6(1)
Cl(1)	15(1)	21(1)	15(1)	10(1)	9(1)	12(1)
P(3)	11(1)	13(1)	10(1)	6(1)	4(1)	6(1)
P(4)	11(1)	15(1)	11(1)	6(1)	6(1)	7(1)
P(1)	11(1)	15(1)	10(1)	4(1)	5(1)	6(1)
P(2)	12(1)	15(1)	12(1)	7(1)	6(1)	7(1)
Cl(2)	18(1)	24(1)	13(1)	12(1)	10(1)	14(1)
C(42)	41(2)	16(2)	18(2)	9(2)	14(2)	11(2)
C(18)	9(1)	23(2)	17(2)	9(1)	5(1)	6(1)
C(38)	13(1)	20(2)	17(2)	9(1)	7(1)	10(1)
C(35)	16(2)	16(2)	11(2)	5(1)	4(1)	7(1)
C(29)	12(2)	24(2)	19(2)	11(2)	8(1)	10(1)
C(34)	16(2)	26(2)	21(2)	9(1)	4(1)	11(1)
C(26)	38(2)	23(2)	18(2)	3(1)	1(2)	7(2)
C(17)	20(2)	23(2)	16(2)	11(1)	12(1)	11(1)
C(4)	13(1)	19(2)	13(2)	5(1)	4(1)	8(1)
C(16)	15(1)	19(1)	19(2)	8(1)	5(1)	8(1)
C(9)	30(2)	21(2)	20(2)	2(1)	1(1)	15(1)

C(57)	10(1)	20(2)	24(2)	11(1)	3(1)	6(1)
C(47)	18(1)	21(1)	28(2)	13(1)	10(1)	9(1)
C(27)	36(2)	20(2)	22(2)	4(1)	11(2)	14(2)
C(10)	19(1)	17(1)	21(2)	1(1)	5(1)	9(1)
C(2)	18(1)	25(2)	20(2)	4(1)	12(1)	9(1)
C(50)	19(2)	26(2)	21(2)	14(1)	6(1)	10(1)
C(22)	12(1)	24(2)	15(2)	11(1)	6(1)	10(1)
C(53)	12(1)	30(2)	16(2)	12(1)	9(1)	12(1)
C(8)	37(2)	15(2)	24(2)	7(1)	11(2)	9(2)
C(67)	34(2)	21(2)	18(2)	5(1)	10(2)	14(1)
C(45)	8(2)	20(2)	18(2)	5(1)	3(1)	6(1)
C(30)	18(2)	30(2)	22(2)	12(2)	4(1)	9(1)
C(52)	9(1)	22(2)	13(2)	8(1)	4(1)	9(1)
C(11)	9(1)	18(1)	18(2)	7(1)	1(1)	6(1)
C(31)	20(2)	41(2)	17(2)	5(2)	-1(1)	12(2)
C(39)	18(1)	11(1)	14(1)	4(1)	6(1)	5(1)
C(48)	15(2)	18(1)	35(2)	6(1)	10(2)	7(1)
C(32)	16(2)	29(2)	26(2)	5(2)	4(1)	7(1)
C(60)	25(2)	19(2)	44(2)	3(2)	-3(2)	8(1)
C(49)	21(2)	28(2)	18(2)	3(2)	0(1)	8(2)
C(19)	25(2)	14(2)	20(2)	10(1)	10(2)	8(1)
C(6)	17(1)	16(1)	25(2)	6(1)	6(1)	8(1)
C(41)	29(2)	16(1)	16(2)	5(1)	2(1)	9(1)
C(13)	16(1)	25(2)	34(2)	17(1)	11(1)	8(1)
C(59)	31(2)	24(2)	57(3)	22(2)	3(2)	8(2)
C(65)	20(2)	25(2)	28(2)	5(1)	5(1)	10(1)
C(20)	43(2)	26(2)	22(2)	14(1)	9(2)	21(2)
C(44)	19(1)	16(1)	23(2)	6(1)	6(1)	6(1)
C(58)	21(2)	31(2)	34(2)	21(2)	6(1)	8(1)
C(62)	15(1)	20(1)	24(2)	9(1)	3(1)	3(1)
C(33)	22(2)	24(2)	28(2)	10(1)	9(2)	11(1)
C(24)	19(1)	19(1)	26(2)	8(1)	5(1)	6(1)
C(46)	17(2)	19(2)	22(2)	9(1)	8(1)	8(1)
C(64)	21(2)	22(1)	23(2)	4(1)	8(1)	13(1)
C(21)	22(1)	27(2)	21(2)	13(1)	9(1)	17(1)

C(1)	12(1)	19(1)	12(1)	2(1)	4(1)	7(1)
C(12)	14(1)	25(1)	18(2)	9(1)	6(1)	9(1)
C(36)	28(2)	19(1)	20(2)	10(1)	14(1)	15(1)
C(7)	20(2)	20(2)	33(2)	9(2)	10(2)	5(1)
C(3)	14(1)	23(1)	24(2)	5(1)	9(1)	9(1)
C(28)	22(2)	22(1)	22(2)	8(1)	6(1)	11(1)
C(61)	22(2)	25(2)	30(2)	3(1)	4(1)	9(1)
C(51)	21(2)	18(2)	5(1)	4(1)	4(1)	10(1)
C(14)	18(2)	16(1)	33(2)	5(1)	2(1)	5(1)
C(68)	22(1)	21(1)	20(2)	10(1)	10(1)	12(1)
C(23)	18(1)	11(1)	18(2)	5(1)	5(1)	4(1)
C(55)	20(1)	26(2)	20(2)	7(1)	7(1)	16(1)
C(66)	28(2)	21(2)	18(2)	0(2)	1(2)	7(2)
C(37)	20(1)	24(1)	23(2)	12(1)	13(1)	15(1)
C(5)	18(1)	13(1)	15(2)	-2(1)	4(1)	6(1)
C(25)	23(2)	29(2)	28(2)	10(2)	-4(1)	6(1)
C(15)	22(2)	23(2)	20(2)	2(1)	0(1)	9(1)
C(40)	20(1)	16(1)	17(2)	5(1)	6(1)	7(1)
C(43)	25(2)	17(1)	28(2)	7(1)	15(1)	5(1)
C(63)	18(1)	14(1)	17(2)	7(1)	7(1)	9(1)
C(56)	17(1)	19(1)	15(2)	7(1)	8(1)	11(1)
C(54)	15(1)	26(2)	17(2)	9(1)	7(1)	13(1)

Table S12. Hydrogen coordinates (x 10^4) and isotropic displacement parameters (Å^2 x 10^3) for **2**.

	y	z	U(eq)	
H(42)	3156	400	13599	30
H(18A)	4043	4786	4819	20
H(18B)	4857	6253	5144	20
H(38)	7076	4441	12888	18
H(35)	3724	2186	10267	18
H(34)	1538	3387	6336	25
H(26)	6347	9439	11641	38
H(17A)	7098	6516	6167	21
H(17B)	6480	5204	5122	21
H(4)	8316	4955	7991	18
H(16)	9103	6834	9520	22
H(9)	6451	1245	8031	30
H(47)	9638	8539	12299	26
H(27)	4135	9245	10576	33
H(10)	7284	2466	7074	25
H(2A)	7156	3494	4826	26
H(2B)	7329	2620	5427	26
H(50)	8788	6208	14406	26
H(22)	1783	5593	6641	18
H(53)	399	4281	11015	21
H(8)	3967	468	7950	32
H(67)	4308	9512	15164	29
H(30)	579	4380	3786	29
H(52A)	3026	5436	12997	17
H(52B)	2234	3983	12180	17
H(31)	-927	2284	2568	36
H(48)	10939	9493	14246	29
H(32)	-1188	747	3214	33
H(60)	-1215	-116	7332	42
H(49)	10487	8322	15296	32
H(19)	5615	8019	7024	23

H(6)	3155	2117	5930	24
H(41)	5470	870	13385	27
H(13)	11203	9106	7588	29
H(59)	-1320	192	9121	48
H(65)	7624	8955	14308	32
H(20A)	2926	8013	7048	34
H(20B)	3799	8313	6219	34
H(44)	2204	1928	11497	25
H(58)	-457	2192	10477	34
H(62)	652	3601	8263	26
H(33)	51	1302	5100	30
H(24)	7130	8065	8784	28
H(46)	7954	6423	11419	23
H(64)	5961	7569	12507	27
H(21A)	1274	6425	5384	24
H(21B)	2565	6308	4923	24
H(1)	4989	2972	5113	19
H(12)	9772	6950	6660	23
H(36A)	5786	2592	9832	24
H(36B)	6100	1916	10612	24
H(7)	2311	895	6880	31
H(3A)	9311	4214	6824	25
H(3B)	9234	5054	6158	25
H(28)	3379	8429	8618	27
H(61)	-258	1585	6898	35
H(51A)	4436	4182	13087	18
H(51B)	5323	5613	13310	18
H(14)	11677	10119	9506	32
H(68)	2609	8065	13370	23
H(55A)	1043	6494	11835	25
H(55B)	1401	7282	11098	25
H(66)	6800	9941	15646	32
H(37A)	8159	3638	11801	23
H(37B)	7916	4276	10965	23
H(25)	7852	8849	10726	38

H(15)	10621	8974	10461	30
H(40)	6190	1919	12269	22
H(43)	1545	972	12688	29
H(56)	3503	7117	11057	19
H(54A)	833	5477	9519	22
H(54B)	-557	5139	9957	22

Table S13. Torsion angles [°] for **2**.

C(22)-P(2)-C(18)-C(17)	-167.1(2)
C(19)-P(2)-C(18)-C(17)	97.7(3)
Rh(02)-P(2)-C(18)-C(17)	-31.4(3)
C(51)-P(3)-C(38)-C(45)	-75.3(3)
C(35)-P(3)-C(38)-C(45)	172.7(3)
Rh(01)-P(3)-C(38)-C(45)	52.6(3)
C(51)-P(3)-C(38)-C(37)	154.0(2)
C(35)-P(3)-C(38)-C(37)	42.0(2)
Rh(01)-P(3)-C(38)-C(37)	-78.1(2)
C(51)-P(3)-C(35)-C(39)	-18.3(3)
C(38)-P(3)-C(35)-C(39)	83.4(3)
Rh(01)-P(3)-C(35)-C(39)	-145.8(2)
C(51)-P(3)-C(35)-C(36)	-144.8(2)
C(38)-P(3)-C(35)-C(36)	-43.1(2)
Rh(01)-P(3)-C(35)-C(36)	87.6(2)
C(30)-C(29)-C(34)-C(33)	0.6(5)
C(22)-C(29)-C(34)-C(33)	179.0(3)
P(2)-C(18)-C(17)-P(1)	39.3(3)
C(4)-P(1)-C(17)-C(18)	-167.3(2)
C(1)-P(1)-C(17)-C(18)	96.6(2)
Rh(02)-P(1)-C(17)-C(18)	-34.4(2)
C(17)-P(1)-C(4)-C(11)	57.7(3)
C(1)-P(1)-C(4)-C(11)	161.8(2)
Rh(02)-P(1)-C(4)-C(11)	-69.6(3)
C(17)-P(1)-C(4)-C(3)	-72.9(2)
C(1)-P(1)-C(4)-C(3)	31.2(2)
Rh(02)-P(1)-C(4)-C(3)	159.85(17)
C(25)-C(26)-C(27)-C(28)	0.7(6)
C(8)-C(9)-C(10)-C(5)	-0.2(4)
C(34)-C(29)-C(22)-C(21)	-175.6(3)
C(30)-C(29)-C(22)-C(21)	2.8(5)
C(34)-C(29)-C(22)-P(2)	63.2(4)
C(30)-C(29)-C(22)-P(2)	-118.5(3)
C(18)-P(2)-C(22)-C(29)	62.0(3)

C(19)-P(2)-C(22)-C(29)	167.0(3)
Rh(02)-P(2)-C(22)-C(29)	-66.7(3)
C(18)-P(2)-C(22)-C(21)	-67.5(2)
C(19)-P(2)-C(22)-C(21)	37.5(2)
Rh(02)-P(2)-C(22)-C(21)	163.72(15)
C(62)-C(57)-C(53)-C(54)	44.2(4)
C(58)-C(57)-C(53)-C(54)	-138.2(3)
C(62)-C(57)-C(53)-P(4)	-80.3(3)
C(58)-C(57)-C(53)-P(4)	97.3(3)
C(52)-P(4)-C(53)-C(57)	-95.1(2)
C(56)-P(4)-C(53)-C(57)	154.7(2)
Rh(01)-P(4)-C(53)-C(57)	33.4(3)
C(52)-P(4)-C(53)-C(54)	132.7(2)
C(56)-P(4)-C(53)-C(54)	22.4(2)
Rh(01)-P(4)-C(53)-C(54)	-98.90(19)
C(10)-C(9)-C(8)-C(7)	0.5(5)
C(49)-C(50)-C(45)-C(46)	-1.0(5)
C(49)-C(50)-C(45)-C(38)	-179.2(3)
C(37)-C(38)-C(45)-C(50)	-117.1(4)
P(3)-C(38)-C(45)-C(50)	118.9(3)
C(37)-C(38)-C(45)-C(46)	64.8(4)
P(3)-C(38)-C(45)-C(46)	-59.2(4)
C(34)-C(29)-C(30)-C(31)	-0.3(5)
C(22)-C(29)-C(30)-C(31)	-178.6(3)
C(56)-P(4)-C(52)-C(51)	-110.5(2)
C(53)-P(4)-C(52)-C(51)	152.2(2)
Rh(01)-P(4)-C(52)-C(51)	18.4(3)
C(15)-C(16)-C(11)-C(12)	-1.1(4)
C(15)-C(16)-C(11)-C(4)	-178.4(3)
C(3)-C(4)-C(11)-C(16)	-145.2(3)
P(1)-C(4)-C(11)-C(16)	90.1(3)
C(3)-C(4)-C(11)-C(12)	37.5(4)
P(1)-C(4)-C(11)-C(12)	-87.1(3)
C(29)-C(30)-C(31)-C(32)	-0.2(6)
C(36)-C(35)-C(39)-C(40)	12.7(4)

P(3)-C(35)-C(39)-C(40)	-105.0(3)
C(36)-C(35)-C(39)-C(44)	-165.4(3)
P(3)-C(35)-C(39)-C(44)	76.9(3)
C(46)-C(47)-C(48)-C(49)	-0.9(5)
C(30)-C(31)-C(32)-C(33)	0.3(6)
C(47)-C(48)-C(49)-C(50)	0.7(5)
C(45)-C(50)-C(49)-C(48)	0.3(6)
C(18)-P(2)-C(19)-C(23)	-146.7(2)
C(22)-P(2)-C(19)-C(23)	109.9(3)
Rh(02)-P(2)-C(19)-C(23)	-21.0(3)
C(18)-P(2)-C(19)-C(20)	85.3(3)
C(22)-P(2)-C(19)-C(20)	-18.2(2)
Rh(02)-P(2)-C(19)-C(20)	-149.02(18)
C(43)-C(42)-C(41)-C(40)	0.2(5)
C(61)-C(60)-C(59)-C(58)	-0.2(6)
C(23)-C(19)-C(20)-C(21)	-131.0(3)
P(2)-C(19)-C(20)-C(21)	-6.5(3)
C(40)-C(39)-C(44)-C(43)	0.4(4)
C(35)-C(39)-C(44)-C(43)	178.6(3)
C(60)-C(59)-C(58)-C(57)	-0.7(5)
C(62)-C(57)-C(58)-C(59)	1.0(5)
C(53)-C(57)-C(58)-C(59)	-176.7(3)
C(58)-C(57)-C(62)-C(61)	-0.4(5)
C(53)-C(57)-C(62)-C(61)	177.2(3)
C(31)-C(32)-C(33)-C(34)	0.1(5)
C(29)-C(34)-C(33)-C(32)	-0.6(5)
C(48)-C(47)-C(46)-C(45)	0.2(5)
C(50)-C(45)-C(46)-C(47)	0.8(5)
C(38)-C(45)-C(46)-C(47)	178.9(3)
C(66)-C(65)-C(64)-C(63)	-0.9(5)
C(29)-C(22)-C(21)-C(20)	-174.3(3)
P(2)-C(22)-C(21)-C(20)	-47.3(3)
C(19)-C(20)-C(21)-C(22)	35.6(3)
C(3)-C(2)-C(1)-C(5)	-92.9(3)
C(3)-C(2)-C(1)-P(1)	27.6(3)

C(17)-P(1)-C(1)-C(5)	-162.4(2)
C(4)-P(1)-C(1)-C(5)	91.4(2)
Rh(02)-P(1)-C(1)-C(5)	-37.3(2)
C(17)-P(1)-C(1)-C(2)	72.4(2)
C(4)-P(1)-C(1)-C(2)	-33.8(2)
Rh(02)-P(1)-C(1)-C(2)	-162.59(16)
C(14)-C(13)-C(12)-C(11)	1.8(4)
C(16)-C(11)-C(12)-C(13)	-0.5(4)
C(4)-C(11)-C(12)-C(13)	176.8(3)
C(39)-C(35)-C(36)-C(37)	-91.4(3)
P(3)-C(35)-C(36)-C(37)	33.6(3)
C(5)-C(6)-C(7)-C(8)	0.3(5)
C(9)-C(8)-C(7)-C(6)	-0.5(5)
C(11)-C(4)-C(3)-C(2)	-147.4(3)
P(1)-C(4)-C(3)-C(2)	-19.7(3)
C(1)-C(2)-C(3)-C(4)	-5.5(4)
C(26)-C(27)-C(28)-C(23)	-1.1(5)
C(59)-C(60)-C(61)-C(62)	0.8(5)
C(57)-C(62)-C(61)-C(60)	-0.5(5)
P(4)-C(52)-C(51)-P(3)	-29.3(3)
C(38)-P(3)-C(51)-C(52)	167.6(2)
C(35)-P(3)-C(51)-C(52)	-97.6(3)
Rh(01)-P(3)-C(51)-C(52)	30.7(3)
C(12)-C(13)-C(14)-C(15)	-1.5(5)
C(66)-C(67)-C(68)-C(63)	-1.5(5)
C(27)-C(28)-C(23)-C(24)	0.8(4)
C(27)-C(28)-C(23)-C(19)	177.8(3)
C(25)-C(24)-C(23)-C(28)	-0.4(4)
C(25)-C(24)-C(23)-C(19)	-177.4(3)
C(20)-C(19)-C(23)-C(28)	14.6(4)
P(2)-C(19)-C(23)-C(28)	-107.5(3)
C(20)-C(19)-C(23)-C(24)	-168.5(3)
P(2)-C(19)-C(23)-C(24)	69.4(3)
C(68)-C(67)-C(66)-C(65)	0.9(6)
C(64)-C(65)-C(66)-C(67)	0.3(6)

C(45)-C(38)-C(37)-C(36)	-157.6(3)
P(3)-C(38)-C(37)-C(36)	-27.6(3)
C(35)-C(36)-C(37)-C(38)	-4.5(4)
C(9)-C(10)-C(5)-C(6)	0.0(4)
C(9)-C(10)-C(5)-C(1)	176.5(3)
C(7)-C(6)-C(5)-C(10)	0.0(4)
C(7)-C(6)-C(5)-C(1)	-176.7(3)
C(2)-C(1)-C(5)-C(10)	24.2(4)
P(1)-C(1)-C(5)-C(10)	-93.9(3)
C(2)-C(1)-C(5)-C(6)	-159.2(3)
P(1)-C(1)-C(5)-C(6)	82.6(3)
C(23)-C(24)-C(25)-C(26)	0.0(5)
C(27)-C(26)-C(25)-C(24)	-0.2(5)
C(13)-C(14)-C(15)-C(16)	0.0(5)
C(11)-C(16)-C(15)-C(14)	1.3(5)
C(44)-C(39)-C(40)-C(41)	1.4(4)
C(35)-C(39)-C(40)-C(41)	-176.7(3)
C(42)-C(41)-C(40)-C(39)	-1.7(5)
C(41)-C(42)-C(43)-C(44)	1.6(5)
C(39)-C(44)-C(43)-C(42)	-1.8(5)
C(67)-C(68)-C(63)-C(64)	0.9(4)
C(67)-C(68)-C(63)-C(56)	-177.7(3)
C(65)-C(64)-C(63)-C(68)	0.3(4)
C(65)-C(64)-C(63)-C(56)	179.0(3)
C(68)-C(63)-C(56)-C(55)	3.9(4)
C(64)-C(63)-C(56)-C(55)	-174.7(3)
C(68)-C(63)-C(56)-P(4)	-120.9(3)
C(64)-C(63)-C(56)-P(4)	60.5(3)
C(54)-C(55)-C(56)-C(63)	-163.1(2)
C(54)-C(55)-C(56)-P(4)	-34.4(3)
C(52)-P(4)-C(56)-C(63)	34.4(2)
C(53)-P(4)-C(56)-C(63)	137.5(2)
Rh(01)-P(4)-C(56)-C(63)	-94.52(19)
C(52)-P(4)-C(56)-C(55)	-96.7(2)
C(53)-P(4)-C(56)-C(55)	6.4(2)

Rh(01)-P(4)-C(56)-C(55)	134.38(17)
C(56)-C(55)-C(54)-C(53)	53.0(3)
C(57)-C(53)-C(54)-C(55)	-174.8(2)
P(4)-C(53)-C(54)-C(55)	-45.6(2)

Symmetry transformations used to generate equivalent atoms:

6.2 [Rh_2(Ph-BPE)$_2$(μ-Cl)(μ-H)] (5)

Table S14. Crystal data and structure refinement for **5**.

Identification code	**5**	
Empirical formula	C34 H36.50 Cl0.50 P2 Rh	
Formula weight	627.70	
Temperature	100(2) K	
Wavelength	0.71073 Å	
Crystal system	Orthorhombic	
Space group	$P2_12_12_1$	
Unit cell dimensions	a = 10.8094(3) Å	α= 90°.
	b = 22.1084(5) Å	β= 90°.
	c = 24.4640(5) Å	γ = 90°.
Volume	5846.4(2) Å^3	
Z	8	
Density (calculated)	1.426 Mg/m^3	
Absorption coefficient	0.761 mm^{-1}	
F(000)	2592	
Crystal size	0.303 x 0.201 x 0.199 mm^3	
Theta range for data collection	1.241 to 27.173°.	
Index ranges	-13<=h<=8, -28<=k<=15, -31<=l<=30	
Reflections collected	28950	
Independent reflections	12742 [R(int) = 0.0219]	
Completeness to theta = 25.242°	100.0 %	
Absorption correction	Semi-empirical from equivalents	
Max. and min. transmission	1.0 and 0.89	
Refinement method	Full-matrix least-squares on F^2	
Data / restraints / parameters	12742 / 48 / 669	
Goodness-of-fit on F^2	1.071	

Final R indices [I>2sigma(I)]	R1 = 0.0248, wR2 = 0.0566
R indices (all data)	R1 = 0.0288, wR2 = 0.0627
Absolute structure parameter	-0.021(8)
Extinction coefficient	n/a
Largest diff. peak and hole	0.605 and -0.355 e.Å^{-3}

Table S15. Atomic coordinates (x 10^4) and equivalent isotropic displacement parameters (Å^2 x 10^3) for **5**. U(eq) is defined as one third of the trace of the orthogonalized U^{ij} tensor.

	x	y	z	U(eq)
Rh(01)	8080(1)	5872(1)	7107(1)	14(1)
Rh(02)	7493(1)	5556(1)	6023(1)	14(1)
Cl(03)	9285(1)	6104(1)	6309(1)	18(1)
P(3)	5759(1)	5136(1)	5809(1)	15(1)
P(2)	7080(1)	5546(1)	7815(1)	16(1)
P(1)	9282(1)	6360(1)	7686(1)	17(1)
P(4)	7876(1)	5552(1)	5145(1)	17(1)
C(22)	5457(3)	5789(2)	7958(1)	19(1)
C(29)	5401(4)	6427(2)	8186(2)	23(1)
C(47)	5690(4)	3873(2)	5584(2)	24(1)
C(40)	5601(4)	4327(1)	6043(1)	18(1)
C(38)	4170(4)	4954(2)	6611(1)	19(1)
C(43)	4409(4)	6992(2)	6573(2)	23(1)
C(56)	8112(4)	6314(2)	4820(1)	21(1)
C(63)	6913(4)	6600(2)	4657(1)	21(1)
C(23)	7185(3)	4250(2)	7553(2)	22(1)
C(6)	10941(4)	6096(2)	7682(2)	24(1)
C(37)	4237(3)	5352(2)	6100(1)	18(1)
C(44)	3367(4)	7234(2)	6327(2)	24(1)
C(34)	5881(4)	6902(2)	7884(2)	23(1)
C(41)	3976(3)	6020(2)	6176(1)	17(1)
C(39)	4442(4)	4309(2)	6411(1)	21(1)
C(60)	11127(4)	3839(2)	5749(2)	36(1)
C(57)	9954(4)	4749(2)	5152(2)	24(1)
C(19)	6726(3)	4722(2)	7952(1)	21(1)
C(35)	5443(4)	5186(2)	5070(1)	18(1)
C(36)	6659(4)	5159(2)	4756(1)	20(1)
C(21)	4902(4)	5288(2)	8304(1)	24(1)
C(53)	9300(4)	5225(2)	4817(2)	23(1)
C(24)	6603(4)	3689(2)	7522(2)	31(1)
C(30)	4875(4)	6551(2)	8694(2)	31(1)

C(3)	9742(4)	7165(2)	7604(2)	25(1)
C(27)	8581(4)	3907(2)	6837(2)	34(1)
C(64)	6430(4)	6565(2)	4129(1)	23(1)
C(58)	10635(4)	4888(2)	5613(2)	29(1)
C(28)	8197(4)	4347(2)	7210(2)	25(1)
C(20)	5321(4)	4691(2)	8056(2)	22(1)
C(52)	4666(5)	3683(2)	5284(2)	34(1)
C(42)	4718(4)	6387(2)	6498(1)	20(1)
C(33)	5897(4)	7486(2)	8091(2)	32(1)
C(4)	10935(4)	7116(2)	7272(2)	27(1)
C(45)	2630(4)	6876(2)	6004(2)	27(1)
C(46)	2931(4)	6266(2)	5930(2)	24(1)
C(1)	8666(3)	6325(2)	8383(1)	20(1)
C(5)	11738(4)	6660(2)	7568(2)	34(1)
C(48)	6844(5)	3643(2)	5443(2)	35(1)
C(26)	7974(5)	3352(2)	6816(2)	37(1)
C(7)	8815(4)	7613(2)	7386(2)	30(1)
C(54)	10057(4)	5782(2)	4640(2)	28(1)
C(25)	7004(4)	3244(2)	7159(2)	38(1)
C(61)	10440(5)	3698(2)	5298(2)	38(1)
C(59)	11215(4)	4436(2)	5911(2)	34(1)
C(55)	9130(4)	6224(2)	4394(2)	27(1)
C(8)	8107(4)	7499(2)	6924(2)	30(1)
C(68)	6197(4)	6886(2)	5060(2)	26(1)
C(32)	5411(5)	7600(2)	8602(2)	43(1)
C(65)	5289(4)	6819(2)	4003(2)	29(1)
C(2)	7929(4)	5744(2)	8455(1)	21(1)
C(66)	4605(4)	7103(2)	4402(2)	36(1)
C(62)	9846(4)	4143(2)	5002(2)	32(1)
C(12)	8701(5)	8176(2)	7635(2)	51(1)
C(31)	4891(5)	7137(2)	8896(2)	44(1)
C(50)	5957(7)	3045(2)	4728(2)	57(2)
C(67)	5056(5)	7133(2)	4934(2)	34(1)
C(10)	7222(5)	8495(2)	6972(3)	66(2)
C(51)	4808(6)	3263(2)	4860(2)	48(2)

C(9)	7304(4)	7936(2)	6718(2)	45(1)
C(11)	7923(6)	8610(2)	7427(3)	71(2)
C(49)	6972(7)	3230(2)	5012(2)	52(2)
C(14)	11047(3)	5096(1)	8157(1)	52(1)
C(13)	11273(3)	5714(1)	8181(1)	27(1)
C(18)	11761(3)	5969(1)	8654(1)	34(1)
C(17)	12023(3)	5605(2)	9102(1)	61(2)
C(16)	11797(3)	4987(2)	9078(1)	85(2)
C(15)	11309(3)	4732(1)	8606(2)	80(2)

Table S16. Bond lengths [Å] and angles [°] for **5**.

Rh(01)-P(2)	2.1656(9)
Rh(01)-P(1)	2.2055(10)
Rh(01)-Cl(03)	2.4018(8)
Rh(01)-Rh(02)	2.8136(3)
Rh(01)-H(1)	1.68(5)
Rh(02)-P(3)	2.1559(10)
Rh(02)-P(4)	2.1886(8)
Rh(02)-Cl(03)	2.3895(9)
Rh(02)-H(1)	1.77(5)
P(3)-C(35)	1.842(3)
P(3)-C(37)	1.854(4)
P(3)-C(40)	1.888(3)
P(2)-C(2)	1.867(4)
P(2)-C(22)	1.869(4)
P(2)-C(19)	1.893(3)
P(1)-C(1)	1.832(4)
P(1)-C(3)	1.858(4)
P(1)-C(6)	1.886(4)
P(4)-C(36)	1.841(4)
P(4)-C(53)	1.879(4)
P(4)-C(56)	1.881(3)
C(22)-C(29)	1.517(5)
C(22)-C(21)	1.519(5)
C(22)-H(22)	1.0000
C(29)-C(34)	1.383(5)
C(29)-C(30)	1.396(5)
C(47)-C(48)	1.389(7)
C(47)-C(52)	1.392(6)
C(47)-C(40)	1.509(5)
C(40)-C(39)	1.544(5)
C(40)-H(40)	1.0000
C(38)-C(37)	1.531(5)
C(38)-C(39)	1.535(5)

C(38)-H(38A)	0.9900
C(38)-H(38B)	0.9900
C(43)-C(44)	1.384(6)
C(43)-C(42)	1.392(5)
C(43)-H(43)	0.9500
C(56)-C(63)	1.496(6)
C(56)-C(55)	1.529(5)
C(56)-H(56)	1.0000
C(63)-C(64)	1.395(5)
C(63)-C(68)	1.403(5)
C(23)-C(24)	1.394(5)
C(23)-C(28)	1.395(5)
C(23)-C(19)	1.512(5)
C(6)-C(13)	1.526(4)
C(6)-C(5)	1.542(5)
C(6)-H(6)	1.0000
C(37)-C(41)	1.516(5)
C(37)-H(37)	1.0000
C(44)-C(45)	1.372(5)
C(44)-H(44)	0.9500
C(34)-C(33)	1.389(5)
C(34)-H(34)	0.9500
C(41)-C(42)	1.387(5)
C(41)-C(46)	1.392(5)
C(39)-H(39A)	0.9900
C(39)-H(39B)	0.9900
C(60)-C(61)	1.367(7)
C(60)-C(59)	1.381(6)
C(60)-H(60)	0.9500
C(57)-C(58)	1.383(6)
C(57)-C(62)	1.393(5)
C(57)-C(53)	1.508(5)
C(19)-C(20)	1.542(5)
C(19)-H(19)	1.0000
C(35)-C(36)	1.524(5)

C(35)-H(35A)	0.9900
C(35)-H(35B)	0.9900
C(36)-H(36A)	0.9900
C(36)-H(36B)	0.9900
C(21)-C(20)	1.521(5)
C(21)-H(21A)	0.9900
C(21)-H(21B)	0.9900
C(53)-C(54)	1.542(5)
C(53)-H(53)	1.0000
C(24)-C(25)	1.395(6)
C(24)-H(24)	0.9500
C(30)-C(31)	1.388(6)
C(30)-H(30)	0.9500
C(3)-C(7)	1.505(6)
C(3)-C(4)	1.527(5)
C(3)-H(3)	1.0000
C(27)-C(26)	1.392(6)
C(27)-C(28)	1.398(5)
C(27)-H(27)	0.9500
C(64)-C(65)	1.391(6)
C(64)-H(64)	0.9500
C(58)-C(59)	1.388(6)
C(58)-H(58)	0.9500
C(28)-H(28)	0.9500
C(20)-H(20A)	0.9900
C(20)-H(20B)	0.9900
C(52)-C(51)	1.401(6)
C(52)-H(52)	0.9500
C(42)-H(42)	0.9500
C(33)-C(32)	1.378(6)
C(33)-H(33)	0.9500
C(4)-C(5)	1.515(6)
C(4)-H(4A)	0.9900
C(4)-H(4B)	0.9900
C(45)-C(46)	1.398(5)

C(45)-H(45)	0.9500
C(46)-H(46)	0.9500
C(1)-C(2)	1.521(5)
C(1)-H(1A)	0.9900
C(1)-H(1B)	0.9900
C(5)-H(5A)	0.9900
C(5)-H(5B)	0.9900
C(48)-C(49)	1.400(6)
C(48)-H(48)	0.9500
C(26)-C(25)	1.363(7)
C(26)-H(26)	0.9500
C(7)-C(8)	1.389(6)
C(7)-C(12)	1.391(6)
C(54)-C(55)	1.524(6)
C(54)-H(54A)	0.9900
C(54)-H(54B)	0.9900
C(25)-H(25)	0.9500
C(61)-C(62)	1.380(6)
C(61)-H(61)	0.9500
C(59)-H(59)	0.9500
C(55)-H(55A)	0.9900
C(55)-H(55B)	0.9900
C(8)-C(9)	1.394(6)
C(8)-H(8)	0.9500
C(68)-C(67)	1.384(6)
C(68)-H(68)	0.9500
C(32)-C(31)	1.372(7)
C(32)-H(32)	0.9500
C(65)-C(66)	1.377(6)
C(65)-H(65)	0.9500
C(2)-H(2A)	0.9900
C(2)-H(2B)	0.9900
C(66)-C(67)	1.391(6)
C(66)-H(66)	0.9500
C(62)-H(62)	0.9500

C(12)-C(11)	1.373(8)
C(12)-H(12)	0.9500
C(31)-H(31)	0.9500
C(50)-C(49)	1.363(9)
C(50)-C(51)	1.371(9)
C(50)-H(50)	0.9500
C(67)-H(67)	0.9500
C(10)-C(11)	1.369(9)
C(10)-C(9)	1.388(7)
C(10)-H(10)	0.9500
C(51)-H(51)	0.9500
C(9)-H(9)	0.9500
C(11)-H(11)	0.9500
C(49)-H(49)	0.9500
C(14)-C(13)	1.3900
C(14)-C(15)	1.3900
C(14)-H(14)	0.9500
C(13)-C(18)	1.3900
C(18)-C(17)	1.3900
C(18)-H(18)	0.9500
C(17)-C(16)	1.3900
C(17)-H(17)	0.9500
C(16)-C(15)	1.3900
C(16)-H(16)	0.9500
C(15)-H(15)	0.9500

P(2)-Rh(01)-P(1)	86.69(3)
P(2)-Rh(01)-Cl(03)	172.77(3)
P(1)-Rh(01)-Cl(03)	95.62(3)
P(2)-Rh(01)-Rh(02)	123.99(3)
P(1)-Rh(01)-Rh(02)	149.30(3)
Cl(03)-Rh(01)-Rh(02)	53.84(2)
P(2)-Rh(01)-H(1)	87.5(17)
P(1)-Rh(01)-H(1)	174.1(16)
Cl(03)-Rh(01)-H(1)	90.0(17)

Rh(02)-Rh(01)-H(1)	36.5(16)
P(3)-Rh(02)-P(4)	85.64(3)
P(3)-Rh(02)-Cl(03)	173.77(3)
P(4)-Rh(02)-Cl(03)	97.82(3)
P(3)-Rh(02)-Rh(01)	122.14(3)
P(4)-Rh(02)-Rh(01)	152.06(3)
Cl(03)-Rh(02)-Rh(01)	54.24(2)
P(3)-Rh(02)-H(1)	88.8(17)
P(4)-Rh(02)-H(1)	172.5(15)
Cl(03)-Rh(02)-H(1)	88.2(17)
Rh(01)-Rh(02)-H(1)	34.3(17)
Rh(02)-Cl(03)-Rh(01)	71.92(3)
C(35)-P(3)-C(37)	101.35(17)
C(35)-P(3)-C(40)	109.70(15)
C(37)-P(3)-C(40)	92.65(16)
C(35)-P(3)-Rh(02)	111.97(13)
C(37)-P(3)-Rh(02)	124.59(12)
C(40)-P(3)-Rh(02)	114.40(12)
C(2)-P(2)-C(22)	103.72(16)
C(2)-P(2)-C(19)	100.14(16)
C(22)-P(2)-C(19)	93.09(16)
C(2)-P(2)-Rh(01)	110.40(12)
C(22)-P(2)-Rh(01)	121.55(11)
C(19)-P(2)-Rh(01)	124.23(12)
C(1)-P(1)-C(3)	103.84(17)
C(1)-P(1)-C(6)	109.67(17)
C(3)-P(1)-C(6)	92.41(19)
C(1)-P(1)-Rh(01)	111.27(12)
C(3)-P(1)-Rh(01)	123.88(13)
C(6)-P(1)-Rh(01)	113.87(12)
C(36)-P(4)-C(53)	100.56(17)
C(36)-P(4)-C(56)	107.60(17)
C(53)-P(4)-C(56)	93.12(17)
C(36)-P(4)-Rh(02)	111.93(12)
C(53)-P(4)-Rh(02)	125.07(12)

C(56)-P(4)-Rh(02)	115.89(11)
C(29)-C(22)-C(21)	117.3(3)
C(29)-C(22)-P(2)	111.9(2)
C(21)-C(22)-P(2)	105.4(2)
C(29)-C(22)-H(22)	107.3
C(21)-C(22)-H(22)	107.3
P(2)-C(22)-H(22)	107.3
C(34)-C(29)-C(30)	118.6(4)
C(34)-C(29)-C(22)	119.7(3)
C(30)-C(29)-C(22)	121.7(4)
C(48)-C(47)-C(52)	118.2(4)
C(48)-C(47)-C(40)	119.0(4)
C(52)-C(47)-C(40)	122.7(4)
C(47)-C(40)-C(39)	118.0(3)
C(47)-C(40)-P(3)	113.5(2)
C(39)-C(40)-P(3)	106.0(2)
C(47)-C(40)-H(40)	106.2
C(39)-C(40)-H(40)	106.2
P(3)-C(40)-H(40)	106.2
C(37)-C(38)-C(39)	105.4(3)
C(37)-C(38)-H(38A)	110.7
C(39)-C(38)-H(38A)	110.7
C(37)-C(38)-H(38B)	110.7
C(39)-C(38)-H(38B)	110.7
H(38A)-C(38)-H(38B)	108.8
C(44)-C(43)-C(42)	120.5(4)
C(44)-C(43)-H(43)	119.7
C(42)-C(43)-H(43)	119.7
C(63)-C(56)-C(55)	119.8(3)
C(63)-C(56)-P(4)	111.9(3)
C(55)-C(56)-P(4)	105.6(2)
C(63)-C(56)-H(56)	106.2
C(55)-C(56)-H(56)	106.2
P(4)-C(56)-H(56)	106.2
C(64)-C(63)-C(68)	118.0(4)

C(64)-C(63)-C(56)	123.1(3)
C(68)-C(63)-C(56)	118.8(3)
C(24)-C(23)-C(28)	117.3(4)
C(24)-C(23)-C(19)	120.0(3)
C(28)-C(23)-C(19)	122.7(3)
C(13)-C(6)-C(5)	117.5(3)
C(13)-C(6)-P(1)	113.1(2)
C(5)-C(6)-P(1)	106.3(3)
C(13)-C(6)-H(6)	106.4
C(5)-C(6)-H(6)	106.4
P(1)-C(6)-H(6)	106.4
C(41)-C(37)-C(38)	116.8(3)
C(41)-C(37)-P(3)	117.5(3)
C(38)-C(37)-P(3)	102.0(2)
C(41)-C(37)-H(37)	106.5
C(38)-C(37)-H(37)	106.5
P(3)-C(37)-H(37)	106.5
C(45)-C(44)-C(43)	120.1(3)
C(45)-C(44)-H(44)	120.0
C(43)-C(44)-H(44)	120.0
C(29)-C(34)-C(33)	121.1(4)
C(29)-C(34)-H(34)	119.5
C(33)-C(34)-H(34)	119.5
C(42)-C(41)-C(46)	119.1(3)
C(42)-C(41)-C(37)	122.2(3)
C(46)-C(41)-C(37)	118.7(3)
C(38)-C(39)-C(40)	108.5(3)
C(38)-C(39)-H(39A)	110.0
C(40)-C(39)-H(39A)	110.0
C(38)-C(39)-H(39B)	110.0
C(40)-C(39)-H(39B)	110.0
H(39A)-C(39)-H(39B)	108.4
C(61)-C(60)-C(59)	119.1(4)
C(61)-C(60)-H(60)	120.5
C(59)-C(60)-H(60)	120.5

C(58)-C(57)-C(62)	118.2(4)
C(58)-C(57)-C(53)	122.4(3)
C(62)-C(57)-C(53)	119.3(4)
C(23)-C(19)-C(20)	113.6(3)
C(23)-C(19)-P(2)	118.9(2)
C(20)-C(19)-P(2)	105.7(2)
C(23)-C(19)-H(19)	105.9
C(20)-C(19)-H(19)	105.9
P(2)-C(19)-H(19)	105.9
C(36)-C(35)-P(3)	109.4(3)
C(36)-C(35)-H(35A)	109.8
P(3)-C(35)-H(35A)	109.8
C(36)-C(35)-H(35B)	109.8
P(3)-C(35)-H(35B)	109.8
H(35A)-C(35)-H(35B)	108.2
C(35)-C(36)-P(4)	109.7(2)
C(35)-C(36)-H(36A)	109.7
P(4)-C(36)-H(36A)	109.7
C(35)-C(36)-H(36B)	109.7
P(4)-C(36)-H(36B)	109.7
H(36A)-C(36)-H(36B)	108.2
C(22)-C(21)-C(20)	107.0(3)
C(22)-C(21)-H(21A)	110.3
C(20)-C(21)-H(21A)	110.3
C(22)-C(21)-H(21B)	110.3
C(20)-C(21)-H(21B)	110.3
H(21A)-C(21)-H(21B)	108.6
C(57)-C(53)-C(54)	117.5(3)
C(57)-C(53)-P(4)	114.9(3)
C(54)-C(53)-P(4)	104.3(3)
C(57)-C(53)-H(53)	106.5
C(54)-C(53)-H(53)	106.5
P(4)-C(53)-H(53)	106.5
C(23)-C(24)-C(25)	121.4(4)
C(23)-C(24)-H(24)	119.3

C(25)-C(24)-H(24)	119.3
C(31)-C(30)-C(29)	119.7(4)
C(31)-C(30)-H(30)	120.1
C(29)-C(30)-H(30)	120.1
C(7)-C(3)-C(4)	114.9(3)
C(7)-C(3)-P(1)	119.3(3)
C(4)-C(3)-P(1)	102.5(3)
C(7)-C(3)-H(3)	106.4
C(4)-C(3)-H(3)	106.4
P(1)-C(3)-H(3)	106.4
C(26)-C(27)-C(28)	119.7(4)
C(26)-C(27)-H(27)	120.1
C(28)-C(27)-H(27)	120.1
C(65)-C(64)-C(63)	121.0(4)
C(65)-C(64)-H(64)	119.5
C(63)-C(64)-H(64)	119.5
C(57)-C(58)-C(59)	120.6(4)
C(57)-C(58)-H(58)	119.7
C(59)-C(58)-H(58)	119.7
C(23)-C(28)-C(27)	121.3(4)
C(23)-C(28)-H(28)	119.4
C(27)-C(28)-H(28)	119.4
C(21)-C(20)-C(19)	108.8(3)
C(21)-C(20)-H(20A)	109.9
C(19)-C(20)-H(20A)	109.9
C(21)-C(20)-H(20B)	109.9
C(19)-C(20)-H(20B)	109.9
H(20A)-C(20)-H(20B)	108.3
C(47)-C(52)-C(51)	120.2(5)
C(47)-C(52)-H(52)	119.9
C(51)-C(52)-H(52)	119.9
C(41)-C(42)-C(43)	120.0(4)
C(41)-C(42)-H(42)	120.0
C(43)-C(42)-H(42)	120.0
C(32)-C(33)-C(34)	119.8(4)

C(32)-C(33)-H(33)	120.1
C(34)-C(33)-H(33)	120.1
C(5)-C(4)-C(3)	106.1(3)
C(5)-C(4)-H(4A)	110.5
C(3)-C(4)-H(4A)	110.5
C(5)-C(4)-H(4B)	110.5
C(3)-C(4)-H(4B)	110.5
H(4A)-C(4)-H(4B)	108.7
C(44)-C(45)-C(46)	119.7(4)
C(44)-C(45)-H(45)	120.1
C(46)-C(45)-H(45)	120.1
C(41)-C(46)-C(45)	120.7(4)
C(41)-C(46)-H(46)	119.7
C(45)-C(46)-H(46)	119.7
C(2)-C(1)-P(1)	109.6(2)
C(2)-C(1)-H(1A)	109.8
P(1)-C(1)-H(1A)	109.8
C(2)-C(1)-H(1B)	109.8
P(1)-C(1)-H(1B)	109.8
H(1A)-C(1)-H(1B)	108.2
C(4)-C(5)-C(6)	107.8(3)
C(4)-C(5)-H(5A)	110.1
C(6)-C(5)-H(5A)	110.1
C(4)-C(5)-H(5B)	110.1
C(6)-C(5)-H(5B)	110.1
H(5A)-C(5)-H(5B)	108.5
C(47)-C(48)-C(49)	120.9(5)
C(47)-C(48)-H(48)	119.5
C(49)-C(48)-H(48)	119.5
C(25)-C(26)-C(27)	119.7(4)
C(25)-C(26)-H(26)	120.1
C(27)-C(26)-H(26)	120.1
C(8)-C(7)-C(12)	118.0(4)
C(8)-C(7)-C(3)	122.4(3)
C(12)-C(7)-C(3)	119.6(4)

C(55)-C(54)-C(53)	105.9(3)
C(55)-C(54)-H(54A)	110.5
C(53)-C(54)-H(54A)	110.5
C(55)-C(54)-H(54B)	110.5
C(53)-C(54)-H(54B)	110.5
H(54A)-C(54)-H(54B)	108.7
C(26)-C(25)-C(24)	120.5(4)
C(26)-C(25)-H(25)	119.8
C(24)-C(25)-H(25)	119.8
C(60)-C(61)-C(62)	120.9(4)
C(60)-C(61)-H(61)	119.5
C(62)-C(61)-H(61)	119.5
C(60)-C(59)-C(58)	120.5(4)
C(60)-C(59)-H(59)	119.7
C(58)-C(59)-H(59)	119.7
C(54)-C(55)-C(56)	106.8(3)
C(54)-C(55)-H(55A)	110.4
C(56)-C(55)-H(55A)	110.4
C(54)-C(55)-H(55B)	110.4
C(56)-C(55)-H(55B)	110.4
H(55A)-C(55)-H(55B)	108.6
C(7)-C(8)-C(9)	120.8(4)
C(7)-C(8)-H(8)	119.6
C(9)-C(8)-H(8)	119.6
C(67)-C(68)-C(63)	120.9(4)
C(67)-C(68)-H(68)	119.6
C(63)-C(68)-H(68)	119.6
C(31)-C(32)-C(33)	119.7(4)
C(31)-C(32)-H(32)	120.1
C(33)-C(32)-H(32)	120.1
C(66)-C(65)-C(64)	120.2(4)
C(66)-C(65)-H(65)	119.9
C(64)-C(65)-H(65)	119.9
C(1)-C(2)-P(2)	110.9(2)
C(1)-C(2)-H(2A)	109.5

P(2)-C(2)-H(2A)	109.5
C(1)-C(2)-H(2B)	109.5
P(2)-C(2)-H(2B)	109.5
H(2A)-C(2)-H(2B)	108.0
C(65)-C(66)-C(67)	119.9(4)
C(65)-C(66)-H(66)	120.1
C(67)-C(66)-H(66)	120.1
C(61)-C(62)-C(57)	120.6(4)
C(61)-C(62)-H(62)	119.7
C(57)-C(62)-H(62)	119.7
C(11)-C(12)-C(7)	121.2(6)
C(11)-C(12)-H(12)	119.4
C(7)-C(12)-H(12)	119.4
C(32)-C(31)-C(30)	121.0(4)
C(32)-C(31)-H(31)	119.5
C(30)-C(31)-H(31)	119.5
C(49)-C(50)-C(51)	120.2(4)
C(49)-C(50)-H(50)	119.9
C(51)-C(50)-H(50)	119.9
C(68)-C(67)-C(66)	120.1(4)
C(68)-C(67)-H(67)	119.9
C(66)-C(67)-H(67)	119.9
C(11)-C(10)-C(9)	119.7(5)
C(11)-C(10)-H(10)	120.2
C(9)-C(10)-H(10)	120.2
C(50)-C(51)-C(52)	120.5(5)
C(50)-C(51)-H(51)	119.8
C(52)-C(51)-H(51)	119.8
C(10)-C(9)-C(8)	119.6(5)
C(10)-C(9)-H(9)	120.2
C(8)-C(9)-H(9)	120.2
C(10)-C(11)-C(12)	120.7(5)
C(10)-C(11)-H(11)	119.7
C(12)-C(11)-H(11)	119.7
C(50)-C(49)-C(48)	120.0(6)

C(50)-C(49)-H(49)	120.0
C(48)-C(49)-H(49)	120.0
C(13)-C(14)-C(15)	120.0
C(13)-C(14)-H(14)	120.0
C(15)-C(14)-H(14)	120.0
C(14)-C(13)-C(18)	120.0
C(14)-C(13)-C(6)	117.9(2)
C(18)-C(13)-C(6)	122.0(2)
C(17)-C(18)-C(13)	120.0
C(17)-C(18)-H(18)	120.0
C(13)-C(18)-H(18)	120.0
C(18)-C(17)-C(16)	120.0
C(18)-C(17)-H(17)	120.0
C(16)-C(17)-H(17)	120.0
C(15)-C(16)-C(17)	120.0
C(15)-C(16)-H(16)	120.0
C(17)-C(16)-H(16)	120.0
C(16)-C(15)-C(14)	120.0
C(16)-C(15)-H(15)	120.0
C(14)-C(15)-H(15)	120.0

Symmetry transformations used to generate equivalent atoms:

Table S17. Anisotropic displacement parameters ($Å^2 x 10^3$) for **5**. The anisotropic displacement factor exponent takes the form: $-2\pi^2[h^2 a^{*2}U^{11} + ... + 2 h k a^* b^* U^{12}]$.

	U^{11}	U^{22}	U^{33}	U^{23}	U^{13}	U^{12}
Rh(01)	11(1)	16(1)	13(1)	0(1)	0(1)	-1(1)
Rh(02)	13(1)	15(1)	13(1)	0(1)	0(1)	0(1)
Cl(03)	15(1)	24(1)	15(1)	-2(1)	2(1)	-7(1)
P(3)	16(1)	14(1)	16(1)	1(1)	-1(1)	1(1)
P(2)	12(1)	21(1)	15(1)	2(1)	-1(1)	-1(1)
P(1)	12(1)	23(1)	17(1)	-4(1)	1(1)	-2(1)
P(4)	19(1)	17(1)	15(1)	-1(1)	2(1)	0(1)
C(22)	12(2)	31(2)	14(2)	1(1)	0(1)	-1(2)
C(29)	13(2)	34(2)	22(2)	-2(2)	-2(2)	5(2)
C(47)	38(3)	12(2)	21(2)	4(1)	3(2)	-3(2)
C(40)	20(2)	16(2)	17(2)	2(1)	0(2)	1(2)
C(38)	15(2)	18(2)	24(2)	2(1)	2(2)	-1(2)
C(43)	23(2)	22(2)	24(2)	-2(2)	2(2)	-1(2)
C(56)	25(2)	19(2)	19(2)	1(1)	4(2)	-3(2)
C(63)	24(2)	14(2)	24(2)	3(1)	4(2)	-6(2)
C(23)	18(2)	18(2)	29(2)	9(1)	-7(2)	3(2)
C(6)	10(2)	39(2)	23(2)	-6(2)	1(2)	0(2)
C(37)	15(2)	17(2)	22(2)	1(1)	-4(2)	-1(2)
C(44)	23(2)	19(2)	31(2)	4(2)	5(2)	7(2)
C(34)	18(2)	29(2)	22(2)	-2(2)	3(2)	7(2)
C(41)	14(2)	18(2)	19(2)	2(1)	3(1)	1(2)
C(39)	22(2)	18(2)	21(2)	2(1)	-1(2)	0(2)
C(60)	25(2)	32(2)	51(3)	10(2)	8(2)	11(2)
C(57)	21(2)	28(2)	24(2)	-1(2)	8(2)	5(2)
C(19)	16(2)	24(2)	22(2)	8(1)	-1(2)	-3(2)
C(35)	21(2)	12(2)	20(2)	0(1)	-5(2)	-2(2)
C(36)	31(2)	12(2)	17(2)	-1(1)	1(2)	-1(2)
C(21)	13(2)	39(2)	20(2)	5(2)	2(2)	-4(2)
C(53)	24(2)	25(2)	21(2)	-2(2)	4(2)	1(2)
C(24)	19(2)	24(2)	51(3)	10(2)	-5(2)	1(2)
C(30)	30(2)	43(2)	20(2)	0(2)	4(2)	7(2)

C(3)	23(2)	29(2)	24(2)	-8(2)	4(2)	-9(2)
C(27)	36(3)	27(2)	37(2)	5(2)	1(2)	10(2)
C(64)	30(2)	16(2)	23(2)	4(1)	3(2)	-4(2)
C(58)	17(2)	28(2)	40(2)	-6(2)	1(2)	1(2)
C(28)	24(2)	17(2)	34(2)	4(1)	1(2)	2(2)
C(20)	15(2)	29(2)	23(2)	10(2)	0(2)	-4(2)
C(52)	55(3)	23(2)	25(2)	5(2)	-5(2)	-11(2)
C(42)	16(2)	23(2)	22(2)	2(1)	-1(2)	4(2)
C(33)	30(3)	30(2)	36(2)	-4(2)	-2(2)	8(2)
C(4)	16(2)	36(2)	30(2)	1(2)	-1(2)	-8(2)
C(45)	21(2)	25(2)	34(2)	9(2)	2(2)	7(2)
C(46)	19(2)	22(2)	30(2)	2(1)	-5(2)	-1(2)
C(1)	11(2)	30(2)	18(2)	-4(1)	1(1)	1(2)
C(5)	15(2)	53(3)	33(2)	16(2)	-2(2)	-8(2)
C(48)	52(3)	21(2)	31(2)	4(2)	11(2)	4(2)
C(26)	40(3)	26(2)	44(2)	-2(2)	-12(2)	13(2)
C(7)	21(2)	22(2)	47(2)	-4(2)	13(2)	-4(2)
C(54)	25(2)	34(2)	25(2)	5(2)	8(2)	2(2)
C(25)	27(2)	21(2)	64(3)	4(2)	-15(3)	4(2)
C(61)	43(3)	25(2)	46(3)	-3(2)	11(2)	5(2)
C(59)	19(2)	39(2)	43(2)	3(2)	-2(2)	7(2)
C(55)	26(2)	33(2)	23(2)	7(2)	7(2)	0(2)
C(8)	24(2)	23(2)	43(2)	8(2)	9(2)	-1(2)
C(68)	34(3)	21(2)	25(2)	0(2)	6(2)	4(2)
C(32)	57(3)	38(3)	36(2)	-11(2)	-6(2)	20(3)
C(65)	29(2)	26(2)	33(2)	6(2)	-6(2)	-6(2)
C(2)	13(2)	35(2)	16(2)	3(1)	1(1)	3(2)
C(66)	26(3)	28(2)	54(3)	9(2)	-2(2)	2(2)
C(62)	37(3)	28(2)	31(2)	-5(2)	5(2)	3(2)
C(12)	36(3)	33(3)	85(4)	-20(2)	19(3)	-5(2)
C(31)	55(3)	54(3)	22(2)	-11(2)	1(2)	22(3)
C(50)	130(6)	19(2)	21(2)	1(2)	22(3)	-2(3)
C(67)	35(3)	26(2)	41(2)	1(2)	15(2)	3(2)
C(10)	29(3)	28(3)	141(6)	21(3)	23(3)	9(2)
C(51)	95(5)	22(2)	27(2)	5(2)	-7(3)	-22(3)

C(9)	24(3)	38(2)	72(3)	21(2)	10(2)	4(2)
C(11)	43(4)	24(2)	147(6)	-16(3)	34(4)	6(3)
C(49)	99(5)	20(2)	38(2)	2(2)	30(3)	13(3)
C(14)	34(3)	35(2)	88(4)	8(3)	24(3)	8(2)
C(13)	10(2)	29(2)	41(2)	5(2)	10(2)	5(2)
C(18)	19(2)	58(3)	27(2)	7(2)	5(2)	10(2)
C(17)	31(3)	118(4)	36(2)	28(3)	17(2)	36(3)
C(16)	51(4)	105(5)	98(4)	73(4)	55(4)	59(4)
C(15)	55(4)	44(3)	141(6)	46(3)	57(4)	22(3)

Table S18. Hydrogen coordinates (x 10^4) and isotropic displacement parameters (Å^{2}x 10^3) for **5**.

	x	y	z	U(eq)
H(22)	5005	5791	7601	23
H(40)	6323	4247	6288	21
H(38A)	3337	4976	6778	23
H(38B)	4791	5083	6884	23
H(43)	4918	7242	6795	27
H(56)	8473	6580	5110	25
H(6)	11032	5826	7357	29
H(37)	3595	5196	5841	22
H(44)	3162	7647	6381	29
H(34)	6205	6826	7530	28
H(39A)	3727	4150	6203	25
H(39B)	4589	4039	6728	25
H(60)	11539	3530	5949	43
H(19)	7129	4624	8310	25
H(35A)	4902	4847	4957	21
H(35B)	5011	5570	4988	21
H(36A)	6557	5352	4394	24
H(36B)	6900	4731	4698	24
H(21A)	3987	5314	8302	29
H(21B)	5193	5321	8687	29
H(53)	9020	5021	4474	28
H(24)	5917	3608	7754	38
H(30)	4507	6235	8902	37
H(3)	9986	7312	7975	30
H(27)	9255	3986	6598	40
H(64)	6888	6364	3852	27
H(58)	10707	5298	5727	34
H(28)	8633	4719	7230	30
H(20A)	4879	4618	7708	27
H(20B)	5131	4354	8310	27
H(52)	3869	3839	5368	41

H(42)	5437	6226	6667	24
H(33)	6241	7807	7882	39
H(4A)	10756	6978	6896	33
H(4B)	11356	7514	7253	33
H(45)	1919	7042	5832	32
H(46)	2416	6018	5709	28
H(1A)	9355	6336	8649	23
H(1B)	8126	6678	8451	23
H(5A)	12045	6833	7915	41
H(5B)	12459	6550	7339	41
H(48)	7555	3767	5642	42
H(26)	8233	3051	6565	44
H(54A)	10480	5965	4958	34
H(54B)	10688	5666	4365	34
H(25)	6599	2863	7150	45
H(61)	10371	3288	5186	45
H(59)	11676	4537	6229	40
H(55A)	8778	6058	4052	33
H(55B)	9538	6614	4310	33
H(8)	8170	7118	6747	36
H(68)	6499	6910	5424	32
H(32)	5436	7998	8749	52
H(65)	4980	6797	3640	35
H(2A)	7330	5795	8758	25
H(2B)	8497	5410	8553	25
H(66)	3826	7278	4315	43
H(62)	9360	4036	4694	39
H(12)	9169	8262	7954	62
H(31)	4537	7220	9244	52
H(50)	6045	2763	4437	68
H(67)	4580	7324	5211	41
H(10)	6682	8797	6832	79
H(51)	4103	3129	4662	57
H(9)	6815	7851	6404	54
H(11)	7872	8994	7600	86

H(49)	7767	3080	4919	63
H(14)	10714	4922	7834	63
H(18)	11916	6391	8670	41
H(17)	12356	5779	9425	74
H(16)	11975	4738	9385	101
H(15)	11154	4309	8589	96
H(1)	7110(50)	5460(20)	6722(19)	56(15)

6.3 [(Ph-BPE)Rh(μ-H)]$_2$ (6)

Table S19. Sample and crystal data for **6**.

**Identification code**	**6**	
**Chemical formula**	$C_{68}H_{72}P_4Rh_2$	
**Formula weight**	1218.95 g/mol	
**Temperature**	100(2) K	
**Wavelength**	0.71073 Å	
**Crystal size**	0.064 x 0.196 x 0.201 mm	
**Crystal habit**	clear red plate	
**Crystal system**	orthorhombic	
**Space group**	P 21 21 21	
**Unit cell dimensions**	a = 13.1998(4) Å	α = 90°
	b = 18.0969(5) Å	β = 90°
	c = 24.5076(8) Å	γ = 90°
**Volume**	5854.3(3) Å^3	
**Z**	4	
**Density (calculated)**	1.383 g/cm^3	
**Absorption coefficient**	0.714 mm^{-1}	
**F(000)**	2520	

Table S20. Data collection and structure refinement for **6**.

**Theta range for data collection**	1.40 to 26.38°	
**Index ranges**	-16<=h<=16, -22<=k<=20, -30<=l<=15	
**Reflections collected**	27044	
**Independent reflections**	11938 [R(int) = 0.0670]	
**Coverage of independent reflections**	99.8%	
**Max. and min. transmission**	0.9560 and 0.8700	
**Refinement method**	Full-matrix least-squares on F^2	
**Refinement program**	SHELXL-2014/7 (Sheldrick, 2014)	
**Function minimized**	Σ w(F_o^2 - F_c^2)^2	
**Data / restraints / parameters**	11938 / 0 / 667	
**Goodness-of-fit on F^2**	0.701	
**Δ/σ_{max}**	0.001	
**Final R indices**	7779 data; I>2σ(I)	R1 = 0.0427, wR2 = 0.0776
	all data	R1 = 0.0841, wR2 = 0.0912
**Weighting scheme**	w=1/[$\sigma^2(F_o^2)+(0.0483P)^2$] where P=($F_o^2+2F_c^2$)/3	
**Absolute structure parameter**	-0.0(0)	
**Largest diff. peak and hole**	0.973 and -0.572 eÅ^{-3}	
**R.M.S. deviation from mean**	0.095 eÅ^{-3}	

Table S21. Atomic coordinates and equivalent isotropic atomic displacement parameters (Å^2) for **6**

U(eq) is defined as one third of the trace of the orthogonalized U_{ij} tensor.

	x/a	y/b	z/c	U(eq)
Rh1	0.16657(5)	0.03758(3)	0.10878(2)	0.01478(14)
Rh2	0.16916(5)	0.08124(3)	0.21090(2)	0.01417(14)
P1	0.20034(15)	0.08865(10)	0.03039(8)	0.0157(5)
P2	0.16420(18)	0.93463(9)	0.06377(7)	0.0138(4)
P3	0.16118(18)	0.18599(9)	0.25414(7)	0.0153(4)
P4	0.18005(16)	0.03221(9)	0.29213(7)	0.0161(4)
C1	0.1145(6)	0.1651(4)	0.0073(3)	0.0218(19)
C2	0.1841(7)	0.2302(4)	0.9904(3)	0.031(2)
C3	0.2775(6)	0.2265(4)	0.0281(3)	0.025(2)
C4	0.3164(6)	0.1461(4)	0.0219(3)	0.0204(18)
C5	0.4066(6)	0.1275(4)	0.0574(3)	0.0181(18)
C6	0.3979(6)	0.1221(4)	0.1137(3)	0.0220(18)
C7	0.4811(7)	0.1033(4)	0.1451(3)	0.029(2)
C8	0.5745(6)	0.0904(4)	0.1211(4)	0.027(2)
C9	0.5836(6)	0.0965(4)	0.0653(4)	0.028(2)
C10	0.5001(6)	0.1136(4)	0.0335(3)	0.0218(19)
C11	0.0320(6)	0.1435(4)	0.9681(3)	0.0202(18)
C12	0.9625(6)	0.0898(4)	0.9837(3)	0.0226(19)
C13	0.8848(6)	0.0685(4)	0.9487(3)	0.028(2)
C14	0.8747(6)	0.1018(4)	0.8978(3)	0.030(2)
C15	0.9433(7)	0.1551(4)	0.8825(3)	0.029(2)
C16	0.0209(6)	0.1758(4)	0.9174(3)	0.0227(19)
C17	0.2145(6)	0.0205(4)	0.9744(3)	0.0204(18)
C1	0.1614(7)	0.9478(3)	0.9896(3)	0.0181(16)

	x/a	y/b	z/c	U(eq)
8				
C19	0.2681(6)	0.8681(4)	0.0840(3)	0.0170(18)
C20	0.2149(6)	0.7965(4)	0.1032(3)	0.024(2)
C21	0.1063(6)	0.8174(4)	0.1205(3)	0.0210(19)
C22	0.0638(6)	0.8641(4)	0.0746(3)	0.0165(18)
C23	0.9580(6)	0.8947(4)	0.0814(3)	0.0173(18)
C24	0.9294(6)	0.9334(4)	0.1281(3)	0.025(2)
C25	0.8305(8)	0.9593(4)	0.1328(3)	0.0333(19)
C26	0.7603(7)	0.9487(4)	0.0921(4)	0.031(2)
C27	0.7901(7)	0.9108(4)	0.0456(4)	0.028(2)
C28	0.8873(7)	0.8846(4)	0.0398(4)	0.023(2)
C29	0.3515(5)	0.8623(4)	0.0435(3)	0.0145(17)
C30	0.4290(6)	0.9144(4)	0.0433(3)	0.0186(18)
C31	0.5064(6)	0.9122(4)	0.0056(3)	0.0229(18)
C32	0.5091(6)	0.8572(4)	0.9663(3)	0.0234(19)
C33	0.4328(6)	0.8056(4)	0.9649(3)	0.0217(19)
C34	0.3548(6)	0.8082(4)	0.0027(3)	0.0183(18)
C35	0.0753(6)	0.2626(4)	0.2318(3)	0.0194(19)
C46	0.9875(7)	0.2239(5)	0.1447(4)	0.038(2)
C36	0.1468(6)	0.3177(4)	0.2044(3)	0.028(2)
C37	0.2368(6)	0.3256(4)	0.2418(3)	0.023(2)
C3	0.2761(6)	0.2477(4)	0.2545(3)	0.0183(18)

	x/a	y/b	z/c	U(eq)
8				
C39	0.3430(7)	0.2390(4)	0.3029(3)	0.0225(18)
C40	0.4272(7)	0.1929(5)	0.3004(4)	0.039(2)
C44	0.3282(8)	0.2750(4)	0.3524(3)	0.0296(19)
C51	0.1225(6)	0.1714(4)	0.3255(3)	0.0177(18)
C52	0.1748(7)	0.1025(3)	0.3475(3)	0.0202(17)
C53	0.0813(6)	0.9633(4)	0.3118(3)	0.0208(18)
C63	0.9799(6)	0.9964(4)	0.3252(3)	0.023(2)
C68	0.9251(6)	0.0329(4)	0.2847(4)	0.031(2)
C67	0.8309(8)	0.0623(4)	0.2956(4)	0.044(2)
C66	0.7907(7)	0.0575(5)	0.3470(4)	0.049(3)
C65	0.8429(7)	0.0222(5)	0.3875(4)	0.045(2)
C64	0.9379(7)	0.9922(5)	0.3767(3)	0.032(2)
C54	0.1325(6)	0.9152(4)	0.3548(3)	0.026(2)
C55	0.2383(6)	0.8982(4)	0.3336(3)	0.026(2)
C56	0.2872(6)	0.9718(4)	0.3162(3)	0.0225(19)
C57	0.3771(6)	0.9623(4)	0.2791(3)	0.0220(18)
C62	0.4702(6)	0.9896(4)	0.2932(4)	0.028(2)
C61	0.5555(7)	0.9769(4)	0.2612(4)	0.032(2)
C60	0.5476(7)	0.9367(4)	0.2138(4)	0.032(2)
C59	0.4548(7)	0.9081(5)	0.1993(3)	0.039(2)
C5	0.3700(7)	0.9218(5)	0.2308(3)	0.035(2)

	x/a	y/b	z/c	U(eq)
8				
C41	0.4912(8)	0.1827(6)	0.3450(5)	0.061(3)
C45	0.9827(6)	0.2398(4)	0.2003(3)	0.026(2)
C50	0.8901(7)	0.2334(4)	0.2267(4)	0.032(2)
C43	0.3909(8)	0.2657(4)	0.3967(4)	0.040(2)
C42	0.4716(9)	0.2194(5)	0.3928(5)	0.056(3)
C47	0.9009(8)	0.2029(5)	0.1168(4)	0.047(3)
C48	0.8096(8)	0.1972(5)	0.1428(4)	0.044(3)
C49	0.8034(7)	0.2120(4)	0.1982(4)	0.043(3)

Table S22. Bond lengths (Å) for **6**.

Rh1-P2	2.1654(17)	Rh1-P1	2.178(2)
Rh1-Rh2	2.6246(7)	Rh2-P3	2.1743(18)
Rh2-P4	2.1842(18)	P1-C17	1.854(7)
P1-C4	1.863(7)	P1-C1	1.876(8)
P2-C18	1.833(6)	P2-C22	1.859(8)
P2-C19	1.891(8)	P3-C51	1.842(7)
P3-C35	1.874(7)	P3-C38	1.884(8)
P4-C52	1.862(7)	P4-C53	1.867(8)
P4-C56	1.882(8)	C1-C11	1.504(10)
C1-C2	1.550(10)	C1-H1	1.0
C2-C3	1.542(11)	C2-H2A	0.99
C2-H2B	0.99	C3-C4	1.550(10)
C3-H3A	0.99	C3-H3B	0.99
C4-C5	1.513(10)	C4-H4	1.0
C5-C6	1.386(10)	C5-C10	1.390(11)
C6-C7	1.384(10)	C6-H6	0.95
C7-C8	1.386(11)	C7-H7	0.95
C8-C9	1.377(11)	C8-H8	0.95
C9-C10	1.386(11)	C9-H9	0.95
C10-H10	0.95	C11-C16	1.383(10)
C11-C12	1.390(10)	C12-C13	1.391(10)

C12-H12	0.95	C13-C14	1.391(11)
C13-H13	0.95	C14-C15	1.375(11)
C14-H14	0.95	C15-C16	1.385(11)
C15-H15	0.95	C16-H16	0.95
C17-C18	1.537(9)	C17-H17A	0.99
C17-H17B	0.99	C18-H18A	0.99
C18-H18B	0.99	C19-C29	1.485(10)
C19-C20	1.548(10)	C19-H19	1.0
C20-C21	1.542(10)	C20-H20A	0.99
C20-H20B	0.99	C21-C22	1.515(10)
C21-H21A	0.99	C21-H21B	0.99
C22-C23	1.511(10)	C22-H22	1.0
C23-C24	1.394(10)	C23-C28	1.395(11)
C24-C25	1.391(12)	C24-H24	0.95
C25-C26	1.376(12)	C25-H25	0.95
C26-C27	1.387(11)	C26-H26	0.95
C27-C28	1.374(11)	C27-H27	0.95
C28-H28	0.95	C29-C30	1.392(10)
C29-C34	1.400(9)	C30-C31	1.379(10)
C30-H30	0.95	C31-C32	1.386(10)
C31-H31	0.95	C32-C33	1.373(11)
C32-H32	0.95	C33-C34	1.384(10)
C33-H33	0.95	C34-H34	0.95
C35-C45	1.503(10)	C35-C36	1.528(10)
C35-H35	1.0	C46-C47	1.386(12)
C46-C45	1.393(11)	C46-H46	0.95
C36-C37	1.509(11)	C36-H36A	0.99
C36-H36B	0.99	C37-C38	1.534(10)
C37-H37A	0.99	C37-H37B	0.99
C38-C39	1.487(10)	C38-H38	1.0
C39-C40	1.391(11)	C39-C44	1.391(10)
C40-C41	1.394(13)	C40-H40	0.95
C44-C43	1.377(11)	C44-H44	0.95
C51-C52	1.523(9)	C51-H51A	0.99
C51-H51B	0.99	C52-H52A	0.99
C52-H52B	0.99	C53-C63	1.502(11)
C53-C54	1.524(10)	C53-H53	1.0
C63-C64	1.382(11)	C63-C68	1.393(11)
C68-C67	1.379(12)	C68-H68	0.95
C67-C66	1.370(12)	C67-H67	0.95
C66-C65	1.367(13)	C66-H66	0.95

C65-C64	1.392(12)	C65-H65	0.95
C64-H64	0.95	C54-C55	1.522(11)
C54-H54A	0.99	C54-H54B	0.99
C55-C56	1.542(10)	C55-H55A	0.99
C55-H55B	0.99	C56-C57	1.505(11)
C56-H56	1.0	C57-C62	1.370(11)
C57-C58	1.394(10)	C62-C61	1.392(11)
C62-H62	0.95	C61-C60	1.375(11)
C61-H61	0.95	C60-C59	1.377(12)
C60-H60	0.95	C59-C58	1.382(11)
C59-H59	0.95	C58-H58	0.95
C41-C42	1.371(15)	C41-H41	0.95
C45-C50	1.388(12)	C50-C49	1.396(11)
C50-H50	0.95	C43-C42	1.359(13)
C43-H43	0.95	C42-H42	0.95
C47-C48	1.369(14)	C47-H47	0.95
C48-C49	1.385(13)	C48-H48	0.95
C49-H49	0.95		

Table S23. Bond angles (°) for **6**.

P2-Rh1-P1	85.34(7)	P2-Rh1-Rh2	138.15(5)
P1-Rh1-Rh2	135.30(6)	P3-Rh2-P4	85.02(7)
P3-Rh2-Rh1	136.59(5)	P4-Rh2-Rh1	138.39(5)
C17-P1-C4	101.9(3)	C17-P1-C1	109.1(4)
C4-P1-C1	92.9(3)	C17-P1-Rh1	113.1(2)
C4-P1-Rh1	120.2(2)	C1-P1-Rh1	117.1(3)
C18-P2-C22	102.5(4)	C18-P2-C19	110.9(4)
C22-P2-C19	92.5(3)	C18-P2-Rh1	113.2(2)
C22-P2-Rh1	121.9(3)	C19-P2-Rh1	113.8(2)
C51-P3-C35	102.5(4)	C51-P3-C38	107.7(3)
C35-P3-C38	92.9(3)	C51-P3-Rh2	110.6(2)
C35-P3-Rh2	122.1(2)	C38-P3-Rh2	118.7(3)
C52-P4-C53	104.0(3)	C52-P4-C56	101.3(4)
C53-P4-C56	93.2(3)	C52-P4-Rh2	112.6(2)
C53-P4-Rh2	117.5(2)	C56-P4-Rh2	124.8(3)
C11-C1-C2	117.1(6)	C11-C1-P1	115.9(5)
C2-C1-P1	106.4(5)	C11-C1-H1	105.4
C2-C1-H1	105.4	P1-C1-H1	105.4
C3-C2-C1	106.3(6)	C3-C2-H2A	110.5
C1-C2-H2A	110.5	C3-C2-H2B	110.5

C1-C2-H2B	110.5	H2A-C2-H2B	108.7
C2-C3-C4	104.3(6)	C2-C3-H3A	110.9
C4-C3-H3A	110.9	C2-C3-H3B	110.9
C4-C3-H3B	110.9	H3A-C3-H3B	108.9
C5-C4-C3	114.4(6)	C5-C4-P1	117.3(5)
C3-C4-P1	103.9(5)	C5-C4-H4	106.8
C3-C4-H4	106.8	P1-C4-H4	106.8
C6-C5-C10	118.7(7)	C6-C5-C4	121.6(7)
C10-C5-C4	119.7(7)	C7-C6-C5	120.4(8)
C7-C6-H6	119.8	C5-C6-H6	119.8
C6-C7-C8	120.7(8)	C6-C7-H7	119.7
C8-C7-H7	119.7	C9-C8-C7	119.1(8)
C9-C8-H8	120.4	C7-C8-H8	120.4
C8-C9-C10	120.5(8)	C8-C9-H9	119.8
C10-C9-H9	119.8	C9-C10-C5	120.6(8)
C9-C10-H10	119.7	C5-C10-H10	119.7
C16-C11-C12	118.1(7)	C16-C11-C1	122.8(7)
C12-C11-C1	119.1(7)	C11-C12-C13	120.8(7)
C11-C12-H12	119.6	C13-C12-H12	119.6
C12-C13-C14	120.2(8)	C12-C13-H13	119.9
C14-C13-H13	119.9	C15-C14-C13	119.0(8)
C15-C14-H14	120.5	C13-C14-H14	120.5
C14-C15-C16	120.6(7)	C14-C15-H15	119.7
C16-C15-H15	119.7	C11-C16-C15	121.3(8)
C11-C16-H16	119.4	C15-C16-H16	119.4
C18-C17-P1	110.1(5)	C18-C17-H17A	109.6
P1-C17-H17A	109.6	C18-C17-H17B	109.6
P1-C17-H17B	109.6	H17A-C17-H17B	108.2
C17-C18-P2	110.1(5)	C17-C18-H18A	109.6
P2-C18-H18A	109.6	C17-C18-H18B	109.6
P2-C18-H18B	109.6	H18A-C18-H18B	108.2
C29-C19-C20	118.7(6)	C29-C19-P2	114.1(5)
C20-C19-P2	106.5(5)	C29-C19-H19	105.5
C20-C19-H19	105.5	P2-C19-H19	105.5
C21-C20-C19	107.5(6)	C21-C20-H20A	110.2
C19-C20-H20A	110.2	C21-C20-H20B	110.2
C19-C20-H20B	110.2	H20A-C20-H20B	108.5
C22-C21-C20	106.1(7)	C22-C21-H21A	110.5
C20-C21-H21A	110.5	C22-C21-H21B	110.5
C20-C21-H21B	110.5	H21A-C21-H21B	108.7
C23-C22-C21	117.6(7)	C23-C22-P2	115.1(5)

C21-C22-P2	103.0(5)	C23-C22-H22	106.8
C21-C22-H22	106.8	P2-C22-H22	106.8
C24-C23-C28	119.0(7)	C24-C23-C22	121.7(7)
C28-C23-C22	119.3(7)	C25-C24-C23	119.4(8)
C25-C24-H24	120.3	C23-C24-H24	120.3
C26-C25-C24	121.7(8)	C26-C25-H25	119.1
C24-C25-H25	119.1	C25-C26-C27	118.3(8)
C25-C26-H26	120.9	C27-C26-H26	120.9
C28-C27-C26	121.3(8)	C28-C27-H27	119.3
C26-C27-H27	119.3	C27-C28-C23	120.3(8)
C27-C28-H28	119.9	C23-C28-H28	119.9
C30-C29-C34	116.7(7)	C30-C29-C19	119.9(6)
C34-C29-C19	123.4(7)	C31-C30-C29	121.8(7)
C31-C30-H30	119.1	C29-C30-H30	119.1
C30-C31-C32	120.4(8)	C30-C31-H31	119.8
C32-C31-H31	119.8	C33-C32-C31	119.1(8)
C33-C32-H32	120.4	C31-C32-H32	120.4
C32-C33-C34	120.4(7)	C32-C33-H33	119.8
C34-C33-H33	119.8	C33-C34-C29	121.6(7)
C33-C34-H34	119.2	C29-C34-H34	119.2
C45-C35-C36	117.1(7)	C45-C35-P3	116.0(5)
C36-C35-P3	103.7(5)	C45-C35-H35	106.4
C36-C35-H35	106.4	P3-C35-H35	106.4
C47-C46-C45	120.2(9)	C47-C46-H46	119.9
C45-C46-H46	119.9	C37-C36-C35	106.3(6)
C37-C36-H36A	110.5	C35-C36-H36A	110.5
C37-C36-H36B	110.5	C35-C36-H36B	110.5
H36A-C36-H36B	108.7	C36-C37-C38	107.6(6)
C36-C37-H37A	110.2	C38-C37-H37A	110.2
C36-C37-H37B	110.2	C38-C37-H37B	110.2
H37A-C37-H37B	108.5	C39-C38-C37	117.4(6)
C39-C38-P3	114.8(5)	C37-C38-P3	105.7(5)
C39-C38-H38	106.0	C37-C38-H38	106.0
P3-C38-H38	106.0	C40-C39-C44	115.6(8)
C40-C39-C38	120.2(7)	C44-C39-C38	124.2(8)
C39-C40-C41	122.0(9)	C39-C40-H40	119.0
C41-C40-H40	119.0	C43-C44-C39	123.2(9)
C43-C44-H44	118.4	C39-C44-H44	118.4
C52-C51-P3	109.1(5)	C52-C51-H51A	109.9
P3-C51-H51A	109.9	C52-C51-H51B	109.9
P3-C51-H51B	109.9	H51A-C51-H51B	108.3

C51-C52-P4	108.6(5)	C51-C52-H52A	110.0
P4-C52-H52A	110.0	C51-C52-H52B	110.0
P4-C52-H52B	110.0	H52A-C52-H52B	108.4
C63-C53-C54	118.2(7)	C63-C53-P4	114.3(5)
C54-C53-P4	104.5(5)	C63-C53-H53	106.3
C54-C53-H53	106.3	P4-C53-H53	106.3
C64-C63-C68	117.9(8)	C64-C63-C53	122.3(8)
C68-C63-C53	119.8(7)	C67-C68-C63	120.9(8)
C67-C68-H68	119.5	C63-C68-H68	119.5
C66-C67-C68	120.2(9)	C66-C67-H67	119.9
C68-C67-H67	119.9	C65-C66-C67	120.1(9)
C65-C66-H66	119.9	C67-C66-H66	119.9
C66-C65-C64	120.0(9)	C66-C65-H65	120.0
C64-C65-H65	120.0	C63-C64-C65	120.9(9)
C63-C64-H64	119.6	C65-C64-H64	119.6
C55-C54-C53	106.6(6)	C55-C54-H54A	110.4
C53-C54-H54A	110.4	C55-C54-H54B	110.4
C53-C54-H54B	110.4	H54A-C54-H54B	108.6
C54-C55-C56	107.7(6)	C54-C55-H55A	110.2
C56-C55-H55A	110.2	C54-C55-H55B	110.2
C56-C55-H55B	110.2	H55A-C55-H55B	108.5
C57-C56-C55	113.4(6)	C57-C56-P4	118.0(5)
C55-C56-P4	105.9(5)	C57-C56-H56	106.2
C55-C56-H56	106.2	P4-C56-H56	106.2
C62-C57-C58	117.7(8)	C62-C57-C56	120.8(7)
C58-C57-C56	121.4(7)	C57-C62-C61	121.5(8)
C57-C62-H62	119.2	C61-C62-H62	119.2
C60-C61-C62	120.2(8)	C60-C61-H61	119.9
C62-C61-H61	119.9	C61-C60-C59	119.0(9)
C61-C60-H60	120.5	C59-C60-H60	120.5
C60-C59-C58	120.6(8)	C60-C59-H59	119.7
C58-C59-H59	119.7	C59-C58-C57	121.0(8)
C59-C58-H58	119.5	C57-C58-H58	119.5
C42-C41-C40	119.4(10)	C42-C41-H41	120.3
C40-C41-H41	120.3	C50-C45-C46	118.7(8)
C50-C45-C35	119.9(8)	C46-C45-C35	121.4(8)
C45-C50-C49	120.7(8)	C45-C50-H50	119.6
C49-C50-H50	119.6	C42-C43-C44	119.4(9)
C42-C43-H43	120.3	C44-C43-H43	120.3
C43-C42-C41	120.5(10)	C43-C42-H42	119.8
C41-C42-H42	119.8	C48-C47-C46	121.1(9)

C48-C47-H47	119.5	C46-C47-H47	119.5
C47-C48-C49	119.6(9)	C47-C48-H48	120.2
C49-C48-H48	120.2	C48-C49-C50	119.8(9)
C48-C49-H49	120.1	C50-C49-H49	120.1

Table S24. Torsion angles (°) for **6**.

C17-P1-C1-C11	32.5(7)	C4-P1-C1-C11	136.1(6)
Rh1-P1-C1-C11	-97.6(6)	C17-P1-C1-C2	-99.8(6)
C4-P1-C1-C2	3.8(6)	Rh1-P1-C1-C2	130.2(5)
C11-C1-C2-C3	-164.2(7)	P1-C1-C2-C3	-32.6(7)
C1-C2-C3-C4	52.9(8)	C2-C3-C4-C5	-177.5(6)
C2-C3-C4-P1	-48.2(7)	C17-P1-C4-C5	-96.9(6)
C1-P1-C4-C5	152.8(6)	Rh1-P1-C4-C5	28.9(7)
C17-P1-C4-C3	135.7(5)	C1-P1-C4-C3	25.4(5)
Rh1-P1-C4-C3	-98.5(5)	C3-C4-C5-C6	69.4(9)
P1-C4-C5-C6	-52.7(8)	C3-C4-C5-C10	-112.3(8)
P1-C4-C5-C10	125.6(6)	C10-C5-C6-C7	0.2(11)
C4-C5-C6-C7	178.5(7)	C5-C6-C7-C8	0.7(11)
C6-C7-C8-C9	-0.1(12)	C7-C8-C9-C10	-1.3(12)
C8-C9-C10-C5	2.2(11)	C6-C5-C10-C9	-1.6(11)
C4-C5-C10-C9	-179.9(6)	C2-C1-C11-C16	4.2(11)
P1-C1-C11-C16	-122.9(7)	C2-C1-C11-C12	-174.4(7)
P1-C1-C11-C12	58.5(9)	C16-C11-C12-C13	1.0(11)
C1-C11-C12-C13	179.7(7)	C11-C12-C13-C14	-1.2(12)
C12-C13-C14-C15	0.9(12)	C13-C14-C15-C16	-0.5(12)
C12-C11-C16-C15	-0.6(12)	C1-C11-C16-C15	-179.3(7)
C14-C15-C16-C11	0.4(12)	C4-P1-C17-C18	151.7(5)
C1-P1-C17-C18	-110.9(6)	Rh1-P1-C17-C18	21.3(6)
P1-C17-C18-P2	-30.1(7)	C22-P2-C18-C17	162.2(5)
C19-P2-C18-C17	-100.2(5)	Rh1-P2-C18-C17	29.1(6)
C18-P2-C19-C29	23.3(7)	C22-P2-C19-C29	127.6(6)
Rh1-P2-C19-C29	-105.7(5)	C18-P2-C19-C20	-109.6(5)
C22-P2-C19-C20	-5.3(5)	Rh1-P2-C19-C20	121.4(5)
C29-C19-C20-C21	-152.9(7)	P2-C19-C20-C21	-22.6(7)
C19-C20-C21-C22	48.3(8)	C20-C21-C22-C23	-178.1(6)
C20-C21-C22-P2	-50.4(6)	C18-P2-C22-C23	-86.9(6)
C19-P2-C22-C23	161.0(6)	Rh1-P2-C22-C23	40.8(7)
C18-P2-C22-C21	143.8(5)	C19-P2-C22-C21	31.7(5)
Rh1-P2-C22-C21	-88.5(5)	C21-C22-C23-C24	51.2(10)
P2-C22-C23-C24	-70.5(8)	C21-C22-C23-C28	-129.5(8)

P2-C22-C23-C28	108.9(7)	C28-C23-C24-C25	1.7(11)
C22-C23-C24-C25	-178.9(7)	C23-C24-C25-C26	-1.0(12)
C24-C25-C26-C27	0.4(12)	C25-C26-C27-C28	-0.5(12)
C26-C27-C28-C23	1.2(12)	C24-C23-C28-C27	-1.8(11)
C22-C23-C28-C27	178.8(7)	C20-C19-C29-C30	-150.6(7)
P2-C19-C29-C30	82.7(8)	C20-C19-C29-C34	32.5(10)
P2-C19-C29-C34	-94.2(7)	C34-C29-C30-C31	-1.2(10)
C19-C29-C30-C31	-178.3(7)	C29-C30-C31-C32	-0.1(11)
C30-C31-C32-C33	1.0(11)	C31-C32-C33-C34	-0.6(11)
C32-C33-C34-C29	-0.7(11)	C30-C29-C34-C33	1.6(10)
C19-C29-C34-C33	178.6(7)	C51-P3-C35-C45	-98.4(6)
C38-P3-C35-C45	152.8(6)	Rh2-P3-C35-C45	26.0(7)
C51-P3-C35-C36	131.7(5)	C38-P3-C35-C36	22.9(5)
Rh2-P3-C35-C36	-103.9(5)	C45-C35-C36-C37	-174.9(6)
P3-C35-C36-C37	-45.7(7)	C35-C36-C37-C38	51.9(8)
C36-C37-C38-C39	-161.8(7)	C36-C37-C38-P3	-32.2(7)
C51-P3-C38-C39	31.8(6)	C35-P3-C38-C39	135.9(6)
Rh2-P3-C38-C39	-94.7(6)	C51-P3-C38-C37	-99.3(5)
C35-P3-C38-C37	4.8(5)	Rh2-P3-C38-C37	134.2(4)
C37-C38-C39-C40	-139.2(8)	P3-C38-C39-C40	95.6(8)
C37-C38-C39-C44	40.3(11)	P3-C38-C39-C44	-85.0(8)
C44-C39-C40-C41	1.1(13)	C38-C39-C40-C41	-179.4(8)
C40-C39-C44-C43	-0.9(12)	C38-C39-C44-C43	179.7(7)
C35-P3-C51-C52	171.6(5)	C38-P3-C51-C52	-91.2(6)
Rh2-P3-C51-C52	39.9(6)	P3-C51-C52-P4	-37.6(6)
C53-P4-C52-C51	-105.4(6)	C56-P4-C52-C51	158.3(5)
Rh2-P4-C52-C51	22.8(6)	C52-P4-C53-C63	50.4(6)
C56-P4-C53-C63	152.9(6)	Rh2-P4-C53-C63	-74.8(6)
C52-P4-C53-C54	-80.4(6)	C56-P4-C53-C54	22.2(5)
Rh2-P4-C53-C54	154.5(4)	C54-C53-C63-C64	8.1(11)
P4-C53-C63-C64	-115.6(7)	C54-C53-C63-C68	-172.0(7)
P4-C53-C63-C68	64.3(8)	C64-C63-C68-C67	-1.5(11)
C53-C63-C68-C67	178.6(7)	C63-C68-C67-C66	1.5(13)
C68-C67-C66-C65	-1.2(13)	C67-C66-C65-C64	1.0(13)
C68-C63-C64-C65	1.3(11)	C53-C63-C64-C65	-178.8(7)
C66-C65-C64-C63	-1.1(12)	C63-C53-C54-C55	-172.6(6)
P4-C53-C54-C55	-44.2(7)	C53-C54-C55-C56	50.1(8)
C54-C55-C56-C57	-162.3(6)	C54-C55-C56-P4	-31.3(7)
C52-P4-C56-C57	-121.8(6)	C53-P4-C56-C57	133.2(6)
Rh2-P4-C56-C57	6.2(7)	C52-P4-C56-C55	109.9(5)
C53-P4-C56-C55	4.9(5)	Rh2-P4-C56-C55	-122.1(5)

C55-C56-C57-C62	-121.8(8)	P4-C56-C57-C62	113.6(7)
C55-C56-C57-C58	54.5(10)	P4-C56-C57-C58	-70.2(9)
C58-C57-C62-C61	-0.8(12)	C56-C57-C62-C61	175.6(7)
C57-C62-C61-C60	0.6(12)	C62-C61-C60-C59	-1.4(12)
C61-C60-C59-C58	2.5(13)	C60-C59-C58-C57	-2.7(13)
C62-C57-C58-C59	1.9(12)	C56-C57-C58-C59	-174.5(8)
C39-C40-C41-C42	-0.5(16)	C47-C46-C45-C50	0.6(12)
C47-C46-C45-C35	179.9(7)	C36-C35-C45-C50	-138.3(8)
P3-C35-C45-C50	98.5(8)	C36-C35-C45-C46	42.3(10)
P3-C35-C45-C46	-80.8(9)	C46-C45-C50-C49	-0.5(12)
C35-C45-C50-C49	-179.9(7)	C39-C44-C43-C42	0.0(13)
C44-C43-C42-C41	0.6(14)	C40-C41-C42-C43	-0.4(16)
C45-C46-C47-C48	-0.1(14)	C46-C47-C48-C49	-0.6(14)
C47-C48-C49-C50	0.7(13)	C45-C50-C49-C48	-0.1(13)

Table S25. Anisotropic atomic displacement parameters (Å^2) for **6**. The anisotropic atomic displacement factor exponent takes the form: $-2\pi^2[h^2 a^{*2} U_{11} + ... + 2 h k a^* b^* U_{12}]$.

	**U_{11}**	**U_{22}**	**U_{33}**	**U_{23}**	**U_{13}**	**U_{12}**
Rh1	0.0203(3)	0.0113(3)	0.0127(3)	-0.0008(2)	0.0000(3)	0.0004(3)
Rh2	0.0186(3)	0.0110(3)	0.0129(3)	-0.0014(2)	-0.0003(3)	-0.0003(3)
P1	0.0195(11)	0.0141(10)	0.0135(10)	-0.0005(9)	0.0007(9)	-0.0003(9)
P2	0.0149(9)	0.0126(9)	0.0139(9)	-0.0014(7)	-0.0013(11)	-0.0008(10)
P3	0.0185(11)	0.0124(9)	0.0150(9)	-0.0007(7)	-0.0003(11)	0.0002(11)
P4	0.0213(11)	0.0143(9)	0.0126(9)	-0.0005(9)	-0.0022(10)	-0.0034(10)
C1	0.021(5)	0.021(4)	0.024(5)	0.001(4)	-0.003(4)	0.001(4)
C2	0.036(6)	0.015(4)	0.040(5)	0.006(4)	-0.013(5)	-0.003(4)
C3	0.023(5)	0.012(4)	0.040(5)	0.002(4)	-0.010(4)	-0.002(4)
C4	0.021(5)	0.022(4)	0.018(4)	0.001(3)	-0.002(4)	-0.012(4)
C5	0.022(5)	0.007(4)	0.025(5)	-0.002(3)	-0.003(4)	-0.008(3)
C6	0.016(4)	0.022(4)	0.028(5)	-0.003(4)	-0.001(4)	0.002(3)
C7	0.036(5)	0.026(5)	0.024(5)	0.001(4)	-0.012(4)	0.000(4)
C8	0.014(4)	0.021(4)	0.047(6)	0.006(4)	-0.009(4)	0.004(4)
C9	0.017(5)	0.013(4)	0.053(6)	0.005(4)	0.007(5)	0.000(4)
C10	0.025(5)	0.014(4)	0.026(5)	-0.002(4)	0.004(4)	-0.002(4)
C11	0.026(5)	0.017(4)	0.018(4)	0.000(3)	0.001(4)	-0.002(4)
C12	0.023(4)	0.021(4)	0.025(4)	0.003(4)	-0.006(4)	0.002(4)
C13	0.024(5)	0.020(4)	0.041(5)	0.002(4)	-0.005(4)	-0.005(4)
C14	0.027(5)	0.032(5)	0.030(5)	-0.008(4)	-0.012(4)	0.003(4)
C15	0.038(5)	0.032(5)	0.016(4)	0.001(4)	-0.005(4)	0.010(4)
C16	0.020(5)	0.030(5)	0.018(4)	0.003(4)	0.002(4)	0.001(4)

	**U_{11}**	**U_{22}**	**U_{33}**	**U_{23}**	**U_{13}**	**U_{12}**
C17	0.020(4)	0.024(4)	0.017(4)	-0.002(3)	0.002(4)	0.002(4)
C18	0.023(4)	0.013(4)	0.019(4)	-0.005(3)	-0.008(4)	0.005(4)
C19	0.024(5)	0.012(4)	0.015(4)	-0.003(3)	-0.001(4)	0.003(4)
C20	0.026(5)	0.018(4)	0.029(5)	0.000(4)	-0.002(4)	0.000(4)
C21	0.022(5)	0.012(4)	0.029(5)	0.005(4)	-0.004(4)	0.001(4)
C22	0.015(4)	0.021(4)	0.013(4)	-0.001(3)	0.003(4)	-0.003(4)
C23	0.015(4)	0.012(4)	0.025(4)	0.003(3)	0.001(4)	-0.005(3)
C24	0.023(5)	0.020(4)	0.034(5)	-0.003(4)	0.005(4)	0.000(4)
C25	0.037(5)	0.027(4)	0.035(5)	-0.002(4)	0.015(6)	0.006(6)
C26	0.023(5)	0.022(5)	0.050(6)	0.011(4)	0.005(5)	0.012(4)
C27	0.023(5)	0.030(5)	0.032(5)	0.009(4)	-0.005(4)	0.007(4)
C28	0.027(5)	0.023(5)	0.020(5)	0.000(4)	0.002(4)	-0.004(4)
C29	0.011(5)	0.019(4)	0.014(4)	0.003(3)	-0.001(3)	0.004(3)
C30	0.020(4)	0.013(4)	0.023(4)	-0.002(4)	-0.005(4)	0.002(4)
C31	0.017(4)	0.019(4)	0.033(5)	0.005(4)	0.000(4)	-0.006(4)
C32	0.019(5)	0.024(4)	0.027(5)	0.006(4)	0.005(4)	0.011(4)
C33	0.029(5)	0.019(4)	0.017(4)	-0.006(4)	-0.002(4)	0.008(4)
C34	0.021(5)	0.013(4)	0.021(4)	-0.001(3)	-0.005(4)	0.002(3)
C35	0.019(4)	0.014(4)	0.025(5)	-0.005(4)	-0.007(4)	0.007(4)
C46	0.028(5)	0.046(6)	0.040(6)	-0.014(5)	-0.013(5)	0.012(5)
C36	0.038(6)	0.014(4)	0.032(5)	0.004(4)	-0.011(5)	-0.001(4)
C37	0.034(5)	0.012(4)	0.024(5)	0.006(3)	-0.001(4)	-0.001(4)
C38	0.018(4)	0.017(4)	0.020(4)	0.000(3)	-0.002(4)	0.000(4)
C39	0.028(5)	0.013(4)	0.027(4)	0.002(3)	-0.008(5)	-0.006(4)
C40	0.034(5)	0.032(5)	0.051(7)	-0.007(5)	-0.015(5)	0.006(4)
C44	0.043(5)	0.023(4)	0.023(4)	-0.002(3)	-0.008(5)	-0.011(5)
C51	0.021(4)	0.017(4)	0.015(4)	-0.004(3)	0.001(4)	-0.004(4)
C52	0.021(4)	0.019(4)	0.021(4)	-0.005(3)	0.000(4)	-0.002(4)
C53	0.027(5)	0.024(4)	0.012(4)	-0.006(4)	-0.004(4)	-0.005(4)
C63	0.028(5)	0.019(4)	0.022(5)	-0.002(4)	0.005(4)	-0.012(4)
C68	0.020(5)	0.030(5)	0.044(6)	0.006(5)	0.007(4)	-0.003(4)
C67	0.033(5)	0.035(5)	0.064(6)	0.015(5)	-0.005(7)	-0.004(5)
C66	0.026(5)	0.055(7)	0.066(8)	-0.018(6)	0.006(6)	-0.012(5)
C65	0.025(5)	0.073(7)	0.036(5)	-0.024(5)	0.008(5)	-0.021(6)
C64	0.027(5)	0.043(5)	0.026(5)	-0.007(4)	-0.004(4)	-0.016(4)
C54	0.035(5)	0.023(4)	0.019(4)	0.003(4)	-0.001(4)	-0.007(4)
C55	0.035(5)	0.019(5)	0.026(5)	0.002(4)	-0.007(4)	-0.001(4)
C56	0.034(5)	0.011(4)	0.023(4)	0.005(4)	-0.005(4)	-0.001(4)
C57	0.028(5)	0.018(4)	0.020(4)	0.001(4)	-0.002(4)	0.004(4)
C62	0.035(5)	0.021(4)	0.029(5)	0.000(4)	-0.010(5)	0.003(4)

	U_{11}	U_{22}	U_{33}	U_{23}	U_{13}	U_{12}
C61	0.022(5)	0.026(5)	0.048(6)	0.007(4)	-0.004(5)	0.008(4)
C60	0.030(5)	0.034(5)	0.033(5)	0.009(4)	0.005(5)	0.015(4)
C59	0.038(6)	0.054(6)	0.026(5)	-0.008(5)	-0.006(5)	0.023(5)
C58	0.025(5)	0.054(6)	0.025(4)	-0.007(5)	-0.006(4)	0.005(5)
C41	0.049(7)	0.039(6)	0.095(10)	-0.005(6)	-0.042(8)	0.016(6)
C45	0.028(5)	0.015(4)	0.034(5)	-0.005(4)	-0.010(5)	0.006(4)
C50	0.035(6)	0.025(5)	0.035(6)	0.002(4)	-0.006(5)	0.002(4)
C43	0.064(7)	0.020(5)	0.036(6)	-0.001(4)	-0.018(6)	-0.014(5)
C42	0.071(8)	0.029(6)	0.069(8)	0.008(6)	-0.041(7)	-0.020(6)
C47	0.055(7)	0.035(6)	0.050(7)	-0.016(5)	-0.027(6)	0.013(5)
C48	0.041(7)	0.026(5)	0.064(7)	0.000(5)	-0.032(6)	0.003(5)
C49	0.031(6)	0.029(5)	0.068(8)	0.011(5)	-0.014(6)	-0.006(4)

Table S26. Hydrogen atomic coordinates and isotropic atomic displacement parameters (Å^2) for **6**.

	x/a	y/b	z/c	U(eq)
H1	0.0787	0.1822	0.0410	0.026
H2A	0.2047	0.2252	-0.0483	0.037
H2B	0.1484	0.2779	-0.0050	0.037
H3A	0.2585	0.2370	0.0664	0.03
H3B	0.3299	0.2623	0.0164	0.03
H4	0.3388	0.1402	-0.0168	0.024
H6	0.3345	0.1312	0.1307	0.026
H7	0.4741	0.0992	0.1836	0.034
H8	0.6315	0.0777	0.1429	0.032
H9	0.6476	0.0888	0.0485	0.033
H10	0.5068	0.1159	-0.0051	0.026
H12	-0.0320	0.0674	0.0186	0.027
H13	-0.1615	0.0311	-0.0404	0.034
H14	-0.1788	0.0880	-0.1260	0.036
H15	-0.0625	0.1779	-0.1523	0.034
H16	0.0673	0.2129	-0.0938	0.027

	x/a	y/b	z/c	U(eq)
H17A	0.1844	0.0407	-0.0595	0.024
H17B	0.2873	0.0111	-0.0324	0.024
H18A	0.1959	-0.0941	-0.0285	0.022
H18B	0.0902	-0.0509	-0.0232	0.022
H19	0.2997	-0.1102	0.1174	0.02
H20A	0.2132	-0.2401	0.0733	0.029
H20B	0.2520	-0.2253	0.1344	0.029
H21A	0.0647	-0.2275	0.1258	0.025
H21B	0.1072	-0.1542	0.1550	0.025
H22	0.0627	-0.1677	0.0412	0.02
H24	-0.0230	-0.0580	0.1565	0.031
H25	-0.1890	-0.0150	0.1649	0.04
H26	-0.3068	-0.0331	0.0957	0.038
H27	-0.2576	-0.0973	0.0172	0.034
H28	-0.0936	-0.1404	0.0073	0.028
H30	0.4286	-0.0474	0.0700	0.022
H31	0.5582	-0.0514	0.0065	0.027
H32	0.5630	-0.1449	-0.0593	0.028
H33	0.4336	-0.2320	-0.0621	0.026
H34	0.3023	-0.2276	0.0008	0.022
H35	0.0503	0.2871	0.2658	0.023
H46	0.0503	0.2275	0.1260	0.046
H36A	0.1684	0.2990	0.1682	0.033
H36B	0.1127	0.3659	0.1993	0.033
H37A	0.2165	0.3509	0.2759	0.028
H37B	0.2904	0.3552	0.2239	0.028
H38	0.3176	0.2327	0.2223	0.022
H40	0.4415	0.1676	0.2673	0.047
H44	0.2721	0.3076	0.3557	0.035
H51A	0.1416	0.2149	0.3478	0.021
H51B	0.0481	0.1653	0.3276	0.021
H52A	0.1366	0.0828	0.3791	0.024
H52B	0.2442	0.1149	0.3598	0.024
H53	0.0707	-0.0690	0.2792	0.025
H68	-0.0469	0.0375	0.2491	0.038
H67	-0.2062	0.0860	0.2673	0.053
H66	-0.2737	0.0787	0.3545	0.059
H65	-0.1856	0.0183	0.4229	0.053
H64	-0.0256	-0.0315	0.4052	0.038
H54A	0.1365	-0.0582	0.3901	0.031

	x/a	y/b	z/c	U(eq)
H54B	0.0938	-0.1310	0.3603	0.031
H55A	0.2343	-0.1359	0.3021	0.032
H55B	0.2792	-0.1256	0.3625	0.032
H56	0.3136	-0.0048	0.3504	0.027
H62	0.4767	0.0179	0.3257	0.034
H61	0.6193	-0.0039	0.2721	0.038
H60	0.6053	-0.0711	0.1913	0.039
H59	0.4490	-0.1214	0.1674	0.047
H58	0.3060	-0.0965	0.2194	0.042
H41	0.5479	0.1505	0.3423	0.074
H50	-0.1143	0.2436	0.2647	0.038
H43	0.3779	0.2914	0.4298	0.048
H42	0.5148	0.2124	0.4234	0.068
H47	-0.0949	0.1923	0.0789	0.056
H48	-0.2493	0.1832	0.1231	0.053
H49	-0.2596	0.2077	0.2166	0.051

7. References

(1) Field, L. D.; Messerie, B. A.; Rehr, M.; Soler, L. P.; Hambley, T. W. *Organometallics* **2003**, *22*, 2387–2395.
(2) Mochida, K.; Kimijima, K.; Wakasa, M.; Hayashi, H. *J. Organomet. Chem.* **1994**, *465*, 101–107.
(3) Grande, J. B.; Thompson, D. B.; Gonzaga, F.; Brook, M. A. *Chem. Commun.* **2010,** *46*, 4988–4990.
